# From data to action: addressing geographic inequalities in rabies burden through targeted policies

**DOI:** 10.3389/fpubh.2025.1683863

**Published:** 2025-10-21

**Authors:** Lu He, En-Li Tan

**Affiliations:** ^1^School of Global Health, Chinese Center for Tropical Diseases Research, Shanghai Jiao Tong University School of Medicine, Shanghai, China; ^2^The First Hospital of Lanzhou University, Lanzhou, China

**Keywords:** rabies, global burden of disease, health inequality, Bayesian age-period-cohort, disability-adjusted life-years

## Abstract

**Objective:**

Rabies remains a preventable yet fatal zoonotic disease, disproportionately affecting low- and middle-income settings. Despite global progress in prevention and control, the extent of geographic, socioeconomic, and age-related disparities in rabies burden has not been fully quantified.

**Methods:**

Using data from the Global Burden of Disease (GBD) Study 2021, we evaluated global, regional, national, and sociodemographic index (SDI)-specific patterns in rabies burden, focusing on age-standardized rate (ASR), including age-standardized incidence (ASIR), prevalence (ASPR), mortality (ASMR), and disability-adjusted life-year rates (ASDR). Temporal trends were quantified using the average annual percentage change (AAPC). Health inequalities were examined using the slope index of inequality (SII) and concentration index (CCI), while frontier analysis was conducted to estimate potential gains relative to socioeconomic development.

**Results:**

In 2021, the global ASIR of rabies was 0.129 per 100,000 population [95% uncertainty interval (*UI*): 0.076, 0.182 per 100,000 population], the ASMR was 0.128 per 100,000 population (95% *UI*: 0.075, 0.181 per 100,000 population). All global ASRs declined from 1990 to 2021, yet the absolute burden remained concentrated in low-SDI countries. Marked inequalities persisted, with ASIR, ASMR, and ASDR highest in low-SDI regions and lowest in high-SDI regions, South Asia carried the greatest absolute number of deaths and disability-adjusted life-year (DALYs) cases, whereas Eastern Sub-Saharan Africa recorded the highest ASR. At the country level, Nepal showed the highest ASR, while India contributed the largest number of cases. Health inequality analysis demonstrated that the absolute gap in ASDR between the highest- and lowest-SDI countries narrowed substantially from 1990 to 2021, but relative inequalities remained stable. Frontier analysis revealed wide substantial room for improvement even in some low-resource and high-income settings.

**Conclusion:**

Global rabies elimination requires comprehensive efforts to reduce disparities both between and within countries, primarily by expanding access to post-exposure prophylaxis, increasing dog vaccination coverage and addressing socioeconomic inequalities.

## 1 Introduction

Rabies is an acute and fatal zoonotic disease caused by the rabies virus (1–3). It is primarily transmitted through contact with the saliva of infected animals via scratches, bites, or direct exposure to mucous membranes (4, 5). Dogs are responsible for up to 99% of human rabies cases. Early symptoms include fever, pain, and neuropathic manifestations. Without timely post-exposure prophylaxis, rabies is nearly universally fatal. The disease has a prolonged incubation period, typically ranging from 1 to 3 months, during which prophylactic vaccination is nearly 100% effective at preventing the onset of clinical disease (1, 2, 6).

According to estimates from the World Health Organization (WHO), rabies causes approximately USD8.6 billion in global losses annually, encompassing the loss of life, livelihoods, healthcare costs, and the immeasurable psychological burden. Rabies is present on all continents except Antarctica, with an estimated 59,000 deaths worldwide each year (7). However, due to underreporting, the documented cases often differ from these estimates. Rabies is classified as a neglected tropical disease, disproportionately affecting marginalized populations. Despite the availability of highly effective human rabies vaccines and immunoglobulins, access remains limited for those in need, often due to affordability (7, 8). The average cost of post-exposure prophylaxis (PEP) is estimated at USD108, including travel expenses and lost income, posing a significant economic burden for individuals earning only USD1–2 per day. Globally, over 29 million people receive human rabies vaccines annually (7).

Studying the global burden of rabies is essential for understanding its epidemiology, health impact, and economic burden. As a nearly 100% fatal yet entirely preventable infectious disease, rabies remains a significant public health challenge in low- and middle-income countries, particularly in Asia and Africa (9, 10). Although previous studies have examined the global burden of rabies (11), they are often limited to specific time periods and regions, lacking comprehensive analyses of major rabies trends and associated health inequalities. In particular, studies utilizing the most recent data from the 2021 Global Burden of Diseases (GBD), Injuries, and Risk Factors Study remain scarce. GBD 2021 systematically analyzes and integrates global health and disease data, serving as a critical tool for assessing the global, regional, and national burden of diseases and injuries (12–14).

This study provides a comprehensive analysis of the burden of rabies. Health inequalities between countries and regions are evaluated using the slope index of inequality (SII) and concentration index (CCI) (15, 16). Frontier analysis estimates optimal health outcomes based on the sociodemographic index (SDI), while decomposition analysis identifies the main contributors to changes in disease burden (15, 16). Joinpoint regression analysis detects key inflection points in temporal trends (15, 16). Additionally, projections based on the Bayesian age-period-cohort (BAPC) model aim to identify potential public health challenges by 2035, offering policymakers a framework to optimize global health resource allocation and develop proactive intervention strategies (15, 16). Such analyses provide critical evidence for developing evidence-based rabies control strategies and resource allocation. Furthermore, they support the implementation of integrated policies for vaccination, dog population management, and post-exposure interventions, aligning with the WHO’s 2030 target of achieving zero human deaths from rabies.

## 2 Materials and methods

### 2.1 Date source

The GBD 2021 study assessed the burden of 371 diseases and injuries, along with 88 risk factors, across 204 countries and territories. Rates and counts of incidence, prevalence, mortality, and Disability-Adjusted Life Years (DALYs) were estimated using stratified models based on various classifications. The analysis systematically adjusted epidemiological data to account for biases arising from variations in data sources, definitions, and measurement methods (13, 14, 17). These adjustments were implemented through sophisticated statistical models, including Bayesian priors, regularization, and trimming using the MR-BRT (stands for meta-regression with Bayesian priors, regularization, and trimming) framework, as well as DisMod-MR 2.1 (a Bayesian mixed-effects meta-regression modeling tool developed for GBD analyses), ensuring internal consistency across estimates for global, five SDI regions, 7 super regions, 21 geographic regions, and 204 countries and territories, age groups, sexes, and time periods (13, 14, 17). The process employed standardization and calibration steps to minimize the impact of heterogeneity on study outcomes. Mortality data in GBD 2021 primarily originated from national vital registration systems, maternal and child health surveillance networks, and census data. Incidence and prevalence data were derived from disease surveillance systems, national health surveys, and published studies. Disability burden estimates were based on data from case notifications, hospital discharge records, household surveys, and cohort studies (13, 14, 17). Detailed information on study design, data collection, and estimation methodologies is provided in the GBD 2021 documentation (13, 14, 17).

Data on rabies were retrieved from the GBD 2021database using the GBD results tool on the Institute for Health Metrics and Evaluation (IHME) website (http://ghdx.healthdata.org), including rabies-related incidence, prevalence, mortality, and DALYs cases, alongside age-standardized incidence rate (ASIR), prevalence (ASPR), mortality (ASMR), and DALYs rate (ASDR) at global, super-regional, regional (21 geographic regions), and national (204 countries and territories) levels. For rabies, the relevant classification code in the WHO’s International Classification of Diseases (ICD-10) is B82 (13).

The SDI is a composite measure of social and demographic development widely used to compare health across settings. Countries and territories were grouped into five levels (low: 0–0.4658, low-middle: 0.4658–0.6188, middle: 0.6188–0.7120, high-middle: 0.7120–0.8103, high: 0.8103–1.0000), facilitating systematic assessment of socioeconomic influences on health outcomes (12, 13).

### 2.2 Statistical analysis

The disease burden of rabies was assessed using rates and total case counts for incidence, prevalence, mortality, and DALYs. Rate was expressed as estimates per 100,000 population, reflect the relative burden, while case counts represent the absolute burden. Both metrics are reported with 95% uncertainty intervals (*UI*) (16, 18, 19).

All statistical analyses were conducted using R software (version 4.4.1, R Foundation for Statistical Computing, Vienna, Austria; available at https://cran.r-project.org). Detailed descriptions of specific analytical methods, including BAPC models, the SII and CCI of inequality analysis, frontier analysis, and decomposition analysis, will be provided in subsequent sections (15, 16, 18).

#### 2.2.1 Percentage change

The percentage change (PC) quantifies the changes in rabies incidence, prevalence, mortality, and DALYs, as well as in ASIR, ASPR, ASMR, and ASDR, by comparing 2021 values to those of 1990. The calculation for PC is as follows (18, 20):

PC = (value_2021−_value_1990_)/value_2021_ × 100%. When the lower bound of the 95% *UI* for the PC is greater than 0, it indicates an increasing trend. Conversely, if the upper bound of the 95% UI is less than 0, it indicates a decreasing trend (20).

#### 2.2.2 Joinpoint regression analysis

The average annual percent change (AAPC) was used to evaluate the overall temporal trends in rabies burden indicators from 1990 to 2021. The formula for calculating AAPC is as follows (21, 22):


$$APC_{i}=\left\{\exp\left(\beta i\right)-1\right\}\times 100$$

$$AAPC_{i}=\left\{\exp\left(\frac{\sum W_{i}\beta_{i}}{\sum W_{i}}\right)-1\right\}\times 100$$

The number and location of joinpoints were identified using the Grid Search Method. In the model, i represents the number of segments, while $\beta_{i}$ denotes the regression coefficients for each linear segment. The length of each segment is represented by $W_{i}$. If the lower bound of the 95% Confidence Interval (*CI*) for the AAPC is greater than 0 and *p* < 0.05, this indicates an increasing trend. Conversely, if the upper bound of the 95% *CI* is less than 0 and *p* < 0.05, this indicates a decreasing trend (21, 22).

#### 2.2.3 BAPC

BAPC model was employed to project global rabies rates for the period 2022–2035. The calculation formula is as follows (19, 21):


$$\log\left(\lambda_{ij}\right)=\alpha +\mu_{i}+\beta_{j}+\gamma_{k}.$$

In this model, i(1≤i≤I) represents time points, j(1≤j≤J) denotes age groups, α represents the intercept, $\mu_{i}$ represents the age effect, $\beta_{j}$ represents the period effect, $\gamma_{k}$ represents the cohort effect.

#### 2.2.4 Estimated annual percent change

The Estimated Annual Percent Change (EAPC) was utilized to quantify the annual trend of the age-standardized rate (ASR, including ASIR, ASPR, ASMR, ASDR). The formula for calculating EAPC is as follows (23, 24):


y=α+βx.

where y represents the natural logarithm of the ASR, x signifies the calendar year. If the lower bound of the 95% *CI* for the EAPC is greater than 0 with and *p* < 0.05, it indicates an increasing trend. Conversely, if the upper bound of the 95% *CI* is less than 0 with and *p* < 0.05, it indicates a decreasing trend (23, 25).

#### 2.2.5 Decomposition analysis

The Das Gupta decomposition method was employed to analyze changes in rabies DALYs cases from 1990 to 2021, with these changes decomposed into three key contributing factors: aging, population growth, and epidemiological changes. Unlike traditional approaches (e.g., linear regression), which primarily explore relationships between variables, decomposition analysis was applied to enable a detailed assessment of the independent contribution of each factor to changes in DALYs cases. Through this deconstruction, a deeper understanding of the core drivers behind changes in rabies DALYs cases was achieved, thereby providing valuable insights for targeted interventions and policy development (15).

#### 2.2.6 Cross-country inequality analysis

The study quantified the absolute and relative inequalities in rabies ASDR using the SII and CCI, as defined by the WHO. The SII was calculated by regressing ASDR against SDI, using the midpoint of the cumulative population distribution ranked by SDI (15). In addition, the CCI is calculated by matching the cumulative proportion of ASDR with the cumulative population distribution ranked by SDI and numerically integrating the area under the Lorenz curve (15).

#### 2.2.7 Frontier analysis

To evaluate the association between rabies disease burden and socioeconomic development, frontier analysis was conducted to construct an ASDR-based model using SDI. A locally weighted regression combined with local polynomial regression was applied, with smoothing spans of 0.3, 0.4, and 0.5, to generate a smooth frontier line that accurately represented the nonlinear relationship between SDI and rabies ASDR. To ensure robustness, 1,000 bootstrap resampling iterations were performed, and the mean ASDR for each SDI value was calculated. The potential for improvement in reducing the rabies burden was assessed by measuring the absolute distance (effective difference) between ASDR and the estimated frontier line (15).

## 3 Results

### 3.1 Global

In 2021, the global ASIR of rabies was 0.129 per 100,000 population, the ASPR was 0.005 per 100,000 population, the ASMR was 0.128 per 100,000 population, and the ASDR was 7.496 per 100,000 population. From 1990 to 2021, all these rates showed significant declines, with the ASIR, ASPR, ASMR, and ASDR decreasing at annual average percentage changes (AAPC) of −0.010, −0.002%, −0.009%, and −0.552%, respectively (Tables 1–4). In absolute terms, there were an estimated 10,181 incident cases of rabies globally in 2021 and 10,084 deaths. From 1990 to 2021, global incident cases, prevalent cases, deaths, and DALYs attributable to rabies all showed a consistent decline (Supplementary Tables S1–S4).

**Table 1:** The ASIR of rabies in 1990 year and 2021 year, and change trend of ASIR were analyzed across GBD regions.

Location	ASIR (per 100, 000 population) (95% UI). 1990 year	ASIR (per 100, 000 population) (95% UI). 2021 year	Percentage change (95% UI). 1990–2021.	EAPC (95% CI). 1990–2021.	AAPC (95% CI). 1990–2021.
Global	0.422 (0.300, 0.549)	0.129 (0.076, 0.182)	−69.378 (−77.609, −61.026)	−4.060 (−4.324, −3.796)	−0.010 (−0.010, −0.009)
East Asia	0.099 (0.058, 0.137)	0.036 (0.020, 0.054)	−63.258 (−75.141, −48.083)	−1.141 (−3.229, 0.991)	−0.002 (−0.002, −0.002)
Southeast Asia	0.329 (0.230, 0.449)	0.098 (0.058, 0.147)	−70.099 (−79.361, −58.263)	−4.480 (−4.864, −4.094)	−0.007 (−0.008, −0.007)
Oceania	0.039 (0.012, 0.128)	0.028 (0.008, 0.084)	−28.108 (−62.581, 35.464)	−1.038 (−1.086, −0.989)	−0.001 (−0.002, −0.001)
Central Asia	0.019 (0.011, 0.035)	0.010 (0.006, 0.015)	−49.341 (−70.998, −14.528)	−2.843 (−3.524, −2.157)	−0.001 (−0.002, −0.001)
Central Europe	0.004 (0.003, 0.005)	0.002 (0.001, 0.003)	−98.089 (−98.544, −97.484)	−14.431 (−15.605, −13.241)	−0.001 (−0.002, −0.001)
Eastern Europe	0.005 (0.005, 0.006)	0.002 (0.002, 0.003)	−63.403 (−69.643, −54.428)	−2.007 (−3.010, −0.993)	−0.001 (−0.002, −0.001)
High-income Asia Pacific	0.002 (0.001, 0.003)	0.001 (0.000, 0.001)	−92.250 (−93.104, −91.255)	−7.040 (−8.487, −5.570)	−0.001 (−0.002, −0.001)
Australasia	0.002 (0.001, 0.003)	0.001 (0.001, 0.002)	37.565 (−10.339, 118.381)	4.668 (2.058, 7.344)	0.001 (−0.001, 0.001)
Western Europe	0.002 (0.001, 0.003)	0.001 (0.000, 0.001)	101.246 (79.344, 126.603)	2.340 (1.576, 3.110)	0.001 (0.000, 0.001)
Southern Latin America	0.002 (0.001, 0.003)	0.001 (0.000, 0.001)	−9.227 (−27.597, 12.578)	−1.433 (−3.140, 0.304)	0.001 (−0.001, 0.001)
High-income North America	0.002 (0.001, 0.003)	0.002 (0.001, 0.002)	181.438 (151.097, 212.731)	2.290 (1.780, 2.803)	0.001 (0.000, 0.001)
Caribbean	0.002 (0.001, 0.003)	0.001 (0.001, 0.002)	−24.885 (−54.189, 33.048)	−0.544 (−1.051, −0.033)	−0.001 (−0.001, −0.001)
Andean Latin America	0.047 (0.037, 0.056)	0.004 (0.002, 0.006)	−99.693 (−99.873, −99.400)	−20.490 (−22.798, −18.112)	−0.002 (−0.003, −0.001)
Central Latin America	0.041 (0.038, 0.044)	0.003 (0.002, 0.004)	−99.888 (−99.906, −99.868)	−19.140 (−19.920, −18.353)	−0.001 (−0.002, −0.001)
Tropical Latin America	0.050 (0.036, 0.067)	0.002 (0.001, 0.003)	−99.645 (−99.745, −99.495)	−15.906 (−17.445, −14.338)	−0.002 (−0.003, −0.001)
North Africa and Middle East	0.028 (0.015, 0.039)	0.003 (0.001, 0.004)	−90.048 (−93.342, −84.762)	−6.905 (−7.350, −6.458)	−0.001 (−0.002, −0.001)
South Asia	1.586 (1.159, 1.985)	0.305 (0.203, 0.417)	−80.783 (−84.806, −75.013)	−5.797 (−6.037, −5.556)	−0.042 (−0.044, −0.042)
Central Sub-Saharan Africa	0.034 (0.006, 0.089)	0.021 (0.004, 0.045)	−41.725 (−62.300, −6.931)	−1.640 (−1.851, −1.429)	−0.001 (−0.003, −0.001)
Eastern Sub-Saharan Africa	1.425 (0.642, 3.215)	0.496 (0.197, 1.132)	−65.165 (−79.084, −43.586)	−3.700 (−3.951, −3.449)	−0.030 (−0.032, −0.0329)
Southern Sub-Saharan Africa	0.055 (0.029, 0.086)	0.038 (0.022, 0.059)	−31.176 (−55.638, 10.714)	−1.070 (−1.600, −0.538)	−0.001 (−0.002, −0.001)
Western Sub-Saharan Africa	0.608 (0.255, 1.103)	0.333 (0.136, 0.526)	−45.297 (−66.628, −16.800)	−1.802 (−2.018, −1.584)	−0.009 (−0.011, −0.007)
High SDI	0.002 (0.001, 0.003)	0.001 (0.001, 0.002)	9.983 (−13.030, 37.640)	1.065 (0.238, 1.899)	0.001 (−0.001, 0.001)
High-middle SDI	0.035 (0.022, 0.047)	0.016 (0.009, 0.024)	−53.903 (−68.310, −37.283)	−1.125 (−2.772, 0.549)	−0.002 (−0.003, −0.001)
Middle SDI	0.231 (0.165, 0.288)	0.075 (0.044, 0.111)	−67.457 (−76.464, −55.623)	−3.306 (−3.929, −2.678)	−0.005 (−0.006, −0.004)
Low-middle SDI	1.101 (0.784, 1.379)	0.217 (0.139, 0.305)	−80.335 (−84.452, −73.661)	−5.736 (−5.968, −5.503)	−0.029 (−0.030, −0.028)
Low SDI	1.427 (0.900, 2.340)	0.415 (0.220, 0.711)	−70.916 (−79.890, −61.429)	−4.353 (−4.554, −4.153)	−0.034 (−0.036, −0.032)

The AAPC was used as the definitive criterion to determine the temporal trends of ASIR from 1990 to 2021. AAPC, average annual percent change; CI, confidence interval; EAPC, estimated annual percentage change; GBD, global burden of disease. UI, uncertainty interval; SDI, socio-demographic index.

**Table 2:** The ASPR of rabies in 1990 year and 2021 year, and change trend of ASPR were analyzed across GBD regions.

Location	ASPR (per 100,000 population) (95% UI). 1990 year	ASPR (per 100,000 population) (95% UI). 2021 year	Percentage change (95% UI). 1990–2021.	EAPC (95% CI). 1990–2021.	AAPC (95% CI). 1990–2021.
Global	0.016 (0.012, 0.021)	0.005 (0.003, 0.007)	−69.389 (−77.614, −61.041)	−4.066 (−4.304, −3.829)	−0.0020 (−0.003, −0.002)
East Asia	0.004 (0.002, 0.005)	0.001 (0.001, 0.002)	−63.262 (−75.137, −48.080)	−0.925 (−2.965, 1.158)	−0.001 (−0.003, −0.001)
Southeast Asia	0.013 (0.009, 0.017)	0.004 (0.002, 0.006)	−70.113 (−79.361, −58.281)	−4.464 (−4.842, −4.085)	−0.001 (−0.002, −0.001)
Oceania	0.002 (0.000, 0.005)	0.001 (0.000, 0.003)	−28.227 (−62.694, 35.102)	−1.017 (−1.063, −0.971)	−0.001 (−0.002, −0.001)
Central Asia	0.001 (0.001, 0.002)	0.001 (0.000, 0.001)	−49.358 (−71.011, −14.548)	−2.908 (−3.399, −2.415)	−0.001 (−0.002, −0.001)
Central Europe	0.001 (0.001, 0.002)	0.001 (0.000, 0.001)	−98.092 (−98.546, −97.488)	−14.340 (−15.587, −13.075)	−0.001 (−0.002, −0.001)
Eastern Europe	0.001 (0.001, 0.002)	0.001 (0.000, 0.001)	−63.408 (−69.646, −54.435)	−1.892 (−2.850, −0.925)	−0.001 (−0.002, −0.001)
High-income Asia Pacific	0.001 (0.001, 0.002)	0.001 (0.000, 0.001)	−92.253 (−93.107, −91.262)	−6.391 (−7.745, −5.016)	−0.001 (−0.002, −0.001)
Australasia	0.001 (0.001, 0.002)	0.001 (0.000, 0.001)	37.587 (−10.329, 118.421)	6.577 (4.200, 9.009)	0.001 (−0.001, 0.001)
Western Europe	0.001 (0.001, 0.002)	0.001 (0.000, 0.001)	101.246 (79.364, 126.603)	2.383 (1.820, 2.950)	0.001 (0.001, 0.002)
Southern Latin America	0.001 (0.001, 0.002)	0.001 (0.000, 0.001)	−9.175 (−27.556, 12.595)	−0.418 (−1.188, 0.358)	−0.001 (−0.001, −0.001)
High-income North America	0.001 (0.001, 0.002)	0.001 (0.000, 0.001)	181.384 (151.049, 212.671)	2.442 (2.025, 2.860)	0.001 (0.001, 0.002)
Caribbean	0.001 (0.001, 0.002)	0.001 (0.000, 0.001)	−24.887 (−54.183, 32.993)	−0.415 (−0.843, 0.015)	−0.001 (−0.002, −0.001)
Andean Latin America	0.002 (0.001, 0.002)	0.001 (0.000, 0.001)	−99.696 (−99.875, −99.405)	−20.077 (−22.302, −17.788)	−0.001 (−0.002, −0.001)
Central Latin America	0.002 (0.001, 0.002)	0.001 (0.000, 0.001)	−99.888 (−99.906, −99.868)	−19.389 (−19.928, −18.847)	−0.001 (−0.002, −0.001)
Tropical Latin America	0.002 (0.001, 0.003)	0.001 (0.000, 0.001)	−99.646 (−99.745, −99.496)	−17.335 (−18.490, −16.164)	−0.001 (−0.002, −0.001)
North Africa and Middle East	0.001 (0.001, 0.002)	0.001 (0.000, 0.001)	−90.070 (−93.356, −84.798)	−6.885 (−7.328, −6.439)	−0.001 (−0.002, −0.001)
South Asia	0.061 (0.045, 0.076)	0.012 (0.008, 0.016)	−80.788 (−84.810, −75.005)	−5.826 (−6.052, −5.599)	−0.002 (−0.003, −0.002)
Central Sub-Saharan Africa	0.001 (0.001, 0.003)	0.001 (0.000, 0.002)	−41.683 (−62.339, −6.781)	−1.630 (−1.820, −1.439)	−0.001 (−0.002, −0.001)
Eastern Sub-Saharan Africa	0.055 (0.025, 0.124)	0.019 (0.008, 0.043)	−65.168 (−79.087, −43.581)	−3.705 (−3.954, −3.456)	−0.001 (−0.002, −0.001)
Southern Sub-Saharan Africa	0.002 (0.001, 0.003)	0.001 (0.001, 0.002)	−31.149 (−55.639, 10.771)	−1.058 (−1.557, −0.558)	−0.001 (−0.002, −0.001)
Western Sub-Saharan Africa	0.023 (0.010, 0.042)	0.013 (0.005, 0.021)	−45.287 (−66.627, −16.806)	−1.805 (−1.998, −1.611)	−0.001 (−0.002, −0.001)
High SDI	0.001 (0.000, 0.001)	0.001 (0.000, 0.001)	9.972 (−13.052, 37.633)	1.269 (0.514, 2.030)	0.001 (0.000, 0.001)
High-middle SDI	0.001 (0.001, 0.002)	0.001 (0.000, 0.001)	−53.893 (−68.310, −37.293)	−0.975 (−2.577, 0.652)	−0.001 (−0.002, −0.001)
Middle SDI	0.009 (0.006, 0.011)	0.003 (0.002, 0.004)	−67.473 (−76.506, −55.645)	−3.265 (−3.854, −2.672)	−0.001 (−0.002, −0.001)
Low-middle SDI	0.042 (0.031, 0.053)	0.008 (0.005, 0.012)	−80.341 (−84.454, −73.667)	−5.760 (−5.981, −5.539)	−0.001 (−0.002, −0.001)
Low SDI	0.055 (0.035, 0.091)	0.016 (0.008, 0.027)	−70.922 (−79.896, −61.435)	−4.361 (−4.550, −4.171)	−0.001 (−0.002, −0.001)

The AAPC was used as the definitive criterion to determine the temporal trends of ASPR from 1990 to 2021. AAPC, average annual percent change; CI, confidence interval; EAPC, estimated annual percentage change. GBD, global burden of disease; UI, uncertainty interval; SDI, socio-demographic index.

**Table 3:** The ASMR of rabies in 1990 year and 2021 year, and change trend of ASMR were analyzed across GBD regions.

Location	ASMR (per 100,000 population) (95% UI). 1990 year	ASMR (per 100,000 population) (95% UI). 2021 year	Percentage change (95% UI). 1990–2021	EAPC (95% CI). 1990–2021	AAPC (95% CI). 1990–2021
Global	0.417 (0.297, 0.543)	0.128 (0.075, 0.181)	−69.351 (−77.619, −61.022)	−4.026 (−4.267, −3.785)	−0.009 (−0.010, −0.008)
East Asia	0.098 (0.057, 0.136)	0.036 (0.019, 0.054)	−63.238 (−75.179, −47.759)	−1.029 (−3.176, 1.166)	−0.002 (−0.002, −0.001)
Southeast Asia	0.326 (0.229, 0.442)	0.097 (0.058, 0.145)	−70.123 (−79.288, −58.224)	−4.488 (−4.871, −4.103)	−0.007 (−0.007, −0.006)
Oceania	0.039 (0.012, 0.126)	0.028 (0.008, 0.081)	−27.908 (−62.120, 34.784)	−1.040 (−1.095, −0.985)	−0.001 (−0.002, −0.001)
Central Asia	0.019 (0.011, 0.034)	0.010 (0.006, 0.015)	−49.215 (−70.525, −14.133)	−2.924 (−3.625, −2.219)	−0.001 (−0.002, −0.001)
Central Europe	0.004 (0.003, 0.005)	0.001 (0.000, 0.001)	−98.082 (−98.525, −97.486)	−13.994 (−15.528, −12.431)	−0.001 (−0.002, −0.001)
Eastern Europe	0.005 (0.004, 0.006)	0.002 (0.002, 0.002)	−63.452 (−69.529, −54.346)	−2.222 (−3.294, −1.138)	−0.001 (−0.002, −0.001)
High-income Asia Pacific	0.001 (0.001, 0.002)	0.001 (0.000, 0.001)	−92.250 (−93.103, −91.271)	−6.899 (−8.399, −5.374)	−0.001 (−0.002, −0.001)
Australasia	0.001 (0.001, 0.002)	0.001 (0.000, 0.001)	37.542 (−10.215, 120.050)	5.273 (2.243, 8.392)	0.001 (−0.003, 0.001)
Western Europe	0.001 (0.001, 0.002)	0.001 (0.000, 0.001)	100.776 (79.253, 124.349)	2.532 (1.632, 3.439)	0.001 (0.001, 0.002)
Southern Latin America	0.001 (0.001, 0.002)	0.001 (0.000, 0.001)	−9.134 (−27.367, 13.859)	−1.554 (−3.225, 0.145)	0.001 (−0.001, 0.001)
High-income North America	0.001 (0.001, 0.001)	0.002 (0.002, 0.002)	181.365 (151.339, 212.650)	2.821 (2.117, 3.530)	0.001 (0.001, 0.002)
Caribbean	0.002 (0.001, 0.003)	0.001 (0.001, 0.002)	−25.006 (−54.593, 35.004)	−0.554 (−0.995, −0.112)	−0.001 (−0.002, −0.001)
Andean Latin America	0.047 (0.037, 0.055)	0.001 (0.000, 0.001)	−99.692 (−99.875, −99.396)	−20.427 (−23.033, −17.734)	−0.002 (−0.002, −0.001)
Central Latin America	0.041 (0.037, 0.044)	0.001 (0.000, 0.001)	−99.887 (−99.905, −99.867)	−19.625 (−20.805, −18.428)	−0.001 (−0.001, −0.001)
Tropical Latin America	0.050 (0.036, 0.066)	0.001 (0.000, 0.001)	−99.643 (−99.744, −99.489)	−16.070 (−17.882, −14.219)	−0.002 (−0.002, −0.002)
North Africa and Middle East	0.027 (0.015, 0.040)	0.003 (0.001, 0.004)	−90.035 (−93.357, −84.943)	−6.919 (−7.366, −6.470)	−0.001 (−0.00’, −0.001)
South Asia	1.570 (1.148, 1.963)	0.302 (0.201, 0.414)	−80.772 (−84.778, −74.892)	−5.771 (−5.997, −5.545)	−0.041 (−0.042, −0.040)
Central Sub-Saharan Africa	0.033 (0.006, 0.085)	0.019 (0.004, 0.045)	−41.510 (−61.713, −7.083)	−1.584 (−1.794, −1.374)	−0.000 (−0.000, −0.000)
Eastern Sub-Saharan Africa	1.408 (0.637, 3.179)	0.492 (0.194, 1.130)	−65.042 (−79.170, −43.225)	−3.678 (−3.920, −3.435)	−0.030 (−0.030, −0.029)
Southern Sub-Saharan Africa	0.054 (0.029, 0.085)	0.037 (0.021, 0.057)	−31.222 (−56.090, 13.490)	−1.027 (−1.556, −0.495)	−0.001 (−0.002, −0.001)
Western Sub-Saharan Africa	0.602 (0.254, 1.107)	0.329 (0.135, 0.521)	−45.289 (−66.222, −17.452)	−1.792 (−2.004, −1.578)	−0.009 (−0.009, −0.008)
High SDI	0.001 (0.001, 0.003)	0.001 (0.001, 0.002)	10.202 (−12.135, 37.195)	1.304 (0.339, 2.278)	0.001 (−0.002, 0.002)
High-middle SDI	0.035 (0.021, 0.047)	0.016 (0.009, 0.024)	−53.876 (−68.342, −37.687)	−1.096 (−2.756, 0.592)	−0.001 (−0.002, −0.001)
Middle SDI	0.228 (0.163, 0.285)	0.074 (0.044, 0.110)	−67.458 (−76.373, −55.813)	−3.297 (−3.904, −2.686)	−0.005 (−0.006, −0.005)
Low-middle SDI	1.090 (0.777, 1.365)	0.214 (0.137, 0.302)	−80.330 (−84.467, −73.574)	−5.710 (−5.933, −5.487)	−0.028 (−0.030, −0.027)
Low SDI	1.412 (0.897, 2.305)	0.411 (0.217, 0.708)	−70.865 (−80.006, −61.361)	−4.312 (−4.499, −4.125)	−0.032 (−0.034, −0.031)

The AAPC was used as the definitive criterion to determine the temporal trends of ASMR from 1990 to 2021. AAPC, average annual percent change; CI, confidence interval; EAPC, estimated annual percentage change; GBD, global burden of disease; UI, uncertainty interval; SDI, socio-demographic index.

**Table 4:** The ASDR of rabies in 1990 year and 2021 year, and change trend of ASDR were analyzed across GBD regions.

Location	ASDR (per 100,000 population) (95% UI). 1990 year	ASDR (per 100,000 population) (95% UI). 2021 year	Percentage change (95% UI). 1990–2021	EAPC (95% CI). 1990–2021	AAPC (95% CI). 1990–2021.
Global	24.485 (17.485, 31.947)	7.496 (4.194, 10.994)	−69.386 (−79.345, −59.476)	−3.996 (−4.253, −3.737)	−0.552 (−0.577, −0.527)
East Asia	5.988 (3.508, 8.450)	1.810 (0.975, 2.761)	−69.773 (−79.867, −56.971)	−1.742 (−3.872, 0.435)	−0.139 (−0.160, −0.118)
Southeast Asia	18.644 (14.103, 25.395)	5.383 (3.121, 8.177)	−71.130 (−81.971, −57.693)	−4.587 (−4.969, −4.203)	−0.424 (−0.429, −0.419)
Oceania	0.651 (0.194, 2.033)	0.565 (0.150, 1.603)	−13.150 (−54.303, 66.833)	−0.313 (−0.402, −0.224)	−0.002 (−0.003, −0.002)
Central Asia	1.158 (0.620, 2.258)	0.534 (0.317, 0.891)	−53.906 (−75.454, −14.665)	−3.162 (−3.792, −2.528)	−0.022 (−0.027, −0.017)
Central Europe	0.124 (0.107, 0.146)	0.002 (0.001, 0.003)	−98.268 (−98.779, −97.626)	−13.768 (−15.474, −12.028)	−0.005 (−0.005, −0.004)
Eastern Europe	0.297 (0.275, 0.322)	0.081 (0.067, 0.101)	−72.562 (−77.214, −66.305)	−3.091 (−4.243, −1.926)	−0.008 (−0.010, −0.006)
High-income Asia Pacific	0.038 (0.034, 0.041)	0.003 (0.002, 0.003)	−92.661 (−93.683, −91.627)	−7.059 (−8.650, −5.439)	−0.001 (−0.001, −0.001)
Australasia	0.011 (0.009, 0.013)	0.019 (0.012, 0.030)	76.084 (9.643, 191.244)	7.098 (3.088, 11.263)	0.001 (−0.001, 0.001)
Western Europe	0.006 (0.005, 0.007)	0.012 (0.011, 0.014)	113.045 (84.250, 145.045)	2.930 (1.934, 3.935)	0.001 (0.001, 0.002)
Southern Latin America	0.002 (0.001, 0.003)	0.001 (0.001, 0.002)	−49.342 (−58.856, −36.811)	−3.218 (−4.846, −1.562)	−0.001 (−0.001, 0.001)
High-income North America	0.045 (0.042, 0.048)	0.119 (0.105, 0.133)	162.289 (131.455, 195.644)	2.647 (1.945, 3.354)	0.002 (0.001, 0.003)
Caribbean	0.043 (0.026, 0.064)	0.040 (0.014, 0.085)	−6.554 (−54.723, 93.345)	0.781 (−0.038, 1.607)	−0.001 (−0.001, 0.001)
Andean Latin America	2.560 (1.998, 3.128)	0.003 (0.001, 0.006)	−99.865 (−99.940, −99.750)	−23.146 (−26.060, −20.118)	−0.089 (−0.103, −0.076)
Central Latin America	2.323 (2.121, 2.532)	0.002 (0.001, 0.003)	−99.932 (−99.945, −99.916)	−20.606 (−21.799, −19.395)	−0.074 (−0.077, −0.071)
Tropical Latin America	3.105 (2.143, 4.259)	0.009 (0.008, 0.011)	−99.700 (−99.793, −99.550)	−16.446 (−18.277, −14.574)	−0.101 (−0.107, −0.094)
North Africa and Middle East	1.579 (0.821, 2.378)	0.137 (0.063, 0.215)	−91.356 (−94.705, −85.377)	−7.190 (−7.686, −6.692)	−0.047 (−0.048, −0.046)
South Asia	79.862 (59.158, 99.394)	14.188 (9.128, 20.181)	−82.235 (−86.388, −76.528)	−5.987 (−6.236, −5.737)	−2.112 (−2.162, −2.063)
Central Sub-Saharan Africa	1.731 (0.344, 5.187)	0.925 (0.185, 2.509)	−46.553 (−70.215, −7.235)	−1.828 (−2.056, −1.599)	−0.026 (−0.028, −0.025)
Eastern Sub-Saharan Africa	76.613 (35.408, 167.615)	26.671 (10.273, 59.735)	−65.188 (−81.046, −41.760)	−3.658 (−3.885, −3.430)	−1.616 (−1.633, −1.600)
Southern Sub-Saharan Africa	3.232 (1.804, 4.998)	2.243 (1.341, 3.663)	−30.606 (−56.531, 16.608)	−1.046 (−1.542, −0.546)	−0.034 (−0.038, −0.031)
Western Sub-Saharan Africa	35.795 (16.323, 62.729)	19.752 (7.864, 31.374)	−44.818 (−69.532, −11.432)	−1.737 (−1.957, −1.517)	−0.513 (−0.529, −0.497)
High SDI	0.069 (0.048, 0.091)	0.080 (0.063, 0.101)	15.711 (−9.638, 46.786)	1.402 (0.540, 2.271)	0.001 (0.000, 0.002)
High-middle SDI	2.114 (1.294, 2.895)	0.799 (0.448, 1.189)	−62.183 (−75.291, −48.623)	−1.810 (−3.438, −0.154)	−0.044 (−0.051, −0.037)
Middle SDI	12.329 (9.241, 15.062)	3.686 (2.082, 5.482)	−70.100 (−79.590, −58.139)	−3.504 (−4.107, −2.898)	−0.280 (−0.287, −0.272)
Low-middle SDI	55.940 (40.433, 71.229)	10.441 (6.422, 14.769)	−81.335 (−85.962, −74.456)	−5.835 (−6.068, −5.601)	−1.459 (−1.491, −1.428)
Low SDI	76.193 (48.327, 122.197)	21.906 (10.818, 37.738)	−71.249 (−82.286, −60.235)	−4.332 (−4.519, −4.144)	−1.764 (−1.792, −1.735)

The AAPC was used as the definitive criterion to determine the temporal trends of ASDR from 1990 to 2021. AAPC, average annual percent change; ASDR, age-standardized DALY rate; CI, confidence interval; EAPC, estimated annual percentage change; GBD, global burden of disease; UI, uncertainty interval; SDI, socio-demographic index.

### 3.2 Five SDI

In 2021, the burden of rabies, as measured by ASIR, ASPR, ASMR, and ASDR, was highest in low-SDI regions and lowest in high-SDI regions. Between 1990 and 2021, these indicators consistently declined across low, low-middle, middle, and high-middle SDI regions, while showing an increase in high-SDI regions (Tables 1–4). Similarly, the absolute burden of incident cases, prevalent cases, deaths, and DALYs was greatest in low-SDI regions and lowest in high-SDI regions, with a decrease observed in the former and an increase in the latter over the study period (Supplementary Tables S1–S4).

### 3.3 Geographical regions

In 2021, the highest ASIR of rabies was observed in Eastern Sub-Saharan Africa, while the greatest absolute number of incident cases was reported in South Asia. Between 1990 and 2021, the ASIR increased in Western Europe and High-income North America, but declined in 17 regions, with the most significant decrease in Eastern Sub-Saharan Africa (Table 1). In terms of absolute incident cases, the number increased in eight regions, particularly in Western Sub-Saharan Africa, but decreased in 13 regions, with the largest reduction observed in South Asia (Supplementary Table 1).

In 2021, the highest ASPR of rabies was recorded in Eastern Sub-Saharan Africa, while the greatest absolute number of prevalent cases occurred in South Asia. Between 1990 and 2021, the ASPR of rabies increased in Western Europe and High-income North America, but declined in 19 regions, with the steepest reduction observed in Eastern Sub-Saharan Africa (Table 2). Additionally, the absolute number of prevalent cases rose in eight regions, notably in Western Sub-Saharan Africa, while it decreased in 12 regions, with the most significant decline in South Asia (Supplementary Table 2).

In 2021, the highest ASMR of rabies was observed in Eastern Sub-Saharan Africa, while the greatest absolute number of deaths occurred in South Asia. From 1990 to 2021, the ASMR of rabies increased in Western Europe and High-income North America, but declined in 17 regions, with the most significant reduction seen in South Asia (Table 3). In addition, the absolute number of deaths rose in seven regions, particularly in Western Sub-Saharan Africa, while it decreased in 12 regions, with the largest decline in South Asia. (Supplementary Table 3).

In 2021, the highest ASDR of rabies was recorded in Eastern Sub-Saharan Africa, while the greatest absolute number of DALYs occurred in South Asia. Between 1990 and 2021, the ASDR of rabies increased in Western Europe and High-income North America, but declined in 17 regions, with the steepest reduction observed in South Asia (Table 4). Additionally, the absolute number of DALYs rose in four regions, particularly in High-income North America, but decreased in 15 regions, with the most significant decline in South Asia (Supplementary Table 4).

### 3.4 Countries and territories

In 2021, Nepal recorded the highest ASIR of rabies, while the largest absolute number of incident cases was observed in India. Between 1990 and 2021, the ASIR increased in 13 countries, with the greatest rise in Somalia, but declined in 165 locations, with the steepest reduction in Nepal. Additionally, the absolute number of incident cases rose in 80 countries, with the largest increase in Nigeria, while it decreased in 102 locations, with the most significant decline in India (Supplementary Tables 5–6).

In 2021, Nepal reported the highest ASPR of rabies, while the largest absolute number of prevalent cases was observed in India. Between 1990 and 2021, the ASPR increased in 31 countries, with the most pronounced rise in Jamaica, but declined in 160 locations, with the steepest reduction in Mexico. Additionally, the absolute number of prevalent cases increased in 90 countries, with the sharpest rise in Jamaica, while it decreased in 91 locations, with the largest decline in Mexico (Supplementary Tables 7, 8).

In 2021, Nepal reported the highest ASMR of rabies, while the largest absolute number of deaths occurred in India. Between 1990 and 2021, the ASMR increased in 20 countries, with the steepest rise in Canada, but declined in 153 locations, with the largest reduction in Guyana. Additionally, the absolute number of deaths rose in 74 countries, with the most pronounced increase in Turkmenistan, while it decreased in 76 locations, with the steepest decline in Saint Lucia (Supplementary Tables 9, 10).

In 2021, Nepal reported the highest ASDR of rabies, while the largest absolute number of DALYs occurred in India. Between 1990 and 2021, the ASDR increased in 15 countries, with the steepest rise in Turkmenistan, but declined in 150 locations, with the largest reduction in Jamaica. Additionally, the absolute number of DALYs rose in 52 countries, with the sharpest increase in Tunisia, while it decreased in 91 locations, with the steepest decline in Saint Vincent and the Grenadines (Supplementary Tables 11, 12).

### 3.5 Age-gender group analysis

**Figure 1:**
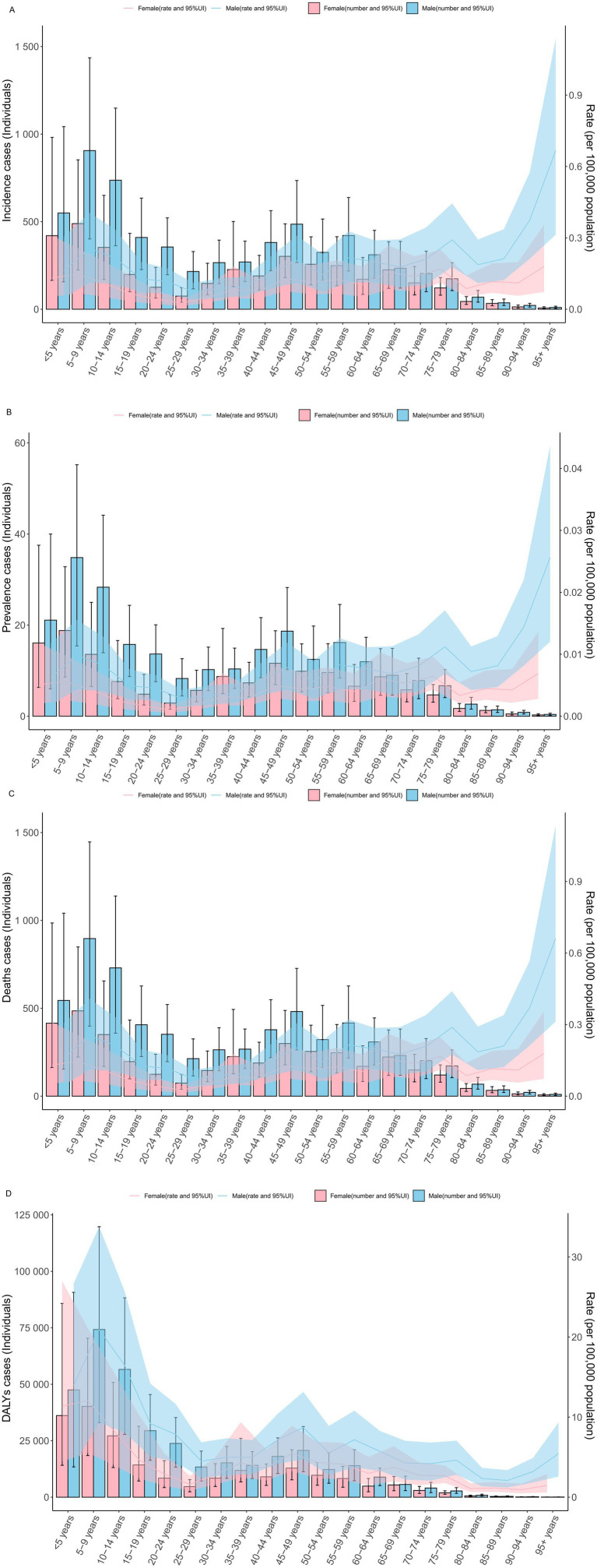
The specific rate of rabies showed notable differences across age and gender distributions in 2021 year **(A)** incidence rate. **(B)** prevalence rate. **(C)** mortality rate. **(D)** DALYs rate. DALYs, disability-adjusted life years; UI, uncertainty interval.

In 2021, there were no significant differences in the incidence rate, prevalence rate, mortality rate, and DALYs rate of rabies between males and females across all age groups globally. Similarly, the absolute numbers of cases—incidence, prevalence, mortality, and DALYs—showed no significant gender-based differences across the various age groups (Figures 1A–D).

Notably, the specific DALYs rate and DALY cases were highest in the age groups under 5 years, 5–9 years, and 10–14 years, while these values were comparatively lower in all age groups over 60 years (Figures 1A–D).

### 3.6 Association between burden indicators and SDI

In 2021, the ASIR, ASPR, ASMR, and ASDR of rabies, together with the absolute numbers of incident cases, prevalent cases, deaths cases, and DALYs cases, were negatively correlated with the SDI across 204 countries and territories (Table 5).

**Table 5:** The association between the burden indicators (ASR and cases) of rabies and SDI.

Locations	Index	*r*	*P*
204 countries and territories (2021 year)	ASIR	−0.695	<0.001
	ASPR	−0.695	<0.001
	ASMR	−0.695	<0.001
	ASDR	−0.694	<0.001
204 countries and territories (2021 year)	Incidence cases	−0.583	<0.001
	Prevalence cases	−0.583	<0.001
	Death cases	−0.583	<0.001
	DALYs cases	−0.613	<0.001

ASDR: age standardized disability-adjusted life year rate; ASIR, age standardized incidence rate; ASMR, age standardized mortality rate; ASPR, age standardized prevalence rate; ASR, age standardized rate; DALYs, disability-adjusted life years; SDI, sociodemographic index.

### 3.7 Projections

By 2035, globally, the ASIR of rabies is projected to decrease to 0.091 per 100,000 population (95% *CI*: 0.061, 0.112 per 100,000 population), while the ASMR is expected to decline to 0.081 per 100,000 population (95% *CI*: 0.061, 0.102 per 100,000 population). The AAPC values further highlight the gradual and steady decline in the global burden of rabies (Table 6).

**Table 6:** The prediction of global burden of rabies for 2022–2035 based on the BAPC model.

Index	ASIR	ASPR	ASMR	ASDR
Value (per 100,000 population) (95% *CI*). 2035 year	0.091 (0.061, 0.112)	0.003 (0.002, 0.004)	0.081 (0.061, 0.102)	5.114 (3.692, 6.536)
AAPC (95% *CI*). 2022–2035 year	−0.003(−0.004,−0.003)	−0.003 (−0.004, −0.002)	-0.003(−0.005, −0.003)	−0.189 (−0.190, −0.188)

ASDR, age standardized disability-adjusted life year rate; ASIR, age standardized incidence rate; ASPR, age standardized prevalence rate; AAPC, average annual percent change; ASMR, age standardized mortality rate; BAPC, Bayesian age-period-cohort; CI, confidence interval; DALYs, disability-adjusted life years.

### 3.8 Decomposition analysis

**Figure 2:**
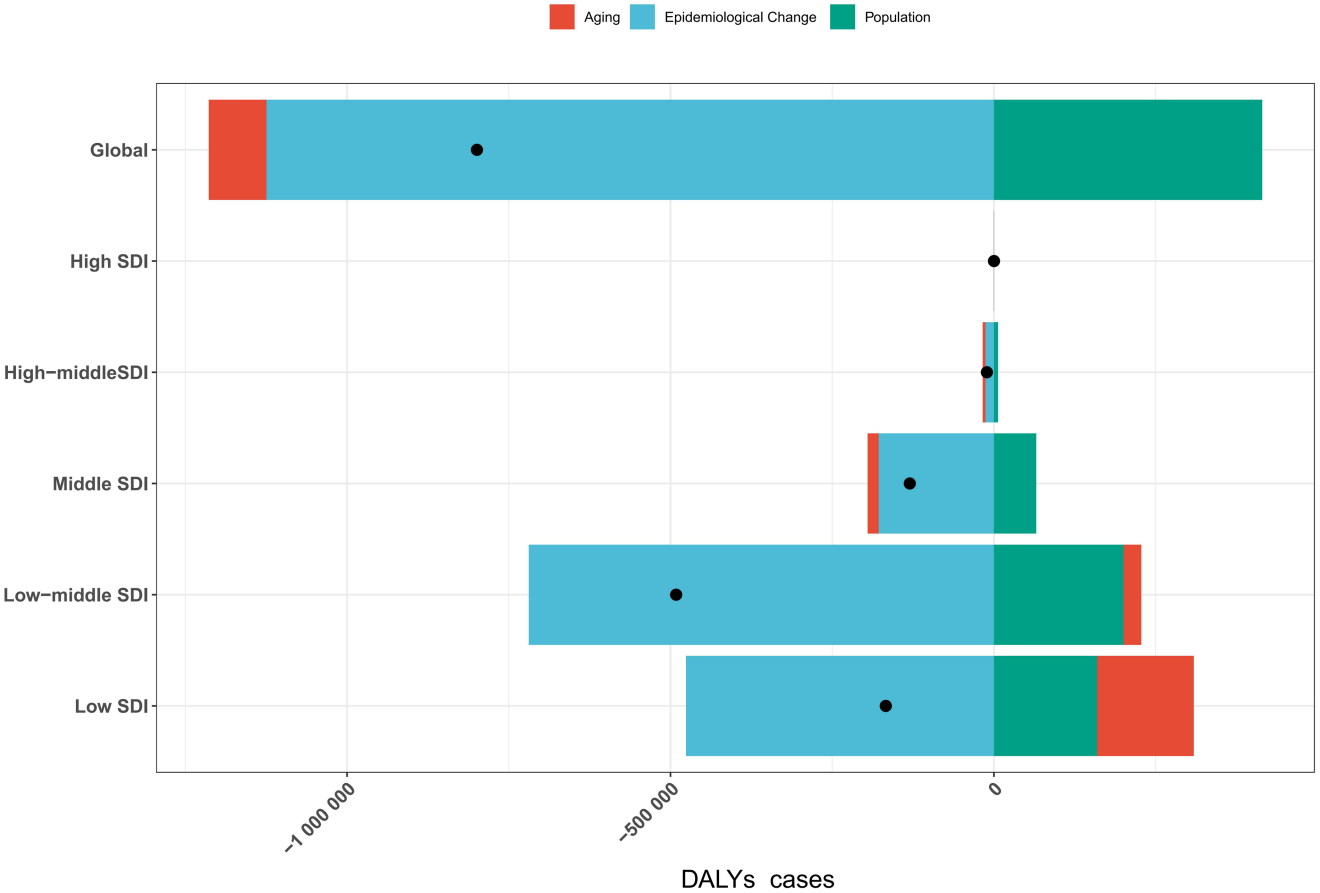
Impact of aging, population growth, and epidemiological changes on rabies DALYs cases globally and across five SDI regions in 2021. Black dots represent the total change contributed by all three factors. A positive value for each component indicates a corresponding increase in DALYs cases, while a negative value reflects a decrease in DALYs cases. DALYs, disability-adjusted life years; SDI, sociodemographic index.

Globally, aging, population growth, and epidemiological changes contributed to changes in DALYs cases by 11.1615, −51.885%, and −1,124,615.561%, respectively. However, there were regional variations: DALYs cases increased in high-SDI regions, while it decreased in high-middle, middle, low-middle, and low-SDI regions (Figure 2; Supplementary Table 13).

### 3.9 Health inequality analysis

**Figure 3:**
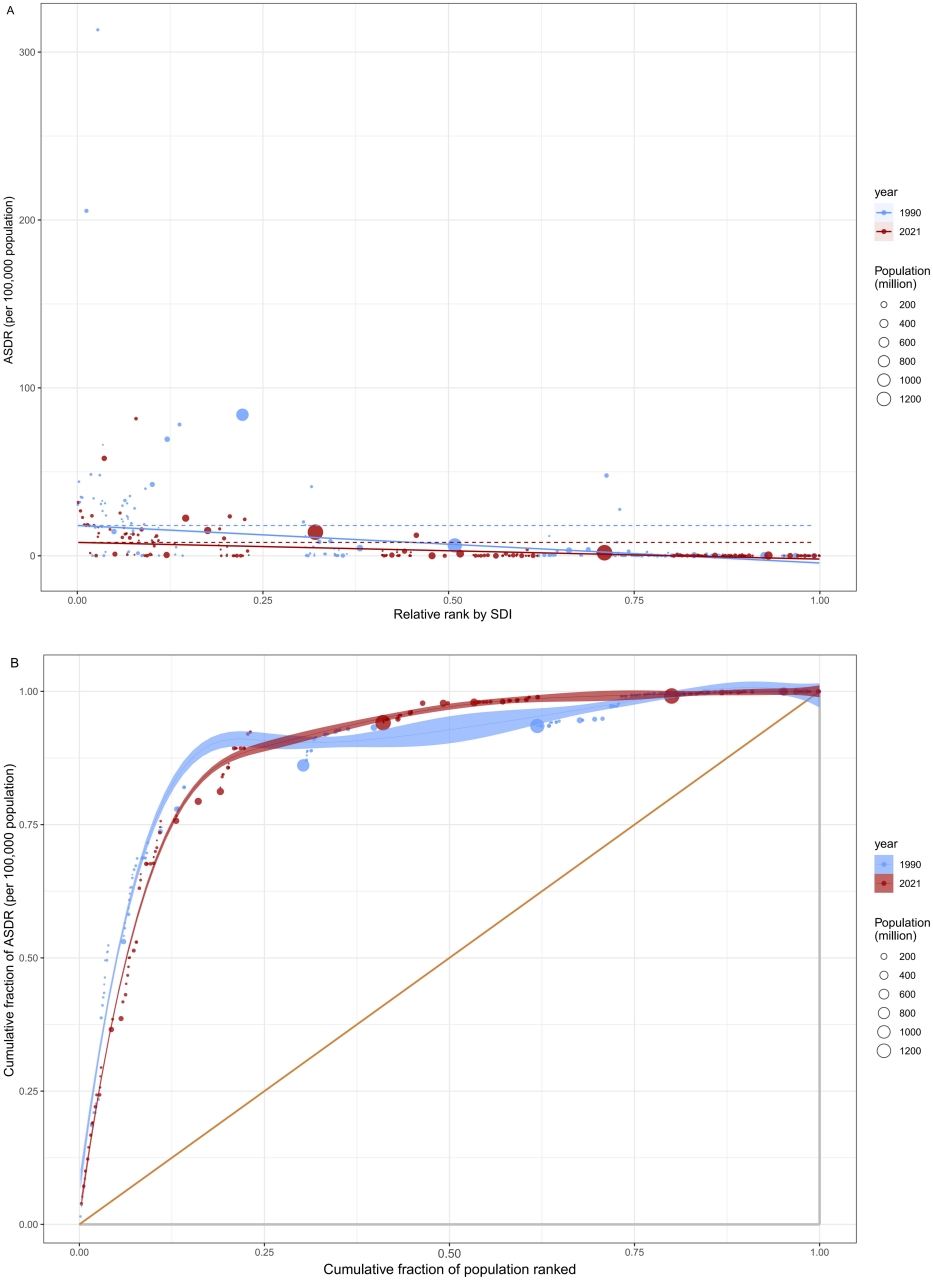
Health inequality regression analysis for rabies ASDR across 204 countries and territories. **(A)** illustrated the inequality slope index, depicting the relationship between rabies ASDR and SDI. Each point represents an individual country and territory, with the size of the point weighted by population. **(B)** presented the concentration index, which quantifies relative inequality by calculating the area under the Lorenz curve. This index aligns the distribution of rabies ASDR with the population distribution categorized by SDI. Data from 1990 is shown in blue, while data from 2021 is represented in red. ASDR, age standardized disability-adjusted life year rate; SDI, socio-demographic index.

Significant absolute and relative health inequalities in rabies ASDR were observed in association with SDI (Figures 3A,B). The SSI reveals a decrease in the gap in ASDR between countries and territories with the highest and lowest SDI, from −22.331 (95% *CI*: −24.722, −19.952) in 1990 to −9.931 (95% *CI*: −11.352, −8.521) in 2021. In contrast, the CCI showed a slight change, moving from −0.712 (95% *CI*: −0.783, −0.616) in 1990 to −0.721 (95% *CI*: −0.770, −0.664) in 2021. These results suggest a reduction in absolute health inequalities in rabies ASDR from 1990 to 2021, while relative inequalities remained relatively stable.

### 3.10 Frontier analysis

**Figure 4:**
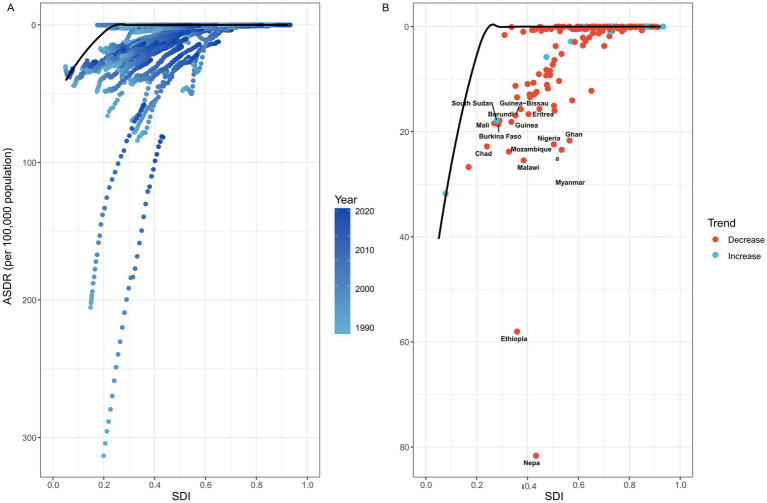
Frontier analysis was conducted to explore the association between ASDR and SDI for rabies in 204 countries and territories. **(A)** This panel showed the temporal trend of rabies ASDR changes, with a color gradient ranging from light blue (representing 1990) to dark blue (representing 2021). This gradient highlights how the ASDR has evolved globally over time. **(B)** Each point represented a specific country or territory in 2021, with the black frontier line indicating the optimal ASDR relative to the country’s SDI. Light blue points represent low-SDI countries that are close to the frontier line, signifying that these countries are approaching the optimal ASDR for their development level. Red points represent high-SDI countries exhibiting the largest gaps from the frontier, indicating significant room for improvement in rabies control despite relatively high development levels. In addition, blue points indicate a decrease in ASDR, while red points indicate an increase. ASDR, age standardized disability-adjusted life year rate; SDI, Socio-demographic index.

A frontier analysis was performed to assess the potential for improvement in rabies ASDR across 204 countries and territories, relative to their respective levels of development as measured by the SDI (Figure 4; Supplementary Table 14). The 15 countries and territories exhibiting the largest disparities in potential improvement (effective difference range: 16.640–81.661) include Nepal, Ethiopia, Malawi, Mozambique, Myanmar, Chad, Nigeria, Ghana, Burkina Faso, Mali, Guinea, Burundi, South Sudan, Guinea-Bissau, and Eritrea. In contrast, low-SDI countries such as Haiti, Yemen, Afghanistan, Niger, and the Solomon Islands are close to the frontier line, indicating that although their development levels are low, their rabies ASDR is relatively close to the optimal level. In addition, high-SDI countries, including the United Arab Emirates, Puerto Rico, the United States, the United Kingdom, and Poland, despite having relatively ample resources, still exhibit significant potential for improvement in rabies ASDR (effective difference range: 302.981–371.140).

## 4 Discussion

This study systematically analyzed global and regional trends in the burden of rabies from 1990 to 2021 and projected future trajectories. Despite marked global declines in ASIR, ASPR, ASMR, and ASDR, the burden of rabies remains unequally distributed. Low SDI regions, particularly Sub-Saharan Africa and South Asia, continue to experience a disproportionately high burden. By contrast, in some high-income regions such as North America and Western Europe, overall levels remain low but incidence has shown an upward trend.

The global burden of rabies is characterized by striking inequalities. Rabies poses a particularly heavy burden in Nepal, South Asia, and many parts of Africa, especially in countries with low socioeconomic development (26). In these regions, where health systems are often under-resourced and structural inequities persist, weak dog population management, widespread stray dogs, and insufficient vaccination coverage have allowed sustained transmission of the virus between animals and humans. Limited health-care resources and the high cost or poor availability of PEP further increase population vulnerability (9, 27, 28). By contrast, in high-income countries, overall incidence is low, yet imported cases and wildlife reservoirs such as bats and raccoons continue to pose ongoing threats to public health (9, 27, 28).

Reducing this unequal burden requires coordinated, multilevel action. In low-income settings, priority should be given to expanding human and canine vaccine coverage, strengthening dog management and stray control, and ensuring affordable and accessible PEP (29, 30); public education campaigns are also essential to improve awareness of wound care and prevention after exposure. In high-income regions, enhanced cross-border surveillance, long-term wildlife reservoir control, and improved case-tracking and data-sharing systems are needed (31). At the global level, while financial, technical, and vaccine supply support from high-income countries and international organizations is crucial, it is essential to emphasize the foundational role of political will, national investment, and sustainable financing. A collaborative framework built on strong national leadership and sustainable financing strategies is critical for achieving lasting progress in the elimination of rabies (31–33).

In the past, successful and sustained interventions, such as dog vaccination campaigns in Latin America and the expansion of access to PEP in Asia, have significantly contributed to the reduction of the rabies burden in these regions. Building on this, to achieve the elimination of rabies in the future, priority should be given to strengthening canine vaccination coverage and standardizing and expanding human post-exposure prophylaxis networks. Additionally, enhancing public education and advocacy, raising awareness of prevention, and reducing exposure events will provide critical support for achieving the goal of rabies elimination. The implementation of these comprehensive measures will significantly reduce the global rabies burden and accelerate progress toward rabies elimination (34, 35).

This study has several limitations. First, the analysis is based on data from the GBD 2021 study, which faces notable challenges in quality and coverage, particularly in low-income and developing countries, where incomplete or inaccurate records may compromise reliability (13, 17). Second, the GBD estimates are model-derived rather than directly observed, it may introduce risks of overestimation of rabies burden (13, 36, 37). Third, the predictive models applied, including the BAPC model, rely heavily on the quality of available data and historical trends. Yet, key determinants such as human–dog interactions, canine infection rates, dog vaccination rate, and fluctuations in dog populations—critical drivers of rabies transmission—are not fully incorporated into the GBD modeling framework. Consequently, the projections may not adequately capture the complexity of future rabies dynamics.

## 5 Conclusion

Rabies remains disproportionately concentrated in low-SDI countries and among children, reflecting inequalities in human and dog vaccination, dog virus surveillance, post-exposure prophylaxis, and the distribution of health resources. Achieving the global elimination target for rabies will require not only increased financial investment, technical assistance, and vaccine support from high-income countries to strengthen North–South collaboration, but also the integration of dog population management, expanded vaccine coverage, cross-sectoral cooperation, and robust public health surveillance systems. Only through such comprehensive and equity-focused strategies can sustainable rabies elimination be accelerated.

## Data Availability

The original contributions presented in the study are included in the article/Supplementary material, further inquiries can be directed to the corresponding author/s.
