# Supplementary material for: From data to action: addressing geographic inequalities in rabies burden through targeted policies
Source: Front Public Health. 2025 Oct 21;13:1683863. doi: 10.3389/fpubh.2025.1683863 (PMC12584861; doi:10.3389/fpubh.2025.1683863)
Supplement: Supplementary file 1 [file Data_Sheet_1.docx]

**Supplementary materials**

**Original Research**

From data to action: addressing geographic inequalities in rabies burden through targeted policies

He Lu^1^, En-Li Tan^3*^

^1^ School of Global Health, Chinese Center for Tropical Diseases Research, Shanghai Jiao Tong University School of Medicine, Shanghai 20025, China; One Health Center, Shanghai Jiao Tong University-The University of Edinburgh, Shanghai, 20025, China.

^2^ The First Hospital of Lanzhou University, Lanzhou 730000, China.

* CORRESPONDENCE.

En-Li Tan.

[13659421557@163.com](mailto:18817338863@126.com);).

**Items**

Supplementary Table 1: Incidence cases of rabies in 2021, and change trend of incidence cases were analyzed across GBD regions.

Supplementary Table 2: Prevalence cases of rabies in 2021, and change trend of incidence cases were analyzed across GBD regions.

Supplementary Table 3: The mortality cases of rabies in 2021, and change trend of incidence cases were analyzed across GBD regions.

Supplementary Table 4: The DALY cases of rabies in 2021, and change trend of DALY cases were analyzed across GBD regions.

Supplementary Table 5: Trends in the ASIR of rabies in 204 countries and territories worldwide from 1990 to 2021.

Supplementary Table 6: Trends in the number of rabies incident cases in 204 countries and territories worldwide from 1990 to 2021.

Supplementary Table 7: Trends in the ASPR of rabies in 204 countries and territories worldwide from 1990 to 2021.

Supplementary Table 8: Trends in the number of rabies prevalent cases in 204 countries and territories worldwide from 1990 to 2021.

Supplementary Table 9: Trends in the ASMR of rabies in 204 countries and territories worldwide from 1990 to 2021.

Supplementary Table 10: Trends in the number of rabies mortality cases in 204 countries and territories Worldwide from 1990 to 2021.

Supplementary Table 11: Trends in the ASDR of rabies in 204 countries and territories worldwide from 1990 to 2021.

Supplementary Table 12: Trends in the number of rabies DALYs in 204 countries and territories Worldwide from 1990 to 2021.

Supplementary Figure 1: Prediction of global disease burden of rabies from 2022–2035 years based on the BAPC model .

Supplementary Table 13: A decomposition analysis was conducted on the number of DALYs attributable to rabies, examining trends globally and within five SDI regions from 1990 to 2021.

Supplementary Table 14: Frontier analysis exploring ASDR of rabies across 204 countries and territories.

Supplementary Table 1: Incidence cases of rabies in 2021, and change trend of incidence cases were analyzed across GBD regions.

| Location | Rabies. Incidence cases (persons) . (95% UI).  1990 year | Rabies. Incidence cases (persons) . (95% UI).  2021 year | Rabies. Percentage change (95% UI). 1990－2021. | Rabies. EAPC (95% CI). 1990－2021. | Rabies. AAPC (95% CI). 1990－2021. |
| --- | --- | --- | --- | --- | --- |
| Global | 22035(15732,28729) | 10181(6080,14293) | -53.798(-66.518,-40.377) | -4.035(-4.283,-3.786) | -390.610(-412.675,-368.545) |
| East Asia | 1103(647,1526) | 627(335,950) | -43.208(-61.524,-18.546) | -0.349(-2.333,1.674) | -14.327(-19.184,-9.471) |
| Southeast Asia | 1469(1095,1968) | 663(393,972) | -54.853(-70.689,-34.237) | -4.453(-4.819,-4.084) | -26.093(-26.709,-25.477) |
| Oceania | 1(0,2) | 2(0,4) | 126.574(16.979,333.250) | 0.091(-0.049,0.232) | 0.029(0.028,0.029) |
| Central Asia | 13(7,25) | 9(6,14) | -31.589(-63.178,24.175) | -3.131(-3.817,-2.441) | -0.188(-0.250,-0.125) |
| Central Europe | 4(4,5) | 0(0,0) | -96.397(-97.281,-95.219) | -12.038(-13.239,-10.820) | -0.149(-0.168,-0.130) |
| Eastern Europe | 12(11,13) | 5(4,7) | -56.091(-64.247,-43.916) | -1.299(-2.230,-0.359) | -0.249(-0.295,-0.202) |
| High-income Asia Pacific | 1(1,1) | 0(0,0) | -62.658(-71.349,-55.681) | -2.543(-3.947,-1.117) | -0.024(-0.028,-0.020) |
| Australasia | 0(0,0) | 0(0,0) | 113.877(45.840,212.633) | 4.349(2.127,6.620) | 0.001(0.000,0.002) |
| Western Europe | 1(0,1) | 2(2,2) | 280.424(224.769,348.655) | 3.647(2.903,4.397) | 0.051(0.041,0.062) |
| Southern Latin America | 0(0,0) | 0(0,0) | 71.383(35.414,114.907) | -0.457(-2.179,1.295) | 0.000(0.000,0.001) |
| High-income North America | 2(2,2) | 7(6,7) | 300.706(255.449,343.679) | 2.534(2.026,3.044) | 0.154(0.133,0.176) |
| Caribbean | 0(0,1) | 1(0,1) | 49.927(-5.011,164.642) | 0.736(0.216,1.258) | 0.005(0.003,0.008) |
| Andean Latin America | 17(13,20) | 0(0,0) | -99.504(-99.798,-99.012) | -20.815(-23.295,-18.255) | -0.626(-0.712,-0.541) |
| Central Latin America | 65(60,71) | 0(0,0) | -99.829(-99.857,-99.797) | -19.115(-19.904,-18.319) | -2.090(-2.194,-1.987) |
| Tropical Latin America | 78(55,105) | 0(0,0) | -99.482(-99.636,-99.262) | -15.993(-17.513,-14.445) | -2.607(-2.732,-2.482) |
| North Africa and Middle East | 94(48,140) | 15(7,23) | -83.726(-89.564,-72.671) | -7.230(-7.721,-6.736) | -2.588(-2.647,-2.529) |
| South Asia | 15417(11334,19205) | 5189(3452,7083) | -66.344(-73.806,-55.458) | -5.725(-5.973,-5.476) | -339.643(-350.364,-328.921) |
| Central Sub-Saharan Africa | 17(3,51) | 22(4,60) | 30.426(-32.949,133.378) | -1.928(-2.157,-1.698) | 0.151(0.137,0.164) |
| Eastern Sub-Saharan Africa | 2478(1162,5344) | 1916(740,4224) | -22.702(-59.198,30.103) | -3.662(-3.876,-3.448) | -18.855(-19.836,-17.874) |
| Southern Sub-Saharan Africa | 29(16,44) | 29(17,47) | 1.974(-34.669,65.887) | -1.239(-1.703,-0.773) | -0.005(-0.044,0.035) |
| Western Sub-Saharan Africa | 1235(567,2162) | 1693(674,2705) | 37.155(-26.100,121.000) | -1.756(-1.977,-1.534) | 14.411(13.441,15.382) |
| High SDI | 11(7,14) | 17(12,23) | 59.969(25.341,106.955) | 1.617(0.687,2.554) | 0.201(0.157,0.244) |
| High-middle SDI | 354(218,479) | 244(136,365) | -30.956(-52.576,-5.438) | -0.472(-2.060,1.142) | -3.390(-4.723,-2.056) |
| Middle SDI | 3592(2636,4418) | 1904(1134,2795) | -46.997(-61.613,-26.942) | -2.926(-3.497,-2.352) | -55.849(-59.070,-52.627) |
| Low-middle SDI | 11414(8211,14550) | 3817(2453,5399) | -66.560(-74.068,-54.687) | -5.660(-5.899,-5.421) | -250.985(-258.265,-243.706) |
| Low SDI | 6660(4227,10637) | 4196(2074,7177) | -37.004(-61.406,-12.851) | -4.395(-4.587,-4.203) | -81.964(-85.291,-78.637) |

Abbreviations: AAPC, average annual percent change; CI, confidence interval; EAPC, estimated annual percentage change; GBD, Global burden of disease; UI, Uncertainty interval; SDI, Socio-demographic index.

Supplementary Table 2: Prevalence cases of rabies in 2021, and change trend of incidence cases were analyzed across GBD regions.

| Location | Rabies. Prevalence cases (persons) . (95% UI).  1990 year. | Rabies. Prevalence cases (persons) . (95% UI).  2021 year. | Rabies. Percentage change (95% UI). 1990－2021. | Rabies. EAPC (95% CI). 1990－2021. | Rabies. AAPC (95% CI). 1990－2021. |
| --- | --- | --- | --- | --- | --- |
| Global | 847(605,1104) | 391(234,549) | -53.810(-66.524,-40.395) | -4.039(-4.263,-3.816) | -14.926(-15.545,-14.308) |
| East Asia | 42(25,59) | 24(13,37) | -43.208(-61.526,-18.548) | -0.131(-2.059,1.836) | -0.423(-0.654,-0.192) |
| Southeast Asia | 56(42,75) | 25(15,37) | -54.867(-70.698,-34.298) | -4.436(-4.798,-4.072) | -0.995(-1.005,-0.984) |
| Oceania | 0(0,0) | 0(0,0) | 126.242(16.834,332.574) | 0.111(-0.013,0.234) | 0.001(0.001,0.001) |
| Central Asia | 1(0,1) | 0(0,1) | -31.618(-63.204,24.136) | -3.200(-3.713,-2.683) | -0.007(-0.009,-0.005) |
| Central Europe | 0(0,0) | 0(0,0) | -96.401(-97.284,-95.227) | -11.833(-13.091,-10.556) | -0.005(-0.005,-0.005) |
| Eastern Europe | 0(0,1) | 0(0,0) | -56.092(-64.248,-43.918) | -1.198(-2.081,-0.307) | -0.008(-0.009,-0.008) |
| High-income Asia Pacific | 0(0,0) | 0(0,0) | -62.663(-71.354,-55.684) | -1.866(-3.163,-0.552) | -0.001(-0.001,-0.001) |
| Australasia | 0(0,0) | 0(0,0) | 113.904(45.860,212.668) | 6.109(4.120,8.136) | 0.000(0.000,0.000) |
| Western Europe | 0(0,0) | 0(0,0) | 280.540(224.918,348.782) | 3.750(3.147,4.356) | 0.002(0.002,0.002) |
| Southern Latin America | 0(0,0) | 0(0,0) | 71.474(35.560,115.073) | 0.564(-0.241,1.377) | 0.000(0.000,0.000) |
| High-income North America | 0(0,0) | 0(0,0) | 300.642(255.397,343.609) | 2.684(2.258,3.111) | 0.006(0.006,0.006) |
| Caribbean | 0(0,0) | 0(0,0) | 49.944(-4.961,164.600) | 0.861(0.413,1.311) | 0.000(0.000,0.000) |
| Andean Latin America | 1(1,1) | 0(0,0) | -99.509(-99.800,-99.020) | -20.415(-22.812,-17.944) | -0.021(-0.022,-0.021) |
| Central Latin America | 3(2,3) | 0(0,0) | -99.829(-99.857,-99.797) | -19.377(-19.922,-18.829) | -0.080(-0.081,-0.080) |
| Tropical Latin America | 3(2,4) | 0(0,0) | -99.483(-99.636,-99.263) | -17.407(-18.539,-16.260) | -0.096(-0.100,-0.092) |
| North Africa and Middle East | 4(2,5) | 1(0,1) | -83.759(-89.581,-72.729) | -7.207(-7.694,-6.717) | -0.098(-0.100,-0.097) |
| South Asia | 593(436,738) | 199(133,272) | -66.349(-73.823,-55.470) | -5.755(-5.992,-5.518) | -12.897(-13.162,-12.632) |
| Central Sub-Saharan Africa | 1(0,2) | 1(0,2) | 30.460(-32.993,133.617) | -1.910(-2.122,-1.697) | 0.006(0.006,0.006) |
| Eastern Sub-Saharan Africa | 95(45,205) | 74(28,162) | -22.719(-59.223,30.115) | -3.668(-3.880,-3.457) | -0.728(-0.763,-0.692) |
| Southern Sub-Saharan Africa | 1(1,2) | 1(1,2) | 2.004(-34.685,66.015) | -1.226(-1.660,-0.790) | 0.000(-0.001,0.002) |
| Western Sub-Saharan Africa | 47(22,83) | 65(26,104) | 37.194(-26.082,121.204) | -1.760(-1.956,-1.563) | 0.570(0.557,0.583) |
| High SDI | 0(0,1) | 1(0,1) | 59.938(25.374,106.780) | 1.833(0.971,2.702) | 0.009(0.007,0.010) |
| High-middle SDI | 14(8,18) | 9(5,14) | -30.931(-52.568,-5.409) | -0.322(-1.857,1.237) | -0.099(-0.170,-0.029) |
| Middle SDI | 138(101,170) | 73(44,107) | -47.018(-61.781,-26.961) | -2.879(-3.417,-2.338) | -2.060(-2.130,-1.989) |
| Low-middle SDI | 439(316,559) | 147(94,208) | -66.567(-74.069,-54.704) | -5.685(-5.913,-5.457) | -9.546(-9.704,-9.388) |
| Low SDI | 256(163,409) | 161(80,276) | -37.015(-61.413,-12.857) | -4.408(-4.590,-4.225) | -3.158(-3.235,-3.081) |

Abbreviations: AAPC, average annual percent change; CI, confidence interval; EAPC, estimated annual percentage change; GBD, Global burden of disease; UI, Uncertainty interval; SDI, Socio-demographic index.

Supplementary Table 3: The mortality cases of rabies in 2021, and change trend of incidence cases were analyzed across GBD regions

| Location | Rabies. Death cases (persons)  (95% UI).  1990 year | Rabies. Death cases (persons)  (95% UI).  2021 year | Rabies. Percentage change (95% UI). 1990－2021. | Rabies. EAPC (95% CI). 1990－2021. | Rabies AAPC (95% CI). 1990－2021. |
| --- | --- | --- | --- | --- | --- |
| Global | 21806(15577,28435) | 10084(6016,14172) | -53.755(-66.541,-40.542) | -3.999(-4.224,-3.773) | -381.648(-404.540,-358.757) |
| East Asia | 1091(642,1511) | 620(329,926) | -43.198(-61.540,-18.992) | -0.227(-2.281,1.871) | -16.672(-21.393,-11.951) |
| Southeast Asia | 1455(1088,1946) | 657(388,962) | -54.883(-70.675,-34.574) | -4.464(-4.829,-4.098) | -25.547(-26.002,-25.091) |
| Oceania | 1(0,2) | 2(0,4) | 127.163(17.132,330.380) | 0.082(-0.071,0.235) | 0.028(0.027,0.029) |
| Central Asia | 13(7,25) | 9(6,14) | -31.400(-62.688,23.067) | -3.224(-3.936,-2.506) | -0.112(-0.194,-0.030) |
| Central Europe | 4(4,5) | 0(0,0) | -96.380(-97.263,-95.208) | -11.497(-13.092,-9.872) | -0.148(-0.169,-0.128) |
| Eastern Europe | 12(11,13) | 5(4,7) | -56.181(-64.177,-44.012) | -1.489(-2.536,-0.431) | -0.231(-0.342,-0.121) |
| High-income Asia Pacific | 1(1,1) | 0(0,0) | -62.643(-70.948,-55.667) | -2.377(-3.872,-0.858) | -0.025(-0.032,-0.018) |
| Australasia | 0(0,0) | 0(0,0) | 113.817(46.488,211.669) | 5.000(2.516,7.544) | 0.001(-0.000,0.002) |
| Western Europe | 1(0,1) | 2(2,2) | 278.782(227.459,340.699) | 3.928(3.055,4.808) | 0.053(0.045,0.062) |
| Southern Latin America | 0(0,0) | 0(0,0) | 71.548(35.995,115.990) | -0.572(-2.258,1.143) | 0.000(0.000,0.001) |
| High-income North America | 2(2,2) | 7(6,7) | 300.567(255.539,343.386) | 3.077(2.373,3.787) | 0.158(0.116,0.199) |
| Caribbean | 0(0,1) | 1(0,1) | 49.677(-5.171,160.188) | 0.766(0.308,1.227) | 0.006(0.002,0.010) |
| Andean Latin America | 17(13,20) | 0(0,0) | -99.503(-99.798,-99.031) | -20.722(-23.557,-17.782) | -0.545(-0.722,-0.367) |
| Central Latin America | 65(59,70) | 0(0,0) | -99.828(-99.857,-99.795) | -19.600(-20.806,-18.376) | -2.047(-2.146,-1.947) |
| Tropical Latin America | 77(54,104) | 0(0,0) | -99.478(-99.634,-99.253) | -16.160(-17.974,-14.305) | -2.599(-2.760,-2.439) |
| North Africa and Middle East | 93(48,139) | 15(7,23) | -83.698(-89.797,-73.103) | -7.245(-7.739,-6.749) | -2.546(-2.608,-2.483) |
| South Asia | 15259(11273,18974) | 5139(3417,7014) | -66.319(-73.772,-55.610) | -5.690(-5.920,-5.460) | -324.440(-337.098,-311.782) |
| Central Sub-Saharan Africa | 16(3,49) | 21(4,59) | 30.975(-31.291,132.163) | -1.875(-2.100,-1.650) | 0.148(0.134,0.163) |
| Eastern Sub-Saharan Africa | 2450(1146,5314) | 1900(732,4205) | -22.435(-59.112,29.809) | -3.639(-3.846,-3.432) | -18.422(-19.466,-17.378) |
| Southern Sub-Saharan Africa | 29(16,45) | 29(17,46) | 1.896(-34.957,70.093) | -1.195(-1.661,-0.727) | -0.005(-0.043,0.034) |
| Western Sub-Saharan Africa | 1221(567,2100) | 1676(666,2664) | 37.285(-26.590,123.034) | -1.745(-1.961,-1.529) | 14.464(13.402,15.526) |
| High SDI | 11(7,14) | 17(12,22) | 60.200(24.786,104.792) | 1.852(0.792,2.923) | 0.189(0.147,0.231) |
| High-middle SDI | 350(216,473) | 242(134,361) | -30.926(-52.801,-5.820) | -0.434(-2.041,1.200) | -4.027(-5.251,-2.803) |
| Middle SDI | 3556(2614,4368) | 1885(1124,2763) | -46.999(-61.724,-27.116) | -2.910(-3.467,-2.351) | -55.067(-57.570,-52.563) |
| Low-middle SDI | 11298(8121,14398) | 3780(2427,5358) | -66.546(-74.042,-54.866) | -5.628(-5.853,-5.403) | -241.831(-250.007,-233.656) |
| Low SDI | 6588(4206,10508) | 4159(2057,7142) | -36.873(-61.531,-12.740) | -4.345(-4.520,-4.171) | -78.982(-82.065,-75.899) |

Abbreviations: AAPC, average annual percent change; CI, confidence interval; EAPC, estimated annual percentage change; GBD, Global burden of disease; UI, Uncertainty interval; SDI, Socio-demographic index.

Supplementary Table 4: The DALY cases of rabies in 2021, and change trend of DALY cases were analyzed across GBD regions.

| Location | Rabies. DALYs cases. (persons)  (95% UI).  1990 year. | Rabies. DALYs cases. (persons)  (95% UI).  2021 year. | Rabies. Percentage change (95% UI). 1990－2021. | Rabies. EAPC (95% CI). 1990－2021. | Rabies. AAPC (95% CI). 1990－2021. |
| --- | --- | --- | --- | --- | --- |
| Global | 1368780(978542,1786737) | 569550(323362,828522) | -58.390(-71.936,-44.807) | -4.305(-4.546,-4.063) | -25979.438(-27353.585,-24605.291) |
| East Asia | 69479(40697,98021) | 26591(14377,39906) | -61.728(-74.516,-45.821) | -1.647(-3.635,0.383) | -1467.831(-1711.608,-1224.055) |
| Southeast Asia | 95997(74309,130900) | 36336(21329,54988) | -62.149(-77.427,-42.933) | -4.995(-5.365,-4.624) | -1915.647(-1940.824,-1890.470) |
| Oceania | 17(5,50) | 49(11,139) | 179.928(55.082,400.918) | 1.108(0.891,1.326) | 1.043(1.021,1.065) |
| Central Asia | 869(447,1746) | 508(304,844) | -41.499(-69.896,12.624) | -3.682(-4.341,-3.018) | -11.908(-16.014,-7.802) |
| Central Europe | 150(130,179) | 3(2,4) | -98.217(-98.669,-97.616) | -13.077(-14.879,-11.236) | -4.974(-5.755,-4.194) |
| Eastern Europe | 628(582,681) | 179(143,225) | -71.512(-76.674,-64.013) | -2.754(-3.903,-1.591) | -16.767(-21.500,-12.035) |
| High-income Asia Pacific | 45(39,49) | 5(4,6) | -88.601(-90.533,-86.917) | -6.150(-7.638,-4.638) | -1.442(-1.628,-1.256) |
| Australasia | 2(2,2) | 4(3,6) | 107.557(29.004,232.090) | 6.103(2.452,9.884) | 0.063(-0.025,0.150) |
| Western Europe | 18(15,20) | 47(40,53) | 159.092(127.897,191.193) | 2.994(2.059,3.939) | 0.776(0.201,1.351) |
| Southern Latin America | 1(1,1) | 0(0,0) | -30.502(-42.773,-14.528) | -3.281(-4.897,-1.636) | -0.009(-0.035,0.016) |
| High-income North America | 107(100,113) | 312(279,350) | 192.462(160.401,226.222) | 2.127(1.430,2.830) | 8.148(5.868,10.428) |
| Caribbean | 14(9,20) | 18(7,38) | 30.528(-38.379,167.925) | 1.048(0.175,1.929) | 0.056(-0.141,0.254) |
| Andean Latin America | 1060(810,1316) | 2(1,4) | -99.801(-99.912,-99.622) | -23.669(-26.685,-20.528) | -36.344(-42.582,-30.106) |
| Central Latin America | 4345(3927,4781) | 4(3,5) | -99.909(-99.927,-99.888) | -20.961(-22.159,-19.744) | -137.761(-144.085,-131.437) |
| Tropical Latin America | 5295(3611,7362) | 20(17,24) | -99.625(-99.745,-99.445) | -16.968(-18.785,-15.109) | -180.141(-190.776,-169.505) |
| North Africa and Middle East | 6375(3139,9863) | 847(393,1346) | -86.708(-92.009,-76.556) | -7.746(-8.266,-7.223) | -181.624(-185.464,-177.785) |
| South Asia | 928413(683557,1177412) | 257449(166295,365123) | -72.270(-79.120,-63.078) | -6.253(-6.516,-5.990) | -21644.034(-22578.427,-20709.642) |
| Central Sub-Saharan Africa | 1119(258,3559) | 1354(272,4062) | 20.954(-43.802,125.279) | -2.081(-2.335,-1.826) | 6.383(5.409,7.357) |
| Eastern Sub-Saharan Africa | 165400(77796,348595) | 124148(46425,272056) | -24.940(-63.779,29.470) | -3.693(-3.907,-3.479) | -1377.781(-1428.898,-1326.664) |
| Southern Sub-Saharan Africa | 1975(1141,3025) | 1847(1101,3053) | -6.469(-41.626,62.694) | -1.514(-1.960,-1.067) | -5.914(-8.902,-2.925) |
| Western Sub-Saharan Africa | 87471(42005,146987) | 119825(47579,192227) | 36.989(-30.632,129.379) | -1.720(-1.951,-1.488) | 1028.003(948.198,1107.808) |
| High SDI | 529(358,702) | 700(523,914) | 32.117(1.825,68.846) | 1.280(0.290,2.279) | 5.211(3.216,7.206) |
| High-middle SDI | 21212(13116,28992) | 10360(5839,15255) | -51.161(-67.443,-33.277) | -1.664(-3.200,-0.104) | -377.340(-444.070,-310.611) |
| Middle SDI | 219108(165879,266668) | 89044(51203,131920) | -59.361(-72.400,-43.137) | -3.726(-4.271,-3.178) | -4287.508(-4423.150,-4151.866) |
| Low-middle SDI | 691064(495900,899196) | 199815(122514,284866) | -71.086(-78.624,-59.972) | -6.039(-6.285,-5.792) | -15791.548(-16404.464,-15178.633) |
| Low SDI | 436599(280848,690532) | 269468(125691,465241) | -38.280(-65.308,-9.494) | -4.373(-4.559,-4.186) | -5406.149(-5653.629,-5158.669) |

Abbreviations: AAPC, average annual percent change; CI, confidence interval; EAPC, estimated annual percentage change; GBD, Global burden of disease; UI, Uncertainty interval; SDI, Socio-demographic index.

Supplementary Table 5: Trends in the ASIR of rabies in 204 countries and territories worldwide from 1990 to 2021.

| Location | ASIR  (per 100,000 population)  (95% UI).  1990 year | ASIR  (per 100,000 population)  (95% UI).  2021 year | Percentage change (95% UI). 1990－2021. | EAPC (95% CI). 1990－2021. | AAPC (95% CI). 1990－2021. |
| --- | --- | --- | --- | --- | --- |
| China | 0.101(0.059,0.140) | 0.037(0.020,0.055) | -63.953(-75.650,-48.649) | -1.171(-3.289,0.993) | -0.002(-0.002,-0.002) |
| Democratic People's Republic of Korea | 0.053(0.023,0.097) | 0.057(0.022,0.099) | 7.460(-37.641,75.733) | 0.254(-0.183,0.693) | 0.000(0.000,0.000) |
| Taiwan (Province of China) | 0.000(0.000,0.000) | 0.000(0.000,0.000) | -69.008(-77.333,-58.511) | -1.563(-3.588,0.504) | -0.000(-0.000,-0.000) |
| Cambodia | 0.552(0.187,0.990) | 0.174(0.058,0.336) | -68.444(-81.775,-47.668) | -4.651(-5.127,-4.172) | -0.012(-0.013,-0.012) |
| Indonesia | 0.069(0.045,0.103) | 0.021(0.012,0.036) | -69.285(-83.765,-45.701) | -3.739(-4.000,-3.478) | -0.002(-0.002,-0.002) |
| Lao People's Democratic Republic | 0.604(0.228,1.084) | 0.186(0.074,0.318) | -69.128(-82.433,-45.031) | -4.188(-4.648,-3.727) | -0.013(-0.014,-0.013) |
| Malaysia | 0.004(0.002,0.007) | 0.001(0.001,0.002) | -64.110(-80.318,-23.506) | -3.108(-3.403,-2.811) | -0.000(-0.000,-0.000) |
| Maldives | 0.007(0.003,0.015) | 0.001(0.001,0.003) | -79.433(-89.782,-53.105) | -4.786(-5.127,-4.444) | -0.000(-0.000,-0.000) |
| Myanmar | 1.362(0.789,2.216) | 0.430(0.196,0.773) | -68.425(-84.537,-38.320) | -3.931(-4.147,-3.714) | -0.030(-0.031,-0.030) |
| Philippines | 0.856(0.643,1.056) | 0.240(0.144,0.338) | -72.003(-79.594,-64.190) | -4.974(-5.591,-4.352) | -0.020(-0.021,-0.019) |
| Sri Lanka | 0.604(0.315,0.928) | 0.089(0.035,0.161) | -85.286(-92.774,-71.924) | -8.113(-8.970,-7.247) | -0.016(-0.017,-0.015) |
| Thailand | 0.042(0.018,0.078) | 0.006(0.003,0.010) | -85.183(-91.847,-71.044) | -8.011(-11.329,-4.569) | -0.001(-0.001,-0.001) |
| Timor-Leste | 0.442(0.113,0.954) | 0.184(0.060,0.342) | -58.418(-75.513,-21.088) | -3.205(-3.430,-2.980) | -0.008(-0.008,-0.008) |
| Viet Nam | 0.168(0.077,0.279) | 0.058(0.029,0.095) | -65.717(-80.075,-35.734) | -3.473(-3.565,-3.381) | -0.004(-0.004,-0.004) |
| Fiji | 0.012(0.004,0.029) | 0.012(0.005,0.025) | 0.550(-61.242,178.893) | 0.754(0.176,1.335) | 0.000(-0.000,0.000) |
| Kiribati | 0.087(0.044,0.164) | 0.068(0.030,0.135) | -22.102(-64.093,67.066) | -0.810(-0.888,-0.731) | -0.001(-0.001,-0.001) |
| Marshall Islands | 0.036(0.011,0.113) | 0.021(0.006,0.052) | -42.492(-74.704,43.840) | -1.974(-2.211,-1.736) | -0.001(-0.001,-0.001) |
| Micronesia (Federated States of) | 0.050(0.018,0.120) | 0.027(0.011,0.063) | -46.338(-74.812,17.551) | -1.855(-1.920,-1.791) | -0.001(-0.001,-0.001) |
| Papua New Guinea | 0.046(0.007,0.181) | 0.033(0.006,0.109) | -27.157(-71.046,94.614) | -0.932(-1.009,-0.854) | -0.000(-0.000,-0.000) |
| Samoa | 0.042(0.014,0.109) | 0.028(0.010,0.064) | -33.024(-68.063,48.261) | -1.083(-1.171,-0.995) | -0.000(-0.000,-0.000) |
| Solomon Islands | 0.045(0.007,0.150) | 0.033(0.008,0.105) | -25.700(-65.661,142.699) | -1.127(-1.256,-0.997) | -0.000(-0.000,-0.000) |
| Tonga | 0.031(0.010,0.090) | 0.019(0.007,0.047) | -38.208(-73.049,55.329) | -1.312(-1.504,-1.119) | -0.000(-0.000,-0.000) |
| Vanuatu | 0.041(0.009,0.123) | 0.027(0.006,0.083) | -33.956(-70.488,59.979) | -1.259(-1.358,-1.159) | -0.000(-0.000,-0.000) |
| Armenia | 0.000(0.000,0.001) | 0.000(0.000,0.000) | -85.659(-91.842,-75.130) | -6.500(-9.685,-3.203) | -0.000(-0.000,0.000) |
| Azerbaijan | 0.038(0.012,0.091) | 0.014(0.004,0.034) | -63.091(-89.433,25.443) | -4.643(-5.560,-3.716) | -0.001(-0.001,-0.001) |
| Georgia | 0.021(0.011,0.036) | 0.017(0.009,0.026) | -20.464(-64.255,74.182) | -1.161(-3.814,1.565) | -0.001(-0.001,0.000) |
| Kazakhstan | 0.011(0.007,0.016) | 0.017(0.009,0.031) | 52.867(-24.417,217.140) | 0.743(-1.283,2.811) | 0.000(-0.000,0.000) |
| Kyrgyzstan | 0.033(0.018,0.057) | 0.009(0.005,0.016) | -71.547(-89.055,-30.610) | -4.445(-6.359,-2.492) | -0.001(-0.001,-0.000) |
| Mongolia | 0.152(0.015,0.515) | 0.047(0.004,0.153) | -69.327(-94.125,66.457) | -4.361(-4.680,-4.040) | -0.003(-0.004,-0.003) |
| Tajikistan | 0.001(0.000,0.003) | 0.000(0.000,0.001) | -58.934(-86.037,37.215) | -3.465(-3.849,-3.079) | -0.000(-0.000,-0.000) |
| Turkmenistan | 0.003(0.002,0.005) | 0.001(0.000,0.002) | -73.941(-89.093,-34.566) | -4.238(-10.914,2.937) | -0.000(-0.000,0.000) |
| Uzbekistan | 0.011(0.006,0.022) | 0.005(0.003,0.008) | -55.400(-76.538,-12.048) | -3.613(-4.017,-3.206) | -0.000(-0.000,-0.000) |
| Albania | 0.001(0.000,0.001) | 0.000(0.000,0.000) | -90.098(-96.951,-67.845) | -10.262(-11.671,-8.829) | -0.000(-0.000,-0.000) |
| Bosnia and Herzegovina | 0.000(0.000,0.000) | 0.000(0.000,0.000) | -88.353(-96.832,-61.671) | -6.974(-8.114,-5.820) | -0.000(-0.000,-0.000) |
| Bulgaria | 0.000(0.000,0.001) | 0.001(0.000,0.001) | 50.305(-6.556,137.563) | 1.291(0.067,2.529) | 0.000(0.000,0.000) |
| Croatia | 0.001(0.001,0.001) | 0.000(0.000,0.000) | -98.665(-99.171,-97.787) | -17.436(-19.580,-15.234) | -0.000(-0.000,-0.000) |
| Czechia | 0.000(0.000,0.000) | 0.000(0.000,0.000) | -77.140(-84.571,-65.696) | -5.590(-8.622,-2.458) | -0.000(-0.000,0.000) |
| Hungary | 0.000(0.000,0.000) | 0.000(0.000,0.000) | 6.402(-23.322,50.160) | -6.945(-10.102,-3.678) | 0.000(-0.000,0.000) |
| North Macedonia | 0.000(0.000,0.000) | 0.000(0.000,0.000) | -55.100(-81.436,19.649) | -4.032(-5.313,-2.734) | -0.000(-0.000,-0.000) |
| Montenegro | 0.001(0.000,0.002) | 0.001(0.000,0.001) | -40.625(-74.874,32.281) | -2.111(-2.418,-1.803) | -0.000(-0.000,-0.000) |
| Poland | 0.004(0.003,0.005) | 0.000(0.000,0.000) | -99.701(-99.764,-99.565) | -20.901(-22.867,-18.886) | -0.000(-0.000,-0.000) |
| Romania | 0.011(0.010,0.014) | 0.000(0.000,0.000) | -99.763(-99.833,-99.671) | -19.188(-20.867,-17.474) | -0.000(-0.000,-0.000) |
| Serbia | 0.001(0.000,0.001) | 0.000(0.000,0.000) | -85.105(-92.814,-66.122) | -8.024(-9.159,-6.875) | -0.000(-0.000,-0.000) |
| Slovakia | 0.000(0.000,0.001) | 0.000(0.000,0.000) | -62.033(-81.577,-21.607) | -3.077(-3.198,-2.955) | -0.000(-0.000,-0.000) |
| Slovenia | 0.000(0.000,0.000) | 0.000(0.000,0.000) | -41.157(-56.074,-16.501) | -6.325(-9.049,-3.519) | 0.000(-0.000,0.000) |
| Belarus | 0.002(0.002,0.003) | 0.010(0.008,0.014) | 343.738(162.729,581.606) | 4.682(2.799,6.600) | 0.000(0.000,0.000) |
| Estonia | 0.000(0.000,0.001) | 0.001(0.000,0.001) | 11.677(-32.536,78.746) | 0.840(-2.320,4.103) | -0.000(-0.000,0.000) |
| Latvia | 0.019(0.014,0.025) | 0.002(0.002,0.003) | -87.010(-91.562,-80.252) | -7.346(-9.096,-5.563) | -0.001(-0.001,-0.000) |
| Lithuania | 0.002(0.001,0.004) | 0.000(0.000,0.000) | -99.253(-99.618,-98.592) | -15.394(-19.837,-10.706) | -0.000(-0.000,-0.000) |
| Republic of Moldova | 0.000(0.000,0.000) | 0.000(0.000,0.000) | -69.083(-81.755,-47.087) | -3.353(-4.370,-2.326) | -0.000(-0.000,-0.000) |
| Russian Federation | 0.006(0.006,0.007) | 0.001(0.001,0.001) | -79.702(-81.770,-77.393) | -3.855(-5.447,-2.237) | -0.000(-0.000,-0.000) |
| Ukraine | 0.003(0.002,0.004) | 0.002(0.001,0.004) | -9.597(-54.574,87.933) | 1.164(0.123,2.216) | -0.000(-0.000,-0.000) |
| Brunei Darussalam | 0.000(0.000,0.000) | 0.000(0.000,0.000) | -65.025(-80.843,-32.539) | -2.512(-3.410,-1.605) | -0.000(-0.000,-0.000) |
| Japan | 0.001(0.001,0.001) | 0.000(0.000,0.000) | -92.228(-93.117,-91.290) | -6.865(-8.433,-5.269) | -0.000(-0.000,-0.000) |
| Republic of Korea | 0.000(0.000,0.001) | 0.000(0.000,0.000) | -97.437(-98.871,-85.414) | -12.882(-14.791,-10.930) | -0.000(-0.000,-0.000) |
| Singapore | 0.000(0.000,0.000) | 0.000(0.000,0.000) | -60.580(-72.884,-45.759) | -5.449(-7.013,-3.860) | -0.000(-0.000,0.000) |
| Australia | 0.000(0.000,0.000) | 0.000(0.000,0.000) | 166.007(54.917,341.314) | 8.421(4.986,11.968) | 0.000(-0.000,0.000) |
| New Zealand | 0.001(0.000,0.001) | 0.000(0.000,0.000) | -75.940(-83.352,-66.720) | -3.335(-4.634,-2.019) | -0.000(-0.000,-0.000) |
| Andorra | 0.000(0.000,0.000) | 0.000(0.000,0.000) | -53.567(-76.587,-10.140) | -2.390(-2.573,-2.206) | -0.000(-0.000,-0.000) |
| Austria | 0.000(0.000,0.000) | 0.000(0.000,0.000) | -37.148(-55.537,-7.710) | -2.600(-4.917,-0.227) | -0.000(-0.000,0.000) |
| Belgium | 0.000(0.000,0.000) | 0.000(0.000,0.000) | -70.898(-79.577,-59.311) | 1.591(-1.175,4.436) | -0.000(-0.000,-0.000) |
| Cyprus | 0.004(0.001,0.011) | 0.001(0.000,0.001) | -86.073(-94.352,-56.267) | -5.497(-6.599,-4.381) | -0.000(-0.000,-0.000) |
| Denmark | 0.001(0.001,0.001) | 0.000(0.000,0.000) | -89.143(-92.088,-85.518) | -6.488(-8.262,-4.680) | -0.000(-0.000,-0.000) |
| Finland | 0.001(0.000,0.001) | 0.000(0.000,0.001) | -49.948(-73.780,-8.157) | -2.551(-2.877,-2.223) | -0.000(-0.000,-0.000) |
| France | 0.000(0.000,0.000) | 0.000(0.000,0.000) | -24.056(-48.622,12.858) | -3.600(-5.609,-1.548) | -0.000(-0.000,0.000) |
| Germany | 0.000(0.000,0.000) | 0.000(0.000,0.000) | 375.587(188.082,628.627) | 5.498(3.964,7.055) | 0.000(0.000,0.000) |
| Greece | 0.000(0.000,0.000) | 0.002(0.001,0.003) | 602.978(363.094,1024.547) | 13.275(9.021,17.696) | 0.000(0.000,0.000) |
| Iceland | 0.002(0.002,0.003) | 0.001(0.001,0.002) | -35.291(-55.038,-8.591) | -3.676(-7.452,0.253) | -0.000(-0.000,0.000) |
| Ireland | 0.000(0.000,0.000) | 0.000(0.000,0.000) | -37.563(-53.647,-12.590) | 0.587(-1.162,2.366) | -0.000(-0.000,0.000) |
| Israel | 0.000(0.000,0.000) | 0.000(0.000,0.000) | -91.182(-94.211,-86.557) | -11.424(-13.506,-9.292) | -0.000(-0.000,-0.000) |
| Italy | 0.000(0.000,0.000) | 0.000(0.000,0.000) | -93.102(-94.128,-91.699) | -8.438(-9.398,-7.469) | -0.000(-0.000,-0.000) |
| Luxembourg | 0.001(0.001,0.001) | 0.000(0.000,0.000) | -56.805(-68.514,-42.288) | -2.555(-6.479,1.534) | -0.000(-0.000,-0.000) |
| Malta | 0.002(0.001,0.002) | 0.000(0.000,0.000) | -97.969(-98.486,-97.249) | -10.649(-13.135,-8.092) | -0.000(-0.000,-0.000) |
| Netherlands | 0.000(0.000,0.000) | 0.000(0.000,0.000) | 218.310(127.042,342.255) | 4.817(1.800,7.924) | 0.000(-0.000,0.000) |
| Norway | 0.001(0.001,0.001) | 0.006(0.005,0.007) | 513.551(391.752,635.558) | 4.148(2.479,5.844) | 0.000(0.000,0.000) |
| Portugal | 0.000(0.000,0.001) | 0.000(0.000,0.000) | -95.685(-96.895,-93.756) | -8.298(-11.040,-5.471) | -0.000(-0.000,-0.000) |
| Spain | 0.000(0.000,0.000) | 0.000(0.000,0.000) | -51.166(-63.401,-34.038) | -0.582(-1.782,0.632) | -0.000(-0.000,-0.000) |
| Sweden | 0.000(0.000,0.000) | 0.000(0.000,0.000) | 29.120(-9.951,78.438) | -1.471(-4.598,1.758) | 0.000(-0.000,0.000) |
| Switzerland | 0.000(0.000,0.000) | 0.000(0.000,0.000) | 282.823(155.226,436.910) | 3.495(-0.527,7.680) | 0.000(-0.000,0.000) |
| United Kingdom | 0.000(0.000,0.000) | 0.001(0.001,0.001) | 601.884(526.810,684.086) | 6.853(5.142,8.592) | 0.000(0.000,0.000) |
| Argentina | 0.000(0.000,0.000) | 0.000(0.000,0.000) | -18.128(-44.835,11.879) | -2.013(-4.427,0.461) | 0.000(-0.000,0.000) |
| Chile | 0.000(0.000,0.000) | 0.000(0.000,0.000) | 30.117(-1.041,74.148) | 0.530(-1.683,2.793) | 0.000(-0.000,0.000) |
| Uruguay | 0.000(0.000,0.000) | 0.000(0.000,0.000) | -70.088(-79.390,-54.146) | -3.817(-4.226,-3.407) | -0.000(-0.000,-0.000) |
| Canada | 0.000(0.000,0.000) | 0.000(0.000,0.000) | 2977.256(1943.748,4570.856) | 9.604(6.341,12.966) | 0.000(0.000,0.000) |
| United States of America | 0.001(0.001,0.001) | 0.002(0.002,0.002) | 178.828(149.398,209.985) | 2.268(1.761,2.777) | 0.000(0.000,0.000) |
| Antigua and Barbuda | 0.007(0.005,0.009) | 0.005(0.004,0.007) | -25.663(-50.066,11.350) | -0.759(-0.986,-0.532) | -0.000(-0.000,-0.000) |
| Bahamas | 0.003(0.002,0.004) | 0.002(0.002,0.004) | -3.834(-40.394,57.821) | -0.208(-0.417,0.001) | -0.000(-0.000,0.000) |
| Barbados | 0.003(0.002,0.004) | 0.008(0.006,0.010) | 176.033(87.231,286.099) | 3.656(2.286,5.045) | 0.000(0.000,0.000) |
| Belize | 0.001(0.001,0.001) | 0.000(0.000,0.000) | -54.944(-69.280,-33.593) | -1.238(-2.472,0.012) | -0.000(-0.000,-0.000) |
| Cuba | 0.000(0.000,0.000) | 0.000(0.000,0.000) | -25.244(-49.967,16.451) | -0.099(-3.134,3.031) | -0.000(-0.000,-0.000) |
| Dominica | 0.008(0.004,0.012) | 0.004(0.002,0.008) | -42.650(-66.637,-3.721) | -1.736(-2.018,-1.452) | -0.000(-0.000,-0.000) |
| Dominican Republic | 0.006(0.002,0.012) | 0.003(0.001,0.008) | -44.431(-78.990,38.447) | -1.224(-1.879,-0.566) | -0.000(-0.000,-0.000) |
| Grenada | 0.010(0.007,0.013) | 0.008(0.005,0.011) | -19.072(-43.427,18.020) | 0.318(0.002,0.635) | -0.000(-0.000,-0.000) |
| Guyana | 0.002(0.001,0.003) | 0.001(0.001,0.001) | -50.414(-73.310,-11.942) | -1.716(-2.532,-0.894) | -0.000(-0.000,-0.000) |
| Haiti | 0.007(0.002,0.019) | 0.003(0.001,0.009) | -47.970(-74.215,0.843) | -2.371(-2.474,-2.268) | -0.000(-0.000,-0.000) |
| Jamaica | 0.000(0.000,0.000) | 0.001(0.000,0.001) | 4330.168(2520.385,6449.208) | 9.597(3.127,16.474) | 0.000(-0.000,0.000) |
| Saint Lucia | 0.000(0.000,0.001) | 0.000(0.000,0.002) | 11.418(-45.838,294.743) | -1.879(-7.167,3.711) | -0.000(-0.000,0.000) |
| Saint Vincent and the Grenadines | 0.033(0.026,0.041) | 0.001(0.001,0.002) | -96.368(-97.683,-93.111) | 5.717(-2.452,14.570) | -0.001(-0.001,-0.001) |
| Suriname | 0.000(0.000,0.000) | 0.001(0.000,0.001) | 468.008(142.086,1230.329) | 1.828(-1.547,5.319) | 0.000(0.000,0.000) |
| Trinidad and Tobago | 0.000(0.000,0.000) | 0.001(0.001,0.002) | 2920.285(1710.016,4984.482) | 6.248(1.509,11.207) | 0.000(-0.000,0.000) |
| Bolivia (Plurinational State of) | 0.018(0.009,0.032) | 0.000(0.000,0.001) | -97.454(-98.927,-94.057) | -11.927(-12.853,-10.991) | -0.001(-0.001,-0.001) |
| Ecuador | 0.148(0.126,0.176) | 0.000(0.000,0.000) | -99.983(-99.989,-99.974) | -28.735(-31.869,-25.457) | -0.006(-0.007,-0.005) |
| Peru | 0.010(0.003,0.016) | 0.000(0.000,0.000) | -98.746(-99.635,-90.810) | -12.285(-13.991,-10.544) | -0.000(-0.000,-0.000) |
| Colombia | 0.007(0.006,0.008) | 0.000(0.000,0.000) | -99.680(-99.769,-99.547) | -14.906(-18.320,-11.350) | -0.000(-0.000,-0.000) |
| Costa Rica | 0.000(0.000,0.000) | 0.000(0.000,0.000) | -44.216(-81.865,66.795) | -1.665(-2.413,-0.911) | -0.000(-0.000,-0.000) |
| El Salvador | 0.026(0.005,0.046) | 0.000(0.000,0.000) | -99.716(-99.869,-97.211) | -20.807(-22.821,-18.741) | -0.001(-0.001,-0.001) |
| Guatemala | 0.093(0.078,0.114) | 0.000(0.000,0.000) | -99.842(-99.893,-99.789) | -18.955(-22.359,-15.402) | -0.003(-0.003,-0.003) |
| Honduras | 0.000(0.000,0.000) | 0.000(0.000,0.000) | -69.424(-82.809,-48.438) | -4.028(-4.200,-3.855) | -0.000(-0.000,-0.000) |
| Mexico | 0.066(0.061,0.072) | 0.000(0.000,0.000) | -99.931(-99.944,-99.918) | -20.092(-20.900,-19.274) | -0.002(-0.002,-0.002) |
| Nicaragua | 0.000(0.000,0.000) | 0.000(0.000,0.000) | -87.764(-94.034,-75.698) | -8.777(-10.349,-7.178) | -0.000(-0.000,-0.000) |
| Panama | 0.001(0.001,0.002) | 0.000(0.000,0.000) | -83.339(-89.375,-71.303) | -4.694(-5.077,-4.310) | -0.000(-0.000,-0.000) |
| Venezuela (Bolivarian Republic of) | 0.001(0.001,0.001) | 0.000(0.000,0.000) | -97.904(-98.610,-96.917) | -15.546(-18.974,-11.972) | -0.000(-0.000,0.000) |
| Brazil | 0.051(0.037,0.068) | 0.000(0.000,0.000) | -99.645(-99.745,-99.493) | -15.851(-17.401,-14.272) | -0.002(-0.002,-0.002) |
| Paraguay | 0.007(0.003,0.011) | 0.000(0.000,0.000) | -99.394(-99.759,-90.320) | -21.839(-24.736,-18.831) | -0.000(-0.000,-0.000) |
| Algeria | 0.001(0.000,0.002) | 0.001(0.000,0.001) | -44.482(-68.241,31.732) | -1.690(-1.820,-1.560) | -0.000(-0.000,-0.000) |
| Bahrain | 0.005(0.002,0.010) | 0.002(0.001,0.003) | -66.589(-79.693,-38.874) | -3.882(-4.105,-3.659) | -0.000(-0.000,-0.000) |
| Egypt | 0.147(0.080,0.212) | 0.008(0.004,0.013) | -94.320(-96.502,-89.897) | -8.880(-9.453,-8.304) | -0.005(-0.005,-0.004) |
| Iran (Islamic Republic of) | 0.003(0.001,0.005) | 0.001(0.001,0.002) | -53.071(-73.915,-6.569) | -0.288(-1.254,0.688) | -0.000(-0.000,-0.000) |
| Iraq | 0.017(0.005,0.035) | 0.006(0.002,0.011) | -66.568(-85.930,-24.221) | -2.367(-3.005,-1.724) | -0.000(-0.000,-0.000) |
| Jordan | 0.000(0.000,0.000) | 0.000(0.000,0.000) | -49.941(-71.835,-7.860) | -1.932(-2.396,-1.465) | -0.000(-0.000,-0.000) |
| Kuwait | 0.000(0.000,0.001) | 0.000(0.000,0.001) | 35.815(-48.550,229.185) | -4.916(-7.008,-2.777) | 0.000(0.000,0.000) |
| Lebanon | 0.000(0.000,0.000) | 0.000(0.000,0.000) | -56.852(-77.281,-11.204) | -2.187(-2.424,-1.949) | -0.000(-0.000,-0.000) |
| Libya | 0.001(0.000,0.002) | 0.001(0.000,0.002) | -7.585(-58.760,120.919) | 0.199(-0.200,0.600) | -0.000(-0.000,-0.000) |
| Morocco | 0.002(0.000,0.004) | 0.001(0.000,0.002) | -51.568(-75.172,7.691) | -1.893(-2.121,-1.664) | -0.000(-0.000,-0.000) |
| Palestine | 0.001(0.000,0.003) | 0.000(0.000,0.001) | -69.351(-86.787,-3.865) | -3.340(-3.670,-3.008) | -0.000(-0.000,-0.000) |
| Oman | 0.000(0.000,0.000) | 0.000(0.000,0.000) | -66.767(-87.756,18.648) | -3.066(-3.431,-2.700) | -0.000(-0.000,-0.000) |
| Qatar | 0.000(0.000,0.000) | 0.000(0.000,0.000) | -48.221(-75.037,30.003) | -3.466(-5.366,-1.527) | -0.000(-0.000,0.000) |
| Saudi Arabia | 0.000(0.000,0.001) | 0.000(0.000,0.000) | -75.515(-88.914,-39.915) | -5.351(-6.159,-4.536) | -0.000(-0.000,-0.000) |
| Syrian Arab Republic | 0.003(0.001,0.008) | 0.004(0.001,0.009) | 12.797(-61.739,248.953) | 1.518(0.506,2.540) | 0.000(-0.000,0.000) |
| Tunisia | 0.001(0.000,0.002) | 0.000(0.000,0.001) | -50.090(-75.198,30.345) | -2.136(-2.207,-2.066) | -0.000(-0.000,-0.000) |
| Türkiye | 0.011(0.004,0.026) | 0.002(0.001,0.005) | -82.179(-90.339,-63.870) | -5.491(-5.701,-5.280) | -0.000(-0.000,-0.000) |
| United Arab Emirates | 0.043(0.012,0.106) | 0.017(0.007,0.043) | -58.952(-85.205,42.377) | -1.752(-2.164,-1.338) | -0.001(-0.001,-0.001) |
| Yemen | 0.002(0.000,0.006) | 0.002(0.000,0.004) | -19.367(-65.360,149.838) | -0.808(-1.041,-0.575) | -0.000(-0.000,-0.000) |
| Afghanistan | 0.003(0.000,0.009) | 0.002(0.000,0.005) | -21.743(-57.197,116.823) | -1.154(-1.760,-0.545) | -0.000(-0.000,-0.000) |
| Bangladesh | 0.322(0.101,0.546) | 0.024(0.010,0.042) | -92.607(-96.377,-82.889) | -10.043(-10.938,-9.140) | -0.010(-0.011,-0.009) |
| Bhutan | 1.320(0.278,2.712) | 0.198(0.074,0.377) | -84.966(-91.444,-66.863) | -6.613(-6.974,-6.251) | -0.037(-0.037,-0.036) |
| India | 1.654(1.214,2.038) | 0.305(0.191,0.431) | -81.528(-85.945,-75.283) | -5.902(-6.166,-5.637) | -0.044(-0.045,-0.043) |
| Nepal | 6.239(3.014,10.708) | 1.711(0.922,2.647) | -72.569(-84.423,-52.467) | -4.391(-4.505,-4.278) | -0.149(-0.150,-0.147) |
| Pakistan | 1.381(0.708,2.397) | 0.313(0.189,0.470) | -77.366(-85.677,-61.122) | -5.280(-5.522,-5.037) | -0.035(-0.035,-0.035) |
| Angola | 0.046(0.010,0.117) | 0.016(0.003,0.040) | -64.053(-82.099,-33.593) | -3.313(-3.534,-3.091) | -0.001(-0.001,-0.001) |
| Central African Republic | 0.045(0.006,0.131) | 0.033(0.006,0.083) | -26.920(-58.849,42.742) | -1.096(-1.238,-0.954) | -0.000(-0.000,-0.000) |
| Congo | 0.021(0.004,0.055) | 0.009(0.001,0.022) | -59.855(-79.469,-33.251) | -2.889(-3.005,-2.772) | -0.000(-0.000,-0.000) |
| Democratic Republic of the Congo | 0.031(0.004,0.080) | 0.021(0.004,0.050) | -31.443(-60.782,19.896) | -1.054(-1.306,-0.801) | -0.000(-0.000,-0.000) |
| Equatorial Guinea | 0.037(0.004,0.095) | 0.010(0.002,0.024) | -73.155(-89.497,38.776) | -5.041(-5.321,-4.759) | -0.001(-0.001,-0.001) |
| Gabon | 0.022(0.006,0.049) | 0.008(0.001,0.020) | -65.032(-83.517,-32.274) | -3.141(-3.233,-3.049) | -0.000(-0.000,-0.000) |
| Burundi | 0.549(0.248,1.056) | 0.332(0.074,1.005) | -39.495(-79.686,30.693) | -1.767(-2.032,-1.502) | -0.007(-0.007,-0.007) |
| Comoros | 0.395(0.150,0.878) | 0.202(0.062,0.497) | -48.926(-74.669,0.852) | -2.214(-2.504,-1.923) | -0.007(-0.008,-0.006) |
| Djibouti | 0.213(0.056,0.551) | 0.158(0.048,0.386) | -25.587(-58.304,59.475) | -1.026(-1.448,-0.603) | -0.002(-0.002,-0.002) |
| Eritrea | 0.585(0.325,1.047) | 0.309(0.134,0.577) | -47.141(-71.355,-9.689) | -1.833(-1.970,-1.696) | -0.009(-0.009,-0.009) |
| Ethiopia | 3.798(1.085,9.803) | 1.051(0.311,3.022) | -72.325(-85.354,-52.736) | -4.503(-4.784,-4.220) | -0.090(-0.091,-0.089) |
| Kenya | 0.371(0.162,0.693) | 0.200(0.089,0.359) | -46.073(-67.089,-10.162) | -1.390(-1.817,-0.960) | -0.006(-0.006,-0.006) |
| Madagascar | 0.267(0.120,0.463) | 0.201(0.088,0.361) | -24.859(-57.827,26.280) | -0.757(-1.097,-0.415) | -0.002(-0.002,-0.002) |
| Malawi | 1.391(0.712,2.379) | 0.769(0.374,1.384) | -44.695(-74.665,43.858) | -2.055(-2.319,-1.790) | -0.020(-0.020,-0.019) |
| Mauritius | 0.016(0.013,0.019) | 0.000(0.000,0.000) | -99.775(-99.837,-99.670) | -11.896(-14.906,-8.779) | -0.001(-0.001,-0.000) |
| Mozambique | 0.841(0.432,1.306) | 0.441(0.195,0.807) | -47.538(-71.462,-9.015) | -1.621(-1.821,-1.420) | -0.013(-0.013,-0.012) |
| Rwanda | 0.631(0.265,1.488) | 0.227(0.039,0.775) | -64.023(-88.150,-20.892) | -4.125(-4.551,-3.697) | -0.013(-0.014,-0.013) |
| Seychelles | 0.000(0.000,0.000) | 0.001(0.000,0.001) | 113.941(13.389,262.716) | 2.037(0.236,3.871) | 0.000(0.000,0.000) |
| Somalia | 0.580(0.098,1.522) | 0.631(0.118,1.870) | 8.875(-47.670,94.155) | 0.289(0.075,0.503) | 0.002(0.002,0.002) |
| United Republic of Tanzania | 0.549(0.280,0.912) | 0.264(0.096,0.559) | -51.858(-80.129,-0.266) | -2.300(-2.561,-2.039) | -0.009(-0.010,-0.009) |
| Uganda | 0.312(0.112,0.696) | 0.194(0.066,0.393) | -37.885(-69.666,46.913) | -2.048(-2.429,-1.665) | -0.004(-0.004,-0.004) |
| Zambia | 0.698(0.406,1.137) | 0.290(0.131,0.525) | -58.451(-79.003,-16.221) | -3.012(-3.497,-2.524) | -0.013(-0.014,-0.013) |
| Botswana | 0.068(0.027,0.143) | 0.032(0.015,0.060) | -53.487(-79.587,1.504) | -2.248(-2.438,-2.057) | -0.001(-0.001,-0.001) |
| Lesotho | 0.085(0.034,0.183) | 0.061(0.029,0.111) | -28.020(-66.783,57.729) | -0.479(-0.845,-0.112) | -0.001(-0.001,-0.001) |
| Namibia | 0.060(0.026,0.115) | 0.035(0.014,0.070) | -41.556(-68.830,10.748) | -1.728(-1.870,-1.585) | -0.001(-0.001,-0.001) |
| South Africa | 0.039(0.020,0.064) | 0.020(0.011,0.031) | -48.920(-67.385,-17.084) | -2.199(-2.986,-1.405) | -0.001(-0.001,-0.001) |
| Eswatini | 0.080(0.037,0.157) | 0.047(0.024,0.085) | -40.490(-70.498,27.567) | -1.320(-1.502,-1.137) | -0.001(-0.001,-0.001) |
| Zimbabwe | 0.121(0.056,0.215) | 0.118(0.062,0.215) | -1.933(-50.910,99.778) | 0.356(-0.134,0.849) | -0.000(-0.000,-0.000) |
| Benin | 0.405(0.106,0.951) | 0.265(0.087,0.563) | -34.576(-67.703,52.116) | -1.246(-1.375,-1.116) | -0.005(-0.005,-0.004) |
| Burkina Faso | 0.580(0.162,1.354) | 0.310(0.103,0.680) | -46.532(-72.193,7.613) | -2.289(-2.501,-2.076) | -0.009(-0.009,-0.009) |
| Cameroon | 0.327(0.099,0.647) | 0.206(0.081,0.375) | -36.965(-66.258,33.811) | -1.217(-1.465,-0.968) | -0.004(-0.004,-0.004) |
| Cabo Verde | 0.234(0.061,0.563) | 0.094(0.034,0.186) | -59.944(-80.228,-4.489) | -3.289(-3.525,-3.054) | -0.005(-0.005,-0.004) |
| Chad | 0.546(0.088,1.446) | 0.388(0.054,0.978) | -28.894(-61.588,36.149) | -1.014(-1.130,-0.898) | -0.005(-0.005,-0.004) |
| Côte d'Ivoire | 0.335(0.088,0.702) | 0.223(0.069,0.431) | -33.549(-62.286,22.791) | -0.946(-1.181,-0.711) | -0.004(-0.004,-0.003) |
| Gambia | 0.350(0.077,0.837) | 0.238(0.091,0.513) | -31.843(-64.547,56.232) | -1.397(-1.624,-1.170) | -0.004(-0.004,-0.003) |
| Ghana | 0.757(0.339,1.290) | 0.415(0.190,0.711) | -45.148(-69.776,-2.286) | -1.478(-1.800,-1.154) | -0.011(-0.011,-0.010) |
| Guinea | 0.562(0.156,1.217) | 0.317(0.082,0.652) | -43.686(-70.840,2.532) | -1.405(-1.591,-1.218) | -0.008(-0.008,-0.007) |
| Guinea-Bissau | 0.570(0.152,1.311) | 0.313(0.087,0.672) | -45.112(-68.773,-1.256) | -1.489(-1.657,-1.319) | -0.008(-0.009,-0.008) |
| Liberia | 0.364(0.082,0.803) | 0.201(0.038,0.476) | -44.734(-74.291,-2.664) | -2.233(-2.438,-2.027) | -0.006(-0.007,-0.006) |
| Mali | 0.614(0.093,1.696) | 0.329(0.064,0.803) | -46.396(-69.725,-3.608) | -1.904(-2.107,-1.702) | -0.009(-0.009,-0.009) |
| Mauritania | 0.328(0.116,0.646) | 0.131(0.040,0.259) | -60.084(-77.649,-27.675) | -2.721(-2.897,-2.544) | -0.006(-0.007,-0.006) |
| Niger | 0.722(0.122,2.053) | 0.473(0.078,1.287) | -34.450(-73.979,25.247) | -1.486(-1.640,-1.331) | -0.008(-0.009,-0.007) |
| Nigeria | 0.706(0.353,1.169) | 0.361(0.149,0.659) | -48.822(-73.564,-7.187) | -2.048(-2.329,-1.767) | -0.011(-0.012,-0.011) |
| Sao Tome and Principe | 0.273(0.067,0.600) | 0.120(0.040,0.232) | -56.013(-74.225,-3.620) | -2.813(-3.078,-2.548) | -0.005(-0.005,-0.005) |
| Senegal | 0.502(0.160,1.063) | 0.230(0.078,0.480) | -54.106(-74.188,-17.748) | -2.138(-2.343,-1.932) | -0.009(-0.010,-0.008) |
| Sierra Leone | 0.439(0.148,0.961) | 0.232(0.057,0.552) | -47.195(-75.292,0.056) | -1.986(-2.305,-1.666) | -0.007(-0.007,-0.007) |
| Togo | 0.399(0.158,0.835) | 0.240(0.091,0.493) | -39.860(-67.668,4.690) | -1.439(-1.592,-1.286) | -0.005(-0.005,-0.005) |
| American Samoa | 0.029(0.011,0.054) | 0.016(0.006,0.029) | -46.198(-76.411,14.117) | -1.941(-2.089,-1.793) | -0.000(-0.000,-0.000) |
| Bermuda | 0.006(0.004,0.007) | 0.004(0.002,0.005) | -34.764(-60.526,4.566) | -0.992(-1.408,-0.575) | -0.000(-0.000,-0.000) |
| Cook Islands | 0.056(0.012,0.128) | 0.021(0.004,0.038) | -62.947(-84.238,35.832) | -3.441(-3.717,-3.164) | -0.001(-0.001,-0.001) |
| Greenland | 0.002(0.001,0.003) | 0.003(0.001,0.005) | 43.242(-28.527,169.535) | 2.982(1.930,4.045) | 0.000(-0.000,0.000) |
| Guam | 0.009(0.004,0.016) | 0.004(0.002,0.007) | -55.768(-73.819,-27.947) | -1.564(-1.969,-1.158) | -0.000(-0.000,-0.000) |
| Monaco | 0.004(0.002,0.007) | 0.002(0.001,0.003) | -52.873(-76.906,-8.757) | -2.099(-2.964,-1.225) | -0.000(-0.000,-0.000) |
| Nauru | 0.033(0.010,0.069) | 0.027(0.009,0.055) | -18.103(-62.396,88.511) | -0.626(-1.039,-0.212) | -0.000(-0.000,-0.000) |
| Niue | 0.014(0.002,0.045) | 0.009(0.002,0.028) | -34.335(-73.617,73.187) | -1.680(-1.817,-1.543) | -0.000(-0.000,-0.000) |
| Northern Mariana Islands | 0.039(0.017,0.080) | 0.029(0.014,0.055) | -26.288(-56.212,33.661) | -0.866(-1.175,-0.555) | -0.000(-0.001,-0.000) |
| Palau | 0.004(0.000,0.012) | 0.003(0.000,0.009) | -24.009(-69.966,113.926) | -1.126(-1.269,-0.983) | -0.000(-0.000,-0.000) |
| Puerto Rico | 0.000(0.000,0.000) | 0.000(0.000,0.000) | 505.654(202.754,769.000) | 6.815(3.746,9.973) | 0.000(-0.000,0.000) |
| Saint Kitts and Nevis | 0.022(0.017,0.027) | 0.015(0.011,0.019) | -31.753(-47.839,-11.041) | -0.968(-1.110,-0.826) | -0.000(-0.000,-0.000) |
| San Marino | 0.014(0.005,0.031) | 0.000(0.000,0.000) | -99.096(-99.673,-97.776) | -18.990(-21.205,-16.714) | -0.000(-0.001,-0.000) |
| Tokelau | 0.019(0.002,0.065) | 0.010(0.002,0.033) | -45.428(-75.081,44.118) | -2.349(-2.522,-2.176) | -0.000(-0.000,-0.000) |
| Tuvalu | 0.067(0.019,0.184) | 0.027(0.011,0.058) | -60.229(-84.542,9.453) | -2.615(-2.746,-2.484) | -0.001(-0.001,-0.001) |
| United States Virgin Islands | 0.001(0.001,0.003) | 0.001(0.000,0.001) | -48.689(-77.609,-1.645) | -1.586(-2.057,-1.113) | -0.000(-0.000,-0.000) |
| South Sudan | 0.308(0.064,0.721) | 0.320(0.065,0.758) | 3.860(-43.925,81.725) | -0.132(-0.383,0.119) | 0.001(0.000,0.001) |
| Sudan | 0.002(0.001,0.005) | 0.001(0.000,0.002) | -52.978(-77.674,10.710) | -2.392(-2.649,-2.135) | -0.000(-0.000,-0.000) |

Abbreviations: ASIR,Age standardized incidence rate; AAPC, average annual percent change; CI, confidence interval; EAPC, estimated annual percentage change; UI, uncertainty interval.

Supplementary Table 6: Trends in the number of rabies incident cases in 204 countries and territories worldwide from 1990 to 2021.

| Location | Rabies. Incidence cases (persons) . (95% UI).  1990 year | Rabies. Incidence cases (persons) . (95% UI).  2021 year | Rabies. Percentage change (95% UI). 1990－2021. | Rabies. EAPC (95% CI). 1990－2021. | Rabies. AAPC (95% CI). 1990－2021. |
| --- | --- | --- | --- | --- | --- |
| China | 1093(642,1511) | 610(322,931) | -44.178(-62.404,-19.521) | -0.368(-2.380,1.684) | -15.601(-20.695,-10.507) |
| Democratic People's Republic of Korea | 10(4,19) | 17(7,29) | 57.921(-5.570,165.809) | 0.572(0.163,0.983) | 0.188(0.179,0.196) |
| Taiwan (Province of China) | 0(0,0) | 0(0,0) | 17.151(-21.811,64.623) | 2.289(0.150,4.473) | -0.000(-0.001,0.001) |
| Cambodia | 50(18,88) | 26(8,49) | -48.580(-74.063,-13.049) | -4.811(-5.305,-4.314) | -0.823(-0.853,-0.793) |
| Indonesia | 133(79,201) | 49(26,83) | -63.347(-82.635,-28.277) | -4.398(-4.641,-4.155) | -2.745(-2.784,-2.706) |
| Lao People's Democratic Republic | 23(10,41) | 12(4,21) | -48.022(-74.413,1.956) | -4.389(-4.855,-3.920) | -0.360(-0.370,-0.349) |
| Malaysia | 1(0,1) | 0(0,1) | -46.303(-71.147,14.515) | -3.837(-4.133,-3.541) | -0.013(-0.014,-0.011) |
| Maldives | 0(0,0) | 0(0,0) | -45.178(-76.348,27.412) | -4.204(-4.605,-3.800) | -0.000(-0.000,-0.000) |
| Myanmar | 539(316,922) | 239(109,433) | -55.699(-80.739,-10.255) | -3.922(-4.125,-3.719) | -9.785(-9.939,-9.630) |
| Philippines | 510(402,627) | 252(152,355) | -50.520(-64.987,-34.942) | -5.022(-5.628,-4.412) | -8.423(-8.839,-8.006) |
| Sri Lanka | 88(45,141) | 22(8,38) | -75.532(-88.459,-52.503) | -7.461(-8.351,-6.562) | -2.069(-2.219,-1.919) |
| Thailand | 19(8,36) | 5(2,8) | -76.110(-87.198,-53.559) | -6.800(-10.204,-3.266) | -0.485(-0.572,-0.398) |
| Timor-Leste | 3(1,6) | 2(1,4) | -28.150(-62.892,40.984) | -3.357(-3.603,-3.110) | -0.030(-0.031,-0.028) |
| Viet Nam | 101(48,164) | 56(28,93) | -44.395(-67.940,8.894) | -3.258(-3.348,-3.168) | -1.537(-1.592,-1.483) |
| Fiji | 0(0,0) | 0(0,0) | 119.715(-17.336,481.840) | 2.861(2.241,3.485) | 0.001(0.001,0.001) |
| Kiribati | 0(0,0) | 0(0,0) | 40.297(-41.647,245.045) | -0.629(-0.723,-0.536) | 0.000(0.000,0.000) |
| Marshall Islands | 0(0,0) | 0(0,0) | -8.125(-61.329,121.165) | -1.258(-1.397,-1.119) | -0.000(-0.000,-0.000) |
| Micronesia (Federated States of) | 0(0,0) | 0(0,0) | -39.081(-72.899,35.476) | -1.415(-1.518,-1.312) | -0.000(-0.000,-0.000) |
| Papua New Guinea | 0(0,2) | 1(0,4) | 160.477(9.745,531.641) | -0.162(-0.331,0.008) | 0.024(0.024,0.025) |
| Samoa | 0(0,0) | 0(0,0) | 27.887(-38.731,186.639) | 0.287(0.082,0.492) | 0.000(0.000,0.000) |
| Solomon Islands | 0(0,0) | 0(0,0) | 136.372(6.433,695.231) | 0.568(0.315,0.822) | 0.001(0.001,0.002) |
| Tonga | 0(0,0) | 0(0,0) | 14.567(-50.411,186.603) | 0.356(0.151,0.561) | 0.000(0.000,0.000) |
| Vanuatu | 0(0,0) | 0(0,0) | 116.552(-1.358,434.478) | 0.096(-0.029,0.221) | 0.001(0.001,0.001) |
| Armenia | 0(0,0) | 0(0,0) | -79.670(-88.348,-63.452) | -4.430(-7.582,-1.170) | -0.001(-0.002,-0.000) |
| Azerbaijan | 3(1,7) | 2(0,4) | -45.217(-84.719,101.431) | -4.798(-5.855,-3.729) | -0.043(-0.048,-0.039) |
| Georgia | 1(1,2) | 1(0,1) | -41.415(-73.498,29.446) | -0.976(-3.661,1.783) | -0.029(-0.057,-0.001) |
| Kazakhstan | 2(1,3) | 3(2,6) | 78.973(-13.109,273.223) | 0.645(-1.392,2.725) | 0.034(0.006,0.062) |
| Kyrgyzstan | 1(1,2) | 1(0,1) | -62.407(-86.025,-8.234) | -5.097(-6.992,-3.164) | -0.025(-0.034,-0.015) |
| Mongolia | 5(0,15) | 2(0,5) | -64.715(-93.237,87.188) | -5.890(-6.319,-5.460) | -0.093(-0.099,-0.087) |
| Tajikistan | 0(0,0) | 0(0,0) | -24.131(-80.621,184.598) | -3.827(-4.164,-3.490) | -0.000(-0.000,-0.000) |
| Turkmenistan | 0(0,0) | 0(0,0) | -58.830(-83.042,2.886) | -3.760(-10.811,3.850) | 0.016(-0.005,0.038) |
| Uzbekistan | 2(1,3) | 1(1,2) | -2.749(-50.271,90.417) | -2.993(-3.479,-2.505) | 0.000(-0.005,0.005) |
| Albania | 0(0,0) | 0(0,0) | -82.272(-94.245,-38.272) | -7.721(-9.155,-6.264) | -0.000(-0.000,-0.000) |
| Bosnia and Herzegovina | 0(0,0) | 0(0,0) | -78.965(-94.704,-29.588) | -3.417(-4.265,-2.561) | -0.000(-0.000,-0.000) |
| Bulgaria | 0(0,0) | 0(0,0) | 272.627(130.808,482.895) | 5.876(4.726,7.038) | 0.002(0.001,0.002) |
| Croatia | 0(0,0) | 0(0,0) | -96.246(-97.806,-93.570) | -14.039(-16.109,-11.919) | -0.001(-0.002,-0.001) |
| Czechia | 0(0,0) | 0(0,0) | -55.916(-71.337,-30.742) | -3.871(-6.677,-0.982) | -0.000(-0.001,0.000) |
| Hungary | 0(0,0) | 0(0,0) | 69.853(20.005,147.647) | -4.590(-7.536,-1.551) | 0.001(-0.001,0.003) |
| North Macedonia | 0(0,0) | 0(0,0) | -42.864(-77.780,41.110) | -4.262(-5.538,-2.968) | -0.000(-0.000,-0.000) |
| Montenegro | 0(0,0) | 0(0,0) | -19.906(-69.094,80.578) | -0.988(-1.276,-0.700) | -0.000(-0.000,-0.000) |
| Poland | 1(1,1) | 0(0,0) | -99.095(-99.277,-98.683) | -18.007(-20.023,-15.941) | -0.029(-0.036,-0.022) |
| Romania | 3(2,4) | 0(0,0) | -99.736(-99.814,-99.630) | -18.314(-20.019,-16.572) | -0.114(-0.127,-0.102) |
| Serbia | 0(0,0) | 0(0,0) | -64.418(-83.262,-17.815) | -3.470(-4.022,-2.915) | -0.001(-0.001,-0.001) |
| Slovakia | 0(0,0) | 0(0,0) | -61.525(-80.370,-14.854) | -2.993(-3.084,-2.902) | -0.000(-0.000,-0.000) |
| Slovenia | 0(0,0) | 0(0,0) | 64.673(20.633,135.224) | -3.116(-5.923,-0.226) | 0.000(0.000,0.000) |
| Belarus | 0(0,0) | 2(1,2) | 504.896(259.102,832.175) | 6.044(3.911,8.222) | 0.054(0.044,0.064) |
| Estonia | 0(0,0) | 0(0,0) | 208.715(90.470,387.017) | 5.079(1.818,8.444) | 0.000(-0.000,0.001) |
| Latvia | 0(0,1) | 0(0,0) | -80.037(-87.184,-68.973) | -4.319(-6.286,-2.310) | -0.010(-0.013,-0.008) |
| Lithuania | 0(0,0) | 0(0,0) | -98.570(-99.260,-97.323) | -13.050(-17.263,-8.623) | -0.003(-0.003,-0.002) |
| Republic of Moldova | 0(0,0) | 0(0,0) | -84.084(-90.639,-73.267) | -4.618(-5.841,-3.378) | -0.000(-0.001,-0.000) |
| Russian Federation | 10(9,11) | 2(2,2) | -77.335(-79.701,-74.678) | -3.468(-5.065,-1.845) | -0.251(-0.306,-0.197) |
| Ukraine | 1(1,2) | 1(1,2) | -13.057(-58.781,89.647) | 1.525(0.507,2.554) | -0.019(-0.028,-0.011) |
| Brunei Darussalam | 0(0,0) | 0(0,0) | -9.784(-45.658,52.878) | -1.198(-1.915,-0.477) | -0.000(-0.000,-0.000) |
| Japan | 1(1,1) | 0(0,0) | -59.069(-67.481,-52.885) | -1.777(-3.362,-0.166) | -0.021(-0.025,-0.017) |
| Republic of Korea | 0(0,0) | 0(0,0) | -93.098(-96.833,-66.120) | -10.528(-12.550,-8.459) | -0.004(-0.004,-0.003) |
| Singapore | 0(0,0) | 0(0,0) | 118.009(37.910,205.369) | -2.202(-3.857,-0.519) | 0.000(-0.000,0.000) |
| Australia | 0(0,0) | 0(0,0) | 271.691(125.229,494.230) | 7.626(4.496,10.850) | 0.001(0.000,0.002) |
| New Zealand | 0(0,0) | 0(0,0) | -36.002(-56.820,-5.310) | -1.344(-2.563,-0.110) | -0.000(-0.000,-0.000) |
| Andorra | 0(0,0) | 0(0,0) | 56.451(-23.272,204.591) | -0.101(-0.474,0.275) | 0.000(0.000,0.000) |
| Austria | 0(0,0) | 0(0,0) | 24.782(-9.515,67.398) | -0.436(-2.359,1.524) | 0.000(0.000,0.000) |
| Belgium | 0(0,0) | 0(0,0) | -40.286(-58.184,-16.460) | 2.385(-0.048,4.877) | -0.000(-0.001,0.000) |
| Cyprus | 0(0,0) | 0(0,0) | -33.100(-71.315,82.779) | -2.175(-3.407,-0.927) | -0.000(-0.000,-0.000) |
| Denmark | 0(0,0) | 0(0,0) | -77.970(-83.405,-70.754) | -5.165(-6.835,-3.466) | -0.002(-0.002,-0.001) |
| Finland | 0(0,0) | 0(0,0) | -16.624(-50.402,30.308) | -1.122(-1.542,-0.700) | -0.000(-0.000,0.000) |
| France | 0(0,0) | 0(0,0) | 50.207(-0.651,124.036) | -1.647(-3.611,0.356) | -0.000(-0.002,0.001) |
| Germany | 0(0,0) | 0(0,0) | 148.786(56.867,264.856) | 3.290(1.868,4.732) | 0.000(-0.000,0.001) |
| Greece | 0(0,0) | 1(0,1) | 3295.864(2152.586,5192.052) | 18.513(14.344,22.834) | 0.021(0.020,0.021) |
| Iceland | 0(0,0) | 0(0,0) | 56.651(7.314,122.793) | -2.051(-5.877,1.930) | 0.000(-0.000,0.001) |
| Ireland | 0(0,0) | 0(0,0) | 19.347(-14.647,71.997) | 1.306(-0.355,2.994) | 0.000(0.000,0.000) |
| Israel | 0(0,0) | 0(0,0) | -78.308(-85.757,-66.866) | -11.063(-13.136,-8.940) | -0.001(-0.001,-0.000) |
| Italy | 0(0,0) | 0(0,0) | -76.074(-79.904,-71.443) | -4.824(-5.817,-3.820) | -0.004(-0.005,-0.002) |
| Luxembourg | 0(0,0) | 0(0,0) | 5.752(-23.872,42.165) | -1.178(-5.142,2.951) | -0.000(-0.000,0.000) |
| Malta | 0(0,0) | 0(0,0) | -95.084(-96.324,-93.429) | -8.528(-11.035,-5.950) | -0.000(-0.000,-0.000) |
| Netherlands | 0(0,0) | 0(0,0) | 352.961(216.765,520.360) | 5.150(2.338,8.039) | 0.000(-0.002,0.002) |
| Norway | 0(0,0) | 1(0,1) | 941.900(746.787,1138.099) | 5.130(3.456,6.832) | 0.019(0.017,0.021) |
| Portugal | 0(0,0) | 0(0,0) | -80.737(-86.697,-70.779) | -3.181(-6.306,0.048) | -0.001(-0.002,0.000) |
| Spain | 0(0,0) | 0(0,0) | 15.341(-16.555,57.158) | 2.153(0.736,3.589) | 0.000(-0.000,0.000) |
| Sweden | 0(0,0) | 0(0,0) | 192.520(105.151,298.592) | 0.961(-1.894,3.900) | 0.001(0.001,0.002) |
| Switzerland | 0(0,0) | 0(0,0) | 917.731(560.075,1401.862) | 5.972(1.742,10.379) | 0.001(-0.000,0.001) |
| United Kingdom | 0(0,0) | 0(0,0) | 735.723(665.855,810.596) | 6.928(5.266,8.615) | 0.013(0.004,0.022) |
| Argentina | 0(0,0) | 0(0,0) | 46.664(0.872,102.461) | -1.205(-3.623,1.274) | 0.000(-0.000,0.000) |
| Chile | 0(0,0) | 0(0,0) | 230.193(135.973,368.415) | 2.009(-0.197,4.264) | 0.000(-0.000,0.000) |
| Uruguay | 0(0,0) | 0(0,0) | -64.654(-75.876,-41.541) | -3.640(-3.945,-3.335) | -0.000(-0.000,-0.000) |
| Canada | 0(0,0) | 0(0,0) | 2177.934(1405.410,3294.931) | 8.653(6.201,11.162) | 0.002(0.002,0.003) |
| United States of America | 2(2,2) | 7(6,7) | 297.261(252.278,339.064) | 2.517(2.010,3.027) | 0.151(0.130,0.173) |
| Antigua and Barbuda | 0(0,0) | 0(0,0) | -0.010(-34.299,52.784) | -1.265(-1.591,-0.938) | 0.000(-0.000,0.000) |
| Bahamas | 0(0,0) | 0(0,0) | 93.308(16.328,213.596) | 0.611(0.375,0.848) | 0.000(0.000,0.000) |
| Barbados | 0(0,0) | 0(0,0) | 348.794(201.131,532.758) | 4.387(2.858,5.938) | 0.001(0.001,0.001) |
| Belize | 0(0,0) | 0(0,0) | -42.876(-61.762,-16.242) | -2.742(-4.161,-1.301) | -0.000(-0.000,0.000) |
| Cuba | 0(0,0) | 0(0,0) | 10.875(-27.638,92.810) | 0.176(-2.748,3.187) | -0.002(-0.004,0.000) |
| Dominica | 0(0,0) | 0(0,0) | -26.429(-56.741,23.498) | -0.738(-1.009,-0.467) | -0.000(-0.000,-0.000) |
| Dominican Republic | 0(0,0) | 0(0,1) | 70.112(-32.186,298.491) | 1.061(0.423,1.704) | 0.004(0.003,0.004) |
| Grenada | 0(0,0) | 0(0,0) | -32.443(-53.062,-3.430) | -0.989(-1.305,-0.672) | -0.000(-0.000,-0.000) |
| Guyana | 0(0,0) | 0(0,0) | -19.111(-55.299,39.958) | 0.503(-0.005,1.013) | -0.000(-0.000,-0.000) |
| Haiti | 0(0,0) | 0(0,0) | 4.890(-46.718,96.712) | -2.237(-2.337,-2.137) | 0.000(0.000,0.000) |
| Jamaica | 0(0,0) | 0(0,0) | 8107.091(4905.421,12121.166) | 11.426(4.863,18.399) | 0.001(0.000,0.001) |
| Saint Lucia | 0(0,0) | 0(0,0) | 222.474(49.587,1060.880) | 1.176(-4.042,6.679) | -0.000(-0.001,0.000) |
| Saint Vincent and the Grenadines | 0(0,0) | 0(0,0) | -92.647(-95.248,-86.091) | 7.589(-0.567,16.414) | -0.001(-0.001,-0.000) |
| Suriname | 0(0,0) | 0(0,0) | 881.295(293.282,2352.913) | 3.111(0.147,6.164) | 0.000(0.000,0.000) |
| Trinidad and Tobago | 0(0,0) | 0(0,0) | 5890.325(3577.574,9650.429) | 8.084(3.425,12.953) | 0.001(-0.000,0.001) |
| Bolivia (Plurinational State of) | 1(0,2) | 0(0,0) | -95.845(-98.378,-89.855) | -12.258(-13.343,-11.160) | -0.030(-0.030,-0.029) |
| Ecuador | 14(12,17) | 0(0,0) | -99.972(-99.982,-99.957) | -29.097(-32.323,-25.717) | -0.475(-0.614,-0.336) |
| Peru | 2(1,3) | 0(0,0) | -98.064(-99.436,-84.919) | -12.227(-14.245,-10.160) | -0.065(-0.066,-0.064) |
| Colombia | 2(2,3) | 0(0,0) | -99.479(-99.627,-99.258) | -14.711(-18.264,-11.004) | -0.071(-0.076,-0.067) |
| Costa Rica | 0(0,0) | 0(0,0) | 23.001(-60.103,267.863) | -0.866(-1.662,-0.063) | 0.000(-0.000,0.000) |
| El Salvador | 1(0,3) | 0(0,0) | -99.642(-99.852,-96.101) | -20.678(-22.732,-18.570) | -0.042(-0.044,-0.041) |
| Guatemala | 7(6,9) | 0(0,0) | -99.719(-99.814,-99.609) | -18.877(-22.337,-15.262) | -0.239(-0.246,-0.232) |
| Honduras | 0(0,0) | 0(0,0) | -41.261(-70.402,6.272) | -4.436(-4.660,-4.213) | -0.000(-0.000,-0.000) |
| Mexico | 54(49,59) | 0(0,0) | -99.900(-99.917,-99.878) | -20.099(-20.914,-19.276) | -1.730(-1.826,-1.635) |
| Nicaragua | 0(0,0) | 0(0,0) | -84.858(-93.219,-68.636) | -9.805(-11.541,-8.036) | -0.000(-0.000,-0.000) |
| Panama | 0(0,0) | 0(0,0) | -57.801(-74.127,-27.666) | -3.482(-3.927,-3.035) | -0.000(-0.000,-0.000) |
| Venezuela (Bolivarian Republic of) | 0(0,0) | 0(0,0) | -95.819(-97.290,-93.768) | -14.703(-18.336,-10.909) | -0.004(-0.015,0.007) |
| Brazil | 78(55,105) | 0(0,0) | -99.484(-99.637,-99.262) | -15.937(-17.466,-14.379) | -2.597(-2.719,-2.474) |
| Paraguay | 0(0,0) | 0(0,0) | -99.003(-99.646,-79.080) | -22.371(-25.425,-19.193) | -0.011(-0.012,-0.010) |
| Algeria | 0(0,0) | 0(0,0) | 13.392(-36.751,142.469) | -1.279(-1.446,-1.111) | 0.001(0.001,0.001) |
| Bahrain | 0(0,0) | 0(0,0) | 12.422(-39.599,122.869) | -3.602(-3.864,-3.339) | 0.000(0.000,0.000) |
| Egypt | 82(43,122) | 8(4,13) | -90.487(-94.343,-81.951) | -9.282(-9.906,-8.653) | -2.443(-2.508,-2.378) |
| Iran (Islamic Republic of) | 1(0,2) | 1(0,2) | -11.832(-50.191,75.222) | 0.525(-0.523,1.584) | -0.006(-0.010,-0.002) |
| Iraq | 4(1,8) | 2(1,5) | -38.523(-78.610,65.551) | -2.893(-3.537,-2.244) | -0.049(-0.053,-0.045) |
| Jordan | 0(0,0) | 0(0,0) | 103.944(12.385,290.206) | -1.066(-1.488,-0.642) | 0.000(0.000,0.000) |
| Kuwait | 0(0,0) | 0(0,0) | 213.026(12.874,765.257) | -4.783(-6.533,-3.001) | 0.001(0.000,0.001) |
| Lebanon | 0(0,0) | 0(0,0) | 19.274(-39.657,143.838) | -0.898(-1.159,-0.637) | 0.000(0.000,0.000) |
| Libya | 0(0,0) | 0(0,0) | 104.614(-7.245,384.663) | 1.035(0.587,1.485) | 0.001(0.001,0.001) |
| Morocco | 0(0,1) | 0(0,1) | -10.762(-53.730,94.758) | -1.071(-1.315,-0.826) | -0.001(-0.001,-0.001) |
| Palestine | 0(0,0) | 0(0,0) | -27.743(-70.674,159.575) | -3.504(-3.716,-3.291) | -0.000(-0.000,-0.000) |
| Oman | 0(0,0) | 0(0,0) | -25.928(-70.054,140.026) | -3.147(-3.439,-2.853) | -0.000(-0.000,-0.000) |
| Qatar | 0(0,0) | 0(0,0) | 579.865(135.413,1602.645) | -1.964(-4.385,0.519) | 0.000(0.000,0.000) |
| Saudi Arabia | 0(0,0) | 0(0,0) | -24.770(-65.774,84.970) | -4.620(-5.560,-3.671) | -0.000(-0.000,-0.000) |
| Syrian Arab Republic | 0(0,0) | 0(0,1) | 56.708(-42.854,283.286) | 3.083(1.721,4.464) | 0.003(0.002,0.004) |
| Tunisia | 0(0,0) | 0(0,0) | -1.580(-52.053,165.975) | -1.024(-1.132,-0.916) | -0.000(-0.000,-0.000) |
| Türkiye | 5(2,13) | 2(1,4) | -67.202(-83.339,-26.880) | -4.630(-4.816,-4.443) | -0.111(-0.114,-0.107) |
| United Arab Emirates | 0(0,1) | 1(0,1) | 25.810(-48.385,345.940) | -4.166(-4.584,-3.746) | 0.004(0.003,0.005) |
| Yemen | 0(0,0) | 0(0,1) | 112.415(-7.408,516.118) | -0.558(-0.817,-0.299) | 0.006(0.006,0.007) |
| Afghanistan | 0(0,1) | 0(0,1) | 99.177(8.197,467.057) | -1.715(-2.171,-1.256) | 0.007(0.007,0.007) |
| Bangladesh | 285(73,568) | 23(9,38) | -92.070(-96.390,-78.735) | -11.804(-13.047,-10.543) | -8.833(-9.411,-8.255) |
| Bhutan | 7(1,15) | 1(0,3) | -80.368(-89.438,-46.853) | -6.529(-6.902,-6.154) | -0.178(-0.183,-0.174) |
| India | 12701(9400,15819) | 4061(2552,5721) | -68.025(-76.011,-56.332) | -5.833(-6.110,-5.555) | -285.624(-297.000,-274.249) |
| Nepal | 1064(519,1771) | 484(264,744) | -54.532(-74.771,-17.204) | -4.236(-4.377,-4.095) | -19.208(-19.611,-18.805) |
| Pakistan | 1360(711,2290) | 620(382,914) | -54.413(-71.856,-21.680) | -5.382(-5.635,-5.129) | -23.928(-24.421,-23.434) |
| Angola | 4(1,12) | 4(1,13) | 2.149(-61.441,111.425) | -3.595(-3.834,-3.356) | 0.002(0.000,0.004) |
| Central African Republic | 1(0,3) | 1(0,5) | 39.541(-34.092,189.334) | -1.221(-1.358,-1.084) | 0.014(0.013,0.015) |
| Congo | 0(0,1) | 0(0,1) | -10.222(-62.735,69.914) | -2.876(-2.998,-2.754) | -0.001(-0.002,-0.001) |
| Democratic Republic of the Congo | 10(2,33) | 15(3,44) | 44.789(-32.749,188.363) | -1.347(-1.623,-1.071) | 0.140(0.125,0.156) |
| Equatorial Guinea | 0(0,0) | 0(0,0) | -16.882(-70.364,269.388) | -5.611(-5.907,-5.315) | -0.001(-0.001,-0.001) |
| Gabon | 0(0,0) | 0(0,0) | -35.774(-71.642,26.567) | -3.097(-3.198,-2.997) | -0.002(-0.002,-0.002) |
| Burundi | 29(13,54) | 40(9,122) | 36.615(-56.170,213.573) | -1.843(-2.129,-1.557) | 0.309(0.290,0.328) |
| Comoros | 2(1,4) | 1(0,4) | -17.125(-60.165,65.419) | -2.193(-2.541,-1.844) | -0.015(-0.020,-0.010) |
| Djibouti | 1(0,2) | 2(1,5) | 128.830(18.799,391.417) | -1.055(-1.451,-0.659) | 0.032(0.031,0.033) |
| Eritrea | 18(10,31) | 19(8,36) | 3.835(-47.892,92.529) | -1.846(-1.978,-1.713) | 0.011(-0.001,0.023) |
| Ethiopia | 1769(534,4353) | 1050(316,2821) | -40.636(-72.213,10.206) | -4.459(-4.699,-4.219) | -23.770(-24.510,-23.031) |
| Kenya | 80(35,156) | 89(38,157) | 10.999(-32.774,83.276) | -1.407(-1.803,-1.010) | 0.267(0.209,0.325) |
| Madagascar | 30(14,50) | 53(23,96) | 77.096(-12.734,212.362) | -0.793(-1.132,-0.453) | 0.707(0.679,0.734) |
| Malawi | 78(40,134) | 82(34,142) | 3.926(-51.494,124.943) | -2.166(-2.478,-1.853) | 0.143(0.087,0.199) |
| Mauritius | 0(0,0) | 0(0,0) | -99.160(-99.374,-98.797) | -8.555(-11.671,-5.330) | -0.002(-0.002,-0.002) |
| Mozambique | 116(60,193) | 124(49,238) | 7.368(-52.826,97.625) | -1.977(-2.183,-1.771) | 0.486(0.384,0.588) |
| Rwanda | 44(19,97) | 28(5,93) | -35.407(-80.560,45.245) | -4.239(-4.661,-3.814) | -0.525(-0.690,-0.360) |
| Seychelles | 0(0,0) | 0(0,0) | 220.939(70.416,441.789) | 1.912(0.066,3.792) | 0.000(0.000,0.000) |
| Somalia | 40(7,107) | 109(21,319) | 171.017(12.656,423.404) | -0.058(-0.274,0.158) | 2.281(2.212,2.349) |
| United Republic of Tanzania | 149(73,264) | 157(52,342) | 5.251(-61.002,128.445) | -2.288(-2.543,-2.033) | 0.225(0.017,0.433) |
| Uganda | 50(18,107) | 77(26,153) | 55.127(-26.138,264.567) | -1.915(-2.243,-1.587) | 0.876(0.796,0.957) |
| Zambia | 54(31,90) | 52(22,95) | -2.851(-55.656,106.736) | -3.108(-3.581,-2.632) | -0.028(-0.088,0.032) |
| Botswana | 1(0,2) | 1(0,1) | -25.298(-67.838,71.684) | -2.684(-2.848,-2.519) | -0.009(-0.009,-0.008) |
| Lesotho | 1(1,3) | 1(1,2) | -21.545(-65.961,84.011) | -0.832(-1.171,-0.493) | -0.011(-0.011,-0.010) |
| Namibia | 1(0,2) | 1(0,2) | -2.649(-53.418,100.436) | -1.871(-1.993,-1.748) | -0.001(-0.001,-0.000) |
| South Africa | 16(8,26) | 11(6,17) | -32.146(-58.500,10.189) | -2.774(-3.494,-2.049) | -0.173(-0.194,-0.152) |
| Eswatini | 1(0,1) | 1(0,1) | -22.657(-65.513,74.506) | -1.711(-1.823,-1.600) | -0.005(-0.006,-0.005) |
| Zimbabwe | 9(4,15) | 15(8,30) | 71.212(-16.719,234.170) | 0.880(0.425,1.336) | 0.187(0.171,0.203) |
| Benin | 21(6,53) | 37(11,81) | 73.000(-29.812,349.986) | -1.348(-1.490,-1.206) | 0.466(0.438,0.494) |
| Burkina Faso | 61(18,129) | 74(23,166) | 21.618(-44.391,161.782) | -2.381(-2.599,-2.163) | 0.404(0.378,0.429) |
| Cameroon | 34(10,72) | 64(24,123) | 85.538(-13.991,344.903) | -1.243(-1.483,-1.003) | 0.937(0.902,0.972) |
| Cabo Verde | 1(0,2) | 1(0,1) | -34.147(-66.087,69.681) | -3.251(-3.524,-2.978) | -0.008(-0.009,-0.008) |
| Chad | 35(6,90) | 72(11,193) | 109.837(-3.314,346.756) | -0.926(-1.037,-0.815) | 1.254(1.203,1.306) |
| Côte d'Ivoire | 41(10,93) | 61(18,128) | 49.947(-24.026,224.674) | -0.942(-1.210,-0.674) | 0.603(0.482,0.724) |
| Gambia | 3(1,9) | 5(2,12) | 56.332(-26.915,272.202) | -1.560(-1.806,-1.314) | 0.059(0.051,0.068) |
| Ghana | 105(48,187) | 127(56,221) | 20.313(-34.232,122.017) | -1.670(-1.959,-1.381) | 0.705(0.475,0.935) |
| Guinea | 38(12,82) | 42(11,90) | 11.844(-51.676,122.476) | -1.700(-1.901,-1.500) | 0.187(0.140,0.234) |
| Guinea-Bissau | 6(2,14) | 6(2,13) | 1.634(-51.157,101.321) | -1.784(-1.975,-1.592) | 0.004(-0.005,0.013) |
| Liberia | 10(2,20) | 11(2,25) | 10.766(-58.325,118.824) | -2.645(-2.886,-2.404) | 0.040(0.024,0.056) |
| Mali | 54(9,146) | 76(15,192) | 40.903(-32.650,175.025) | -2.040(-2.293,-1.787) | 0.737(0.654,0.819) |
| Mauritania | 7(2,13) | 6(2,11) | -15.472(-56.821,57.755) | -2.720(-2.929,-2.512) | -0.036(-0.039,-0.032) |
| Niger | 66(13,188) | 118(18,340) | 78.028(-46.876,313.339) | -1.826(-2.007,-1.644) | 1.714(1.582,1.846) |
| Nigeria | 678(363,1061) | 920(380,1577) | 35.713(-38.143,154.974) | -1.815(-2.113,-1.516) | 7.704(7.152,8.255) |
| Sao Tome and Principe | 0(0,1) | 0(0,0) | -34.306(-68.181,54.281) | -3.273(-3.459,-3.086) | -0.004(-0.005,-0.004) |
| Senegal | 41(13,86) | 35(11,75) | -14.614(-57.723,67.880) | -2.407(-2.645,-2.168) | -0.248(-0.316,-0.180) |
| Sierra Leone | 19(7,44) | 20(5,51) | 2.704(-60.627,124.374) | -2.361(-2.661,-2.059) | 0.011(-0.019,0.041) |
| Togo | 15(6,31) | 19(7,38) | 28.486(-38.450,157.880) | -1.616(-1.753,-1.478) | 0.134(0.122,0.146) |
| American Samoa | 0(0,0) | 0(0,0) | 12.760(-44.253,173.348) | 0.525(0.379,0.670) | 0.000(0.000,0.000) |
| Bermuda | 0(0,0) | 0(0,0) | 88.576(13.975,204.966) | 2.263(1.646,2.884) | 0.000(0.000,0.000) |
| Cook Islands | 0(0,0) | 0(0,0) | -21.654(-69.265,186.074) | -0.742(-1.030,-0.453) | -0.000(-0.000,-0.000) |
| Greenland | 0(0,0) | 0(0,0) | 58.080(-26.231,223.705) | 2.738(1.891,3.592) | 0.000(0.000,0.000) |
| Guam | 0(0,0) | 0(0,0) | 1.928(-38.832,67.967) | 0.464(0.105,0.824) | -0.000(-0.000,0.000) |
| Monaco | 0(0,0) | 0(0,0) | -45.567(-76.128,17.244) | -2.045(-3.132,-0.946) | -0.000(-0.000,-0.000) |
| Nauru | 0(0,0) | 0(0,0) | 33.124(-41.177,213.544) | 0.416(-0.174,1.009) | 0.000(0.000,0.000) |
| Niue | 0(0,0) | 0(0,0) | -51.277(-80.286,31.005) | -1.046(-1.233,-0.859) | -0.000(-0.000,-0.000) |
| Northern Mariana Islands | 0(0,0) | 0(0,0) | 40.412(-24.610,171.512) | 1.090(0.641,1.541) | 0.000(0.000,0.000) |
| Palau | 0(0,0) | 0(0,0) | 34.022(-46.446,248.678) | 0.137(-0.029,0.304) | 0.000(0.000,0.000) |
| Puerto Rico | 0(0,0) | 0(0,0) | 1226.234(588.910,1809.229) | 9.471(6.530,12.493) | 0.000(-0.000,0.001) |
| Saint Kitts and Nevis | 0(0,0) | 0(0,0) | -19.146(-38.253,8.333) | -1.705(-1.916,-1.494) | -0.000(-0.000,-0.000) |
| San Marino | 0(0,0) | 0(0,0) | -97.460(-99.041,-93.709) | -17.414(-19.941,-14.807) | -0.000(-0.000,-0.000) |
| Tokelau | 0(0,0) | 0(0,0) | -31.867(-70.917,85.483) | -0.793(-1.228,-0.356) | -0.000(-0.000,-0.000) |
| Tuvalu | 0(0,0) | 0(0,0) | -21.318(-70.523,127.892) | -1.714(-1.920,-1.507) | -0.000(-0.000,-0.000) |
| United States Virgin Islands | 0(0,0) | 0(0,0) | -14.540(-56.843,76.578) | 0.359(-0.034,0.753) | -0.000(-0.000,-0.000) |
| South Sudan | 17(4,39) | 30(6,73) | 78.122(-18.239,238.568) | 0.013(-0.338,0.365) | 0.438(0.389,0.487) |
| Sudan | 0(0,1) | 0(0,1) | 3.562(-51.620,137.298) | -2.296(-2.556,-2.035) | 0.000(0.000,0.001) |

Abbreviations: AAPC, average annual percent change; CI, confidence interval; EAPC, estimated annual percentage change; UI, uncertainty interval.

Supplementary Table 7: Trends in the ASPR of rabies in 204 countries and territories worldwide from 1990 to 2021.

| Location | ASPR (per 100,000 population)  (95% UI).  1990 year | ASPR (per 100,000 population)  (95% UI).  2021 year | Percentage change  (95% UI). 1990－2021. | EAPC (95% CI). 1990－2021. | AAPC (95% CI). 1990－2021. |
| --- | --- | --- | --- | --- | --- |
| China | 0.004(0.002,0.005) | 0.001(0.001,0.002) | -63.957(-75.652,-48.645) | -0.952(-3.022,1.163) | -2.103(-2.765, -1.442) |
| Democratic People's Republic of Korea | 0.002(0.001,0.004) | 0.002(0.001,0.004) | 7.444(-37.619,75.653) | 0.266(-0.163,0.697) | 0.275(0.216, 0.333) |
| Taiwan (Province of China) | 0.000(0.000,0.000) | 0.000(0.000,0.000) | -69.006(-77.333,-58.515) | -2.470(-3.988,-0.929) | -3.302(-4.066, -2.537) |
| Cambodia | 0.021(0.007,0.038) | 0.007(0.002,0.013) | -68.453(-81.786,-47.664) | -4.634(-5.099,-4.167) | -3.61(-3.843, -3.377) |
| Indonesia | 0.003(0.002,0.004) | 0.001(0.000,0.001) | -69.337(-83.784,-45.836) | -3.745(-3.974,-3.515) | -3.73(-3.827, -3.632) |
| Lao People's Democratic Republic | 0.023(0.009,0.042) | 0.007(0.003,0.012) | -69.143(-82.443,-45.047) | -4.177(-4.621,-3.731) | -3.682(-3.771, -3.593) |
| Malaysia | 0.000(0.000,0.000) | 0.000(0.000,0.000) | -64.075(-80.309,-23.408) | -3.087(-3.375,-2.798) | -3.363(-3.853, -2.873) |
| Maldives | 0.000(0.000,0.001) | 0.000(0.000,0.000) | -79.392(-89.762,-53.011) | -4.930(-5.204,-4.655) | -4.907(-5.209, -4.605) |
| Myanmar | 0.052(0.030,0.085) | 0.017(0.008,0.030) | -68.416(-84.520,-38.291) | -3.924(-4.147,-3.701) | -3.637(-3.693, -3.582) |
| Philippines | 0.033(0.025,0.041) | 0.009(0.006,0.013) | -72.017(-79.604,-64.200) | -4.976(-5.592,-4.355) | -3.899(-4.088, -3.71) |
| Sri Lanka | 0.023(0.012,0.036) | 0.003(0.001,0.006) | -85.301(-92.780,-71.983) | -8.020(-8.829,-7.204) | -5.924(-6.393, -5.456) |
| Thailand | 0.002(0.001,0.003) | 0.000(0.000,0.000) | -85.193(-91.856,-71.056) | -7.771(-11.025,-4.399) | -2.183(-3.604, -0.762) |
| Timor-Leste | 0.017(0.004,0.037) | 0.007(0.002,0.013) | -58.437(-75.513,-21.159) | -3.195(-3.426,-2.964) | -2.729(-2.842, -2.616) |
| Viet Nam | 0.006(0.003,0.011) | 0.002(0.001,0.004) | -65.722(-80.089,-35.771) | -3.465(-3.548,-3.383) | -3.402(-3.444, -3.359) |
| Fiji | 0.000(0.000,0.001) | 0.000(0.000,0.001) | 0.705(-61.146,179.229) | 0.755(0.210,1.303) | 0.12(-0.13, 0.371) |
| Kiribati | 0.003(0.002,0.006) | 0.003(0.001,0.005) | -22.086(-64.069,67.071) | -0.763(-0.826,-0.700) | -0.793(-0.842, -0.744) |
| Marshall Islands | 0.001(0.000,0.004) | 0.001(0.000,0.002) | -42.447(-74.614,43.659) | -1.934(-2.169,-1.699) | -1.833(-2.076, -1.59) |
| Micronesia (Federated States of) | 0.002(0.001,0.005) | 0.001(0.000,0.002) | -46.298(-74.761,17.676) | -1.830(-1.891,-1.770) | -2.013(-2.066, -1.96) |
| Papua New Guinea | 0.002(0.000,0.007) | 0.001(0.000,0.004) | -27.334(-71.176,94.182) | -0.913(-1.000,-0.826) | -0.981(-1.043, -0.92) |
| Samoa | 0.002(0.001,0.004) | 0.001(0.000,0.002) | -33.056(-68.107,48.478) | -1.091(-1.173,-1.008) | -1.329(-1.435, -1.223) |
| Solomon Islands | 0.002(0.000,0.006) | 0.001(0.000,0.004) | -25.693(-65.665,142.929) | -1.050(-1.195,-0.905) | -0.949(-1.006, -0.893) |
| Tonga | 0.001(0.000,0.003) | 0.001(0.000,0.002) | -37.974(-72.990,55.991) | -1.290(-1.471,-1.109) | -1.551(-1.702, -1.4) |
| Vanuatu | 0.002(0.000,0.005) | 0.001(0.000,0.003) | -34.053(-70.565,59.852) | -1.253(-1.355,-1.152) | -1.343(-1.465, -1.222) |
| Armenia | 0.000(0.000,0.000) | 0.000(0.000,0.000) | -85.653(-91.838,-75.119) | -7.569(-9.324,-5.780) | -3.436(-5.817, -1.055) |
| Azerbaijan | 0.001(0.000,0.004) | 0.001(0.000,0.001) | -63.107(-89.439,25.432) | -4.562(-5.416,-3.700) | -2.919(-3.192, -2.645) |
| Georgia | 0.001(0.000,0.001) | 0.001(0.000,0.001) | -20.486(-64.264,74.158) | -1.469(-3.690,0.803) | 2.751(-0.139, 5.641) |
| Kazakhstan | 0.000(0.000,0.001) | 0.001(0.000,0.001) | 52.852(-24.422,217.060) | 0.830(-1.133,2.831) | 3.28(1.143, 5.418) |
| Kyrgyzstan | 0.001(0.001,0.002) | 0.000(0.000,0.001) | -71.556(-89.059,-30.629) | -4.833(-6.700,-2.928) | -2.517(-3.571, -1.462) |
| Mongolia | 0.006(0.001,0.020) | 0.002(0.000,0.006) | -69.371(-94.134,66.269) | -4.358(-4.683,-4.032) | -3.437(-4.193, -2.682) |
| Tajikistan | 0.000(0.000,0.000) | 0.000(0.000,0.000) | -58.881(-86.014,37.394) | -3.465(-3.832,-3.096) | -2.845(-2.995, -2.695) |
| Turkmenistan | 0.000(0.000,0.000) | 0.000(0.000,0.000) | -73.956(-89.097,-34.611) | -4.514(-10.434,1.796) | 20.581(6.645, 34.516) |
| Uzbekistan | 0.000(0.000,0.001) | 0.000(0.000,0.000) | -55.360(-76.514,-11.956) | -3.718(-4.121,-3.313) | -2.347(-2.839, -1.856) |
| Albania | 0.000(0.000,0.000) | 0.000(0.000,0.000) | -90.110(-96.955,-67.876) | -10.153(-11.488,-8.798) | -6.566(-7.066, -6.066) |
| Bosnia and Herzegovina | 0.000(0.000,0.000) | 0.000(0.000,0.000) | -88.495(-96.850,-62.267) | -6.989(-8.141,-5.823) | -6.407(-6.645, -6.168) |
| Bulgaria | 0.000(0.000,0.000) | 0.000(0.000,0.000) | 50.303(-6.563,137.574) | 0.533(-0.774,1.857) | 2.381(1.57, 3.191) |
| Croatia | 0.000(0.000,0.000) | 0.000(0.000,0.000) | -98.666(-99.171,-97.789) | -17.526(-19.709,-15.284) | -9.988(-11.894, -8.082) |
| Czechia | 0.000(0.000,0.000) | 0.000(0.000,0.000) | -77.159(-84.578,-65.730) | -4.465(-7.111,-1.743) | -2.922(-4.58, -1.265) |
| Hungary | 0.000(0.000,0.000) | 0.000(0.000,0.000) | 6.360(-23.350,50.097) | -5.275(-8.814,-1.599) | 4.91(2.212, 7.607) |
| North Macedonia | 0.000(0.000,0.000) | 0.000(0.000,0.000) | -55.116(-81.444,19.591) | -3.718(-4.796,-2.628) | -2.402(-2.897, -1.907) |
| Montenegro | 0.000(0.000,0.000) | 0.000(0.000,0.000) | -40.632(-74.896,32.202) | -2.127(-2.435,-1.818) | -1.414(-1.684, -1.144) |
| Poland | 0.000(0.000,0.000) | 0.000(0.000,0.000) | -99.701(-99.764,-99.566) | -20.806(-22.879,-18.677) | -14.304(-15.936, -12.671) |
| Romania | 0.000(0.000,0.001) | 0.000(0.000,0.000) | -99.763(-99.833,-99.671) | -19.408(-20.514,-18.287) | -17.209(-17.727, -16.691) |
| Serbia | 0.000(0.000,0.000) | 0.000(0.000,0.000) | -85.095(-92.814,-66.111) | -8.092(-9.242,-6.928) | -5.36(-5.797, -4.924) |
| Slovakia | 0.000(0.000,0.000) | 0.000(0.000,0.000) | -62.299(-81.628,-22.381) | -3.057(-3.176,-2.938) | -2.988(-3.149, -2.828) |
| Slovenia | 0.000(0.000,0.000) | 0.000(0.000,0.000) | -41.168(-56.084,-16.517) | -6.433(-9.118,-3.669) | 3.509(0.153, 6.865) |
| Belarus | 0.000(0.000,0.000) | 0.000(0.000,0.001) | 344.095(163.014,582.028) | 4.055(2.215,5.928) | 5.945(4.371, 7.519) |
| Estonia | 0.000(0.000,0.000) | 0.000(0.000,0.000) | 11.676(-32.537,78.759) | -0.886(-4.238,2.583) | 2.874(1.074, 4.674) |
| Latvia | 0.001(0.001,0.001) | 0.000(0.000,0.000) | -87.012(-91.564,-80.255) | -6.997(-9.146,-4.798) | -2.762(-5.238, -0.285) |
| Lithuania | 0.000(0.000,0.000) | 0.000(0.000,0.000) | -99.253(-99.618,-98.592) | -15.055(-19.577,-10.279) | -3.179(-8.458, 2.1) |
| Republic of Moldova | 0.000(0.000,0.000) | 0.000(0.000,0.000) | -69.095(-81.767,-47.113) | -3.537(-3.824,-3.249) | -3.722(-3.926, -3.518) |
| Russian Federation | 0.000(0.000,0.000) | 0.000(0.000,0.000) | -79.706(-81.773,-77.398) | -3.654(-5.222,-2.060) | -4.54(-5.07, -4.01) |
| Ukraine | 0.000(0.000,0.000) | 0.000(0.000,0.000) | -9.631(-54.592,87.843) | 1.060(0.040,2.091) | 0.084(-0.93, 1.098) |
| Brunei Darussalam | 0.000(0.000,0.000) | 0.000(0.000,0.000) | -64.969(-80.816,-32.473) | -2.307(-3.099,-1.509) | -3.013(-3.325, -2.701) |
| Japan | 0.000(0.000,0.000) | 0.000(0.000,0.000) | -92.229(-93.118,-91.291) | -6.218(-7.678,-4.735) | -6.678(-7.61, -5.745) |
| Republic of Korea | 0.000(0.000,0.000) | 0.000(0.000,0.000) | -97.455(-98.878,-85.555) | -12.523(-14.103,-10.914) | -9.768(-10.545, -8.99) |
| Singapore | 0.000(0.000,0.000) | 0.000(0.000,0.000) | -60.576(-72.884,-45.753) | -4.151(-5.212,-3.078) | -2.373(-2.917, -1.829) |
| Australia | 0.000(0.000,0.000) | 0.000(0.000,0.000) | 166.054(54.942,341.385) | 11.202(7.995,14.505) | 5.686(4.443, 6.929) |
| New Zealand | 0.000(0.000,0.000) | 0.000(0.000,0.000) | -75.941(-83.354,-66.721) | -2.635(-4.038,-1.211) | -3.528(-4.254, -2.802) |
| Andorra | 0.000(0.000,0.000) | 0.000(0.000,0.000) | -53.450(-76.502,-10.040) | -2.384(-2.556,-2.212) | -2.51(-2.845, -2.174) |
| Austria | 0.000(0.000,0.000) | 0.000(0.000,0.000) | -37.150(-55.542,-7.714) | -2.238(-4.379,-0.048) | 0.872(-0.173, 1.917) |
| Belgium | 0.000(0.000,0.000) | 0.000(0.000,0.000) | -70.902(-79.580,-59.317) | 1.705(-0.763,4.234) | -2.613(-3.807, -1.42) |
| Cyprus | 0.000(0.000,0.000) | 0.000(0.000,0.000) | -86.044(-94.339,-56.160) | -5.346(-6.319,-4.363) | -5.849(-6.338, -5.361) |
| Denmark | 0.000(0.000,0.000) | 0.000(0.000,0.000) | -89.143(-92.088,-85.518) | -5.835(-7.530,-4.109) | -4.984(-6.309, -3.658) |
| Finland | 0.000(0.000,0.000) | 0.000(0.000,0.000) | -49.948(-73.772,-8.221) | -2.718(-3.004,-2.430) | -2.187(-2.71, -1.663) |
| France | 0.000(0.000,0.000) | 0.000(0.000,0.000) | -24.073(-48.635,12.810) | -2.526(-3.667,-1.371) | -0.55(-1.837, 0.736) |
| Germany | 0.000(0.000,0.000) | 0.000(0.000,0.000) | 375.817(188.297,628.813) | 6.565(5.459,7.681) | 6.141(5.109, 7.172) |
| Greece | 0.000(0.000,0.000) | 0.000(0.000,0.000) | 603.079(363.148,1024.803) | 13.444(9.179,17.876) | 18.436(13.075, 23.797) |
| Iceland | 0.000(0.000,0.000) | 0.000(0.000,0.000) | -35.281(-55.034,-8.576) | -6.764(-10.780,-2.568) | 6.171(3.292, 9.05) |
| Ireland | 0.000(0.000,0.000) | 0.000(0.000,0.000) | -37.555(-53.640,-12.580) | 0.688(-0.844,2.244) | 0.952(-0.244, 2.148) |
| Israel | 0.000(0.000,0.000) | 0.000(0.000,0.000) | -91.183(-94.213,-86.558) | -11.755(-13.907,-9.550) | -3.014(-8.074, 2.045) |
| Italy | 0.000(0.000,0.000) | 0.000(0.000,0.000) | -93.105(-94.131,-91.703) | -8.511(-8.892,-8.129) | -8.31(-8.661, -7.96) |
| Luxembourg | 0.000(0.000,0.000) | 0.000(0.000,0.000) | -56.808(-68.516,-42.293) | -2.635(-6.527,1.418) | -0.055(-2.738, 2.628) |
| Malta | 0.000(0.000,0.000) | 0.000(0.000,0.000) | -97.970(-98.487,-97.249) | -9.867(-11.969,-7.715) | -7.436(-10.706, -4.166) |
| Netherlands | 0.000(0.000,0.000) | 0.000(0.000,0.000) | 218.465(127.139,342.305) | 4.553(2.221,6.938) | 7.976(4.112, 11.84) |
| Norway | 0.000(0.000,0.000) | 0.000(0.000,0.000) | 513.422(391.626,635.339) | 4.258(2.519,6.026) | 8.59(5.849, 11.331) |
| Portugal | 0.000(0.000,0.000) | 0.000(0.000,0.000) | -95.686(-96.895,-93.757) | -8.922(-11.263,-6.520) | -3.085(-6.413, 0.244) |
| Spain | 0.000(0.000,0.000) | 0.000(0.000,0.000) | -51.215(-63.421,-34.092) | -0.988(-1.841,-0.127) | -1.876(-2.97, -0.782) |
| Sweden | 0.000(0.000,0.000) | 0.000(0.000,0.000) | 29.093(-9.962,78.394) | -0.884(-4.288,2.640) | 11.554(3.21, 19.898) |
| Switzerland | 0.000(0.000,0.000) | 0.000(0.000,0.000) | 283.205(155.462,437.371) | 3.188(-0.614,7.135) | 11.885(7.598, 16.171) |
| United Kingdom | 0.000(0.000,0.000) | 0.000(0.000,0.000) | 601.745(526.702,683.906) | 6.494(5.236,7.768) | 8.062(4.801, 11.322) |
| Argentina | 0.000(0.000,0.000) | 0.000(0.000,0.000) | -18.022(-44.680,11.939) | -0.859(-1.246,-0.472) | -0.541(-0.789, -0.294) |
| Chile | 0.000(0.000,0.000) | 0.000(0.000,0.000) | 30.109(-1.055,74.062) | 1.116(-0.977,3.254) | 3.516(0.174, 6.858) |
| Uruguay | 0.000(0.000,0.000) | 0.000(0.000,0.000) | -70.117(-79.403,-54.278) | -3.820(-4.178,-3.460) | -3.912(-4.248, -3.576) |
| Canada | 0.000(0.000,0.000) | 0.000(0.000,0.000) | 2977.565(1943.881,4571.017) | 9.484(5.843,13.252) | 16.425(10.803, 22.048) |
| United States of America | 0.000(0.000,0.000) | 0.000(0.000,0.000) | 178.772(149.346,209.925) | 2.420(2.009,2.832) | 3.403(2.976, 3.83) |
| Antigua and Barbuda | 0.000(0.000,0.000) | 0.000(0.000,0.000) | -25.672(-50.072,11.106) | -0.718(-0.936,-0.501) | -0.759(-1.078, -0.44) |
| Bahamas | 0.000(0.000,0.000) | 0.000(0.000,0.000) | -3.818(-40.382,57.779) | -0.084(-0.276,0.108) | -0.094(-0.262, 0.074) |
| Barbados | 0.000(0.000,0.000) | 0.000(0.000,0.000) | 176.031(87.215,286.083) | 3.524(2.280,4.783) | 4.408(3.569, 5.247) |
| Belize | 0.000(0.000,0.000) | 0.000(0.000,0.000) | -54.966(-69.301,-33.635) | -1.317(-2.384,-0.237) | -1.988(-2.778, -1.198) |
| Cuba | 0.000(0.000,0.000) | 0.000(0.000,0.000) | -25.268(-49.970,16.286) | 0.023(-3.037,3.180) | 3.372(-1.889, 8.632) |
| Dominica | 0.000(0.000,0.000) | 0.000(0.000,0.000) | -42.690(-66.680,-3.774) | -1.759(-2.019,-1.498) | -1.874(-2.004, -1.744) |
| Dominican Republic | 0.000(0.000,0.000) | 0.000(0.000,0.000) | -44.492(-79.013,38.307) | -1.237(-1.874,-0.595) | -1.699(-2.177, -1.221) |
| Grenada | 0.000(0.000,0.000) | 0.000(0.000,0.000) | -19.085(-43.435,17.974) | 0.325(0.034,0.617) | -0.597(-0.739, -0.455) |
| Guyana | 0.000(0.000,0.000) | 0.000(0.000,0.000) | -50.437(-73.323,-12.007) | -1.702(-2.481,-0.918) | -2.108(-2.338, -1.878) |
| Haiti | 0.000(0.000,0.001) | 0.000(0.000,0.000) | -47.881(-74.172,1.027) | -2.379(-2.486,-2.271) | -2.068(-2.13, -2.007) |
| Jamaica | 0.000(0.000,0.000) | 0.000(0.000,0.000) | 4333.011(2522.368,6453.497) | 10.853(5.024,17.005) | 27.283(20.825, 33.74) |
| Saint Lucia | 0.000(0.000,0.000) | 0.000(0.000,0.000) | 11.058(-45.974,293.590) | -2.807(-7.391,2.004) | 13.041(3.17, 22.911) |
| Saint Vincent and the Grenadines | 0.001(0.001,0.002) | 0.000(0.000,0.000) | -96.370(-97.684,-93.122) | 5.038(-2.663,13.348) | 10.329(-4.982, 25.641) |
| Suriname | 0.000(0.000,0.000) | 0.000(0.000,0.000) | 469.423(143.538,1232.285) | 2.298(-0.887,5.585) | 8.044(7.06, 9.028) |
| Trinidad and Tobago | 0.000(0.000,0.000) | 0.000(0.000,0.000) | 2923.275(1711.384,4986.896) | 8.341(4.707,12.102) | 12.745(11.499, 13.991) |
| Bolivia (Plurinational State of) | 0.001(0.000,0.001) | 0.000(0.000,0.000) | -97.470(-98.938,-94.078) | -11.942(-12.858,-11.018) | -11.041(-11.27, -10.812) |
| Ecuador | 0.006(0.005,0.007) | 0.000(0.000,0.000) | -99.983(-99.989,-99.974) | -28.339(-31.447,-25.091) | -13.791(-26.12, -1.463) |
| Peru | 0.000(0.000,0.001) | 0.000(0.000,0.000) | -98.759(-99.640,-90.940) | -12.164(-13.890,-10.404) | -12.164(-12.678, -11.65) |
| Colombia | 0.000(0.000,0.000) | 0.000(0.000,0.000) | -99.680(-99.769,-99.547) | -16.082(-19.058,-12.997) | -9.365(-18.541, -0.188) |
| Costa Rica | 0.000(0.000,0.000) | 0.000(0.000,0.000) | -44.191(-81.857,66.869) | -1.485(-1.984,-0.984) | -1.82(-2.463, -1.178) |
| El Salvador | 0.001(0.000,0.002) | 0.000(0.000,0.000) | -99.725(-99.873,-97.312) | -20.711(-22.859,-18.503) | -16.03(-16.701, -15.359) |
| Guatemala | 0.004(0.003,0.004) | 0.000(0.000,0.000) | -99.842(-99.893,-99.789) | -20.122(-23.113,-17.016) | -12.106(-17.858, -6.354) |
| Honduras | 0.000(0.000,0.000) | 0.000(0.000,0.000) | -69.489(-82.846,-48.548) | -4.053(-4.228,-3.879) | -3.732(-3.814, -3.651) |
| Mexico | 0.003(0.002,0.003) | 0.000(0.000,0.000) | -99.931(-99.944,-99.918) | -19.966(-20.728,-19.197) | -21.5(-22.485, -20.516) |
| Nicaragua | 0.000(0.000,0.000) | 0.000(0.000,0.000) | -87.795(-94.050,-75.753) | -8.484(-9.940,-7.003) | -5.958(-7.393, -4.523) |
| Panama | 0.000(0.000,0.000) | 0.000(0.000,0.000) | -83.331(-89.365,-71.292) | -4.659(-5.045,-4.271) | -5.546(-5.627, -5.465) |
| Venezuela (Bolivarian Republic of) | 0.000(0.000,0.000) | 0.000(0.000,0.000) | -97.906(-98.611,-96.919) | -13.663(-15.885,-11.383) | -9.066(-9.793, -8.339) |
| Brazil | 0.002(0.001,0.003) | 0.000(0.000,0.000) | -99.645(-99.745,-99.493) | -17.286(-18.450,-16.105) | -15.738(-16.807, -14.669) |
| Paraguay | 0.000(0.000,0.000) | 0.000(0.000,0.000) | -99.410(-99.763,-90.775) | -21.794(-24.598,-18.886) | -12.276(-13.554, -10.998) |
| Algeria | 0.000(0.000,0.000) | 0.000(0.000,0.000) | -44.499(-68.237,31.761) | -1.660(-1.786,-1.533) | -1.913(-1.988, -1.838) |
| Bahrain | 0.000(0.000,0.000) | 0.000(0.000,0.000) | -66.618(-79.714,-38.889) | -3.903(-4.135,-3.671) | -3.478(-3.549, -3.406) |
| Egypt | 0.006(0.003,0.008) | 0.000(0.000,0.001) | -94.339(-96.509,-89.949) | -8.852(-9.407,-8.294) | -8.912(-9.38, -8.444) |
| Iran (Islamic Republic of) | 0.000(0.000,0.000) | 0.000(0.000,0.000) | -53.067(-73.902,-6.542) | -0.330(-1.236,0.584) | -2.316(-2.844, -1.788) |
| Iraq | 0.001(0.000,0.001) | 0.000(0.000,0.000) | -66.577(-85.950,-24.176) | -2.361(-2.981,-1.736) | -3.398(-3.564, -3.232) |
| Jordan | 0.000(0.000,0.000) | 0.000(0.000,0.000) | -49.912(-71.832,-7.716) | -1.935(-2.389,-1.480) | -2.207(-2.542, -1.872) |
| Kuwait | 0.000(0.000,0.000) | 0.000(0.000,0.000) | 36.022(-48.523,229.612) | -5.061(-6.992,-3.090) | 6.664(2.433, 10.895) |
| Lebanon | 0.000(0.000,0.000) | 0.000(0.000,0.000) | -56.909(-77.322,-11.297) | -2.292(-2.511,-2.072) | -2.686(-2.767, -2.605) |
| Libya | 0.000(0.000,0.000) | 0.000(0.000,0.000) | -7.724(-58.823,120.570) | 0.172(-0.230,0.575) | -0.173(-0.284, -0.062) |
| Morocco | 0.000(0.000,0.000) | 0.000(0.000,0.000) | -51.559(-75.167,7.817) | -1.890(-2.108,-1.671) | -2.296(-2.363, -2.229) |
| Palestine | 0.000(0.000,0.000) | 0.000(0.000,0.000) | -69.308(-86.764,-3.647) | -3.381(-3.675,-3.086) | -3.741(-3.812, -3.67) |
| Oman | 0.000(0.000,0.000) | 0.000(0.000,0.000) | -66.679(-87.712,19.004) | -3.079(-3.435,-2.722) | -3.296(-3.651, -2.941) |
| Qatar | 0.000(0.000,0.000) | 0.000(0.000,0.000) | -48.219(-75.021,30.134) | -3.103(-5.025,-1.141) | 0.697(-1.363, 2.758) |
| Saudi Arabia | 0.000(0.000,0.000) | 0.000(0.000,0.000) | -75.517(-88.903,-39.899) | -5.354(-6.168,-4.533) | -3.76(-4.452, -3.068) |
| Syrian Arab Republic | 0.000(0.000,0.000) | 0.000(0.000,0.000) | 12.993(-61.660,249.759) | 1.570(0.670,2.478) | 0.654(0.397, 0.91) |
| Tunisia | 0.000(0.000,0.000) | 0.000(0.000,0.000) | -49.911(-75.102,30.742) | -2.103(-2.170,-2.036) | -2.223(-2.274, -2.173) |
| Türkiye | 0.000(0.000,0.001) | 0.000(0.000,0.000) | -82.236(-90.355,-63.895) | -5.554(-5.766,-5.342) | -5.401(-5.499, -5.303) |
| United Arab Emirates | 0.002(0.000,0.004) | 0.001(0.000,0.002) | -58.900(-85.207,42.435) | -1.748(-2.126,-1.368) | -2.77(-3.582, -1.958) |
| Yemen | 0.000(0.000,0.000) | 0.000(0.000,0.000) | -19.139(-65.243,150.488) | -0.788(-1.001,-0.575) | -0.614(-0.743, -0.484) |
| Afghanistan | 0.000(0.000,0.000) | 0.000(0.000,0.000) | -21.672(-57.176,117.264) | -1.126(-1.713,-0.536) | -0.785(-0.898, -0.671) |
| Bangladesh | 0.012(0.004,0.021) | 0.001(0.000,0.002) | -92.675(-96.396,-83.120) | -10.067(-10.976,-9.149) | -7.908(-8.473, -7.343) |
| Bhutan | 0.051(0.011,0.104) | 0.008(0.003,0.014) | -84.990(-91.457,-66.881) | -6.631(-6.987,-6.274) | -5.909(-6.041, -5.776) |
| India | 0.064(0.047,0.078) | 0.012(0.007,0.017) | -81.534(-85.947,-75.293) | -5.938(-6.183,-5.693) | -5.297(-5.424, -5.17) |
| Nepal | 0.240(0.116,0.412) | 0.066(0.035,0.102) | -72.562(-84.421,-52.455) | -4.389(-4.500,-4.278) | -4.083(-4.133, -4.033) |
| Pakistan | 0.053(0.027,0.092) | 0.012(0.007,0.018) | -77.363(-85.677,-61.120) | -5.293(-5.543,-5.042) | -4.678(-4.784, -4.571) |
| Angola | 0.002(0.000,0.004) | 0.001(0.000,0.002) | -64.150(-82.191,-33.731) | -3.297(-3.522,-3.072) | -3.254(-3.344, -3.164) |
| Central African Republic | 0.002(0.000,0.005) | 0.001(0.000,0.003) | -26.806(-58.666,42.942) | -1.063(-1.185,-0.940) | -0.995(-1.043, -0.947) |
| Congo | 0.001(0.000,0.002) | 0.000(0.000,0.001) | -59.960(-79.519,-33.267) | -2.889(-2.969,-2.808) | -2.756(-2.935, -2.577) |
| Democratic Republic of the Congo | 0.001(0.000,0.003) | 0.001(0.000,0.002) | -31.354(-60.779,19.981) | -1.047(-1.270,-0.823) | -1.219(-1.344, -1.094) |
| Equatorial Guinea | 0.001(0.000,0.004) | 0.000(0.000,0.001) | -73.137(-89.482,38.612) | -5.010(-5.276,-4.742) | -4.117(-4.249, -3.985) |
| Gabon | 0.001(0.000,0.002) | 0.000(0.000,0.001) | -65.096(-83.534,-32.401) | -3.184(-3.238,-3.130) | -3.334(-3.388, -3.279) |
| Burundi | 0.021(0.010,0.041) | 0.013(0.003,0.039) | -39.523(-79.693,30.661) | -1.766(-2.026,-1.505) | -1.604(-1.662, -1.546) |
| Comoros | 0.015(0.006,0.034) | 0.008(0.002,0.019) | -48.989(-74.687,0.911) | -2.221(-2.549,-1.891) | -2.171(-2.459, -1.883) |
| Djibouti | 0.008(0.002,0.021) | 0.006(0.002,0.015) | -25.696(-58.388,59.359) | -1.036(-1.455,-0.615) | -0.957(-1.044, -0.87) |
| Eritrea | 0.022(0.012,0.040) | 0.012(0.005,0.022) | -47.068(-71.333,-9.492) | -1.816(-1.953,-1.679) | -2.015(-2.074, -1.957) |
| Ethiopia | 0.146(0.042,0.377) | 0.040(0.012,0.116) | -72.328(-85.357,-52.736) | -4.513(-4.794,-4.230) | -4.101(-4.262, -3.94) |
| Kenya | 0.014(0.006,0.027) | 0.008(0.003,0.014) | -46.070(-67.085,-10.152) | -1.344(-1.771,-0.915) | -1.935(-2.079, -1.792) |
| Madagascar | 0.010(0.005,0.018) | 0.008(0.003,0.014) | -24.860(-57.833,26.228) | -0.748(-1.083,-0.411) | -0.901(-0.959, -0.843) |
| Malawi | 0.053(0.027,0.091) | 0.030(0.014,0.053) | -44.694(-74.669,43.760) | -2.045(-2.308,-1.782) | -1.834(-1.922, -1.747) |
| Mauritius | 0.001(0.001,0.001) | 0.000(0.000,0.000) | -99.775(-99.837,-99.670) | -11.784(-14.777,-8.685) | -16.027(-18.567, -13.487) |
| Mozambique | 0.032(0.017,0.050) | 0.017(0.007,0.031) | -47.550(-71.461,-9.038) | -1.610(-1.795,-1.424) | -2.039(-2.095, -1.983) |
| Rwanda | 0.024(0.010,0.057) | 0.009(0.001,0.030) | -64.050(-88.158,-20.948) | -4.054(-4.471,-3.635) | -3.248(-3.394, -3.101) |
| Seychelles | 0.000(0.000,0.000) | 0.000(0.000,0.000) | 114.385(13.553,263.442) | 2.120(0.356,3.916) | 3.308(2.348, 4.267) |
| Somalia | 0.022(0.004,0.058) | 0.024(0.005,0.072) | 8.859(-47.686,94.173) | 0.286(0.082,0.490) | 0.273(0.205, 0.341) |
| United Republic of Tanzania | 0.021(0.011,0.035) | 0.010(0.004,0.021) | -51.844(-80.116,-0.271) | -2.307(-2.559,-2.055) | -2.331(-2.454, -2.209) |
| Uganda | 0.012(0.004,0.027) | 0.007(0.003,0.015) | -37.883(-69.673,46.945) | -2.061(-2.447,-1.673) | -1.565(-1.757, -1.373) |
| Zambia | 0.027(0.016,0.044) | 0.011(0.005,0.020) | -58.447(-79.007,-16.208) | -3.022(-3.498,-2.544) | -2.747(-2.889, -2.605) |
| Botswana | 0.003(0.001,0.005) | 0.001(0.001,0.002) | -53.476(-79.574,1.438) | -2.226(-2.427,-2.025) | -2.406(-2.509, -2.302) |
| Lesotho | 0.003(0.001,0.007) | 0.002(0.001,0.004) | -27.960(-66.772,58.010) | -0.436(-0.797,-0.074) | -0.979(-1.13, -0.828) |
| Namibia | 0.002(0.001,0.004) | 0.001(0.001,0.003) | -41.551(-68.835,10.770) | -1.705(-1.853,-1.556) | -1.693(-1.792, -1.594) |
| South Africa | 0.001(0.001,0.002) | 0.001(0.000,0.001) | -48.922(-67.392,-17.045) | -2.205(-2.954,-1.450) | -2.065(-2.37, -1.759) |
| Eswatini | 0.003(0.001,0.006) | 0.002(0.001,0.003) | -40.433(-70.500,27.862) | -1.336(-1.510,-1.161) | -1.596(-1.677, -1.516) |
| Zimbabwe | 0.005(0.002,0.008) | 0.005(0.002,0.008) | -1.911(-50.912,99.864) | 0.369(-0.092,0.831) | -0.056(-0.234, 0.122) |
| Benin | 0.016(0.004,0.037) | 0.010(0.003,0.022) | -34.563(-67.706,52.132) | -1.263(-1.383,-1.143) | -1.405(-1.583, -1.226) |
| Burkina Faso | 0.022(0.006,0.052) | 0.012(0.004,0.026) | -46.533(-72.183,7.575) | -2.302(-2.503,-2.100) | -1.986(-2.064, -1.908) |
| Cameroon | 0.013(0.004,0.025) | 0.008(0.003,0.014) | -36.951(-66.257,33.870) | -1.196(-1.441,-0.951) | -1.473(-1.519, -1.427) |
| Cabo Verde | 0.009(0.002,0.022) | 0.004(0.001,0.007) | -59.882(-80.190,-4.271) | -3.298(-3.546,-3.049) | -2.834(-3.002, -2.665) |
| Chad | 0.021(0.003,0.055) | 0.015(0.002,0.038) | -28.786(-61.494,36.464) | -0.949(-1.051,-0.847) | -1.014(-1.173, -0.856) |
| Côte d'Ivoire | 0.013(0.003,0.027) | 0.009(0.003,0.017) | -33.562(-62.305,22.788) | -0.975(-1.194,-0.756) | -1.381(-1.691, -1.07) |
| Gambia | 0.013(0.003,0.032) | 0.009(0.003,0.020) | -31.646(-64.469,56.700) | -1.436(-1.667,-1.204) | -1.218(-1.434, -1.001) |
| Ghana | 0.029(0.013,0.050) | 0.016(0.007,0.027) | -45.120(-69.770,-2.235) | -1.498(-1.776,-1.218) | -1.888(-1.973, -1.802) |
| Guinea | 0.022(0.006,0.047) | 0.012(0.003,0.025) | -43.662(-70.838,2.559) | -1.398(-1.573,-1.224) | -1.813(-1.87, -1.755) |
| Guinea-Bissau | 0.022(0.006,0.050) | 0.012(0.003,0.026) | -45.096(-68.782,-1.193) | -1.507(-1.642,-1.372) | -1.904(-1.975, -1.833) |
| Liberia | 0.014(0.003,0.031) | 0.008(0.001,0.018) | -44.681(-74.229,-2.656) | -2.028(-2.185,-1.870) | -1.885(-1.967, -1.803) |
| Mali | 0.024(0.004,0.065) | 0.013(0.002,0.031) | -46.369(-69.714,-3.536) | -1.944(-2.130,-1.757) | -1.959(-2.023, -1.895) |
| Mauritania | 0.013(0.004,0.025) | 0.005(0.002,0.010) | -60.095(-77.660,-27.669) | -2.668(-2.852,-2.483) | -2.93(-3.021, -2.84) |
| Niger | 0.028(0.005,0.078) | 0.018(0.003,0.049) | -34.095(-73.843,25.579) | -1.458(-1.600,-1.316) | -1.327(-1.5, -1.153) |
| Nigeria | 0.027(0.014,0.045) | 0.014(0.006,0.025) | -48.851(-73.578,-7.267) | -2.058(-2.311,-1.805) | -2.147(-2.232, -2.061) |
| Sao Tome and Principe | 0.010(0.003,0.023) | 0.005(0.002,0.009) | -56.022(-74.261,-3.563) | -2.678(-2.881,-2.474) | -2.54(-2.682, -2.397) |
| Senegal | 0.019(0.006,0.041) | 0.009(0.003,0.018) | -54.098(-74.187,-17.739) | -2.051(-2.272,-1.829) | -2.47(-2.542, -2.397) |
| Sierra Leone | 0.017(0.006,0.037) | 0.009(0.002,0.021) | -47.199(-75.293,0.074) | -1.982(-2.295,-1.669) | -2.034(-2.118, -1.95) |
| Togo | 0.015(0.006,0.032) | 0.009(0.003,0.019) | -39.855(-67.674,4.736) | -1.424(-1.571,-1.277) | -1.625(-1.679, -1.572) |
| American Samoa | 0.001(0.000,0.002) | 0.001(0.000,0.001) | -46.237(-76.434,14.105) | -1.903(-2.000,-1.806) | -1.937(-2.073, -1.802) |
| Bermuda | 0.000(0.000,0.000) | 0.000(0.000,0.000) | -34.782(-60.541,4.536) | -1.001(-1.394,-0.606) | -1.284(-1.531, -1.037) |
| Cook Islands | 0.002(0.000,0.005) | 0.001(0.000,0.001) | -63.017(-84.270,35.574) | -3.422(-3.684,-3.160) | -3.097(-3.224, -2.97) |
| Greenland | 0.000(0.000,0.000) | 0.000(0.000,0.000) | 44.346(-28.442,171.266) | 3.044(2.133,3.963) | 1.451(0.73, 2.172) |
| Guam | 0.000(0.000,0.001) | 0.000(0.000,0.000) | -55.812(-73.842,-27.991) | -1.582(-1.939,-1.223) | -2.572(-2.794, -2.35) |
| Monaco | 0.000(0.000,0.000) | 0.000(0.000,0.000) | -53.041(-76.957,-8.982) | -2.093(-2.954,-1.225) | -2.196(-2.553, -1.839) |
| Nauru | 0.001(0.000,0.003) | 0.001(0.000,0.002) | -18.089(-62.358,88.822) | -0.668(-1.049,-0.286) | -0.619(-0.709, -0.53) |
| Niue | 0.001(0.000,0.002) | 0.000(0.000,0.001) | -34.414(-73.649,73.144) | -1.695(-1.841,-1.549) | -1.313(-1.478, -1.148) |
| Northern Mariana Islands | 0.001(0.001,0.003) | 0.001(0.001,0.002) | -26.219(-56.227,33.748) | -0.857(-1.172,-0.541) | -1.383(-1.869, -0.897) |
| Palau | 0.000(0.000,0.000) | 0.000(0.000,0.000) | -24.304(-70.059,113.513) | -1.084(-1.214,-0.954) | -0.896(-1.038, -0.754) |
| Puerto Rico | 0.000(0.000,0.000) | 0.000(0.000,0.000) | 506.478(204.086,769.446) | 6.756(4.028,9.556) | 9.975(7.627, 12.323) |
| Saint Kitts and Nevis | 0.001(0.001,0.001) | 0.001(0.000,0.001) | -31.744(-47.843,-11.037) | -0.976(-1.064,-0.888) | -1.229(-1.272, -1.186) |
| San Marino | 0.001(0.000,0.001) | 0.000(0.000,0.000) | -99.099(-99.674,-97.782) | -18.925(-21.105,-16.684) | -12.333(-13.742, -10.924) |
| Tokelau | 0.001(0.000,0.002) | 0.000(0.000,0.001) | -45.515(-75.135,44.076) | -2.342(-2.512,-2.172) | -1.871(-2.112, -1.63) |
| Tuvalu | 0.003(0.001,0.007) | 0.001(0.000,0.002) | -60.147(-84.528,9.795) | -2.605(-2.742,-2.468) | -2.923(-2.967, -2.88) |
| United States Virgin Islands | 0.000(0.000,0.000) | 0.000(0.000,0.000) | -48.693(-77.628,-1.615) | -1.598(-2.048,-1.145) | -2.03(-2.284, -1.776) |
| South Sudan | 0.012(0.002,0.028) | 0.012(0.002,0.029) | 4.085(-43.874,82.123) | -0.154(-0.422,0.115) | 0.149(-0.032, 0.33) |
| Sudan | 0.000(0.000,0.000) | 0.000(0.000,0.000) | -52.906(-77.636,11.003) | -2.370(-2.622,-2.117) | -2.38(-2.453, -2.307) |

Abbreviations: ASPR, Age standardized prevalence rate; AAPC, average annual percent change; CI, confidence interval; EAPC, estimated annual percentage change; UI, uncertainty interval.

Supplementary Table 8: Trends in the number of rabies prevalent cases in 204 countries and territories worldwide from 1990 to 2021.

| Location | Rabies. Prevalence cases (persons) . (95% UI).  1990 year. | Rabies. Prevalence cases (persons) . (95% UI).  2021 year. | Rabies. Percentage change (95% UI). 1990－2021. | Rabies. EAPC (95% CI). 1990－2021. | Rabies. AAPC (95% CI). 1990－2021. |
| --- | --- | --- | --- | --- | --- |
| China | 42(25,58) | 23(12,36) | -44.178(-62.406,-19.518) | -0.147(-2.103,1.848) | -0.796(-1.438, -0.154) |
| Democratic People's Republic of Korea | 0(0,1) | 1(0,1) | 57.953(-5.551,165.980) | 0.587(0.186,0.990) | 1.535(1.462, 1.608) |
| Taiwan (Province of China) | 0(0,0) | 0(0,0) | 17.184(-21.761,64.663) | 1.464(-0.317,3.277) | 0.945(-0.07, 1.96) |
| Cambodia | 2(1,3) | 1(0,2) | -48.592(-74.062,-13.068) | -4.792(-5.274,-4.307) | -2.043(-2.237, -1.848) |
| Indonesia | 5(3,8) | 2(1,3) | -63.405(-82.642,-28.541) | -4.405(-4.613,-4.196) | -3.185(-3.273, -3.097) |
| Lao People's Democratic Republic | 1(0,2) | 0(0,1) | -48.040(-74.425,1.931) | -4.378(-4.824,-3.930) | -2.047(-2.166, -1.929) |
| Malaysia | 0(0,0) | 0(0,0) | -46.253(-71.117,14.677) | -3.817(-4.106,-3.527) | -2.108(-2.578, -1.638) |
| Maldives | 0(0,0) | 0(0,0) | -45.061(-76.306,27.865) | -4.334(-4.661,-4.006) | -1.799(-2.217, -1.381) |
| Myanmar | 21(12,35) | 9(4,17) | -55.674(-80.752,-10.250) | -3.914(-4.124,-3.704) | -2.587(-2.656, -2.519) |
| Philippines | 20(15,24) | 10(6,14) | -50.566(-65.018,-35.003) | -5.020(-5.626,-4.410) | -2.127(-2.352, -1.901) |
| Sri Lanka | 3(2,5) | 1(0,1) | -75.550(-88.474,-52.539) | -7.361(-8.201,-6.514) | -4.34(-4.772, -3.907) |
| Thailand | 1(0,1) | 0(0,0) | -76.125(-87.220,-53.580) | -6.549(-9.893,-3.082) | -0.314(-1.559, 0.931) |
| Timor-Leste | 0(0,0) | 0(0,0) | -28.250(-62.964,40.849) | -3.354(-3.607,-3.100) | -1.02(-1.128, -0.912) |
| Viet Nam | 4(2,6) | 2(1,4) | -44.390(-67.949,8.886) | -3.247(-3.332,-3.161) | -1.882(-1.936, -1.829) |
| Fiji | 0(0,0) | 0(0,0) | 120.100(-17.144,482.904) | 2.857(2.269,3.449) | 2.728(2.304, 3.151) |
| Kiribati | 0(0,0) | 0(0,0) | 40.379(-41.621,245.299) | -0.586(-0.667,-0.504) | 1.112(1.071, 1.153) |
| Marshall Islands | 0(0,0) | 0(0,0) | -8.095(-61.340,121.359) | -1.214(-1.346,-1.081) | -0.453(-0.702, -0.204) |
| Micronesia (Federated States of) | 0(0,0) | 0(0,0) | -39.105(-72.925,35.471) | -1.392(-1.491,-1.293) | -1.598(-1.697, -1.5) |
| Papua New Guinea | 0(0,0) | 0(0,0) | 159.969(9.540,533.278) | -0.145(-0.292,0.003) | 3.211(3.065, 3.356) |
| Samoa | 0(0,0) | 0(0,0) | 27.808(-38.826,186.548) | 0.276(0.077,0.475) | 0.762(0.622, 0.903) |
| Solomon Islands | 0(0,0) | 0(0,0) | 136.623(6.363,697.345) | 0.645(0.380,0.910) | 2.841(2.735, 2.948) |
| Tonga | 0(0,0) | 0(0,0) | 15.040(-50.154,187.938) | 0.378(0.187,0.570) | 0.435(0.259, 0.611) |
| Vanuatu | 0(0,0) | 0(0,0) | 116.289(-1.537,434.458) | 0.107(-0.016,0.230) | 2.484(2.344, 2.625) |
| Armenia | 0(0,0) | 0(0,0) | -79.659(-88.341,-63.440) | -5.457(-7.193,-3.688) | -2.667(-4.531, -0.802) |
| Azerbaijan | 0(0,0) | 0(0,0) | -45.241(-84.730,101.365) | -4.709(-5.710,-3.697) | -1.65(-2.039, -1.261) |
| Georgia | 0(0,0) | 0(0,0) | -41.429(-73.498,29.408) | -1.292(-3.523,0.991) | 1.665(-1.189, 4.52) |
| Kazakhstan | 0(0,0) | 0(0,0) | 78.941(-13.113,273.112) | 0.720(-1.255,2.733) | 3.868(1.698, 6.038) |
| Kyrgyzstan | 0(0,0) | 0(0,0) | -62.418(-86.030,-8.261) | -5.532(-7.369,-3.660) | -1.705(-2.682, -0.729) |
| Mongolia | 0(0,1) | 0(0,0) | -64.766(-93.248,86.980) | -5.888(-6.328,-5.446) | -3.01(-3.548, -2.472) |
| Tajikistan | 0(0,0) | 0(0,0) | -24.045(-80.588,184.836) | -3.849(-4.174,-3.522) | -0.836(-1.115, -0.556) |
| Turkmenistan | 0(0,0) | 0(0,0) | -58.854(-83.048,2.812) | -4.094(-10.406,2.662) | 25.365(8.567, 42.163) |
| Uzbekistan | 0(0,0) | 0(0,0) | -2.666(-50.224,90.617) | -3.084(-3.580,-2.586) | 0.213(-0.355, 0.781) |
| Albania | 0(0,0) | 0(0,0) | -82.280(-94.248,-38.316) | -7.608(-8.987,-6.208) | -4.783(-5.271, -4.294) |
| Bosnia and Herzegovina | 0(0,0) | 0(0,0) | -79.247(-94.762,-30.735) | -3.432(-4.293,-2.564) | -4.642(-5.108, -4.176) |
| Bulgaria | 0(0,0) | 0(0,0) | 272.760(130.852,483.105) | 5.233(4.383,6.089) | 4.963(3.94, 5.986) |
| Croatia | 0(0,0) | 0(0,0) | -96.247(-97.807,-93.574) | -14.090(-16.135,-11.995) | -7.137(-8.65, -5.625) |
| Czechia | 0(0,0) | 0(0,0) | -55.960(-71.361,-30.935) | -2.787(-5.174,-0.339) | -1(-2.607, 0.607) |
| Hungary | 0(0,0) | 0(0,0) | 69.807(19.978,147.579) | -2.968(-6.284,0.465) | 5.315(3.065, 7.564) |
| North Macedonia | 0(0,0) | 0(0,0) | -42.897(-77.788,41.006) | -3.908(-4.945,-2.860) | -1.339(-1.769, -0.909) |
| Montenegro | 0(0,0) | 0(0,0) | -19.907(-69.077,80.675) | -0.995(-1.278,-0.712) | -0.498(-0.794, -0.201) |
| Poland | 0(0,0) | 0(0,0) | -99.096(-99.277,-98.685) | -17.906(-20.031,-15.724) | -11.252(-12.438, -10.066) |
| Romania | 0(0,0) | 0(0,0) | -99.736(-99.814,-99.630) | -18.602(-19.720,-17.469) | -16.946(-17.416, -16.476) |
| Serbia | 0(0,0) | 0(0,0) | -64.398(-83.253,-17.730) | -3.540(-4.115,-2.962) | -2.993(-3.471, -2.515) |
| Slovakia | 0(0,0) | 0(0,0) | -61.757(-80.470,-16.115) | -2.972(-3.054,-2.889) | -3.065(-3.17, -2.96) |
| Slovenia | 0(0,0) | 0(0,0) | 64.645(20.614,135.188) | -3.219(-5.965,-0.394) | 7.126(3.578, 10.673) |
| Belarus | 0(0,0) | 0(0,0) | 505.457(259.550,832.995) | 5.408(3.274,7.585) | 7.631(5.619, 9.642) |
| Estonia | 0(0,0) | 0(0,0) | 208.740(90.485,387.033) | 3.382(-0.160,7.050) | 6.264(4.505, 8.024) |
| Latvia | 0(0,0) | 0(0,0) | -80.038(-87.184,-68.976) | -3.960(-6.395,-1.463) | -2.086(-4.081, -0.091) |
| Lithuania | 0(0,0) | 0(0,0) | -98.569(-99.259,-97.322) | -12.516(-16.828,-7.980) | -2.444(-8.61, 3.722) |
| Republic of Moldova | 0(0,0) | 0(0,0) | -84.090(-90.642,-73.273) | -4.817(-5.185,-4.447) | -5.702(-5.953, -5.451) |
| Russian Federation | 0(0,0) | 0(0,0) | -77.339(-79.705,-74.683) | -3.268(-4.841,-1.668) | -4.21(-4.667, -3.753) |
| Ukraine | 0(0,0) | 0(0,0) | -13.084(-58.804,89.608) | 1.412(0.407,2.427) | -0.023(-1.01, 0.963) |
| Brunei Darussalam | 0(0,0) | 0(0,0) | -9.731(-45.639,52.834) | -1.057(-1.711,-0.398) | -0.279(-0.805, 0.247) |
| Japan | 0(0,0) | 0(0,0) | -59.071(-67.482,-52.888) | -1.092(-2.540,0.378) | -1.763(-2.778, -0.749) |
| Republic of Korea | 0(0,0) | 0(0,0) | -93.138(-96.851,-66.373) | -10.151(-11.812,-8.458) | -6.702(-7.713, -5.692) |
| Singapore | 0(0,0) | 0(0,0) | 118.129(38.050,205.469) | -0.759(-1.789,0.281) | 3.178(2.622, 3.733) |
| Australia | 0(0,0) | 0(0,0) | 271.750(125.258,494.334) | 10.160(7.329,13.065) | 6.341(5.253, 7.429) |
| New Zealand | 0(0,0) | 0(0,0) | -36.002(-56.823,-5.309) | -0.610(-1.806,0.602) | -0.657(-1.423, 0.108) |
| Andorra | 0(0,0) | 0(0,0) | 56.699(-22.705,204.618) | -0.102(-0.472,0.269) | 1.543(1.006, 2.08) |
| Austria | 0(0,0) | 0(0,0) | 24.792(-9.507,67.371) | -0.061(-1.749,1.656) | 2.634(1.326, 3.942) |
| Belgium | 0(0,0) | 0(0,0) | -40.290(-58.184,-16.468) | 2.314(0.373,4.294) | -0.58(-1.146, -0.014) |
| Cyprus | 0(0,0) | 0(0,0) | -32.991(-71.261,83.122) | -2.020(-3.128,-0.900) | -0.86(-1.264, -0.457) |
| Denmark | 0(0,0) | 0(0,0) | -77.969(-83.401,-70.753) | -4.635(-6.119,-3.128) | -3.195(-4.494, -1.895) |
| Finland | 0(0,0) | 0(0,0) | -16.622(-50.389,30.270) | -1.370(-1.703,-1.037) | -0.445(-1.146, 0.255) |
| France | 0(0,0) | 0(0,0) | 50.182(-0.679,123.951) | -0.546(-1.734,0.657) | 2.112(0.43, 3.794) |
| Germany | 0(0,0) | 0(0,0) | 148.883(56.976,264.915) | 4.042(2.873,5.225) | 3.486(2.579, 4.392) |
| Greece | 0(0,0) | 0(0,0) | 3296.752(2153.102,5193.407) | 18.684(14.509,23.012) | 22.31(17.319, 27.301) |
| Iceland | 0(0,0) | 0(0,0) | 56.675(7.326,122.824) | -5.277(-9.345,-1.027) | 9.08(6.045, 12.115) |
| Ireland | 0(0,0) | 0(0,0) | 19.370(-14.635,72.019) | 1.392(-0.070,2.876) | 2.8(1.763, 3.837) |
| Israel | 0(0,0) | 0(0,0) | -78.311(-85.759,-66.868) | -11.412(-13.541,-9.232) | 2.092(-5.083, 9.267) |
| Italy | 0(0,0) | 0(0,0) | -76.078(-79.907,-71.448) | -4.881(-5.076,-4.687) | -4.443(-5, -3.885) |
| Luxembourg | 0(0,0) | 0(0,0) | 5.743(-23.877,42.154) | -1.270(-5.201,2.824) | 2.768(-2.004, 7.54) |
| Malta | 0(0,0) | 0(0,0) | -95.084(-96.325,-93.430) | -7.700(-9.739,-5.614) | -4.895(-7.744, -2.046) |
| Netherlands | 0(0,0) | 0(0,0) | 353.356(217.689,521.129) | 4.805(2.665,6.990) | 8.301(5.065, 11.537) |
| Norway | 0(0,0) | 0(0,0) | 941.881(746.784,1138.043) | 5.217(3.458,7.007) | 10.005(6.91, 13.101) |
| Portugal | 0(0,0) | 0(0,0) | -80.738(-86.697,-70.785) | -3.835(-6.575,-1.015) | 2.955(-3.811, 9.722) |
| Spain | 0(0,0) | 0(0,0) | 15.230(-16.618,56.853) | 1.737(0.702,2.783) | 0.919(-0.586, 2.425) |
| Sweden | 0(0,0) | 0(0,0) | 192.528(105.155,298.599) | 1.566(-1.571,4.803) | 13.057(5.798, 20.316) |
| Switzerland | 0(0,0) | 0(0,0) | 918.860(560.754,1403.207) | 5.659(1.652,9.823) | 15.767(10.743, 20.792) |
| United Kingdom | 0(0,0) | 0(0,0) | 735.634(665.779,810.504) | 6.556(5.363,7.762) | 8.164(6.792, 9.536) |
| Argentina | 0(0,0) | 0(0,0) | 46.861(1.217,102.582) | -0.048(-0.455,0.360) | 1.347(1.065, 1.629) |
| Chile | 0(0,0) | 0(0,0) | 230.171(135.938,368.586) | 2.590(0.487,4.737) | 6.619(2.856, 10.383) |
| Uruguay | 0(0,0) | 0(0,0) | -64.723(-75.905,-41.799) | -3.645(-3.897,-3.393) | -3.38(-3.663, -3.097) |
| Canada | 0(0,0) | 0(0,0) | 2178.140(1405.519,3295.142) | 8.582(5.969,11.258) | 13.193(9.696, 16.69) |
| United States of America | 0(0,0) | 0(0,0) | 297.195(252.212,338.987) | 2.669(2.246,3.093) | 4.6(4.261, 4.939) |
| Antigua and Barbuda | 0(0,0) | 0(0,0) | -0.039(-34.317,52.523) | -1.214(-1.564,-0.863) | 0.058(-0.211, 0.328) |
| Bahamas | 0(0,0) | 0(0,0) | 93.372(16.374,213.661) | 0.720(0.478,0.963) | 2.194(1.956, 2.432) |
| Barbados | 0(0,0) | 0(0,0) | 348.782(201.124,532.731) | 4.243(2.834,5.670) | 6.255(5.251, 7.26) |
| Belize | 0(0,0) | 0(0,0) | -42.913(-61.796,-16.297) | -2.824(-4.057,-1.575) | -0.974(-1.844, -0.104) |
| Cuba | 0(0,0) | 0(0,0) | 10.817(-27.640,92.420) | 0.304(-2.681,3.381) | 6.214(-3.553, 15.981) |
| Dominica | 0(0,0) | 0(0,0) | -26.465(-56.770,23.454) | -0.761(-1.009,-0.511) | -0.979(-1.093, -0.865) |
| Dominican Republic | 0(0,0) | 0(0,0) | 69.874(-32.280,297.832) | 1.057(0.443,1.675) | 1.907(1.38, 2.434) |
| Grenada | 0(0,0) | 0(0,0) | -32.468(-53.081,-3.457) | -0.985(-1.296,-0.672) | -1.18(-1.287, -1.072) |
| Guyana | 0(0,0) | 0(0,0) | -19.159(-55.321,39.861) | 0.518(0.030,1.009) | -0.643(-1.07, -0.217) |
| Haiti | 0(0,0) | 0(0,0) | 5.086(-46.603,97.234) | -2.243(-2.349,-2.137) | 0.161(0.087, 0.236) |
| Jamaica | 0(0,0) | 0(0,0) | 8112.279(4906.958,12131.495) | 12.712(6.814,18.936) | 41.615(36.27, 46.959) |
| Saint Lucia | 0(0,0) | 0(0,0) | 221.402(49.228,1055.976) | 0.333(-4.125,4.999) | 15.8(6.69, 24.91) |
| Saint Vincent and the Grenadines | 0(0,0) | 0(0,0) | -92.652(-95.250,-86.114) | 6.945(-0.779,15.270) | 12.161(-2.802, 27.123) |
| Suriname | 0(0,0) | 0(0,0) | 882.175(293.039,2357.603) | 3.603(0.852,6.430) | 9.399(8.781, 10.018) |
| Trinidad and Tobago | 0(0,0) | 0(0,0) | 5895.567(3580.655,9655.278) | 10.203(6.710,13.811) | 15.077(13.876, 16.279) |
| Bolivia (Plurinational State of) | 0(0,0) | 0(0,0) | -95.877(-98.397,-89.904) | -12.275(-13.348,-11.188) | -9.633(-9.919, -9.347) |
| Ecuador | 1(0,1) | 0(0,0) | -99.972(-99.982,-99.957) | -28.714(-31.911,-25.366) | -11.97(-25.781, 1.841) |
| Peru | 0(0,0) | 0(0,0) | -98.084(-99.445,-85.146) | -12.091(-14.132,-10.001) | -10.564(-11.217, -9.911) |
| Colombia | 0(0,0) | 0(0,0) | -99.479(-99.627,-99.258) | -15.874(-18.992,-12.637) | -7.139(-13.241, -1.037) |
| Costa Rica | 0(0,0) | 0(0,0) | 23.054(-60.086,268.024) | -0.651(-1.229,-0.071) | 0.716(0.107, 1.325) |
| El Salvador | 0(0,0) | 0(0,0) | -99.652(-99.855,-96.227) | -20.563(-22.751,-18.314) | -15.354(-16.034, -14.674) |
| Guatemala | 0(0,0) | 0(0,0) | -99.719(-99.814,-99.609) | -20.084(-23.134,-16.914) | -10.3(-16.273, -4.327) |
| Honduras | 0(0,0) | 0(0,0) | -41.398(-70.472,6.022) | -4.461(-4.689,-4.232) | -1.675(-1.79, -1.561) |
| Mexico | 2(2,2) | 0(0,0) | -99.900(-99.918,-99.878) | -19.974(-20.744,-19.196) | -20.536(-21.583, -19.489) |
| Nicaragua | 0(0,0) | 0(0,0) | -84.901(-93.239,-68.699) | -9.476(-11.089,-7.834) | -5.166(-6.788, -3.544) |
| Panama | 0(0,0) | 0(0,0) | -57.772(-74.110,-27.633) | -3.448(-3.898,-2.996) | -2.651(-2.759, -2.543) |
| Venezuela (Bolivarian Republic of) | 0(0,0) | 0(0,0) | -95.822(-97.291,-93.772) | -12.809(-15.196,-10.355) | -6.67(-7.952, -5.388) |
| Brazil | 3(2,4) | 0(0,0) | -99.484(-99.638,-99.263) | -17.356(-18.498,-16.199) | -14.753(-15.867, -13.64) |
| Paraguay | 0(0,0) | 0(0,0) | -99.034(-99.653,-80.234) | -22.349(-25.315,-19.266) | -10.474(-11.962, -8.987) |
| Algeria | 0(0,0) | 0(0,0) | 13.369(-36.756,142.416) | -1.257(-1.421,-1.092) | 0.4(0.316, 0.483) |
| Bahrain | 0(0,0) | 0(0,0) | 12.306(-39.698,122.779) | -3.608(-3.890,-3.325) | 0.419(0.338, 0.499) |
| Egypt | 3(2,5) | 0(0,0) | -90.500(-94.349,-81.978) | -9.245(-9.850,-8.636) | -7.352(-7.8, -6.904) |
| Iran (Islamic Republic of) | 0(0,0) | 0(0,0) | -11.827(-50.422,75.294) | 0.479(-0.500,1.468) | -0.288(-0.734, 0.157) |
| Iraq | 0(0,0) | 0(0,0) | -38.559(-78.641,65.512) | -2.884(-3.513,-2.251) | -1.392(-1.657, -1.127) |
| Jordan | 0(0,0) | 0(0,0) | 104.234(12.551,290.975) | -1.054(-1.457,-0.648) | 2.348(2.102, 2.595) |
| Kuwait | 0(0,0) | 0(0,0) | 213.504(12.873,766.197) | -4.917(-6.509,-3.297) | 6.65(3.846, 9.454) |
| Lebanon | 0(0,0) | 0(0,0) | 19.136(-39.759,143.734) | -0.989(-1.247,-0.731) | 0.617(0.523, 0.711) |
| Libya | 0(0,0) | 0(0,0) | 104.430(-7.332,384.243) | 1.007(0.554,1.463) | 2.408(2.255, 2.561) |
| Morocco | 0(0,0) | 0(0,0) | -10.691(-53.729,95.110) | -1.060(-1.294,-0.824) | -0.333(-0.432, -0.234) |
| Palestine | 0(0,0) | 0(0,0) | -27.538(-70.609,160.301) | -3.595(-3.734,-3.456) | -1(-1.071, -0.929) |
| Oman | 0(0,0) | 0(0,0) | -25.801(-70.009,140.789) | -3.157(-3.439,-2.875) | -0.971(-1.18, -0.761) |
| Qatar | 0(0,0) | 0(0,0) | 580.370(135.423,1604.875) | -1.831(-4.190,0.585) | 9.281(6.212, 12.349) |
| Saudi Arabia | 0(0,0) | 0(0,0) | -24.709(-65.735,84.971) | -4.625(-5.571,-3.669) | 0.01(-0.791, 0.812) |
| Syrian Arab Republic | 0(0,0) | 0(0,0) | 56.835(-42.837,283.519) | 3.166(1.921,4.427) | 2.227(1.624, 2.829) |
| Tunisia | 0(0,0) | 0(0,0) | -1.233(-51.893,166.601) | -0.995(-1.102,-0.888) | -0.047(-0.08, -0.015) |
| Türkiye | 0(0,0) | 0(0,0) | -67.299(-83.366,-27.098) | -4.698(-4.880,-4.515) | -3.514(-3.616, -3.411) |
| United Arab Emirates | 0(0,0) | 0(0,0) | 25.779(-48.414,346.146) | -4.134(-4.538,-3.728) | 0.857(0.511, 1.204) |
| Yemen | 0(0,0) | 0(0,0) | 112.905(-7.186,517.471) | -0.544(-0.774,-0.314) | 2.489(2.384, 2.593) |
| Afghanistan | 0(0,0) | 0(0,0) | 99.330(8.116,467.127) | -1.675(-2.114,-1.233) | 2.299(2.053, 2.545) |
| Bangladesh | 11(3,22) | 1(0,1) | -92.197(-96.417,-78.839) | -11.854(-13.132,-10.556) | -7.516(-8.077, -6.956) |
| Bhutan | 0(0,1) | 0(0,0) | -80.401(-89.463,-46.933) | -6.542(-6.909,-6.174) | -5.134(-5.324, -4.944) |
| India | 488(362,608) | 156(98,220) | -68.031(-76.013,-56.417) | -5.872(-6.133,-5.610) | -3.6(-3.742, -3.457) |
| Nepal | 41(20,68) | 19(10,29) | -54.506(-74.749,-17.141) | -4.233(-4.371,-4.095) | -2.502(-2.544, -2.459) |
| Pakistan | 52(27,88) | 24(15,35) | -54.398(-71.860,-21.667) | -5.400(-5.665,-5.133) | -2.474(-2.59, -2.357) |
| Angola | 0(0,0) | 0(0,1) | 1.827(-61.667,110.930) | -3.574(-3.814,-3.333) | 0.028(-0.091, 0.147) |
| Central African Republic | 0(0,0) | 0(0,0) | 39.834(-34.094,190.540) | -1.184(-1.303,-1.065) | 1.119(1.051, 1.188) |
| Congo | 0(0,0) | 0(0,0) | -10.460(-62.834,69.494) | -2.876(-2.954,-2.799) | -0.35(-0.406, -0.295) |
| Democratic Republic of the Congo | 0(0,1) | 1(0,2) | 44.934(-32.690,188.684) | -1.330(-1.581,-1.078) | 1.2(1.079, 1.32) |
| Equatorial Guinea | 0(0,0) | 0(0,0) | -16.922(-70.480,268.872) | -5.591(-5.875,-5.306) | -0.575(-0.753, -0.396) |
| Gabon | 0(0,0) | 0(0,0) | -35.837(-71.698,26.398) | -3.135(-3.207,-3.062) | -1.417(-1.459, -1.375) |
| Burundi | 1(1,2) | 2(0,5) | 36.593(-56.183,213.533) | -1.839(-2.119,-1.558) | 1.004(0.841, 1.168) |
| Comoros | 0(0,0) | 0(0,0) | -17.237(-60.173,65.022) | -2.200(-2.593,-1.805) | -0.619(-0.949, -0.288) |
| Djibouti | 0(0,0) | 0(0,0) | 128.285(18.377,390.444) | -1.067(-1.460,-0.673) | 2.735(2.564, 2.907) |
| Eritrea | 1(0,1) | 1(0,1) | 3.913(-47.854,92.750) | -1.821(-1.950,-1.692) | 0.137(-0.101, 0.375) |
| Ethiopia | 68(21,167) | 40(12,108) | -40.662(-72.235,10.199) | -4.471(-4.710,-4.231) | -1.693(-1.818, -1.567) |
| Kenya | 3(1,6) | 3(1,6) | 10.988(-32.787,83.233) | -1.368(-1.758,-0.976) | 0.38(0.234, 0.526) |
| Madagascar | 1(1,2) | 2(1,4) | 77.136(-12.757,212.418) | -0.798(-1.129,-0.466) | 1.862(1.766, 1.958) |
| Malawi | 3(2,5) | 3(1,5) | 3.951(-51.470,124.902) | -2.153(-2.466,-1.840) | 0.111(0.015, 0.207) |
| Mauritius | 0(0,0) | 0(0,0) | -99.160(-99.374,-98.797) | -8.436(-11.532,-5.231) | -12.38(-14.954, -9.807) |
| Mozambique | 4(2,7) | 5(2,9) | 7.331(-52.834,97.568) | -1.962(-2.143,-1.781) | 0.249(0.186, 0.311) |
| Rwanda | 2(1,4) | 1(0,4) | -35.430(-80.575,45.211) | -4.147(-4.559,-3.733) | -1.608(-2.375, -0.842) |
| Seychelles | 0(0,0) | 0(0,0) | 221.624(70.585,442.663) | 1.999(0.194,3.836) | 4.693(3.77, 5.615) |
| Somalia | 2(0,4) | 4(1,12) | 170.975(12.622,423.396) | -0.053(-0.255,0.149) | 3.371(3.1, 3.641) |
| United Republic of Tanzania | 6(3,10) | 6(2,13) | 5.301(-60.968,128.865) | -2.296(-2.543,-2.049) | 0.169(0.079, 0.26) |
| Uganda | 2(1,4) | 3(1,6) | 55.154(-26.142,264.696) | -1.935(-2.268,-1.601) | 1.392(1.185, 1.599) |
| Zambia | 2(1,3) | 2(1,4) | -2.821(-55.651,106.783) | -3.121(-3.582,-2.658) | -0.023(-0.173, 0.128) |
| Botswana | 0(0,0) | 0(0,0) | -25.298(-67.852,71.776) | -2.663(-2.838,-2.487) | -0.912(-1.022, -0.803) |
| Lesotho | 0(0,0) | 0(0,0) | -21.518(-65.980,84.101) | -0.789(-1.120,-0.457) | -0.711(-0.833, -0.589) |
| Namibia | 0(0,0) | 0(0,0) | -2.676(-53.459,100.480) | -1.845(-1.976,-1.714) | -0.052(-0.149, 0.044) |
| South Africa | 1(0,1) | 0(0,1) | -32.169(-58.541,10.161) | -2.789(-3.471,-2.102) | -1.169(-1.469, -0.869) |
| Eswatini | 0(0,0) | 0(0,0) | -22.578(-65.483,74.753) | -1.728(-1.828,-1.628) | -0.771(-0.902, -0.639) |
| Zimbabwe | 0(0,1) | 1(0,1) | 71.262(-16.701,234.302) | 0.917(0.494,1.343) | 1.716(1.494, 1.939) |
| Benin | 1(0,2) | 1(0,3) | 73.084(-29.726,350.062) | -1.369(-1.502,-1.236) | 1.742(1.524, 1.961) |
| Burkina Faso | 2(1,5) | 3(1,6) | 21.606(-44.405,161.775) | -2.398(-2.607,-2.189) | 0.657(0.56, 0.754) |
| Cameroon | 1(0,3) | 2(1,5) | 85.603(-13.957,345.120) | -1.221(-1.457,-0.984) | 2.057(1.976, 2.139) |
| Cabo Verde | 0(0,0) | 0(0,0) | -34.063(-66.033,69.998) | -3.256(-3.540,-2.971) | -1.315(-1.458, -1.172) |
| Chad | 1(0,3) | 3(0,7) | 110.341(-2.960,347.035) | -0.851(-0.950,-0.752) | 2.504(2.27, 2.737) |
| Côte d'Ivoire | 2(0,4) | 2(1,5) | 49.879(-24.072,224.538) | -0.971(-1.233,-0.708) | 1.253(0.98, 1.525) |
| Gambia | 0(0,0) | 0(0,0) | 56.873(-26.684,273.379) | -1.606(-1.855,-1.356) | 1.467(1.219, 1.715) |
| Ghana | 4(2,7) | 5(2,8) | 20.338(-34.224,122.077) | -1.694(-1.947,-1.440) | 0.651(0.541, 0.762) |
| Guinea | 1(0,3) | 2(0,3) | 11.895(-51.679,122.676) | -1.694(-1.883,-1.506) | 0.406(0.311, 0.502) |
| Guinea-Bissau | 0(0,1) | 0(0,1) | 1.627(-51.161,101.399) | -1.813(-1.963,-1.663) | 0.066(0.011, 0.121) |
| Liberia | 0(0,1) | 0(0,1) | 10.903(-58.188,118.977) | -2.377(-2.512,-2.242) | 0.391(0.052, 0.731) |
| Mali | 2(0,6) | 3(1,7) | 40.978(-32.621,175.121) | -2.095(-2.325,-1.864) | 1.201(1.05, 1.352) |
| Mauritania | 0(0,1) | 0(0,0) | -15.511(-56.849,57.783) | -2.649(-2.869,-2.429) | -0.544(-0.639, -0.449) |
| Niger | 3(1,7) | 5(1,13) | 79.425(-46.292,317.235) | -1.796(-1.967,-1.625) | 1.933(1.692, 2.175) |
| Nigeria | 26(14,41) | 35(15,61) | 35.645(-38.166,154.618) | -1.826(-2.093,-1.559) | 0.996(0.901, 1.09) |
| Sao Tome and Principe | 0(0,0) | 0(0,0) | -34.364(-68.272,54.280) | -3.126(-3.253,-2.999) | -1.264(-1.4, -1.127) |
| Senegal | 2(0,3) | 1(0,3) | -14.591(-57.726,67.911) | -2.311(-2.570,-2.051) | -0.483(-0.587, -0.379) |
| Sierra Leone | 1(0,2) | 1(0,2) | 2.664(-60.657,124.416) | -2.362(-2.655,-2.068) | 0.097(-0.077, 0.27) |
| Togo | 1(0,1) | 1(0,1) | 28.480(-38.485,157.886) | -1.601(-1.735,-1.467) | 0.825(0.766, 0.883) |
| American Samoa | 0(0,0) | 0(0,0) | 12.749(-44.349,173.385) | 0.552(0.432,0.673) | 0.425(0.325, 0.525) |
| Bermuda | 0(0,0) | 0(0,0) | 88.531(13.954,204.854) | 2.249(1.652,2.850) | 2.211(1.904, 2.518) |
| Cook Islands | 0(0,0) | 0(0,0) | -21.780(-69.323,185.624) | -0.719(-0.996,-0.442) | -0.864(-1.217, -0.512) |
| Greenland | 0(0,0) | 0(0,0) | 60.160(-26.037,227.247) | 2.817(2.148,3.490) | 1.625(0.735, 2.515) |
| Guam | 0(0,0) | 0(0,0) | 1.819(-38.921,67.732) | 0.429(0.115,0.744) | 0.042(-0.295, 0.379) |
| Monaco | 0(0,0) | 0(0,0) | -45.661(-76.121,17.069) | -2.046(-3.124,-0.956) | -1.766(-2.085, -1.446) |
| Nauru | 0(0,0) | 0(0,0) | 33.187(-41.131,213.884) | 0.376(-0.187,0.942) | 0.931(0.79, 1.071) |
| Niue | 0(0,0) | 0(0,0) | -51.324(-80.319,30.863) | -1.060(-1.240,-0.879) | -2.281(-2.442, -2.12) |
| Northern Mariana Islands | 0(0,0) | 0(0,0) | 40.648(-24.553,171.100) | 1.118(0.661,1.577) | 0.993(0.487, 1.5) |
| Palau | 0(0,0) | 0(0,0) | 33.483(-46.590,248.202) | 0.150(-0.001,0.302) | 1.035(0.809, 1.262) |
| Puerto Rico | 0(0,0) | 0(0,0) | 1228.045(592.100,1810.349) | 9.429(6.841,12.079) | 12.348(10.066, 14.63) |
| Saint Kitts and Nevis | 0(0,0) | 0(0,0) | -19.121(-38.220,8.362) | -1.712(-1.849,-1.575) | -0.653(-0.73, -0.577) |
| San Marino | 0(0,0) | 0(0,0) | -97.465(-99.043,-93.719) | -17.343(-19.835,-14.775) | -9.128(-10.953, -7.303) |
| Tokelau | 0(0,0) | 0(0,0) | -31.990(-70.966,85.252) | -0.786(-1.227,-0.342) | -1.082(-1.366, -0.798) |
| Tuvalu | 0(0,0) | 0(0,0) | -21.169(-70.504,128.622) | -1.701(-1.899,-1.503) | -0.751(-0.847, -0.655) |
| United States Virgin Islands | 0(0,0) | 0(0,0) | -14.537(-56.840,77.075) | 0.355(-0.020,0.732) | -0.483(-0.76, -0.205) |
| South Sudan | 1(0,1) | 1(0,3) | 78.368(-18.160,239.219) | -0.017(-0.393,0.360) | 1.918(1.638, 2.198) |
| Sudan | 0(0,0) | 0(0,0) | 3.801(-51.483,137.674) | -2.279(-2.537,-2.019) | 0.121(-0.002, 0.244) |

Abbreviations: AAPC, average annual percent change; CI, confidence interval; EAPC, estimated annual percentage change; UI, uncertainty interval.

Supplementary Table 9: Trends in the ASMR of rabies in 204 countries and territories worldwide from 1990 to 2021.

| Location | ASMR (per 100,000 population)  (95% UI).  1990 year | ASMR (per 100,000 population)  (95% UI).  2021 year | Percentage change  (95% UI). 1990－2021. | EAPC (95% CI). 1990－2021. | AAPC (95% CI). 1990－2021. |
| --- | --- | --- | --- | --- | --- |
| China | 0.100(0.059,0.139) | 0.036(0.020,0.055) | -63.935(-75.714,-48.166) | -1.055(-3.235,1.174) | -2.224(-3.15, -1.298) |
| Democratic People's Republic of Korea | 0.052(0.022,0.095) | 0.056(0.022,0.101) | 7.525(-36.952,84.613) | 0.257(-0.177,0.693) | 0.282(0.171, 0.394) |
| Taiwan (Province of China) | 0.000(0.000,0.000) | 0.000(0.000,0.000) | -69.016(-77.238,-58.532) | -1.811(-3.832,0.252) | -3.8(-7.637, 0.038) |
| Cambodia | 0.546(0.183,0.976) | 0.173(0.055,0.338) | -68.415(-81.890,-47.518) | -4.629(-5.115,-4.140) | -3.535(-3.752, -3.317) |
| Indonesia | 0.069(0.044,0.104) | 0.021(0.012,0.036) | -69.364(-84.371,-45.004) | -3.798(-4.048,-3.548) | -3.742(-3.984, -3.5) |
| Lao People's Democratic Republic | 0.598(0.229,1.079) | 0.184(0.073,0.320) | -69.161(-82.351,-44.650) | -4.181(-4.633,-3.727) | -3.73(-3.929, -3.532) |
| Malaysia | 0.004(0.002,0.007) | 0.001(0.001,0.002) | -64.042(-80.213,-23.370) | -3.125(-3.401,-2.849) | -3.289(-3.852, -2.726) |
| Maldives | 0.007(0.003,0.015) | 0.001(0.001,0.003) | -79.353(-89.986,-53.019) | -4.767(-5.132,-4.401) | -4.912(-5.563, -4.26) |
| Myanmar | 1.349(0.786,2.178) | 0.425(0.195,0.758) | -68.472(-84.680,-39.998) | -3.956(-4.155,-3.756) | -3.67(-3.912, -3.428) |
| Philippines | 0.848(0.635,1.049) | 0.237(0.142,0.334) | -72.026(-79.524,-64.108) | -4.988(-5.614,-4.358) | -3.898(-4.093, -3.703) |
| Sri Lanka | 0.598(0.308,0.916) | 0.088(0.035,0.159) | -85.245(-92.840,-71.917) | -7.980(-8.845,-7.107) | -5.77(-6.511, -5.028) |
| Thailand | 0.041(0.018,0.076) | 0.006(0.003,0.010) | -85.127(-91.898,-70.709) | -7.593(-11.178,-3.863) | -1.435(-2.809, -0.06) |
| Timor-Leste | 0.438(0.111,0.939) | 0.182(0.060,0.337) | -58.454(-74.821,-22.027) | -3.226(-3.477,-2.973) | -2.758(-3.02, -2.495) |
| Viet Nam | 0.167(0.076,0.277) | 0.057(0.029,0.096) | -65.670(-79.820,-36.538) | -3.484(-3.585,-3.383) | -3.396(-3.482, -3.31) |
| Fiji | 0.011(0.004,0.028) | 0.012(0.005,0.025) | 0.623(-60.874,179.345) | 0.819(0.194,1.449) | 0.259(-0.368, 0.886) |
| Kiribati | 0.086(0.044,0.170) | 0.067(0.030,0.133) | -22.100(-63.991,68.277) | -0.706(-0.815,-0.597) | -0.767(-0.952, -0.583) |
| Marshall Islands | 0.036(0.010,0.110) | 0.021(0.006,0.052) | -42.238(-74.485,44.695) | -2.041(-2.295,-1.786) | -1.812(-2.127, -1.498) |
| Micronesia (Federated States of) | 0.050(0.018,0.120) | 0.027(0.011,0.062) | -46.214(-74.764,18.090) | -1.840(-1.916,-1.763) | -2.021(-2.164, -1.878) |
| Papua New Guinea | 0.045(0.007,0.177) | 0.033(0.006,0.107) | -26.805(-71.398,92.780) | -0.939(-1.018,-0.860) | -0.917(-1.074, -0.76) |
| Samoa | 0.042(0.014,0.106) | 0.028(0.010,0.065) | -32.960(-68.133,50.995) | -1.067(-1.157,-0.977) | -1.296(-1.402, -1.189) |
| Solomon Islands | 0.044(0.007,0.150) | 0.033(0.008,0.105) | -25.677(-66.250,141.787) | -1.138(-1.266,-1.009) | -1.048(-1.269, -0.828) |
| Tonga | 0.031(0.010,0.089) | 0.019(0.007,0.049) | -37.965(-71.410,53.476) | -1.305(-1.565,-1.044) | -1.491(-1.795, -1.187) |
| Vanuatu | 0.041(0.009,0.128) | 0.027(0.006,0.082) | -34.195(-70.364,59.857) | -1.324(-1.431,-1.217) | -1.357(-1.531, -1.184) |
| Armenia | 0.000(0.000,0.001) | 0.000(0.000,0.000) | -85.665(-91.729,-75.071) | -7.075(-10.361,-3.669) | -0.847(-9.7, 8.005) |
| Azerbaijan | 0.038(0.012,0.089) | 0.014(0.004,0.033) | -63.058(-89.234,25.460) | -4.623(-5.557,-3.680) | -2.858(-3.361, -2.355) |
| Georgia | 0.021(0.011,0.036) | 0.016(0.009,0.026) | -20.354(-62.262,67.078) | -1.879(-5.070,1.419) | 8.687(1.501, 15.873) |
| Kazakhstan | 0.011(0.007,0.016) | 0.017(0.009,0.031) | 52.861(-24.726,218.322) | 1.443(-0.945,3.890) | 8.066(0.674, 15.458) |
| Kyrgyzstan | 0.033(0.018,0.057) | 0.009(0.005,0.016) | -71.532(-88.951,-32.780) | -6.350(-9.057,-3.561) | 0.413(-5.284, 6.11) |
| Mongolia | 0.150(0.015,0.519) | 0.046(0.004,0.159) | -69.229(-94.233,55.466) | -4.449(-4.823,-4.073) | -3.266(-4.515, -2.016) |
| Tajikistan | 0.001(0.000,0.003) | 0.000(0.000,0.001) | -58.830(-85.903,37.485) | -3.363(-3.768,-2.957) | -2.679(-3.046, -2.312) |
| Turkmenistan | 0.003(0.002,0.005) | 0.001(0.000,0.002) | -73.956(-89.218,-35.188) | -1.609(-8.870,6.230) | 73.028(4.028, 142.028) |
| Uzbekistan | 0.011(0.006,0.021) | 0.005(0.003,0.008) | -55.191(-75.205,-16.577) | -4.298(-5.135,-3.453) | -1.91(-4.251, 0.432) |
| Albania | 0.001(0.000,0.001) | 0.000(0.000,0.000) | -90.085(-96.979,-67.837) | -10.039(-11.650,-8.399) | -5.594(-6.385, -4.804) |
| Bosnia and Herzegovina | 0.000(0.000,0.000) | 0.000(0.000,0.000) | -88.449(-96.841,-62.226) | -6.849(-8.033,-5.651) | -6.17(-6.576, -5.765) |
| Bulgaria | 0.000(0.000,0.001) | 0.001(0.000,0.001) | 50.362(-6.828,137.007) | 1.156(-0.267,2.599) | -2.015(-7.945, 3.915) |
| Croatia | 0.001(0.001,0.001) | 0.000(0.000,0.000) | -98.666(-99.168,-97.801) | -15.588(-18.435,-12.641) | -3.178(-9.95, 3.594) |
| Czechia | 0.000(0.000,0.000) | 0.000(0.000,0.000) | -77.160(-84.603,-65.713) | -5.375(-8.715,-1.913) | 2.179(-3.896, 8.253) |
| Hungary | 0.000(0.000,0.000) | 0.000(0.000,0.000) | 6.385(-22.888,50.453) | -7.432(-10.760,-3.980) | -1.669(-2.828, -0.51) |
| North Macedonia | 0.000(0.000,0.000) | 0.000(0.000,0.000) | -55.151(-81.678,19.414) | -3.975(-5.275,-2.658) | -1.421(-1.744, -1.098) |
| Montenegro | 0.001(0.000,0.002) | 0.001(0.000,0.001) | -40.624(-74.660,32.291) | -2.072(-2.391,-1.751) | -15.647(-21.174, -10.12) |
| Poland | 0.004(0.004,0.005) | 0.000(0.000,0.000) | -99.701(-99.764,-99.565) | -20.145(-22.753,-17.450) | -5.346(-5.886, -4.806) |
| Romania | 0.011(0.010,0.013) | 0.000(0.000,0.000) | -99.761(-99.827,-99.670) | -17.404(-20.779,-13.886) | -3.018(-3.272, -2.763) |
| Serbia | 0.001(0.000,0.001) | 0.000(0.000,0.000) | -85.088(-92.777,-65.723) | -7.826(-8.876,-6.764) | 4.447(-3.812, 12.707) |
| Slovakia | 0.000(0.000,0.001) | 0.000(0.000,0.000) | -62.232(-81.850,-20.453) | -3.104(-3.217,-2.991) | 5.198(2.544, 7.852) |
| Slovenia | 0.000(0.000,0.000) | 0.000(0.000,0.000) | -41.161(-56.129,-15.858) | -5.390(-8.337,-2.348) | 2.174(-3.461, 7.81) |
| Belarus | 0.002(0.002,0.003) | 0.010(0.008,0.014) | 345.136(167.032,561.635) | 4.772(2.672,6.916) | -2.605(-13.316, 8.105) |
| Estonia | 0.000(0.000,0.001) | 0.001(0.000,0.001) | 11.773(-33.283,79.895) | 1.630(-1.976,5.368) | -8.361(-21.681, 4.958) |
| Latvia | 0.019(0.014,0.025) | 0.002(0.002,0.003) | -86.858(-91.420,-80.124) | -7.731(-9.820,-5.593) | -3.504(-7.46, 0.451) |
| Lithuania | 0.002(0.001,0.004) | 0.000(0.000,0.000) | -99.253(-99.620,-98.581) | -14.465(-18.554,-10.171) | 2.235(-11.165, 15.636) |
| Republic of Moldova | 0.000(0.000,0.000) | 0.000(0.000,0.000) | -69.113(-81.747,-47.572) | -3.494(-4.567,-2.410) | -2.667(-3.948, -1.386) |
| Russian Federation | 0.006(0.006,0.007) | 0.001(0.001,0.001) | -79.699(-81.654,-77.472) | -4.004(-5.655,-2.325) | -4.433(-14.459, 5.592) |
| Ukraine | 0.003(0.002,0.004) | 0.002(0.001,0.004) | -9.496(-54.573,86.497) | 0.231(-1.243,1.727) | -8.553(-9.997, -7.109) |
| Brunei Darussalam | 0.000(0.000,0.000) | 0.000(0.000,0.000) | -64.932(-80.427,-32.671) | -2.583(-3.486,-1.672) | 14.948(-6.586, 36.481) |
| Japan | 0.001(0.001,0.001) | 0.000(0.000,0.000) | -92.229(-93.124,-91.287) | -6.705(-8.369,-5.011) | -4.56(-7.577, -1.544) |
| Republic of Korea | 0.000(0.000,0.001) | 0.000(0.000,0.000) | -97.442(-98.874,-85.640) | -12.763(-14.803,-10.675) | -2.476(-2.726, -2.225) |
| Singapore | 0.000(0.000,0.000) | 0.000(0.000,0.000) | -60.609(-72.652,-45.502) | -4.995(-6.622,-3.341) | 3.742(-3.375, 10.86) |
| Australia | 0.000(0.000,0.000) | 0.000(0.000,0.000) | 165.985(55.853,342.763) | 10.781(6.268,15.487) | -3.165(-8.333, 2.004) |
| New Zealand | 0.001(0.000,0.001) | 0.000(0.000,0.000) | -75.964(-83.302,-66.811) | -2.996(-4.867,-1.089) | -5.713(-7.24, -4.186) |
| Andorra | 0.000(0.000,0.000) | 0.000(0.000,0.000) | -53.206(-76.562,-8.432) | -2.364(-2.540,-2.189) | -8.411(-13.143, -3.679) |
| Austria | 0.000(0.000,0.000) | 0.000(0.000,0.000) | -37.152(-55.323,-7.304) | -3.600(-6.809,-0.280) | 3.52(-6.698, 13.738) |
| Belgium | 0.000(0.000,0.000) | 0.000(0.000,0.000) | -70.886(-79.566,-59.347) | 0.975(-1.968,4.005) | 12.571(6.63, 18.513) |
| Cyprus | 0.004(0.001,0.011) | 0.001(0.000,0.001) | -86.065(-94.325,-57.233) | -5.541(-6.632,-4.436) | 0.845(-7.777, 9.467) |
| Denmark | 0.001(0.001,0.001) | 0.000(0.000,0.000) | -89.143(-92.097,-85.597) | -6.940(-9.523,-4.284) | 2.58(-4.777, 9.938) |
| Finland | 0.001(0.000,0.001) | 0.000(0.000,0.001) | -49.956(-73.740,-7.183) | -2.476(-2.808,-2.143) | 0.156(-2.265, 2.576) |
| France | 0.000(0.000,0.000) | 0.000(0.000,0.000) | -24.024(-48.831,12.740) | -3.730(-5.866,-1.545) | 19.41(-0.685, 39.506) |
| Germany | 0.000(0.000,0.000) | 0.000(0.000,0.000) | 376.046(187.362,624.326) | 4.880(2.754,7.051) | 7.719(4.541, 10.896) |
| Greece | 0.000(0.000,0.000) | 0.002(0.001,0.003) | 593.625(362.302,993.052) | 12.972(8.777,17.330) | 1.113(-12.049, 14.275) |
| Iceland | 0.002(0.002,0.003) | 0.001(0.001,0.002) | -35.308(-54.773,-7.609) | -1.905(-5.886,2.244) | -2.893(-5.869, 0.083) |
| Ireland | 0.000(0.000,0.000) | 0.000(0.000,0.000) | -37.550(-53.979,-12.509) | 1.417(-0.705,3.585) | 11.957(-3.07, 26.983) |
| Israel | 0.000(0.000,0.000) | 0.000(0.000,0.000) | -91.179(-94.181,-86.639) | -7.156(-10.178,-4.031) | 10.146(1.935, 18.357) |
| Italy | 0.000(0.000,0.000) | 0.000(0.000,0.000) | -93.105(-94.139,-91.692) | -8.653(-9.873,-7.415) | 25.626(-32, 83.252) |
| Luxembourg | 0.001(0.001,0.001) | 0.000(0.000,0.000) | -56.821(-68.583,-42.320) | -2.234(-6.081,1.772) | 5.77(-15.324, 26.863) |
| Malta | 0.002(0.001,0.002) | 0.000(0.000,0.000) | -97.971(-98.489,-97.230) | -10.537(-13.182,-7.811) | -3.701(-4.514, -2.888) |
| Netherlands | 0.000(0.000,0.000) | 0.000(0.000,0.000) | 218.509(126.510,340.709) | 4.812(1.447,8.289) | 13.678(6.93, 20.427) |
| Norway | 0.001(0.001,0.001) | 0.006(0.005,0.007) | 513.530(393.872,639.423) | 3.962(2.326,5.625) | -0.791(-1.259, -0.323) |
| Portugal | 0.000(0.000,0.001) | 0.000(0.000,0.000) | -95.685(-96.878,-93.764) | -8.746(-11.886,-5.494) | -0.033(-0.472, 0.406) |
| Spain | 0.000(0.000,0.000) | 0.000(0.000,0.000) | -51.196(-63.461,-34.416) | -0.944(-2.216,0.345) | 5.923(-0.146, 11.992) |
| Sweden | 0.000(0.000,0.000) | 0.000(0.000,0.000) | 29.116(-9.834,78.458) | -0.868(-4.025,2.393) | -1.251(-6.009, 3.507) |
| Switzerland | 0.000(0.000,0.000) | 0.000(0.000,0.000) | 283.320(157.488,438.471) | 2.945(-0.754,6.782) | -1.737(-2.019, -1.456) |
| United Kingdom | 0.000(0.000,0.000) | 0.001(0.001,0.001) | 601.923(527.602,684.358) | 7.052(5.162,8.975) | -1.703(-2.12, -1.286) |
| Argentina | 0.000(0.000,0.000) | 0.000(0.000,0.000) | -18.022(-44.258,11.994) | -1.958(-4.418,0.565) | -0.617(-1.2, -0.034) |
| Chile | 0.000(0.000,0.000) | 0.000(0.000,0.000) | 30.211(-2.029,75.040) | 0.398(-2.057,2.914) | -1.805(-2.415, -1.195) |
| Uruguay | 0.000(0.000,0.000) | 0.000(0.000,0.000) | -70.091(-79.353,-54.679) | -3.750(-4.173,-3.325) | -2.041(-2.176, -1.906) |
| Canada | 0.000(0.000,0.000) | 0.000(0.000,0.000) | 2977.115(1942.320,4484.265) | 10.539(6.876,14.327) | 101.109(21.449, 180.77) |
| United States of America | 0.001(0.001,0.001) | 0.002(0.002,0.002) | 178.753(149.100,210.121) | 2.799(2.097,3.506) | 28.457(-6.017, 62.931) |
| Antigua and Barbuda | 0.007(0.006,0.009) | 0.005(0.004,0.007) | -25.696(-49.811,10.553) | -0.693(-0.915,-0.471) | 17.873(-8.13, 43.875) |
| Bahamas | 0.003(0.002,0.004) | 0.002(0.002,0.004) | -3.918(-40.573,55.886) | -0.221(-0.423,-0.019) | 10.079(8.538, 11.621) |
| Barbados | 0.003(0.002,0.004) | 0.008(0.006,0.010) | 176.054(85.135,285.386) | 3.977(2.344,5.636) | 36.844(21.272, 52.417) |
| Belize | 0.001(0.001,0.001) | 0.000(0.000,0.000) | -54.936(-69.407,-33.029) | -1.490(-2.575,-0.393) | -10.986(-11.269, -10.703) |
| Cuba | 0.000(0.000,0.000) | 0.000(0.000,0.000) | -25.249(-49.673,14.930) | 0.106(-3.456,3.800) | -12.743(-21.748, -3.739) |
| Dominica | 0.008(0.004,0.012) | 0.004(0.002,0.008) | -42.693(-66.961,-4.770) | -1.734(-2.020,-1.447) | -11.995(-13.338, -10.651) |
| Dominican Republic | 0.006(0.002,0.012) | 0.003(0.001,0.008) | -44.727(-78.793,33.949) | -1.181(-1.864,-0.492) | -13.207(-20.128, -6.287) |
| Grenada | 0.010(0.007,0.013) | 0.008(0.005,0.011) | -19.078(-43.285,17.772) | 0.396(0.039,0.754) | -2.506(-3.973, -1.038) |
| Guyana | 0.002(0.001,0.003) | 0.001(0.001,0.001) | -50.483(-72.899,-12.937) | -1.619(-2.437,-0.795) | -15.985(-18.391, -13.579) |
| Haiti | 0.007(0.002,0.019) | 0.003(0.001,0.009) | -47.861(-74.192,0.992) | -2.326(-2.428,-2.224) | -14.331(-25.826, -2.836) |
| Jamaica | 0.000(0.000,0.000) | 0.001(0.000,0.001) | 4330.931(2502.498,6385.127) | 9.859(3.264,16.875) | -3.667(-3.876, -3.458) |
| Saint Lucia | 0.000(0.000,0.001) | 0.000(0.000,0.002) | 10.951(-45.794,297.573) | 1.440(-4.440,7.681) | -5.121(-6.854, -3.387) |
| Saint Vincent and the Grenadines | 0.033(0.026,0.041) | 0.001(0.001,0.002) | -96.374(-97.674,-93.183) | 6.230(-2.200,15.387) | -5.478(-5.82, -5.135) |
| Suriname | 0.000(0.000,0.000) | 0.001(0.000,0.001) | 470.576(141.282,1227.367) | 2.518(-1.036,6.200) | -6.696(-15.467, 2.076) |
| Trinidad and Tobago | 0.000(0.000,0.000) | 0.001(0.001,0.002) | 2921.837(1728.905,4914.358) | 7.936(2.485,13.678) | -11.262(-12.752, -9.772) |
| Bolivia (Plurinational State of) | 0.018(0.009,0.032) | 0.000(0.000,0.001) | -97.438(-98.931,-94.301) | -11.916(-12.865,-10.957) | -1.914(-2.041, -1.787) |
| Ecuador | 0.146(0.126,0.172) | 0.000(0.000,0.000) | -99.983(-99.989,-99.974) | -29.472(-33.428,-25.281) | -3.482(-3.676, -3.289) |
| Peru | 0.010(0.003,0.016) | 0.000(0.000,0.000) | -98.748(-99.643,-91.004) | -12.423(-14.176,-10.634) | -8.942(-9.431, -8.454) |
| Colombia | 0.007(0.006,0.008) | 0.000(0.000,0.000) | -99.677(-99.765,-99.545) | -12.877(-16.966,-8.587) | -2.109(-2.699, -1.519) |
| Costa Rica | 0.000(0.000,0.000) | 0.000(0.000,0.000) | -44.169(-81.670,67.069) | -1.678(-2.417,-0.934) | -3.407(-3.66, -3.154) |
| El Salvador | 0.025(0.006,0.042) | 0.000(0.000,0.000) | -99.723(-99.871,-97.283) | -20.621(-22.674,-18.513) | -2.172(-2.53, -1.814) |
| Guatemala | 0.092(0.078,0.110) | 0.000(0.000,0.000) | -99.841(-99.890,-99.788) | -18.897(-23.521,-13.993) | 3.93(-4.197, 12.057) |
| Honduras | 0.000(0.000,0.000) | 0.000(0.000,0.000) | -69.436(-82.926,-48.139) | -3.955(-4.136,-3.774) | -2.691(-3.033, -2.348) |
| Mexico | 0.066(0.061,0.071) | 0.000(0.000,0.000) | -99.931(-99.943,-99.917) | -21.059(-22.409,-19.686) | -0.189(-0.475, 0.097) |
| Nicaragua | 0.000(0.000,0.000) | 0.000(0.000,0.000) | -87.762(-94.068,-76.034) | -8.685(-10.344,-6.995) | -2.292(-2.423, -2.161) |
| Panama | 0.001(0.001,0.002) | 0.000(0.000,0.000) | -83.316(-89.344,-71.073) | -4.648(-5.027,-4.268) | -3.761(-3.94, -3.581) |
| Venezuela (Bolivarian Republic of) | 0.001(0.001,0.001) | 0.000(0.000,0.000) | -97.905(-98.614,-96.898) | -14.499(-18.266,-10.559) | -3.291(-3.889, -2.692) |
| Brazil | 0.051(0.036,0.068) | 0.000(0.000,0.000) | -99.642(-99.743,-99.487) | -16.013(-17.834,-14.152) | 0.485(-1.698, 2.667) |
| Paraguay | 0.007(0.003,0.011) | 0.000(0.000,0.000) | -99.400(-99.761,-90.877) | -21.381(-24.414,-18.227) | -3.793(-4.787, -2.798) |
| Algeria | 0.001(0.000,0.002) | 0.001(0.000,0.001) | -44.474(-67.945,31.557) | -1.675(-1.808,-1.542) | 0.892(-0.069, 1.853) |
| Bahrain | 0.005(0.002,0.010) | 0.002(0.001,0.003) | -66.567(-80.071,-38.610) | -3.887(-4.098,-3.675) | -2.236(-2.359, -2.113) |
| Egypt | 0.145(0.080,0.212) | 0.008(0.004,0.013) | -94.327(-96.488,-89.998) | -8.903(-9.489,-8.313) | -5.418(-5.678, -5.158) |
| Iran (Islamic Republic of) | 0.003(0.001,0.005) | 0.001(0.001,0.002) | -53.108(-73.935,-7.066) | -0.336(-1.364,0.702) | -2.616(-3.545, -1.688) |
| Iraq | 0.017(0.005,0.034) | 0.006(0.002,0.011) | -66.608(-85.585,-23.273) | -2.397(-3.034,-1.756) | -0.589(-0.83, -0.348) |
| Jordan | 0.000(0.000,0.000) | 0.000(0.000,0.000) | -49.818(-71.778,-7.042) | -1.938(-2.370,-1.504) | -0.787(-0.952, -0.622) |
| Kuwait | 0.000(0.000,0.001) | 0.000(0.000,0.001) | 36.123(-48.809,227.036) | -2.455(-4.438,-0.431) | -7.996(-8.909, -7.082) |
| Lebanon | 0.000(0.000,0.000) | 0.000(0.000,0.000) | -56.778(-77.280,-10.867) | -2.187(-2.418,-1.956) | -5.921(-6.312, -5.53) |
| Libya | 0.001(0.000,0.002) | 0.001(0.000,0.002) | -7.487(-57.923,119.822) | 0.220(-0.202,0.645) | -5.243(-5.446, -5.04) |
| Morocco | 0.001(0.000,0.004) | 0.001(0.000,0.002) | -50.468(-74.270,6.623) | -1.855(-2.075,-1.634) | -4.186(-4.399, -3.972) |
| Palestine | 0.001(0.000,0.003) | 0.000(0.000,0.001) | -69.356(-86.391,-2.389) | -3.379(-3.695,-3.062) | -4.654(-4.804, -4.504) |
| Oman | 0.000(0.000,0.000) | 0.000(0.000,0.000) | -66.705(-87.576,20.174) | -3.105(-3.470,-2.738) | -3.408(-3.634, -3.181) |
| Qatar | 0.000(0.000,0.000) | 0.000(0.000,0.000) | -48.275(-75.196,29.464) | -3.254(-5.049,-1.426) | -1.001(-1.241, -0.761) |
| Saudi Arabia | 0.000(0.000,0.001) | 0.000(0.000,0.000) | -75.554(-89.182,-39.368) | -5.334(-6.104,-4.557) | -2.74(-3.334, -2.145) |
| Syrian Arab Republic | 0.003(0.001,0.008) | 0.004(0.001,0.009) | 11.316(-61.130,247.044) | 1.509(0.488,2.542) | -1.15(-1.382, -0.919) |
| Tunisia | 0.001(0.000,0.002) | 0.000(0.000,0.001) | -49.740(-75.116,32.336) | -2.114(-2.187,-2.041) | -4.006(-4.503, -3.508) |
| Türkiye | 0.011(0.004,0.026) | 0.002(0.001,0.005) | -82.217(-90.332,-64.900) | -5.426(-5.639,-5.212) | -3.178(-3.39, -2.966) |
| United Arab Emirates | 0.042(0.012,0.104) | 0.017(0.007,0.042) | -58.268(-85.224,39.218) | -1.794(-2.302,-1.283) | -1.562(-1.739, -1.384) |
| Yemen | 0.002(0.000,0.006) | 0.002(0.000,0.004) | -18.564(-64.968,151.804) | -0.801(-1.043,-0.558) | -2.135(-3.2, -1.07) |
| Afghanistan | 0.003(0.000,0.009) | 0.002(0.000,0.005) | -21.412(-56.696,113.938) | -1.101(-1.711,-0.487) | -0.94(-1.369, -0.51) |
| Bangladesh | 0.319(0.101,0.544) | 0.024(0.010,0.042) | -92.628(-96.377,-83.227) | -9.999(-10.912,-9.077) | -2.063(-2.243, -1.882) |
| Bhutan | 1.313(0.278,2.700) | 0.197(0.073,0.376) | -84.973(-91.580,-66.479) | -6.512(-6.934,-6.087) | -4.053(-4.181, -3.926) |
| India | 1.637(1.202,2.021) | 0.303(0.190,0.428) | -81.508(-85.961,-75.296) | -5.876(-6.118,-5.633) | -2.006(-2.198, -1.814) |
| Nepal | 6.184(2.960,10.525) | 1.694(0.910,2.620) | -72.605(-84.391,-51.903) | -4.394(-4.507,-4.281) | -0.901(-1.084, -0.717) |
| Pakistan | 1.369(0.708,2.390) | 0.309(0.186,0.464) | -77.412(-85.595,-61.847) | -5.261(-5.509,-5.011) | -1.849(-1.96, -1.737) |
| Angola | 0.045(0.010,0.113) | 0.016(0.003,0.039) | -63.987(-82.257,-35.426) | -3.258(-3.468,-3.048) | -15.682(-18.227, -13.137) |
| Central African Republic | 0.044(0.006,0.129) | 0.032(0.006,0.082) | -27.201(-58.071,37.229) | -1.091(-1.239,-0.942) | -2.019(-2.213, -1.825) |
| Congo | 0.021(0.004,0.053) | 0.009(0.001,0.022) | -59.759(-79.693,-33.573) | -2.834(-3.010,-2.657) | -3.187(-3.396, -2.978) |
| Democratic Republic of the Congo | 0.030(0.004,0.078) | 0.021(0.004,0.050) | -31.071(-59.356,19.547) | -0.993(-1.252,-0.733) | 3.358(2.014, 4.702) |
| Equatorial Guinea | 0.036(0.004,0.093) | 0.010(0.002,0.024) | -72.974(-89.489,38.165) | -5.093(-5.406,-4.778) | 0.367(0.245, 0.489) |
| Gabon | 0.022(0.006,0.049) | 0.008(0.001,0.019) | -65.192(-83.779,-33.510) | -3.096(-3.215,-2.976) | -2.311(-2.49, -2.132) |
| Burundi | 0.543(0.247,1.055) | 0.329(0.074,0.953) | -39.484(-79.763,32.215) | -1.742(-2.002,-1.481) | -1.52(-1.831, -1.209) |
| Comoros | 0.389(0.150,0.843) | 0.200(0.061,0.496) | -48.597(-73.637,-2.651) | -2.247(-2.590,-1.902) | -2.68(-2.851, -2.51) |
| Djibouti | 0.210(0.056,0.537) | 0.156(0.048,0.381) | -25.643(-58.885,48.803) | -0.895(-1.349,-0.439) | -2.538(-2.867, -2.209) |
| Eritrea | 0.579(0.326,1.040) | 0.306(0.134,0.580) | -47.077(-71.306,-9.710) | -1.798(-1.943,-1.652) | -1.009(-1.207, -0.811) |
| Ethiopia | 3.753(1.077,9.678) | 1.043(0.309,3.024) | -72.203(-85.539,-52.837) | -4.481(-4.754,-4.209) | -1.702(-1.967, -1.437) |
| Kenya | 0.368(0.162,0.684) | 0.198(0.088,0.356) | -46.015(-66.878,-9.978) | -1.397(-1.815,-0.977) | -2.064(-2.502, -1.626) |
| Madagascar | 0.265(0.119,0.456) | 0.199(0.088,0.361) | -24.666(-57.547,26.633) | -0.693(-1.040,-0.345) | -1.607(-1.809, -1.405) |
| Malawi | 1.376(0.703,2.357) | 0.763(0.369,1.378) | -44.584(-75.218,44.339) | -2.059(-2.316,-1.801) | -0.044(-0.355, 0.267) |
| Mauritius | 0.016(0.013,0.019) | 0.000(0.000,0.000) | -99.775(-99.838,-99.672) | -13.322(-15.967,-10.594) | -1.381(-1.647, -1.115) |
| Mozambique | 0.832(0.436,1.288) | 0.438(0.194,0.790) | -47.362(-71.833,-7.866) | -1.610(-1.804,-1.417) | -1.95(-2.137, -1.763) |
| Rwanda | 0.623(0.262,1.455) | 0.225(0.039,0.764) | -63.883(-88.079,-21.520) | -4.079(-4.512,-3.645) | -1.521(-1.775, -1.266) |
| Seychelles | 0.000(0.000,0.000) | 0.001(0.000,0.001) | 114.105(13.249,259.740) | 2.250(0.399,4.136) | -2.854(-3.173, -2.535) |
| Somalia | 0.573(0.100,1.501) | 0.626(0.116,1.850) | 9.239(-47.252,95.675) | 0.345(0.113,0.578) | -1.036(-1.177, -0.895) |
| United Republic of Tanzania | 0.544(0.272,0.913) | 0.262(0.095,0.548) | -51.857(-80.372,0.182) | -2.294(-2.553,-2.033) | -1.284(-1.716, -0.851) |
| Uganda | 0.309(0.110,0.682) | 0.192(0.065,0.388) | -37.678(-70.020,50.299) | -2.022(-2.394,-1.647) | -1.213(-1.818, -0.609) |
| Zambia | 0.691(0.406,1.109) | 0.288(0.130,0.529) | -58.303(-79.181,-17.569) | -2.941(-3.432,-2.448) | -1.873(-2.045, -1.701) |
| Botswana | 0.068(0.027,0.143) | 0.032(0.015,0.059) | -53.457(-79.360,-0.014) | -2.259(-2.434,-2.085) | -1.781(-2.042, -1.521) |
| Lesotho | 0.084(0.033,0.177) | 0.061(0.029,0.107) | -27.831(-66.270,53.810) | -0.405(-0.809,0.001) | -1.884(-2.152, -1.617) |
| Namibia | 0.059(0.026,0.113) | 0.035(0.014,0.068) | -41.465(-69.272,8.594) | -1.701(-1.831,-1.571) | -1.947(-2.392, -1.501) |
| South Africa | 0.039(0.020,0.063) | 0.020(0.011,0.030) | -48.933(-67.435,-19.383) | -2.198(-2.976,-1.415) | -1.92(-2.144, -1.696) |
| Eswatini | 0.079(0.037,0.150) | 0.047(0.024,0.084) | -40.631(-70.087,19.089) | -1.271(-1.466,-1.076) | -2.914(-3.177, -2.65) |
| Zimbabwe | 0.119(0.055,0.206) | 0.117(0.062,0.209) | -1.912(-51.116,100.051) | 0.449(-0.061,0.961) | -1.194(-1.656, -0.731) |
| Benin | 0.400(0.107,0.946) | 0.263(0.086,0.555) | -34.290(-67.756,51.694) | -1.205(-1.339,-1.071) | -2.171(-2.283, -2.058) |
| Burkina Faso | 0.574(0.161,1.350) | 0.308(0.103,0.677) | -46.280(-72.017,6.793) | -2.244(-2.471,-2.017) | -2.721(-3.141, -2.301) |
| Cameroon | 0.323(0.100,0.647) | 0.204(0.080,0.365) | -36.708(-66.199,33.230) | -1.207(-1.446,-0.969) | -2.451(-2.759, -2.143) |
| Cabo Verde | 0.233(0.061,0.557) | 0.093(0.034,0.179) | -59.968(-79.986,-4.371) | -3.308(-3.540,-3.075) | -2.05(-2.374, -1.725) |
| Chad | 0.541(0.087,1.480) | 0.385(0.053,0.973) | -28.731(-61.439,34.640) | -1.019(-1.141,-0.897) | -1.595(-1.782, -1.408) |
| Côte d'Ivoire | 0.330(0.086,0.695) | 0.221(0.067,0.433) | -33.107(-62.438,22.983) | -0.877(-1.112,-0.641) | -1.719(-2.097, -1.34) |
| Gambia | 0.346(0.077,0.837) | 0.236(0.090,0.519) | -31.825(-65.147,53.511) | -1.330(-1.580,-1.079) | -1.14(-1.604, -0.677) |
| Ghana | 0.751(0.336,1.294) | 0.412(0.189,0.723) | -45.137(-69.295,-1.163) | -1.502(-1.817,-1.185) | -3.143(-3.425, -2.861) |
| Guinea | 0.556(0.154,1.238) | 0.314(0.081,0.644) | -43.464(-70.655,0.800) | -1.386(-1.577,-1.194) | 1.689(0.566, 2.812) |
| Guinea-Bissau | 0.564(0.152,1.288) | 0.310(0.086,0.668) | -45.016(-68.432,-0.990) | -1.458(-1.641,-1.274) | -2.589(-3.127, -2.051) |
| Liberia | 0.359(0.082,0.796) | 0.199(0.038,0.466) | -44.590(-73.611,-0.783) | -2.208(-2.442,-1.973) | -2.007(-2.92, -1.094) |
| Mali | 0.606(0.094,1.681) | 0.326(0.066,0.789) | -46.201(-70.249,-2.851) | -1.859(-2.063,-1.654) | -0.545(-0.861, -0.23) |
| Mauritania | 0.324(0.115,0.638) | 0.130(0.040,0.261) | -59.893(-77.844,-26.842) | -2.743(-2.909,-2.577) | -1.293(-1.49, -1.096) |
| Niger | 0.710(0.121,2.016) | 0.469(0.078,1.271) | -34.012(-72.752,23.339) | -1.495(-1.697,-1.291) | -1.363(-1.857, -0.87) |
| Nigeria | 0.700(0.350,1.156) | 0.357(0.148,0.646) | -48.971(-73.476,-8.122) | -2.042(-2.325,-1.758) | -0.907(-1.006, -0.807) |
| Sao Tome and Principe | 0.270(0.067,0.589) | 0.120(0.040,0.233) | -55.742(-74.777,-0.406) | -2.885(-3.172,-2.597) | 8.621(2.405, 14.837) |
| Senegal | 0.495(0.160,1.033) | 0.229(0.077,0.481) | -53.802(-74.083,-16.564) | -2.175(-2.368,-1.982) | -0.905(-1.277, -0.533) |
| Sierra Leone | 0.433(0.152,0.935) | 0.230(0.057,0.557) | -47.011(-74.816,1.110) | -1.934(-2.247,-1.619) | -12.113(-13.45, -10.777) |
| Togo | 0.395(0.157,0.820) | 0.238(0.091,0.485) | -39.860(-67.736,5.594) | -1.416(-1.568,-1.264) | -1.856(-2.088, -1.624) |
| American Samoa | 0.029(0.011,0.055) | 0.016(0.006,0.029) | -46.303(-76.829,16.132) | -1.839(-2.048,-1.629) | -2.921(-2.988, -2.853) |
| Bermuda | 0.006(0.004,0.007) | 0.004(0.002,0.005) | -34.756(-60.594,5.307) | -0.934(-1.361,-0.505) | -1.985(-2.289, -1.681) |
| Cook Islands | 0.055(0.012,0.128) | 0.021(0.004,0.037) | -62.988(-84.346,35.165) | -3.431(-3.711,-3.149) | 0.295(-0.089, 0.679) |
| Greenland | 0.002(0.001,0.003) | 0.003(0.001,0.005) | 44.656(-28.712,173.938) | 3.136(2.052,4.230) | -2.392(-2.499, -2.284) |
| Guam | 0.009(0.004,0.016) | 0.004(0.002,0.007) | -55.720(-73.516,-29.235) | -1.622(-2.049,-1.192) | -0.389(-0.753, -0.026) |
| Monaco | 0.004(0.002,0.007) | 0.002(0.001,0.003) | -53.136(-77.499,-9.847) | -2.060(-2.963,-1.149) | 2.073(1.473, 2.672) |
| Nauru | 0.033(0.010,0.069) | 0.027(0.009,0.055) | -18.268(-62.934,90.430) | -0.550(-0.970,-0.129) | 0.099(-0.044, 0.241) |
| Niue | 0.014(0.002,0.046) | 0.009(0.002,0.028) | -34.317(-73.867,72.957) | -1.655(-1.790,-1.519) | -1.313(-1.478, -1.148) |
| Northern Mariana Islands | 0.039(0.017,0.080) | 0.029(0.014,0.055) | -26.283(-56.163,33.635) | -0.857(-1.164,-0.548) | -1.383(-1.869, -0.897) |
| Palau | 0.004(0.000,0.012) | 0.003(0.000,0.009) | -24.381(-69.883,111.227) | -1.116(-1.246,-0.985) | -0.896(-1.038, -0.754) |
| Puerto Rico | 0.000(0.000,0.000) | 0.000(0.000,0.000) | 506.413(205.660,775.952) | 6.368(3.180,9.654) | 9.975(7.627, 12.323) |
| Saint Kitts and Nevis | 0.022(0.017,0.027) | 0.015(0.011,0.019) | -31.754(-47.702,-11.047) | -0.746(-0.962,-0.530) | -1.229(-1.272, -1.186) |
| San Marino | 0.014(0.005,0.030) | 0.000(0.000,0.000) | -99.099(-99.674,-97.808) | -19.009(-21.302,-16.649) | -12.333(-13.742, -10.924) |
| Tokelau | 0.019(0.002,0.065) | 0.010(0.002,0.033) | -45.216(-74.795,47.049) | -2.324(-2.500,-2.148) | -1.871(-2.112, -1.63) |
| Tuvalu | 0.066(0.019,0.186) | 0.026(0.011,0.059) | -59.998(-84.235,9.897) | -2.596(-2.736,-2.455) | -2.923(-2.967, -2.88) |
| United States Virgin Islands | 0.001(0.001,0.003) | 0.001(0.000,0.001) | -48.672(-77.407,-2.754) | -1.542(-1.998,-1.084) | -2.03(-2.284, -1.776) |
| South Sudan | 0.304(0.064,0.728) | 0.317(0.065,0.764) | 4.162(-44.591,83.399) | 0.026(-0.237,0.290) | 0.149(-0.032, 0.33) |
| Sudan | 0.002(0.001,0.005) | 0.001(0.000,0.002) | -52.889(-77.465,12.798) | -2.387(-2.635,-2.138) | -2.38(-2.453, -2.307) |

Abbreviations: ASMR, Age standardized mortality rate; AAPC, average annual percent change; CI, confidence interval; EAPC, estimated annual percentage change; UI, uncertainty interval.

Supplementary Table 10: Trends in the number of rabies mortality cases in 204 countries and territories worldwide from 1990 to 2021.

| Location | Rabies. Death cases (persons) . (95% UI).  1990 year. | Rabies. Death cases (persons) . (95% UI).  2021 year. | Rabies. Percentage change  (95% UI). 1990－2021. | Rabies. EAPC (95% CI). 1990－2021. | Rabies. AAPC (95% CI). 1990－2021. |
| --- | --- | --- | --- | --- | --- |
| China | 1081(634,1493) | 604(317,912) | -44.170(-62.461,-19.780) | -0.241(-2.327,1.889) | -0.607(-1.624, 0.41) |
| Democratic People's Republic of Korea | 10(4,19) | 16(7,28) | 57.996(-5.661,172.993) | 0.577(0.169,0.986) | 1.518(1.38, 1.656) |
| Taiwan (Province of China) | 0(0,0) | 0(0,0) | 17.145(-22.060,65.238) | 2.031(-0.090,4.197) | 0.37(-2.998, 3.737) |
| Cambodia | 50(18,87) | 26(8,50) | -48.541(-74.187,-12.057) | -4.789(-5.293,-4.283) | -1.989(-2.201, -1.778) |
| Indonesia | 132(79,199) | 48(26,81) | -63.438(-82.990,-27.744) | -4.459(-4.691,-4.227) | -3.178(-3.398, -2.958) |
| Lao People's Democratic Republic | 23(10,40) | 12(4,21) | -48.051(-74.193,2.306) | -4.382(-4.839,-3.922) | -2.059(-2.245, -1.874) |
| Malaysia | 1(0,1) | 0(0,1) | -46.175(-70.762,14.442) | -3.849(-4.121,-3.576) | -2.038(-2.617, -1.459) |
| Maldives | 0(0,0) | 0(0,0) | -44.947(-76.635,30.303) | -4.180(-4.599,-3.759) | -1.784(-2.553, -1.015) |
| Myanmar | 534(314,910) | 236(109,420) | -55.766(-80.751,-10.538) | -3.948(-4.136,-3.759) | -2.618(-2.838, -2.399) |
| Philippines | 505(397,620) | 250(150,351) | -50.557(-65.030,-34.745) | -5.048(-5.656,-4.437) | -2.109(-2.305, -1.914) |
| Sri Lanka | 87(45,139) | 21(8,39) | -75.449(-88.416,-53.484) | -7.320(-8.218,-6.413) | -4.173(-4.873, -3.474) |
| Thailand | 19(8,35) | 5(2,8) | -76.034(-87.180,-52.965) | -6.379(-10.067,-2.541) | 0.453(-0.863, 1.768) |
| Timor-Leste | 3(1,6) | 2(1,4) | -28.074(-61.666,37.115) | -3.377(-3.644,-3.109) | -1.121(-1.346, -0.896) |
| Viet Nam | 100(48,164) | 56(28,95) | -44.281(-68.095,11.068) | -3.270(-3.378,-3.163) | -1.886(-1.985, -1.786) |
| Fiji | 0(0,0) | 0(0,0) | 120.142(-16.192,473.268) | 2.925(2.226,3.628) | 2.821(2.216, 3.427) |
| Kiribati | 0(0,0) | 0(0,0) | 40.458(-42.557,250.188) | -0.525(-0.663,-0.387) | 1.139(0.998, 1.279) |
| Marshall Islands | 0(0,0) | 0(0,0) | -7.827(-62.163,118.914) | -1.331(-1.524,-1.137) | -0.37(-0.717, -0.022) |
| Micronesia (Federated States of) | 0(0,0) | 0(0,0) | -38.980(-72.687,36.592) | -1.398(-1.511,-1.284) | -1.576(-1.722, -1.431) |
| Papua New Guinea | 0(0,2) | 1(0,4) | 161.598(10.447,534.306) | -0.175(-0.370,0.019) | 3.234(3.097, 3.37) |
| Samoa | 0(0,0) | 0(0,0) | 28.019(-38.763,184.831) | 0.302(0.102,0.502) | 0.766(0.638, 0.893) |
| Solomon Islands | 0(0,0) | 0(0,0) | 136.631(7.475,690.282) | 0.560(0.316,0.804) | 2.761(2.505, 3.016) |
| Tonga | 0(0,0) | 0(0,0) | 14.971(-49.790,190.012) | 0.363(0.117,0.609) | 0.499(0.179, 0.819) |
| Vanuatu | 0(0,0) | 0(0,0) | 115.927(-3.054,440.006) | 0.031(-0.088,0.150) | 2.47(2.382, 2.559) |
| Armenia | 0(0,0) | 0(0,0) | -79.678(-88.329,-63.694) | -5.096(-8.367,-1.707) | 19.739(-57.046, 96.523) |
| Azerbaijan | 3(1,7) | 1(0,4) | -45.158(-84.418,102.609) | -4.778(-5.836,-3.708) | -1.505(-2.345, -0.665) |
| Georgia | 1(1,2) | 1(0,1) | -41.230(-72.219,28.002) | -1.674(-4.879,1.638) | 7.349(0.255, 14.442) |
| Kazakhstan | 2(1,3) | 3(2,6) | 79.015(-13.725,277.684) | 1.339(-1.098,3.836) | 8.781(0.997, 16.564) |
| Kyrgyzstan | 1(1,2) | 1(0,1) | -62.393(-85.967,-10.082) | -6.934(-9.657,-4.130) | 1.354(-4.389, 7.097) |
| Mongolia | 4(0,15) | 2(0,5) | -64.596(-93.360,74.786) | -5.972(-6.446,-5.496) | -2.771(-3.912, -1.63) |
| Tajikistan | 0(0,0) | 0(0,0) | -24.043(-80.969,184.023) | -3.683(-4.056,-3.308) | -0.629(-1.191, -0.067) |
| Turkmenistan | 0(0,0) | 0(0,0) | -58.847(-83.046,2.815) | -0.733(-8.476,7.664) | 94.64(12.566, 176.714) |
| Uzbekistan | 2(1,3) | 1(1,2) | -2.323(-48.774,86.395) | -3.794(-4.795,-2.783) | 0.704(-2.192, 3.6) |
| Albania | 0(0,0) | 0(0,0) | -82.246(-94.303,-37.363) | -7.500(-9.134,-5.836) | -3.978(-5.166, -2.79) |
| Bosnia and Herzegovina | 0(0,0) | 0(0,0) | -79.156(-94.793,-30.900) | -3.293(-4.180,-2.398) | -4.356(-4.79, -3.922) |
| Bulgaria | 0(0,0) | 0(0,0) | 272.936(130.906,479.429) | 5.878(4.519,7.255) | 4.387(-0.855, 9.629) |
| Croatia | 0(0,0) | 0(0,0) | -96.249(-97.816,-93.600) | -12.231(-14.996,-9.377) | 0.084(-5.356, 5.525) |
| Czechia | 0(0,0) | 0(0,0) | -55.964(-71.489,-30.716) | -3.417(-6.627,-0.096) | -3.52(-7.476, 0.436) |
| Hungary | 0(0,0) | 0(0,0) | 69.826(19.960,149.587) | -4.953(-8.002,-1.803) | 2.99(-2.668, 8.648) |
| North Macedonia | 0(0,0) | 0(0,0) | -42.907(-77.672,38.482) | -4.191(-5.470,-2.895) | -0.882(-1.976, 0.212) |
| Montenegro | 0(0,0) | 0(0,0) | -19.895(-69.001,84.485) | -0.947(-1.241,-0.653) | -0.428(-1.079, 0.223) |
| Poland | 1(1,1) | 0(0,0) | -99.096(-99.279,-98.684) | -17.199(-19.796,-14.519) | -12.615(-18.446, -6.785) |
| Romania | 3(2,3) | 0(0,0) | -99.734(-99.811,-99.633) | -16.508(-19.933,-12.937) | 4.284(-48.533, 57.101) |
| Serbia | 0(0,0) | 0(0,0) | -64.375(-83.194,-17.680) | -3.282(-3.783,-2.778) | -2.812(-3.541, -2.083) |
| Slovakia | 0(0,0) | 0(0,0) | -61.678(-80.703,-16.948) | -3.011(-3.101,-2.922) | -3.006(-3.226, -2.787) |
| Slovenia | 0(0,0) | 0(0,0) | 64.680(21.057,137.203) | -2.119(-5.161,1.021) | 8.081(-0.534, 16.695) |
| Belarus | 0(0,0) | 2(1,2) | 505.001(259.094,807.904) | 6.134(3.777,8.544) | 8.925(6.363, 11.486) |
| Estonia | 0(0,0) | 0(0,0) | 208.954(90.232,393.070) | 5.819(2.126,9.646) | 5.938(0.45, 11.426) |
| Latvia | 0(0,1) | 0(0,0) | -79.834(-87.087,-68.875) | -4.496(-6.726,-2.213) | -1.709(-10.487, 7.068) |
| Lithuania | 0(0,0) | 0(0,0) | -98.569(-99.256,-97.334) | -12.205(-16.149,-8.075) | -7.505(-20.644, 5.633) |
| Republic of Moldova | 0(0,0) | 0(0,0) | -84.102(-90.608,-73.414) | -4.813(-6.112,-3.496) | -3.145(-7.133, 0.844) |
| Russian Federation | 10(9,10) | 2(2,2) | -77.328(-79.672,-74.743) | -3.614(-5.270,-1.928) | 1.82(-4.557, 8.197) |
| Ukraine | 1(1,2) | 1(1,2) | -12.952(-57.691,87.286) | 0.628(-0.858,2.135) | -0.258(-1.508, 0.993) |
| Brunei Darussalam | 0(0,0) | 0(0,0) | -9.661(-45.382,54.908) | -1.286(-1.992,-0.574) | -0.344(-10.059, 9.372) |
| Japan | 1(1,1) | 0(0,0) | -59.063(-67.346,-52.927) | -1.577(-3.321,0.198) | -5.403(-6.876, -3.931) |
| Republic of Korea | 0(0,0) | 0(0,0) | -93.110(-96.820,-65.911) | -10.395(-12.548,-8.190) | 14.646(-4.802, 34.094) |
| Singapore | 0(0,0) | 0(0,0) | 117.975(39.090,209.286) | -1.709(-3.476,0.091) | -1.381(-4.179, 1.417) |
| Australia | 0(0,0) | 0(0,0) | 271.660(125.061,495.959) | 9.831(5.738,14.083) | 1.502(1.018, 1.986) |
| New Zealand | 0(0,0) | 0(0,0) | -36.080(-56.825,-6.044) | -0.989(-2.780,0.835) | 4.503(-1.351, 10.356) |
| Andorra | 0(0,0) | 0(0,0) | 57.095(-22.591,205.209) | -0.095(-0.457,0.269) | -0.811(-5.244, 3.622) |
| Austria | 0(0,0) | 0(0,0) | 24.736(-9.516,68.361) | -1.292(-3.876,1.361) | -1.044(-2.385, 0.297) |
| Belgium | 0(0,0) | 0(0,0) | -40.283(-58.065,-16.504) | 2.059(-0.464,4.645) | 0.819(-13.352, 14.99) |
| Cyprus | 0(0,0) | 0(0,0) | -33.031(-71.867,84.570) | -2.182(-3.430,-0.919) | -0.577(-3.134, 1.979) |
| Denmark | 0(0,0) | 0(0,0) | -77.969(-83.440,-70.846) | -5.498(-7.982,-2.947) | 17.744(10.914, 24.574) |
| Finland | 0(0,0) | 0(0,0) | -16.621(-50.148,31.364) | -0.993(-1.450,-0.534) | 8.721(-0.834, 18.275) |
| France | 0(0,0) | 0(0,0) | 50.277(-1.292,124.268) | -1.768(-3.866,0.376) | 4.269(-2.473, 11.011) |
| Germany | 0(0,0) | 0(0,0) | 148.998(57.403,268.663) | 3.010(1.440,4.604) | 3.12(0.499, 5.741) |
| Greece | 0(0,0) | 1(0,1) | 3247.586(2144.899,5033.197) | 18.219(13.937,22.661) | 21.428(3.317, 39.538) |
| Iceland | 0(0,0) | 0(0,0) | 56.611(7.805,122.593) | -0.233(-4.275,3.980) | 9.572(6.018, 13.126) |
| Ireland | 0(0,0) | 0(0,0) | 19.375(-14.258,71.532) | 2.061(0.059,4.104) | 9.326(1.674, 16.978) |
| Israel | 0(0,0) | 0(0,0) | -78.304(-85.690,-67.143) | -6.634(-9.679,-3.487) | 1.299(-3.961, 6.559) |
| Italy | 0(0,0) | 0(0,0) | -76.078(-79.904,-71.387) | -5.056(-6.271,-3.825) | 7.193(-6.017, 20.404) |
| Luxembourg | 0(0,0) | 0(0,0) | 5.712(-23.808,40.896) | -0.873(-4.734,3.144) | 16.07(-0.254, 32.394) |
| Malta | 0(0,0) | 0(0,0) | -95.087(-96.359,-93.441) | -8.446(-11.092,-5.722) | 11.239(3.204, 19.275) |
| Netherlands | 0(0,0) | 0(0,0) | 353.457(220.143,523.495) | 5.218(2.133,8.396) | 2.577(-4.248, 9.401) |
| Norway | 0(0,0) | 1(1,1) | 942.083(753.429,1130.584) | 4.997(3.354,6.666) | 8.001(-1.25, 17.252) |
| Portugal | 0(0,0) | 0(0,0) | -80.734(-86.688,-70.809) | -3.798(-7.096,-0.382) | -3.273(-3.811, -2.736) |
| Spain | 0(0,0) | 0(0,0) | 15.283(-16.784,56.500) | 1.643(0.186,3.122) | 10.758(5.682, 15.835) |
| Sweden | 0(0,0) | 0(0,0) | 192.519(104.942,297.676) | 1.496(-1.135,4.196) | 0.067(-0.488, 0.621) |
| Switzerland | 0(0,0) | 0(0,0) | 919.128(557.880,1403.457) | 5.334(1.427,9.392) | 2.255(1.868, 2.641) |
| United Kingdom | 0(0,0) | 0(0,0) | 735.805(666.639,809.849) | 7.163(5.334,9.024) | 8.236(1.208, 15.264) |
| Argentina | 0(0,0) | 0(0,0) | 46.855(1.203,102.773) | -1.146(-3.611,1.382) | -1.427(-4.311, 1.457) |
| Chile | 0(0,0) | 0(0,0) | 230.525(135.059,365.031) | 1.915(-0.549,4.439) | -0.973(-1.253, -0.692) |
| Uruguay | 0(0,0) | 0(0,0) | -64.709(-75.768,-41.763) | -3.570(-3.897,-3.241) | 1.94(1.491, 2.39) |
| Canada | 0(0,0) | 0(0,0) | 2177.752(1406.151,3247.914) | 9.346(6.493,12.276) | -1.166(-1.683, -0.648) |
| United States of America | 2(2,2) | 6(6,7) | 297.120(253.086,338.784) | 3.062(2.356,3.772) | -0.444(-0.912, 0.023) |
| Antigua and Barbuda | 0(0,0) | 0(0,0) | -0.080(-34.356,53.011) | -1.205(-1.538,-0.871) | 0.288(0.037, 0.538) |
| Bahamas | 0(0,0) | 0(0,0) | 93.157(17.173,207.607) | 0.611(0.365,0.859) | 93.704(32.524, 154.884) |
| Barbados | 0(0,0) | 0(0,0) | 348.826(199.016,536.531) | 4.733(2.873,6.626) | 22.998(2.748, 43.248) |
| Belize | 0(0,0) | 0(0,0) | -42.871(-61.614,-15.860) | -3.086(-4.325,-1.832) | 20.438(-21.124, 62) |
| Cuba | 0(0,0) | 0(0,0) | 10.860(-27.525,89.721) | 0.537(-2.906,4.103) | 11.55(9.551, 13.548) |
| Dominica | 0(0,0) | 0(0,0) | -26.472(-57.022,22.973) | -0.736(-1.011,-0.461) | 39.129(26.263, 51.994) |
| Dominican Republic | 0(0,0) | 0(0,1) | 69.177(-32.225,296.566) | 1.122(0.447,1.801) | -9.475(-9.811, -9.14) |
| Grenada | 0(0,0) | 0(0,0) | -32.448(-53.153,-3.637) | -0.915(-1.279,-0.549) | -10.428(-20.721, -0.136) |
| Guyana | 0(0,0) | 0(0,0) | -19.232(-55.180,37.975) | 0.596(0.096,1.098) | -10.352(-11.629, -9.074) |
| Haiti | 0(0,0) | 0(0,0) | 5.253(-46.292,97.066) | -2.185(-2.290,-2.081) | -11.901(-19.205, -4.598) |
| Jamaica | 0(0,0) | 0(0,0) | 8107.165(4876.734,12027.410) | 11.672(4.990,18.778) | 1.218(-0.157, 2.594) |
| Saint Lucia | 0(0,0) | 0(0,0) | 221.081(50.507,1054.039) | 4.446(-1.376,10.613) | -15.329(-17.799, -12.858) |
| Saint Vincent and the Grenadines | 0(0,0) | 0(0,0) | -92.658(-95.231,-86.142) | 8.272(-0.095,17.340) | -12.637(-24.531, -0.742) |
| Suriname | 0(0,0) | 0(0,0) | 883.663(284.615,2385.701) | 3.719(0.611,6.922) | -1.632(-1.776, -1.488) |
| Trinidad and Tobago | 0(0,0) | 0(0,0) | 5892.917(3582.456,9375.821) | 9.769(4.388,15.427) | -4.296(-6.577, -2.016) |
| Bolivia (Plurinational State of) | 1(0,2) | 0(0,0) | -95.827(-98.303,-89.580) | -12.249(-13.361,-11.123) | -2.597(-2.925, -2.269) |
| Ecuador | 14(12,16) | 0(0,0) | -99.972(-99.981,-99.957) | -29.783(-33.993,-25.304) | -0.287(-7.806, 7.232) |
| Peru | 2(1,3) | 0(0,0) | -98.067(-99.442,-85.215) | -12.368(-14.433,-10.253) | -9.345(-10.811, -7.878) |
| Colombia | 2(2,3) | 0(0,0) | -99.474(-99.618,-99.255) | -12.666(-16.901,-8.215) | 0.403(0.268, 0.537) |
| Costa Rica | 0(0,0) | 0(0,0) | 23.103(-59.735,268.172) | -0.887(-1.665,-0.102) | 0.394(0.237, 0.552) |
| El Salvador | 1(0,2) | 0(0,0) | -99.649(-99.848,-96.204) | -20.483(-22.584,-18.325) | -7.4(-7.922, -6.877) |
| Guatemala | 7(6,9) | 0(0,0) | -99.717(-99.808,-99.607) | -18.816(-23.523,-13.820) | -0.083(-0.743, 0.578) |
| Honduras | 0(0,0) | 0(0,0) | -41.251(-70.452,6.936) | -4.383(-4.616,-4.149) | -1.35(-1.706, -0.994) |
| Mexico | 54(49,58) | 0(0,0) | -99.899(-99.917,-99.878) | -21.074(-22.438,-19.687) | 2.375(1.999, 2.751) |
| Nicaragua | 0(0,0) | 0(0,0) | -84.854(-93.256,-68.479) | -9.703(-11.542,-7.826) | 8.288(0.39, 16.187) |
| Panama | 0(0,0) | 0(0,0) | -57.730(-74.222,-27.180) | -3.453(-3.894,-3.010) | 0.613(0.345, 0.882) |
| Venezuela (Bolivarian Republic of) | 0(0,0) | 0(0,0) | -95.821(-97.306,-93.777) | -13.350(-17.424,-9.074) | 2.409(2.121, 2.696) |
| Brazil | 77(54,104) | 0(0,0) | -99.480(-99.635,-99.253) | -16.101(-17.924,-14.238) | -0.318(-0.457, -0.179) |
| Paraguay | 0(0,0) | 0(0,0) | -99.021(-99.648,-80.523) | -21.874(-25.081,-18.531) | -1.019(-1.38, -0.658) |
| Algeria | 0(0,0) | 0(0,0) | 13.367(-35.456,142.684) | -1.270(-1.437,-1.103) | -0.967(-1.359, -0.575) |
| Bahrain | 0(0,0) | 0(0,0) | 12.496(-40.041,118.995) | -3.596(-3.846,-3.345) | 9.77(6.383, 13.158) |
| Egypt | 81(42,122) | 8(4,13) | -90.481(-94.329,-82.149) | -9.304(-9.943,-8.662) | 0.051(-0.924, 1.026) |
| Iran (Islamic Republic of) | 1(0,2) | 1(0,2) | -11.934(-50.395,75.547) | 0.487(-0.638,1.624) | 3.138(1.723, 4.553) |
| Iraq | 4(1,8) | 2(1,5) | -38.519(-78.147,69.604) | -2.915(-3.561,-2.264) | -0.058(-0.214, 0.097) |
| Jordan | 0(0,0) | 0(0,0) | 104.816(13.122,292.260) | -1.116(-1.514,-0.718) | -3.539(-3.85, -3.229) |
| Kuwait | 0(0,0) | 0(0,0) | 213.698(13.170,757.445) | -2.603(-4.341,-0.833) | 0.711(0.105, 1.317) |
| Lebanon | 0(0,0) | 0(0,0) | 19.467(-40.455,143.324) | -0.900(-1.153,-0.646) | 2.465(2.32, 2.611) |
| Libya | 0(0,0) | 0(0,0) | 105.035(-5.714,383.596) | 1.058(0.581,1.537) | 2.397(2.099, 2.695) |
| Morocco | 0(0,1) | 0(0,1) | -9.287(-51.280,94.195) | -1.036(-1.275,-0.797) | -7.448(-7.973, -6.923) |
| Palestine | 0(0,0) | 0(0,0) | -27.623(-71.094,171.620) | -3.560(-3.761,-3.358) | -5.139(-5.533, -4.745) |
| Oman | 0(0,0) | 0(0,0) | -25.787(-69.888,141.179) | -3.182(-3.465,-2.898) | -3.555(-3.877, -3.233) |
| Qatar | 0(0,0) | 0(0,0) | 578.763(134.516,1604.872) | -1.624(-3.913,0.720) | -2.523(-2.698, -2.349) |
| Saudi Arabia | 0(0,0) | 0(0,0) | -24.776(-66.405,85.957) | -4.614(-5.503,-3.717) | -2.488(-2.696, -2.281) |
| Syrian Arab Republic | 0(0,0) | 0(0,1) | 56.136(-43.850,284.537) | 3.093(1.635,4.572) | 0.153(-0.116, 0.422) |
| Tunisia | 0(0,0) | 0(0,0) | -0.961(-52.014,163.406) | -1.003(-1.110,-0.895) | 1.044(0.805, 1.282) |
| Türkiye | 5(2,13) | 2(1,4) | -67.258(-83.376,-28.656) | -4.549(-4.735,-4.363) | 0.116(-0.399, 0.63) |
| United Arab Emirates | 0(0,1) | 1(0,1) | 26.559(-48.609,345.323) | -4.273(-4.795,-3.749) | 1.186(0.878, 1.494) |
| Yemen | 0(0,0) | 0(0,1) | 114.911(-6.446,511.970) | -0.555(-0.826,-0.283) | -0.454(-0.971, 0.062) |
| Afghanistan | 0(0,1) | 0(0,1) | 100.032(10.037,453.098) | -1.673(-2.133,-1.212) | -1.321(-1.575, -1.068) |
| Bangladesh | 282(72,560) | 22(9,36) | -92.123(-96.423,-78.522) | -11.768(-13.046,-10.471) | 1.051(0.82, 1.282) |
| Bhutan | 7(1,15) | 1(0,3) | -80.337(-89.362,-47.457) | -6.407(-6.835,-5.976) | -0.476(-1.664, 0.712) |
| India | 12567(9298,15681) | 4023(2530,5677) | -67.984(-75.994,-56.139) | -5.797(-6.049,-5.544) | 2.773(2.401, 3.145) |
| Nepal | 1054(514,1777) | 479(261,744) | -54.569(-74.590,-17.180) | -4.240(-4.380,-4.100) | 0.101(-0.111, 0.314) |
| Pakistan | 1349(711,2282) | 614(378,914) | -54.516(-72.077,-20.908) | -5.369(-5.631,-5.106) | -1.654(-1.787, -1.522) |
| Angola | 4(1,12) | 4(1,13) | 2.301(-60.603,110.692) | -3.539(-3.768,-3.310) | 0.387(0.155, 0.619) |
| Central African Republic | 1(0,3) | 1(0,4) | 39.138(-30.310,178.930) | -1.230(-1.376,-1.084) | 1.907(1.651, 2.164) |
| Congo | 0(0,1) | 0(0,1) | -9.738(-63.094,63.722) | -2.790(-2.981,-2.599) | 0.188(-0.053, 0.429) |
| Democratic Republic of the Congo | 10(2,32) | 15(3,44) | 45.702(-32.265,186.221) | -1.292(-1.569,-1.014) | -12.079(-14.723, -9.435) |
| Equatorial Guinea | 0(0,0) | 0(0,0) | -16.191(-70.204,275.409) | -5.654(-5.986,-5.320) | 0.303(0.094, 0.512) |
| Gabon | 0(0,0) | 0(0,0) | -36.033(-71.306,25.608) | -3.046(-3.169,-2.923) | -1.507(-2.245, -0.77) |
| Burundi | 29(13,53) | 39(9,120) | 36.805(-56.758,205.814) | -1.815(-2.100,-1.528) | 5.341(3.65, 7.032) |
| Comoros | 2(1,4) | 1(0,4) | -16.357(-59.342,60.784) | -2.229(-2.649,-1.808) | 3.34(2.993, 3.687) |
| Djibouti | 1(0,2) | 2(1,5) | 129.019(21.303,377.486) | -0.876(-1.323,-0.427) | 0.185(-0.015, 0.386) |
| Eritrea | 18(10,31) | 18(8,35) | 4.049(-45.558,95.151) | -1.801(-1.946,-1.657) | 1.38(0.997, 1.763) |
| Ethiopia | 1749(531,4308) | 1043(313,2905) | -40.379(-72.286,11.602) | -4.438(-4.670,-4.205) | 0.047(-0.239, 0.333) |
| Kenya | 79(34,154) | 88(38,156) | 11.333(-32.308,83.163) | -1.402(-1.794,-1.010) | -0.896(-1.156, -0.635) |
| Madagascar | 30(14,50) | 53(23,97) | 77.528(-13.083,209.802) | -0.703(-1.053,-0.352) | -0.752(-0.938, -0.565) |
| Malawi | 78(40,136) | 81(34,143) | 3.765(-50.849,128.225) | -2.148(-2.457,-1.839) | -0.017(-0.299, 0.266) |
| Mauritius | 0(0,0) | 0(0,0) | -99.160(-99.371,-98.795) | -10.052(-12.793,-7.224) | -1.159(-1.573, -0.745) |
| Mozambique | 114(60,191) | 123(48,236) | 7.816(-52.853,98.509) | -1.981(-2.183,-1.777) | -0.738(-0.891, -0.585) |
| Rwanda | 43(19,98) | 28(5,93) | -35.027(-80.317,43.891) | -4.158(-4.596,-3.718) | 1.787(1.091, 2.482) |
| Seychelles | 0(0,0) | 0(0,0) | 221.175(71.651,440.226) | 2.164(0.251,4.113) | 1.77(1.418, 2.122) |
| Somalia | 40(7,104) | 108(20,318) | 172.160(12.932,435.548) | 0.002(-0.232,0.237) | 0.667(0.448, 0.885) |
| United Republic of Tanzania | 148(70,261) | 156(52,338) | 5.231(-61.534,120.668) | -2.289(-2.539,-2.038) | 2.028(1.859, 2.197) |
| Uganda | 49(18,105) | 77(26,151) | 55.700(-27.766,268.521) | -1.893(-2.209,-1.576) | -1.262(-1.581, -0.942) |
| Zambia | 53(31,87) | 52(22,93) | -2.430(-55.222,109.868) | -3.029(-3.509,-2.546) | 2.492(2.257, 2.727) |
| Botswana | 1(0,2) | 1(0,1) | -25.140(-67.662,69.493) | -2.698(-2.843,-2.552) | 1.349(0.881, 1.816) |
| Lesotho | 1(1,3) | 1(1,2) | -21.387(-65.889,81.294) | -0.761(-1.144,-0.377) | 1.516(0.724, 2.308) |
| Namibia | 1(0,2) | 1(0,2) | -2.484(-53.648,101.241) | -1.835(-1.946,-1.724) | 0.672(0.414, 0.93) |
| South Africa | 16(8,26) | 11(6,16) | -32.140(-58.891,9.867) | -2.777(-3.487,-2.061) | 0.451(0.124, 0.779) |
| Eswatini | 1(0,1) | 1(0,1) | -22.869(-64.582,63.129) | -1.662(-1.787,-1.537) | 0.089(-0.376, 0.555) |
| Zimbabwe | 9(4,15) | 15(8,30) | 70.964(-18.819,231.588) | 1.016(0.497,1.538) | 0.455(-0.251, 1.161) |
| Benin | 21(6,53) | 37(11,81) | 73.849(-27.010,345.377) | -1.315(-1.462,-1.167) | 1.324(1.005, 1.643) |
| Burkina Faso | 60(18,128) | 74(23,165) | 22.142(-45.196,160.445) | -2.335(-2.565,-2.104) | -0.599(-0.831, -0.367) |
| Cameroon | 34(10,70) | 63(24,125) | 86.255(-14.725,340.295) | -1.231(-1.462,-0.999) | 1.857(1.258, 2.456) |
| Cabo Verde | 1(0,2) | 0(0,1) | -34.142(-65.931,67.519) | -3.275(-3.549,-3.001) | 0.979(0.822, 1.136) |
| Chad | 34(6,89) | 72(11,195) | 110.585(-1.922,343.429) | -0.929(-1.043,-0.816) | -1.282(-2.367, -0.198) |
| Côte d'Ivoire | 40(10,90) | 61(17,127) | 51.066(-24.065,220.638) | -0.874(-1.140,-0.607) | -0.612(-1.224, 0) |
| Gambia | 3(1,8) | 5(2,12) | 56.587(-26.063,265.277) | -1.478(-1.752,-1.203) | 0.112(-0.358, 0.581) |
| Ghana | 104(47,188) | 126(55,218) | 20.431(-34.703,124.038) | -1.688(-1.977,-1.399) | 0.83(0.613, 1.047) |
| Guinea | 37(12,82) | 42(11,89) | 12.450(-51.409,119.808) | -1.679(-1.885,-1.473) | 0.609(0.347, 0.871) |
| Guinea-Bissau | 6(2,13) | 6(2,13) | 1.749(-50.365,100.805) | -1.751(-1.961,-1.541) | 2.054(1.441, 2.666) |
| Liberia | 9(2,20) | 10(2,24) | 11.169(-57.932,120.730) | -2.629(-2.929,-2.328) | -0.695(-1.189, -0.201) |
| Mali | 53(9,145) | 76(15,193) | 41.446(-34.284,186.544) | -1.988(-2.241,-1.735) | 2.23(0.926, 3.535) |
| Mauritania | 6(2,13) | 5(2,11) | -15.240(-58.127,61.782) | -2.757(-2.954,-2.560) | 0.005(-0.434, 0.445) |
| Niger | 65(13,177) | 117(18,341) | 79.665(-44.496,317.079) | -1.819(-2.036,-1.601) | -1.64(-2.425, -0.854) |
| Nigeria | 671(358,1061) | 909(382,1544) | 35.454(-38.378,154.844) | -1.810(-2.109,-1.511) | 0.892(0.557, 1.227) |
| Sao Tome and Principe | 0(0,1) | 0(0,0) | -33.731(-67.391,56.691) | -3.346(-3.589,-3.103) | -2.221(-2.375, -2.068) |
| Senegal | 40(13,86) | 35(11,74) | -14.039(-58.734,68.196) | -2.453(-2.678,-2.228) | 1.186(0.747, 1.625) |
| Sierra Leone | 19(7,42) | 20(5,50) | 3.131(-59.096,125.020) | -2.293(-2.591,-1.994) | 1.043(0.801, 1.285) |
| Togo | 15(6,31) | 19(7,39) | 28.404(-38.457,157.223) | -1.601(-1.739,-1.462) | 11.296(5.218, 17.375) |
| American Samoa | 0(0,0) | 0(0,0) | 12.597(-46.229,172.202) | 0.608(0.442,0.774) | -0.618(-1.08, -0.156) |
| Bermuda | 0(0,0) | 0(0,0) | 88.619(14.087,204.223) | 2.346(1.722,2.974) | -8.72(-10.202, -7.239) |
| Cook Islands | 0(0,0) | 0(0,0) | -21.692(-69.804,187.248) | -0.730(-1.024,-0.435) | -1.028(-1.308, -0.747) |
| Greenland | 0(0,0) | 0(0,0) | 60.544(-25.536,227.419) | 2.904(1.964,3.851) | -0.763(-0.848, -0.677) |
| Guam | 0(0,0) | 0(0,0) | 2.056(-39.189,69.830) | 0.403(0.037,0.771) | -0.389(-0.753, -0.026) |
| Monaco | 0(0,0) | 0(0,0) | -45.710(-75.927,15.147) | -2.000(-3.132,-0.854) | 2.073(1.473, 2.672) |
| Nauru | 0(0,0) | 0(0,0) | 32.844(-42.487,207.620) | 0.495(-0.098,1.092) | 0.099(-0.044, 0.241) |
| Niue | 0(0,0) | 0(0,0) | -51.245(-80.680,30.330) | -1.020(-1.217,-0.822) | -1.313(-1.478, -1.148) |
| Northern Mariana Islands | 0(0,0) | 0(0,0) | 40.325(-25.276,169.646) | 1.101(0.652,1.553) | -1.383(-1.869, -0.897) |
| Palau | 0(0,0) | 0(0,0) | 33.457(-46.182,237.737) | 0.158(-0.006,0.322) | -0.896(-1.038, -0.754) |
| Puerto Rico | 0(0,0) | 0(0,0) | 1228.146(586.406,1803.750) | 9.339(6.256,12.511) | 9.975(7.627, 12.323) |
| Saint Kitts and Nevis | 0(0,0) | 0(0,0) | -19.151(-38.181,7.992) | -1.502(-1.787,-1.216) | -1.229(-1.272, -1.186) |
| San Marino | 0(0,0) | 0(0,0) | -97.465(-99.056,-93.571) | -17.429(-20.013,-14.761) | -12.333(-13.742, -10.924) |
| Tokelau | 0(0,0) | 0(0,0) | -31.545(-70.513,88.643) | -0.765(-1.196,-0.332) | -1.871(-2.112, -1.63) |
| Tuvalu | 0(0,0) | 0(0,0) | -20.820(-70.072,128.512) | -1.693(-1.889,-1.496) | -2.923(-2.967, -2.88) |
| United States Virgin Islands | 0(0,0) | 0(0,0) | -14.436(-57.244,81.485) | 0.418(0.032,0.806) | -2.03(-2.284, -1.776) |
| South Sudan | 17(4,38) | 30(6,72) | 78.541(-17.811,247.896) | 0.217(-0.149,0.584) | 0.149(-0.032, 0.33) |
| Sudan | 0(0,1) | 0(0,1) | 3.934(-51.217,140.403) | -2.282(-2.527,-2.036) | -2.38(-2.453, -2.307) |

Abbreviations: AAPC, average annual percent change; CI, confidence interval; EAPC, estimated annual percentage change; UI, uncertainty interval.

Supplementary Table 11: Trends in the ASDR of rabies in 204 countries and territories worldwide from 1990 to 2021.

| Location | ASDR (per 100,000 population)  (95% UI).  1990 year | ASDR (per 100,000 population)  (95% UI).  2021 year | Percentage change  (95% UI). 1990－2021. | EAPC (95% CI). 1990－2021. | AAPC (95% CI). 1990－2021. |
| --- | --- | --- | --- | --- | --- |
| China | 6.149(3.593,8.683) | 1.819(0.974,2.795) | -70.426(-80.388,-57.771) | -1.784(-3.946,0.427) | -2.888(-3.826, -1.951) |
| Democratic People's Republic of Korea | 2.823(1.073,5.380) | 2.833(1.138,5.380) | 0.368(-43.314,68.718) | 0.062(-0.382,0.508) | 0.039(-0.102, 0.18) |
| Taiwan (Province of China) | 0.011(0.009,0.014) | 0.003(0.002,0.004) | -74.435(-81.768,-65.697) | -2.386(-4.595,-0.127) | -4.458(-9.345, 0.428) |
| Cambodia | 28.222(10.133,48.409) | 8.228(2.355,16.571) | -70.846(-84.844,-51.449) | -4.919(-5.422,-4.412) | -3.77(-3.986, -3.554) |
| Indonesia | 4.560(2.567,6.929) | 1.240(0.667,2.327) | -72.816(-88.028,-40.084) | -4.130(-4.341,-3.918) | -4.099(-4.297, -3.901) |
| Lao People's Democratic Republic | 31.214(13.245,54.038) | 9.200(3.356,17.000) | -70.526(-85.406,-41.621) | -4.315(-4.777,-3.851) | -3.866(-4.076, -3.655) |
| Malaysia | 0.269(0.119,0.480) | 0.090(0.037,0.156) | -66.542(-82.807,-28.068) | -3.313(-3.612,-3.013) | -3.505(-4.079, -2.932) |
| Maldives | 0.254(0.118,0.470) | 0.047(0.017,0.087) | -81.411(-92.538,-54.299) | -4.703(-5.223,-4.179) | -5.1(-5.994, -4.206) |
| Myanmar | 78.155(45.651,132.462) | 23.449(10.318,44.155) | -69.997(-87.171,-36.207) | -4.136(-4.345,-3.925) | -3.829(-4.071, -3.588) |
| Philippines | 47.786(37.634,58.601) | 12.210(7.474,17.123) | -74.449(-81.880,-66.517) | -5.271(-5.883,-4.656) | -4.184(-4.386, -3.982) |
| Sri Lanka | 27.588(14.112,43.877) | 3.686(1.384,6.807) | -86.638(-94.021,-73.251) | -8.519(-9.463,-7.565) | -5.845(-6.713, -4.978) |
| Thailand | 1.956(0.836,3.725) | 0.311(0.143,0.505) | -84.093(-91.804,-67.791) | -7.043(-10.825,-3.101) | -0.627(-2.051, 0.797) |
| Timor-Leste | 23.803(6.827,48.620) | 9.048(2.916,16.877) | -61.988(-79.193,-27.827) | -3.588(-3.882,-3.292) | -3.115(-3.341, -2.889) |
| Viet Nam | 8.254(4.009,13.499) | 2.741(1.412,4.622) | -66.793(-81.482,-34.395) | -3.588(-3.679,-3.496) | -3.486(-3.594, -3.377) |
| Fiji | 0.179(0.072,0.424) | 0.192(0.085,0.399) | 6.847(-54.249,157.602) | 1.270(0.449,2.098) | 0.554(-0.203, 1.311) |
| Kiribati | 1.126(0.572,2.136) | 0.860(0.379,1.704) | -23.648(-65.584,74.844) | -0.747(-0.853,-0.641) | -0.809(-0.95, -0.667) |
| Marshall Islands | 0.573(0.170,1.727) | 0.372(0.111,0.864) | -35.031(-70.800,47.156) | -1.566(-1.857,-1.275) | -1.48(-1.89, -1.071) |
| Micronesia (Federated States of) | 0.807(0.354,1.895) | 0.477(0.228,1.036) | -40.931(-71.609,27.327) | -1.493(-1.583,-1.404) | -1.726(-1.853, -1.599) |
| Papua New Guinea | 0.755(0.120,2.855) | 0.656(0.119,1.978) | -13.047(-62.917,113.647) | -0.271(-0.370,-0.173) | -0.34(-0.53, -0.15) |
| Samoa | 0.654(0.247,1.634) | 0.483(0.203,1.105) | -26.061(-63.897,53.185) | -0.694(-0.795,-0.593) | -0.978(-1.064, -0.893) |
| Solomon Islands | 0.720(0.109,2.392) | 0.602(0.153,1.832) | -16.318(-61.279,165.531) | -0.642(-0.779,-0.506) | -0.661(-0.891, -0.431) |
| Tonga | 0.479(0.158,1.359) | 0.327(0.124,0.819) | -31.769(-69.669,62.395) | -0.968(-1.199,-0.737) | -1.178(-1.465, -0.892) |
| Vanuatu | 0.650(0.153,1.952) | 0.484(0.110,1.402) | -25.456(-65.284,69.193) | -0.840(-0.974,-0.707) | -0.979(-1.099, -0.859) |
| Armenia | 0.016(0.009,0.029) | 0.002(0.001,0.002) | -88.901(-93.409,-80.609) | -6.842(-10.619,-2.907) | -7.244(-13.326, -1.162) |
| Azerbaijan | 2.278(0.742,5.537) | 0.843(0.224,2.085) | -62.971(-89.630,35.002) | -4.666(-5.625,-3.698) | -2.845(-3.392, -2.298) |
| Georgia | 1.149(0.584,1.995) | 0.837(0.471,1.357) | -27.152(-66.365,57.119) | -2.037(-5.216,1.249) | 8.495(1.095, 15.895) |
| Kazakhstan | 0.689(0.451,0.985) | 0.928(0.493,1.722) | 34.803(-34.914,185.738) | 1.190(-1.204,3.643) | 7.654(0.096, 15.212) |
| Kyrgyzstan | 1.796(0.960,3.116) | 0.364(0.184,0.639) | -79.752(-92.589,-50.614) | -6.941(-9.668,-4.133) | -0.676(-6.367, 5.014) |
| Mongolia | 11.754(0.964,40.206) | 3.588(0.326,12.272) | -69.470(-94.239,50.957) | -4.475(-4.850,-4.099) | -3.283(-4.532, -2.033) |
| Tajikistan | 0.032(0.009,0.096) | 0.014(0.003,0.043) | -56.970(-89.189,61.260) | -3.290(-3.685,-2.894) | -2.512(-2.859, -2.164) |
| Turkmenistan | 0.149(0.086,0.245) | 0.042(0.019,0.082) | -72.022(-88.519,-30.143) | -0.571(-8.393,7.919) | 96.377(12.257, 180.497) |
| Uzbekistan | 0.418(0.226,0.822) | 0.190(0.125,0.285) | -54.645(-76.911,-14.301) | -4.458(-5.428,-3.478) | -1.392(-4.367, 1.584) |
| Albania | 0.019(0.005,0.043) | 0.001(0.000,0.003) | -95.003(-98.313,-80.837) | -12.593(-14.333,-10.818) | -7.57(-8.395, -6.745) |
| Bosnia and Herzegovina | 0.003(0.001,0.007) | 0.000(0.000,0.001) | -86.456(-96.158,-57.479) | -6.152(-7.111,-5.184) | -5.822(-6.286, -5.358) |
| Bulgaria | 0.014(0.011,0.017) | 0.014(0.009,0.021) | -1.270(-38.976,56.339) | -1.432(-3.256,0.427) | -0.441(-3.97, 3.088) |
| Croatia | 0.011(0.009,0.015) | 0.000(0.000,0.000) | -97.712(-98.465,-96.589) | -13.340(-15.696,-10.919) | -4.822(-10.859, 1.215) |
| Czechia | 0.001(0.001,0.002) | 0.000(0.000,0.000) | -83.633(-89.261,-75.466) | -5.544(-8.434,-2.562) | -5.971(-9.53, -2.412) |
| Hungary | 0.005(0.003,0.005) | 0.006(0.004,0.008) | 31.608(-7.589,98.140) | -8.602(-12.473,-4.559) | 4.372(-3.112, 11.856) |
| North Macedonia | 0.002(0.001,0.006) | 0.001(0.000,0.002) | -67.761(-86.576,-23.287) | -5.337(-6.510,-4.149) | -2.799(-3.771, -1.827) |
| Montenegro | 0.029(0.012,0.057) | 0.014(0.005,0.030) | -51.117(-79.016,9.367) | -2.608(-3.023,-2.191) | -1.813(-2.206, -1.419) |
| Poland | 0.048(0.040,0.055) | 0.000(0.000,0.000) | -99.352(-99.476,-99.156) | -17.473(-20.083,-14.778) | -13.285(-18.092, -8.479) |
| Romania | 0.543(0.460,0.658) | 0.001(0.001,0.001) | -99.801(-99.857,-99.721) | -17.669(-21.220,-13.958) | 13.092(-59.599, 85.783) |
| Serbia | 0.008(0.002,0.015) | 0.001(0.000,0.002) | -84.535(-91.861,-66.790) | -7.572(-8.554,-6.580) | -5.288(-5.738, -4.838) |
| Slovakia | 0.018(0.006,0.038) | 0.007(0.002,0.016) | -63.062(-83.500,-16.506) | -3.207(-3.327,-3.086) | -3.055(-3.314, -2.796) |
| Slovenia | 0.013(0.009,0.016) | 0.004(0.003,0.005) | -69.100(-77.779,-54.846) | -7.816(-10.890,-4.636) | 4.648(-7.764, 17.061) |
| Belarus | 0.087(0.060,0.136) | 0.220(0.163,0.304) | 152.599(44.909,321.381) | 2.703(0.919,4.518) | 4.981(-0.392, 10.353) |
| Estonia | 0.021(0.014,0.030) | 0.005(0.004,0.008) | -74.398(-85.504,-57.002) | -3.230(-7.079,0.778) | -2.261(-7.892, 3.37) |
| Latvia | 1.451(1.051,1.933) | 0.104(0.077,0.145) | -92.854(-95.398,-88.603) | -10.181(-12.630,-7.664) | -3.809(-7.718, 0.1) |
| Lithuania | 0.192(0.104,0.357) | 0.001(0.000,0.001) | -99.725(-99.867,-99.452) | -16.859(-21.194,-12.285) | 1.541(-4.908, 7.99) |
| Republic of Moldova | 0.025(0.016,0.036) | 0.007(0.004,0.012) | -70.645(-82.874,-49.099) | -3.695(-4.655,-2.726) | -2.961(-4.029, -1.894) |
| Russian Federation | 0.357(0.336,0.380) | 0.066(0.059,0.074) | -81.666(-83.457,-79.679) | -4.348(-5.996,-2.672) | -7.991(-12.287, -3.695) |
| Ukraine | 0.141(0.102,0.199) | 0.117(0.064,0.196) | -17.163(-56.266,60.665) | -0.013(-1.465,1.461) | -7.737(-9.126, -6.349) |
| Brunei Darussalam | 0.001(0.000,0.002) | 0.000(0.000,0.001) | -62.744(-77.180,-35.916) | -2.227(-2.967,-1.481) | 31.834(-2.48, 66.149) |
| Japan | 0.054(0.050,0.058) | 0.004(0.003,0.004) | -93.041(-94.053,-92.053) | -7.110(-8.796,-5.393) | -5.149(-8.595, -1.703) |
| Republic of Korea | 0.011(0.003,0.021) | 0.000(0.000,0.001) | -97.108(-98.613,-89.455) | -12.157(-14.229,-10.036) | -2.959(-3.353, -2.564) |
| Singapore | 0.002(0.002,0.003) | 0.001(0.000,0.001) | -75.035(-82.807,-65.946) | -5.912(-7.606,-4.187) | 5.138(-5.174, 15.451) |
| Australia | 0.007(0.005,0.010) | 0.022(0.014,0.035) | 208.407(74.483,430.987) | 12.918(7.585,18.515) | -3.572(-10.444, 3.3) |
| New Zealand | 0.028(0.023,0.033) | 0.005(0.003,0.006) | -83.628(-88.369,-77.231) | -4.669(-7.216,-2.051) | -5.825(-7.302, -4.348) |
| Andorra | 0.003(0.001,0.008) | 0.001(0.000,0.003) | -59.352(-80.707,-20.157) | -2.629(-2.823,-2.435) | -9.823(-15.106, -4.541) |
| Austria | 0.009(0.007,0.012) | 0.005(0.003,0.006) | -47.611(-64.963,-18.851) | -5.219(-9.416,-0.827) | -1.351(-14.243, 11.54) |
| Belgium | 0.015(0.011,0.019) | 0.004(0.003,0.005) | -74.029(-83.543,-60.708) | 1.169(-2.365,4.831) | 12.03(4.983, 19.078) |
| Cyprus | 0.045(0.011,0.117) | 0.006(0.002,0.013) | -86.374(-94.212,-61.362) | -5.602(-6.667,-4.526) | 0.477(-7.803, 8.758) |
| Denmark | 0.054(0.039,0.076) | 0.004(0.002,0.005) | -93.416(-95.840,-89.965) | -8.271(-11.412,-5.019) | -9.853(-12.457, -7.248) |
| Finland | 0.049(0.030,0.085) | 0.022(0.012,0.039) | -55.312(-78.930,-9.991) | -2.959(-3.255,-2.662) | -1.75(-4.407, 0.907) |
| France | 0.001(0.001,0.001) | 0.000(0.000,0.000) | -52.204(-63.906,-34.062) | -5.288(-7.614,-2.904) | -13.013(-19.034, -6.992) |
| Germany | 0.000(0.000,0.001) | 0.005(0.003,0.007) | 1019.972(625.560,1526.396) | 7.160(4.313,10.086) | 23.393(-4.266, 51.052) |
| Greece | 0.003(0.003,0.004) | 0.021(0.014,0.030) | 535.438(348.893,830.912) | 12.572(8.499,16.799) | 7.272(4.074, 10.469) |
| Iceland | 0.029(0.021,0.037) | 0.018(0.014,0.023) | -37.138(-55.157,-12.504) | -2.054(-5.888,1.936) | -2.691(-10.687, 5.306) |
| Ireland | 0.004(0.003,0.005) | 0.003(0.002,0.004) | -26.872(-47.772,4.186) | 2.230(-0.102,4.615) | -3.431(-5.196, -1.667) |
| Israel | 0.010(0.007,0.012) | 0.001(0.000,0.001) | -93.871(-95.841,-91.232) | -8.212(-11.301,-5.016) | 3.08(-9.849, 16.008) |
| Italy | 0.009(0.007,0.010) | 0.000(0.000,0.001) | -94.730(-95.484,-93.639) | -9.970(-11.377,-8.540) | 10.867(-3.191, 24.926) |
| Luxembourg | 0.013(0.011,0.015) | 0.003(0.003,0.004) | -73.847(-80.623,-65.579) | -3.943(-7.927,0.213) | 10.006(4.199, 15.812) |
| Malta | 0.093(0.072,0.119) | 0.002(0.001,0.002) | -98.294(-98.873,-97.377) | -10.827(-14.061,-7.471) | 0.435(-7.12, 7.989) |
| Netherlands | 0.002(0.002,0.003) | 0.007(0.005,0.010) | 222.932(120.475,365.359) | 4.950(1.194,8.846) | 2.048(-20.533, 24.629) |
| Norway | 0.028(0.019,0.036) | 0.147(0.104,0.202) | 422.799(278.551,634.004) | 3.022(1.291,4.784) | -4.241(-4.762, -3.721) |
| Portugal | 0.008(0.005,0.012) | 0.000(0.000,0.000) | -96.149(-97.533,-93.559) | -9.569(-12.161,-6.900) | 16.652(8.063, 25.24) |
| Spain | 0.001(0.001,0.001) | 0.000(0.000,0.000) | -66.495(-75.509,-53.471) | -2.932(-4.155,-1.693) | -1.902(-2.398, -1.405) |
| Sweden | 0.019(0.013,0.025) | 0.017(0.013,0.022) | -8.989(-38.753,30.293) | -2.552(-6.630,1.704) | -0.625(-1.02, -0.23) |
| Switzerland | 0.000(0.000,0.001) | 0.001(0.001,0.001) | 96.928(41.067,160.430) | 0.708(-2.973,4.529) | 1.229(-1.282, 3.74) |
| United Kingdom | 0.006(0.006,0.007) | 0.046(0.041,0.053) | 609.651(518.478,708.935) | 7.111(5.178,9.079) | -3.794(-8.074, 0.485) |
| Argentina | 0.001(0.000,0.001) | 0.000(0.000,0.000) | -27.099(-43.272,-5.661) | -2.450(-5.119,0.295) | -0.95(-8.332, 6.431) |
| Chile | 0.001(0.001,0.002) | 0.001(0.001,0.001) | -35.910(-51.629,-16.057) | -1.070(-4.242,2.207) | -2.252(-2.572, -1.931) |
| Uruguay | 0.009(0.007,0.011) | 0.002(0.001,0.003) | -74.810(-83.724,-59.878) | -4.284(-4.781,-3.784) | 0.685(-0.568, 1.937) |
| Canada | 0.001(0.000,0.001) | 0.029(0.019,0.041) | 5412.867(3544.533,8172.145) | 11.773(7.184,16.559) | -2.047(-2.552, -1.543) |
| United States of America | 0.050(0.047,0.053) | 0.128(0.113,0.144) | 157.350(126.958,190.735) | 2.599(1.903,3.300) | -2.184(-2.677, -1.691) |
| Antigua and Barbuda | 0.133(0.101,0.174) | 0.077(0.059,0.101) | -42.593(-60.626,-13.336) | -1.483(-1.758,-1.206) | -2.348(-2.529, -2.166) |
| Bahamas | 0.068(0.048,0.096) | 0.055(0.036,0.083) | -19.660(-51.194,40.222) | -0.832(-1.070,-0.593) | 54.109(16.881, 91.338) |
| Barbados | 0.137(0.107,0.177) | 0.147(0.108,0.193) | 7.226(-25.551,58.038) | 0.761(-0.052,1.581) | 29.712(-1.428, 60.853) |
| Belize | 0.045(0.033,0.062) | 0.012(0.009,0.017) | -72.307(-81.898,-58.796) | -2.641(-4.120,-1.138) | 19.693(-9.757, 49.142) |
| Cuba | 0.008(0.006,0.012) | 0.006(0.005,0.008) | -27.161(-52.043,19.652) | 1.674(-2.382,5.898) | 6.595(5.111, 8.079) |
| Dominica | 0.156(0.077,0.256) | 0.076(0.032,0.138) | -51.354(-71.342,-20.270) | -2.144(-2.535,-1.750) | 16.801(8.854, 24.747) |
| Dominican Republic | 0.113(0.050,0.195) | 0.113(0.017,0.283) | -0.416(-70.505,162.716) | 1.663(0.147,3.201) | -12.665(-13.054, -12.275) |
| Grenada | 0.268(0.197,0.346) | 0.141(0.102,0.188) | -47.386(-62.344,-24.639) | -0.830(-1.276,-0.383) | -14.282(-27.502, -1.061) |
| Guyana | 0.018(0.013,0.026) | 0.009(0.006,0.013) | -51.377(-71.848,-19.955) | -1.638(-2.393,-0.876) | -14.031(-15.383, -12.679) |
| Haiti | 0.115(0.036,0.277) | 0.054(0.018,0.127) | -52.669(-74.799,-13.816) | -2.596(-2.712,-2.480) | -15.202(-22.719, -7.685) |
| Jamaica | 0.000(0.000,0.001) | 0.007(0.004,0.010) | 1372.860(801.720,2104.809) | 6.855(1.545,12.442) | -16.475(-19.292, -13.658) |
| Saint Lucia | 0.006(0.004,0.007) | 0.009(0.006,0.028) | 68.893(-3.406,378.635) | 2.831(-3.179,9.214) | -14.647(-26.387, -2.908) |
| Saint Vincent and the Grenadines | 0.862(0.669,1.097) | 0.014(0.010,0.027) | -98.351(-98.933,-96.804) | 4.620(-3.716,13.677) | -4.296(-4.451, -4.141) |
| Suriname | 0.004(0.001,0.007) | 0.010(0.004,0.021) | 168.870(15.649,490.840) | 1.597(-1.182,4.454) | -6.483(-8.539, -4.427) |
| Trinidad and Tobago | 0.001(0.001,0.001) | 0.012(0.008,0.019) | 1165.244(708.952,1773.433) | 5.489(0.689,10.517) | -6.98(-7.25, -6.711) |
| Bolivia (Plurinational State of) | 0.833(0.375,1.555) | 0.011(0.004,0.025) | -98.623(-99.396,-96.818) | -13.911(-15.063,-12.742) | -0.871(-8.803, 7.061) |
| Ecuador | 8.052(6.750,9.735) | 0.001(0.000,0.001) | -99.993(-99.995,-99.991) | -31.889(-36.326,-27.143) | -11.616(-13.007, -10.225) |
| Peru | 0.547(0.147,0.911) | 0.003(0.001,0.005) | -99.536(-99.828,-95.569) | -15.543(-17.678,-13.353) | -1.922(-2.093, -1.752) |
| Colombia | 0.402(0.324,0.489) | 0.001(0.001,0.001) | -99.831(-99.875,-99.773) | -14.854(-19.073,-10.415) | -3.441(-3.622, -3.259) |
| Costa Rica | 0.007(0.003,0.015) | 0.004(0.001,0.008) | -44.322(-81.838,68.027) | -1.716(-2.531,-0.894) | -9.399(-9.915, -8.883) |
| El Salvador | 1.390(0.273,2.545) | 0.003(0.001,0.005) | -99.790(-99.918,-97.471) | -21.527(-23.624,-19.372) | -1.628(-2.28, -0.976) |
| Guatemala | 5.091(4.153,6.337) | 0.007(0.005,0.009) | -99.864(-99.908,-99.814) | -19.126(-23.800,-14.164) | -3.627(-4.035, -3.219) |
| Honduras | 0.002(0.001,0.003) | 0.000(0.000,0.001) | -74.955(-88.057,-53.975) | -4.681(-4.883,-4.479) | -2.029(-2.664, -1.394) |
| Mexico | 3.725(3.389,4.046) | 0.001(0.001,0.002) | -99.966(-99.972,-99.959) | -22.365(-23.764,-20.941) | 3.519(-4.355, 11.393) |
| Nicaragua | 0.006(0.002,0.010) | 0.001(0.000,0.001) | -90.639(-95.893,-79.567) | -9.405(-11.240,-7.532) | -2.777(-3.132, -2.422) |
| Panama | 0.044(0.021,0.088) | 0.005(0.003,0.008) | -89.580(-93.410,-82.804) | -6.025(-6.465,-5.582) | 0.007(-0.326, 0.34) |
| Venezuela (Bolivarian Republic of) | 0.038(0.030,0.048) | 0.001(0.001,0.002) | -97.136(-98.211,-95.683) | -13.055(-17.299,-8.594) | -2.366(-2.479, -2.253) |
| Brazil | 3.187(2.194,4.378) | 0.010(0.008,0.011) | -99.699(-99.792,-99.546) | -16.383(-18.222,-14.503) | -4.125(-4.489, -3.761) |
| Paraguay | 0.353(0.132,0.563) | 0.001(0.000,0.002) | -99.582(-99.877,-87.823) | -22.686(-25.976,-19.250) | -3.945(-4.33, -3.561) |
| Algeria | 0.038(0.009,0.088) | 0.021(0.005,0.041) | -45.472(-68.813,16.354) | -1.793(-1.944,-1.642) | 1.28(-2.335, 4.895) |
| Bahrain | 0.246(0.099,0.440) | 0.083(0.031,0.151) | -66.451(-82.144,-37.209) | -3.868(-4.073,-3.663) | -3.526(-4.563, -2.489) |
| Egypt | 8.691(4.532,13.209) | 0.421(0.199,0.699) | -95.155(-97.141,-91.038) | -9.279(-9.907,-8.646) | 1.591(0.375, 2.806) |
| Iran (Islamic Republic of) | 0.112(0.037,0.212) | 0.056(0.023,0.091) | -49.739(-72.512,-2.024) | 0.178(-1.035,1.406) | -2.148(-2.255, -2.04) |
| Iraq | 1.051(0.283,2.307) | 0.323(0.097,0.717) | -69.223(-89.370,-15.220) | -2.539(-3.218,-1.856) | -5.58(-5.979, -5.18) |
| Jordan | 0.002(0.001,0.004) | 0.001(0.000,0.003) | -47.363(-72.052,1.334) | -1.548(-1.989,-1.104) | -2.848(-3.412, -2.284) |
| Kuwait | 0.019(0.008,0.070) | 0.023(0.014,0.042) | 22.577(-52.753,207.225) | -2.550(-4.447,-0.615) | -0.61(-0.921, -0.3) |
| Lebanon | 0.003(0.001,0.007) | 0.001(0.000,0.003) | -57.875(-78.614,-14.974) | -2.127(-2.366,-1.888) | -0.486(-0.649, -0.323) |
| Libya | 0.030(0.009,0.060) | 0.029(0.007,0.062) | -1.883(-54.651,139.171) | 0.424(0.018,0.831) | -9.962(-10.548, -9.376) |
| Morocco | 0.056(0.008,0.140) | 0.027(0.005,0.057) | -51.915(-73.880,-4.400) | -1.816(-2.096,-1.535) | -6.122(-6.586, -5.659) |
| Palestine | 0.044(0.005,0.165) | 0.012(0.002,0.038) | -72.622(-89.693,-1.006) | -3.711(-3.961,-3.461) | -5.554(-5.948, -5.161) |
| Oman | 0.001(0.000,0.003) | 0.000(0.000,0.001) | -71.274(-88.116,-5.084) | -3.440(-3.752,-3.126) | -4.353(-4.601, -4.104) |
| Qatar | 0.002(0.001,0.004) | 0.001(0.000,0.003) | -37.218(-73.110,51.841) | -2.792(-4.807,-0.735) | -4.746(-4.995, -4.498) |
| Saudi Arabia | 0.007(0.002,0.015) | 0.002(0.001,0.004) | -74.695(-88.464,-44.269) | -5.172(-5.992,-4.345) | -3.521(-3.809, -3.233) |
| Syrian Arab Republic | 0.052(0.014,0.103) | 0.059(0.017,0.143) | 14.722(-55.173,180.461) | 2.286(0.655,3.943) | -1.123(-1.358, -0.888) |
| Tunisia | 0.034(0.007,0.080) | 0.017(0.005,0.036) | -49.245(-74.134,23.215) | -1.957(-2.060,-1.853) | -2.635(-3.132, -2.138) |
| Türkiye | 0.472(0.169,1.280) | 0.078(0.026,0.211) | -83.484(-92.717,-60.338) | -5.416(-5.576,-5.257) | -1.517(-1.838, -1.196) |
| United Arab Emirates | 1.793(0.452,4.756) | 0.711(0.244,1.981) | -60.369(-87.927,60.625) | -2.758(-3.337,-2.176) | -4.318(-4.783, -3.852) |
| Yemen | 0.078(0.012,0.206) | 0.063(0.015,0.131) | -19.450(-64.632,122.620) | -0.758(-1.038,-0.477) | -3.257(-3.5, -3.014) |
| Afghanistan | 0.101(0.010,0.302) | 0.087(0.016,0.198) | -14.163(-49.753,122.676) | -0.762(-1.378,-0.141) | -1.688(-1.97, -1.406) |
| Bangladesh | 14.431(3.748,28.193) | 0.474(0.174,0.858) | -96.714(-98.564,-90.534) | -12.955(-14.052,-11.844) | -2.132(-3.28, -0.984) |
| Bhutan | 66.061(12.607,141.101) | 9.301(3.272,19.034) | -85.921(-92.794,-61.457) | -6.737(-7.132,-6.340) | -0.965(-1.507, -0.422) |
| India | 83.993(62.162,105.131) | 14.047(8.387,20.104) | -83.276(-88.327,-76.972) | -6.159(-6.447,-5.870) | -2.051(-2.201, -1.901) |
| Nepal | 313.289(152.120,527.491) | 81.658(45.498,129.558) | -73.935(-85.354,-51.061) | -4.515(-4.680,-4.351) | -3.997(-4.127, -3.866) |
| Pakistan | 69.413(36.594,117.650) | 15.058(9.257,22.498) | -78.306(-86.703,-62.925) | -5.318(-5.578,-5.058) | -2.156(-2.354, -1.959) |
| Angola | 2.439(0.636,6.942) | 0.792(0.156,2.250) | -67.512(-86.657,-36.531) | -3.534(-3.767,-3.301) | -0.954(-1.184, -0.723) |
| Central African Republic | 2.281(0.363,7.321) | 1.596(0.285,4.779) | -30.030(-63.263,40.144) | -1.214(-1.363,-1.066) | -2.005(-2.136, -1.875) |
| Congo | 1.051(0.221,3.028) | 0.408(0.050,1.232) | -61.195(-83.843,-28.531) | -2.915(-3.095,-2.735) | -15.349(-18, -12.698) |
| Democratic Republic of the Congo | 1.559(0.251,4.777) | 0.982(0.185,2.871) | -37.006(-68.673,20.317) | -1.225(-1.501,-0.948) | -2.238(-2.401, -2.074) |
| Equatorial Guinea | 1.913(0.220,5.598) | 0.474(0.106,1.284) | -75.222(-91.051,14.248) | -5.441(-5.764,-5.118) | -3.275(-3.537, -3.012) |
| Gabon | 1.069(0.305,2.885) | 0.369(0.059,1.052) | -65.523(-84.604,-31.851) | -3.056(-3.187,-2.924) | 4.33(2.795, 5.865) |
| Burundi | 30.815(14.247,57.903) | 17.887(4.142,53.110) | -41.954(-81.456,28.781) | -1.749(-2.017,-1.481) | 0.219(0.085, 0.353) |
| Comoros | 21.880(8.112,45.579) | 11.089(3.309,26.550) | -49.318(-76.163,1.603) | -2.312(-2.718,-1.904) | -2.348(-2.541, -2.155) |
| Djibouti | 11.835(3.070,30.482) | 8.423(2.468,21.299) | -28.827(-62.426,46.119) | -1.010(-1.489,-0.529) | -1.507(-1.868, -1.146) |
| Eritrea | 31.260(17.416,54.630) | 16.646(6.857,31.641) | -46.749(-72.282,-0.988) | -1.806(-1.952,-1.659) | -2.783(-2.993, -2.573) |
| Ethiopia | 205.451(61.391,508.921) | 58.031(17.354,163.639) | -71.754(-86.513,-48.338) | -4.396(-4.643,-4.147) | -2.347(-2.603, -2.091) |
| Kenya | 20.172(8.899,37.802) | 10.345(4.380,18.313) | -48.717(-68.012,-16.707) | -1.462(-1.878,-1.044) | -0.98(-1.199, -0.762) |
| Madagascar | 14.836(7.272,24.857) | 10.927(4.765,20.237) | -26.345(-63.050,26.268) | -0.739(-1.096,-0.380) | -1.662(-1.878, -1.447) |
| Malawi | 48.029(24.350,82.193) | 25.453(10.970,44.038) | -47.005(-74.001,14.748) | -2.123(-2.421,-1.824) | -2.233(-2.728, -1.739) |
| Mauritius | 0.236(0.203,0.270) | 0.001(0.001,0.001) | -99.684(-99.753,-99.583) | -13.207(-15.448,-10.905) | -1.576(-1.774, -1.379) |
| Mozambique | 48.429(25.354,79.370) | 23.798(9.741,44.423) | -50.860(-77.469,-11.683) | -1.825(-2.021,-1.629) | 0.334(-0.318, 0.987) |
| Rwanda | 35.555(15.514,81.970) | 12.418(2.109,41.320) | -65.075(-89.114,-22.859) | -4.147(-4.578,-3.714) | -1.393(-1.709, -1.076) |
| Seychelles | 0.013(0.005,0.025) | 0.034(0.011,0.072) | 151.445(20.384,443.512) | 2.632(0.528,4.781) | -1.958(-2.173, -1.743) |
| Somalia | 30.406(5.633,80.675) | 31.778(5.938,94.634) | 4.511(-53.893,94.327) | 0.223(-0.021,0.467) | -1.567(-1.741, -1.392) |
| United Republic of Tanzania | 32.917(16.049,57.925) | 15.679(5.280,33.808) | -52.367(-82.009,1.125) | -2.258(-2.514,-2.001) | -2.808(-3.189, -2.427) |
| Uganda | 17.037(6.205,37.106) | 10.671(3.689,20.927) | -37.367(-70.451,46.987) | -1.946(-2.295,-1.595) | -0.998(-1.144, -0.852) |
| Zambia | 39.949(23.635,65.684) | 16.022(6.794,28.757) | -59.892(-81.491,-15.001) | -3.015(-3.510,-2.519) | -1.279(-1.744, -0.814) |
| Botswana | 4.149(1.635,8.803) | 2.048(0.989,3.978) | -50.637(-79.831,14.110) | -2.081(-2.245,-1.916) | -1.306(-1.981, -0.63) |
| Lesotho | 5.127(2.050,10.510) | 3.711(1.655,6.969) | -27.620(-68.938,71.821) | -0.415(-0.781,-0.047) | -1.99(-2.24, -1.74) |
| Namibia | 3.557(1.634,6.616) | 2.129(0.876,4.390) | -40.141(-71.658,25.351) | -1.599(-1.701,-1.497) | -1.947(-2.273, -1.621) |
| South Africa | 2.644(1.432,4.422) | 1.275(0.729,2.007) | -51.765(-71.812,-16.951) | -2.483(-3.227,-1.733) | -2.078(-2.441, -1.715) |
| Eswatini | 4.874(2.337,9.610) | 2.918(1.431,5.467) | -40.131(-71.844,23.698) | -1.260(-1.405,-1.115) | -2.347(-2.857, -1.837) |
| Zimbabwe | 5.303(2.552,8.678) | 5.787(3.074,11.012) | 9.141(-47.206,116.381) | 0.866(0.333,1.401) | -1.955(-2.235, -1.675) |
| Benin | 23.999(6.417,58.441) | 15.705(4.972,34.263) | -34.559(-71.633,58.671) | -1.194(-1.336,-1.053) | -3.024(-3.207, -2.841) |
| Burkina Faso | 34.688(10.406,74.710) | 18.576(5.811,41.638) | -46.449(-74.968,11.078) | -2.206(-2.437,-1.974) | -1.599(-2.151, -1.048) |
| Cameroon | 18.695(5.732,38.732) | 11.786(4.468,23.182) | -36.955(-70.178,40.207) | -1.155(-1.399,-0.910) | -2.053(-2.189, -1.917) |
| Cabo Verde | 12.916(3.309,31.116) | 5.196(1.614,10.721) | -59.770(-80.090,-7.823) | -3.349(-3.619,-3.078) | -3.012(-3.961, -2.064) |
| Chad | 31.967(5.418,86.067) | 22.813(3.294,59.445) | -28.635(-66.059,48.380) | -0.971(-1.084,-0.858) | -2.669(-3.017, -2.321) |
| Côte d'Ivoire | 19.287(5.063,41.786) | 12.897(3.747,26.842) | -33.131(-65.244,35.847) | -0.851(-1.103,-0.599) | -2.187(-2.564, -1.809) |
| Gambia | 20.337(4.583,49.438) | 13.431(4.648,30.551) | -33.955(-67.496,50.074) | -1.420(-1.697,-1.142) | -1.806(-2.009, -1.603) |
| Ghana | 41.172(18.914,72.608) | 21.686(9.430,38.863) | -47.330(-70.848,-2.970) | -1.658(-1.955,-1.360) | -1.181(-1.442, -0.921) |
| Guinea | 34.182(10.371,74.873) | 18.136(4.690,38.537) | -46.941(-75.766,-0.008) | -1.528(-1.730,-1.325) | -2.259(-3.019, -1.5) |
| Guinea-Bissau | 32.681(9.125,75.231) | 16.911(4.585,38.190) | -48.255(-73.757,1.128) | -1.609(-1.825,-1.393) | -2.625(-3.018, -2.232) |
| Liberia | 21.788(5.071,46.597) | 11.284(2.060,26.576) | -48.209(-79.357,-2.027) | -2.418(-2.683,-2.153) | 0.948(-0.304, 2.199) |
| Mali | 34.950(5.850,93.138) | 18.401(3.723,46.522) | -47.349(-73.848,0.794) | -1.913(-2.149,-1.676) | -2.225(-2.716, -1.735) |
| Mauritania | 18.251(6.344,36.597) | 7.254(2.114,14.964) | -60.256(-79.612,-24.829) | -2.788(-2.980,-2.595) | -1.176(-2.397, 0.045) |
| Niger | 44.141(8.218,122.525) | 26.728(4.201,74.480) | -39.448(-79.589,26.645) | -1.767(-1.987,-1.547) | -0.105(-0.509, 0.3) |
| Nigeria | 42.487(22.647,67.288) | 22.398(9.413,38.301) | -47.283(-75.521,-0.981) | -1.904(-2.198,-1.609) | 0.067(-0.326, 0.46) |
| Sao Tome and Principe | 16.693(4.194,36.596) | 6.378(1.870,12.791) | -61.794(-80.909,-11.563) | -3.277(-3.549,-3.004) | -1.195(-1.646, -0.743) |
| Senegal | 29.822(9.754,63.349) | 12.901(4.115,27.565) | -56.738(-78.416,-17.100) | -2.345(-2.568,-2.122) | -0.634(-0.754, -0.514) |
| Sierra Leone | 26.679(9.650,58.617) | 13.454(3.281,33.861) | -49.570(-79.208,7.962) | -2.091(-2.382,-1.798) | 9.108(1.531, 16.686) |
| Togo | 23.080(9.536,47.301) | 13.033(4.680,27.300) | -43.532(-71.396,10.532) | -1.586(-1.739,-1.433) | -1.33(-1.963, -0.698) |
| American Samoa | 0.674(0.225,1.508) | 0.456(0.147,1.102) | -32.323(-68.540,46.966) | -0.860(-1.035,-0.685) | -11.691(-12.93, -10.451) |
| Bermuda | 0.109(0.084,0.143) | 0.051(0.036,0.071) | -53.603(-70.220,-28.019) | -1.707(-2.434,-0.975) | -0.36(-0.933, 0.212) |
| Cook Islands | 1.325(0.389,2.852) | 0.556(0.195,1.004) | -58.028(-82.619,47.641) | -3.234(-3.564,-2.902) | -2.64(-2.708, -2.571) |
| Greenland | 0.085(0.036,0.163) | 0.082(0.022,0.188) | -3.562(-62.077,120.595) | 1.209(-0.026,2.459) | -1.175(-1.653, -0.697) |
| Guam | 0.316(0.153,0.571) | 0.161(0.080,0.293) | -48.850(-72.458,-8.058) | -0.994(-1.452,-0.533) | 0.339(-0.158, 0.836) |
| Monaco | 0.100(0.049,0.194) | 0.064(0.028,0.123) | -35.678(-69.343,36.496) | -1.560(-2.243,-0.872) | -2.242(-2.429, -2.056) |
| Nauru | 0.536(0.222,1.065) | 0.506(0.248,1.022) | -5.613(-56.203,104.548) | -0.004(-0.502,0.495) | -0.619(-0.709, -0.53) |
| Niue | 0.215(0.030,0.720) | 0.202(0.031,0.572) | -6.158(-66.827,161.938) | -0.994(-1.312,-0.675) | -1.313(-1.478, -1.148) |
| Northern Mariana Islands | 1.188(0.480,2.560) | 0.883(0.398,1.809) | -25.737(-59.983,38.463) | -0.548(-0.894,-0.201) | -1.383(-1.869, -0.897) |
| Palau | 0.058(0.006,0.173) | 0.047(0.006,0.129) | -18.988(-64.731,105.196) | -0.855(-0.962,-0.749) | -0.896(-1.038, -0.754) |
| Puerto Rico | 0.001(0.001,0.001) | 0.005(0.004,0.006) | 632.625(346.911,910.760) | 5.786(1.912,9.807) | 9.975(7.627, 12.323) |
| Saint Kitts and Nevis | 0.525(0.410,0.674) | 0.340(0.255,0.457) | -35.094(-54.008,-4.497) | -0.879(-1.127,-0.630) | -1.229(-1.272, -1.186) |
| San Marino | 0.142(0.053,0.290) | 0.002(0.000,0.004) | -98.881(-99.587,-97.354) | -18.268(-20.471,-16.004) | -12.333(-13.742, -10.924) |
| Tokelau | 0.297(0.037,1.007) | 0.242(0.030,0.750) | -18.457(-65.764,99.024) | -1.692(-2.088,-1.295) | -1.871(-2.112, -1.63) |
| Tuvalu | 1.069(0.328,3.036) | 0.467(0.216,0.998) | -56.301(-82.756,13.546) | -2.277(-2.417,-2.136) | -2.923(-2.967, -2.88) |
| United States Virgin Islands | 0.046(0.018,0.085) | 0.030(0.007,0.066) | -35.496(-74.058,39.997) | -0.440(-0.804,-0.075) | -2.03(-2.284, -1.776) |
| South Sudan | 17.147(3.729,39.732) | 17.859(3.749,42.324) | 4.148(-50.404,96.875) | 0.059(-0.255,0.373) | 0.149(-0.032, 0.33) |
| Sudan | 0.086(0.021,0.179) | 0.042(0.010,0.089) | -51.080(-76.874,7.723) | -2.167(-2.457,-1.875) | -2.38(-2.453, -2.307) |

Abbreviations: ASDR, age standardized Disability-adjusted life year rate; AAPC, average annual percent change; CI, confidence interval; EAPC, estimated annual percentage change; UI, uncertainty interval.

Supplementary Table 12: Trends in the number of rabies DALYs cases in 204 countries and territories Worldwide from 1990 to 2021.

| Location | Rabies. DALYs cases. (persons)  (95% UI).  1990 year. | Rabies. DALYs cases. (persons)  (95% UI).  2021 year. | Rabies. Percentage change (95% UI). 1990－2021. | Rabies. EAPC (95% CI). 1990－2021. | Rabies. AAPC (95% CI). 1990－2021. |
| --- | --- | --- | --- | --- | --- |
| China | 68897(40292,97255) | 25855(14027,39256) | -0.625(-0.751, -0.465) | -1.675(-3.692,0.385) | -1.901(-2.892, -0.911) |
| Democratic People's Republic of Korea | 580(221,1114) | 736(288,1316) | 0.268(-0.269, 1.241) | -0.129(-0.559,0.303) | 0.802(0.643, 0.961) |
| Taiwan (Province of China) | 2(1,2) | 1(0,1) | -0.688(-0.775, -0.568) | -2.293(-4.440,-0.097) | -3.896(-8.255, 0.463) |
| Cambodia | 3325(1183,6001) | 1385(389,2767) | -0.583(-0.802, -0.26) | -5.519(-6.053,-4.981) | -2.655(-2.878, -2.431) |
| Indonesia | 9933(5419,15325) | 2987(1618,5465) | -0.699(-0.869, -0.334) | -4.994(-5.219,-4.769) | -3.778(-4.027, -3.529) |
| Lao People's Democratic Republic | 1488(673,2584) | 679(243,1268) | -0.544(-0.797, -0.008) | -4.795(-5.292,-4.296) | -2.465(-2.659, -2.271) |
| Malaysia | 57(25,104) | 28(11,48) | -0.52(-0.749, 0.037) | -4.157(-4.461,-3.851) | -2.395(-3.007, -1.784) |
| Maldives | 1(0,1) | 0(0,0) | -0.637(-0.874, -0.048) | -5.311(-5.831,-4.788) | -3.009(-3.919, -2.098) |
| Myanmar | 34842(19349,60177) | 13460(6001,25087) | -0.614(-0.846, -0.124) | -4.382(-4.580,-4.184) | -3.033(-3.274, -2.791) |
| Philippines | 34195(27557,42209) | 13898(8517,19508) | -0.594(-0.717, -0.454) | -5.645(-6.253,-5.033) | -2.729(-2.96, -2.498) |
| Sri Lanka | 4725(2419,7567) | 857(321,1585) | -0.819(-0.919, -0.636) | -8.391(-9.334,-7.438) | -4.882(-5.691, -4.074) |
| Thailand | 1129(477,2177) | 196(87,317) | -0.827(-0.914, -0.645) | -7.133(-10.915,-3.191) | -0.219(-1.687, 1.249) |
| Timor-Leste | 218(67,434) | 134(44,251) | -0.386(-0.71, 0.246) | -3.937(-4.221,-3.652) | -1.644(-1.895, -1.392) |
| Viet Nam | 5944(2823,9881) | 2662(1367,4400) | -0.552(-0.761, -0.133) | -4.033(-4.144,-3.922) | -2.549(-2.677, -2.421) |
| Fiji | 1(0,1) | 1(1,3) | 1.139(-0.056, 3.659) | 3.393(2.240,4.559) | 3.078(2.112, 4.044) |
| Kiribati | 0(0,0) | 0(0,1) | 0.353(-0.478, 2.494) | -0.621(-0.713,-0.529) | 1.015(0.868, 1.163) |
| Marshall Islands | 0(0,0) | 0(0,0) | 0.168(-0.438, 1.548) | -0.256(-0.568,0.057) | 0.387(-0.065, 0.84) |
| Micronesia (Federated States of) | 0(0,1) | 0(0,1) | -0.292(-0.645, 0.418) | -0.847(-0.981,-0.713) | -1.136(-1.303, -0.969) |
| Papua New Guinea | 12(2,39) | 40(7,120) | 2.36(0.675, 6.318) | 1.054(0.831,1.277) | 4.078(3.782, 4.374) |
| Samoa | 0(0,1) | 1(0,2) | 0.368(-0.278, 1.722) | 0.542(0.338,0.747) | 0.996(0.896, 1.095) |
| Solomon Islands | 1(0,3) | 2(1,6) | 1.56(0.308, 5.935) | 0.946(0.649,1.244) | 2.96(2.692, 3.228) |
| Tonga | 0(0,1) | 0(0,1) | 0.151(-0.48, 1.706) | 0.440(0.210,0.670) | 0.481(0.178, 0.784) |
| Vanuatu | 0(0,1) | 1(0,2) | 1.417(0.24, 4.142) | 0.625(0.398,0.852) | 2.946(2.813, 3.078) |
| Armenia | 1(0,1) | 0(0,0) | -0.894(-0.938, -0.811) | -6.532(-10.106,-2.815) | 36.833(-118.466, 192.132) |
| Azerbaijan | 173(51,443) | 88(24,218) | -0.489(-0.862, 0.926) | -5.043(-6.131,-3.944) | -1.617(-2.548, -0.686) |
| Georgia | 61(31,107) | 30(17,48) | -0.51(-0.767, 0.047) | -2.107(-5.286,1.179) | 6.908(-0.397, 14.213) |
| Kazakhstan | 118(77,168) | 177(93,332) | 0.495(-0.284, 2.211) | 0.903(-1.537,3.405) | 8.338(0.545, 16.13) |
| Kyrgyzstan | 90(48,156) | 24(12,43) | -0.73(-0.903, -0.344) | -7.533(-10.265,-4.717) | 0.259(-5.5, 6.018) |
| Mongolia | 351(26,1188) | 124(11,419) | -0.648(-0.934, 0.712) | -5.999(-6.479,-5.518) | -2.784(-3.923, -1.645) |
| Tajikistan | 2(0,6) | 1(0,5) | -0.249(-0.846, 2.138) | -3.962(-4.458,-3.464) | -0.631(-1.272, 0.01) |
| Turkmenistan | 6(3,9) | 2(1,4) | -0.614(-0.842, -0.023) | -0.421(-8.487,8.355) | 107.613(8.03, 207.197) |
| Uzbekistan | 68(37,133) | 62(39,93) | -0.088(-0.535, 0.79) | -4.170(-5.302,-3.025) | 1.135(-2.205, 4.475) |
| Albania | 1(0,1) | 0(0,0) | -0.944(-0.983, -0.757) | -11.722(-13.517,-9.890) | -7.252(-8.011, -6.492) |
| Bosnia and Herzegovina | 0(0,0) | 0(0,0) | -0.789(-0.944, -0.338) | -3.327(-4.012,-2.638) | -4.435(-4.809, -4.06) |
| Bulgaria | 1(1,1) | 1(1,1) | 0.161(-0.272, 0.847) | 1.037(-0.355,2.449) | -1.874(-7.11, 3.362) |
| Croatia | 0(0,1) | 0(0,0) | -0.95(-0.968, -0.921) | -10.652(-12.928,-8.316) | -4.445(-7.729, -1.161) |
| Czechia | 0(0,0) | 0(0,0) | -0.71(-0.807, -0.551) | -4.111(-6.766,-1.381) | 2.657(-3.667, 8.982) |
| Hungary | 0(0,1) | 1(0,1) | 0.201(-0.142, 0.739) | -7.443(-10.865,-3.890) | -2.529(-4.311, -0.747) |
| North Macedonia | 0(0,0) | 0(0,0) | -0.547(-0.819, 0.04) | -5.186(-6.478,-3.875) | -1.381(-1.71, -1.051) |
| Montenegro | 0(0,0) | 0(0,0) | -0.427(-0.769, 0.24) | -2.031(-2.419,-1.641) | -11.253(-15.957, -6.549) |
| Poland | 13(11,15) | 0(0,0) | -0.986(-0.989, -0.981) | -15.414(-17.942,-12.809) | 13.147(-61, 87.295) |
| Romania | 131(111,157) | 0(0,0) | -0.998(-0.999, -0.997) | -17.376(-20.942,-13.650) | -2.986(-3.742, -2.229) |
| Serbia | 1(0,1) | 0(0,0) | -0.65(-0.822, -0.257) | -3.513(-4.114,-2.908) | -3.822(-4.12, -3.524) |
| Slovakia | 1(0,2) | 0(0,1) | -0.696(-0.856, -0.331) | -3.757(-3.875,-3.639) | 6.661(-6.192, 19.515) |
| Slovenia | 0(0,0) | 0(0,0) | -0.277(-0.464, 0.034) | -4.905(-7.782,-1.939) | 6.283(3.807, 8.759) |
| Belarus | 10(7,15) | 30(22,42) | 2.034(0.671, 3.935) | 3.349(1.318,5.420) | 1.68(-3.312, 6.671) |
| Estonia | 0(0,0) | 0(0,0) | -0.2(-0.535, 0.326) | 1.276(-2.526,5.228) | -4.696(-15.761, 6.369) |
| Latvia | 29(21,38) | 2(1,2) | -0.942(-0.962, -0.91) | -8.672(-10.916,-6.372) | 84.366(-465.244, 633.975) |
| Lithuania | 6(3,10) | 0(0,0) | -0.996(-0.998, -0.993) | -15.695(-19.853,-11.321) | -3.774(-7.708, 0.16) |
| Republic of Moldova | 1(1,2) | 0(0,0) | -0.867(-0.923, -0.77) | -5.380(-6.571,-4.173) | 0.823(-13.639, 15.285) |
| Russian Federation | 511(480,542) | 93(84,105) | -0.817(-0.836, -0.796) | -4.307(-5.969,-2.617) | -0.683(-1.811, 0.444) |
| Ukraine | 71(52,103) | 54(27,96) | -0.25(-0.623, 0.498) | 0.231(-1.230,1.713) | -6.608(-10.673, -2.543) |
| Brunei Darussalam | 0(0,0) | 0(0,0) | -0.183(-0.518, 0.385) | -1.356(-2.067,-0.640) | -5.467(-7.021, -3.913) |
| Japan | 41(38,44) | 5(4,6) | -0.882(-0.904, -0.863) | -5.576(-7.277,-3.843) | 23.514(-8.716, 55.744) |
| Republic of Korea | 4(1,7) | 0(0,0) | -0.941(-0.972, -0.834) | -10.589(-12.758,-8.366) | -3.623(-6.616, -0.629) |
| Singapore | 0(0,0) | 0(0,0) | -0.159(-0.494, 0.279) | -3.869(-5.767,-1.933) | 0.033(-0.407, 0.474) |
| Australia | 1(1,1) | 4(2,6) | 2.524(1.012, 4.973) | 11.461(6.421,16.740) | 4.185(-3.713, 12.083) |
| New Zealand | 1(1,1) | 0(0,0) | -0.745(-0.818, -0.656) | -4.130(-6.303,-1.906) | -2.787(-8.292, 2.719) |
| Andorra | 0(0,0) | 0(0,0) | -0.032(-0.524, 0.881) | -1.475(-1.704,-1.246) | -1.433(-2.713, -0.153) |
| Austria | 0(0,1) | 0(0,0) | -0.302(-0.515, 0.05) | -3.779(-7.139,-0.297) | -8.513(-13.809, -3.217) |
| Belgium | 1(1,1) | 0(0,0) | -0.679(-0.776, -0.544) | 1.125(-1.965,4.312) | 15.958(9.447, 22.469) |
| Cyprus | 0(0,1) | 0(0,0) | -0.397(-0.736, 0.617) | -2.673(-3.824,-1.509) | 3.075(-6.475, 12.626) |
| Denmark | 2(2,3) | 0(0,0) | -0.892(-0.924, -0.847) | -7.495(-10.239,-4.666) | 4.053(-4.079, 12.186) |
| Finland | 2(1,3) | 1(0,1) | -0.547(-0.774, -0.128) | -3.163(-3.471,-2.854) | 1.586(-0.871, 4.043) |
| France | 0(0,1) | 0(0,1) | -0.015(-0.303, 0.425) | -3.208(-5.363,-1.004) | -12.071(-17.046, -7.096) |
| Germany | 0(0,1) | 2(1,3) | 4.475(2.535, 6.958) | 4.979(2.819,7.183) | 20.366(-3.144, 43.875) |
| Greece | 0(0,0) | 6(4,9) | 20.803(14.269, 30.117) | 16.727(12.545,21.066) | 8.325(5.081, 11.57) |
| Iceland | 0(0,0) | 0(0,0) | 0.382(-0.022, 0.959) | -0.590(-4.521,3.502) | 4.612(-1.92, 11.144) |
| Ireland | 0(0,0) | 0(0,0) | -0.086(-0.324, 0.276) | 1.773(-0.493,4.090) | -1.843(-4.548, 0.862) |
| Israel | 0(0,1) | 0(0,0) | -0.858(-0.902, -0.797) | -7.822(-10.910,-4.627) | 3.68(-6.293, 13.653) |
| Italy | 4(3,4) | 0(0,1) | -0.88(-0.899, -0.858) | -7.383(-8.660,-6.088) | 12.684(-1.396, 26.765) |
| Luxembourg | 0(0,0) | 0(0,0) | -0.28(-0.473, -0.043) | -2.317(-6.184,1.710) | 10.76(1.923, 19.597) |
| Malta | 0(0,0) | 0(0,0) | -0.978(-0.984, -0.969) | -10.389(-13.262,-7.420) | 31.031(-44.772, 106.833) |
| Netherlands | 0(0,0) | 1(1,1) | 2.216(1.19, 3.592) | 4.369(0.840,8.022) | 2.148(-18.915, 23.212) |
| Norway | 1(1,2) | 11(8,14) | 6.349(4.518, 8.571) | 3.548(1.838,5.286) | -4.893(-5.426, -4.359) |
| Portugal | 1(0,1) | 0(0,0) | -0.895(-0.938, -0.793) | -6.184(-9.081,-3.194) | 14.979(7.81, 22.149) |
| Spain | 0(0,0) | 0(0,0) | -0.356(-0.521, -0.104) | -0.928(-2.157,0.317) | -0.846(-1.931, 0.239) |
| Sweden | 1(1,2) | 2(1,2) | 0.253(-0.147, 0.767) | -1.577(-4.987,1.956) | 0.857(0.393, 1.32) |
| Switzerland | 0(0,0) | 0(0,0) | 3.93(2.363, 5.926) | 2.942(-0.534,6.538) | 2.563(-0.877, 6.003) |
| United Kingdom | 3(3,3) | 22(20,25) | 6.612(5.772, 7.553) | 6.894(4.995,8.828) | -1.891(-6.48, 2.698) |
| Argentina | 0(0,0) | 0(0,0) | 0.096(-0.146, 0.444) | -2.194(-4.851,0.537) | -1.562(-8.776, 5.652) |
| Chile | 0(0,0) | 0(0,0) | -0.059(-0.29, 0.25) | -1.362(-4.431,1.805) | -1.71(-2.018, -1.401) |
| Uruguay | 0(0,0) | 0(0,0) | -0.791(-0.861, -0.672) | -5.103(-5.603,-4.599) | 3.046(1.722, 4.37) |
| Canada | 0(0,0) | 6(4,8) | 41.949(27.269, 62.52) | 10.532(6.726,14.474) | -2.471(-3.013, -1.928) |
| United States of America | 107(100,113) | 306(274,344) | 1.876(1.561, 2.209) | 2.096(1.400,2.795) | -1.011(-1.36, -0.662) |
| Antigua and Barbuda | 0(0,0) | 0(0,0) | -0.169(-0.437, 0.371) | -1.684(-1.970,-1.397) | -0.616(-0.876, -0.357) |
| Bahamas | 0(0,0) | 0(0,0) | 0.29(-0.213, 1.365) | -0.755(-1.073,-0.436) | 56.006(11.264, 100.749) |
| Barbados | 0(0,0) | 1(0,1) | 0.549(0.05, 1.236) | 1.320(0.242,2.409) | 31.314(9.323, 53.305) |
| Belize | 0(0,0) | 0(0,0) | -0.649(-0.778, -0.464) | -4.336(-5.852,-2.797) | 20.322(-25.382, 66.026) |
| Cuba | 1(1,1) | 1(0,1) | -0.235(-0.489, 0.252) | 0.870(-3.040,4.937) | 7.493(5.785, 9.2) |
| Dominica | 0(0,0) | 0(0,0) | -0.422(-0.669, 0.01) | -1.405(-1.723,-1.085) | 26.204(-28.465, 80.874) |
| Dominican Republic | 6(2,10) | 12(2,29) | 0.91(-0.487, 4.319) | 2.941(1.145,4.769) | -11.56(-11.959, -11.16) |
| Grenada | 0(0,0) | 0(0,0) | -0.548(-0.695, -0.342) | -1.885(-2.316,-1.452) | -12.708(-27.604, 2.188) |
| Guyana | 0(0,0) | 0(0,0) | -0.326(-0.583, 0.058) | 0.058(-0.417,0.534) | -12.825(-14.374, -11.276) |
| Haiti | 4(2,8) | 4(1,7) | -0.188(-0.598, 0.46) | -3.006(-3.179,-2.832) | -14.532(-22.363, -6.7) |
| Jamaica | 0(0,0) | 0(0,0) | 21.578(13.152, 33.629) | 8.026(2.791,13.527) | 1.102(-0.523, 2.728) |
| Saint Lucia | 0(0,0) | 0(0,0) | 2.907(1.089, 10.798) | 4.561(-1.481,10.973) | -16.536(-19.142, -13.931) |
| Saint Vincent and the Grenadines | 1(1,1) | 0(0,0) | -0.979(-0.986, -0.958) | 5.624(-2.654,14.607) | -17.314(-28.789, -5.839) |
| Suriname | 0(0,0) | 0(0,0) | 2.521(0.419, 7.639) | 1.796(-0.564,4.211) | -2.542(-2.669, -2.415) |
| Trinidad and Tobago | 0(0,0) | 0(0,0) | 19.108(11.591, 28.467) | 6.731(2.175,11.490) | -5.849(-8.005, -3.693) |
| Bolivia (Plurinational State of) | 57(24,112) | 1(0,2) | -0.98(-0.992, -0.945) | -14.629(-15.870,-13.370) | -5.056(-5.494, -4.619) |
| Ecuador | 870(706,1085) | 0(0,0) | -1(-1, -1) | -32.293(-36.911,-27.337) | -5.145(-19.449, 9.16) |
| Peru | 133(32,229) | 1(0,2) | -0.994(-0.998, -0.936) | -16.011(-18.310,-13.648) | -10.195(-12.151, -8.24) |
| Colombia | 141(113,173) | 0(0,0) | -0.998(-0.998, -0.997) | -15.216(-19.457,-10.753) | -0.3(-0.461, -0.14) |
| Costa Rica | 0(0,0) | 0(0,0) | 0.207(-0.606, 2.636) | -1.040(-1.924,-0.147) | 0.015(-0.128, 0.158) |
| El Salvador | 84(16,162) | 0(0,0) | -0.998(-0.999, -0.973) | -21.876(-23.967,-19.727) | -7.842(-8.463, -7.222) |
| Guatemala | 491(383,635) | 1(1,1) | -0.998(-0.999, -0.997) | -19.323(-24.004,-14.353) | -0.563(-1.546, 0.42) |
| Honduras | 0(0,0) | 0(0,0) | -0.558(-0.821, -0.129) | -5.360(-5.590,-5.128) | -1.618(-2.043, -1.192) |
| Mexico | 3621(3269,3995) | 2(1,2) | -1(-1, -0.999) | -22.745(-24.145,-21.320) | 2.016(1.303, 2.73) |
| Nicaragua | 0(0,1) | 0(0,0) | -0.889(-0.953, -0.748) | -10.525(-12.441,-8.567) | -0.286(-0.624, 0.053) |
| Panama | 1(1,2) | 0(0,0) | -0.804(-0.883, -0.661) | -5.858(-6.325,-5.389) | 1.908(1.535, 2.28) |
| Venezuela (Bolivarian Republic of) | 7(5,9) | 0(0,0) | -0.961(-0.976, -0.94) | -12.843(-17.333,-8.109) | -1.028(-1.154, -0.902) |
| Brazil | 5279(3592,7346) | 20(17,23) | -0.996(-0.997, -0.994) | -16.909(-18.735,-15.043) | -1.317(-1.861, -0.774) |
| Paraguay | 16(6,27) | 0(0,0) | -0.994(-0.998, -0.799) | -23.321(-26.695,-19.791) | -1.396(-1.894, -0.897) |
| Algeria | 9(2,22) | 8(2,17) | -0.092(-0.502, 1.008) | -2.045(-2.227,-1.863) | 9.612(6.529, 12.694) |
| Bahrain | 1(0,2) | 1(0,2) | -0.001(-0.484, 0.996) | -3.927(-4.168,-3.686) | 0.007(-1.118, 1.132) |
| Egypt | 5671(2795,8827) | 464(212,781) | -0.918(-0.954, -0.842) | -9.661(-10.331,-8.986) | 4.226(2.603, 5.848) |
| Iran (Islamic Republic of) | 63(21,118) | 45(19,74) | -0.283(-0.612, 0.395) | -0.040(-1.276,1.211) | -0.919(-1.037, -0.802) |
| Iraq | 262(63,577) | 147(40,336) | -0.44(-0.827, 0.707) | -3.148(-3.831,-2.460) | -4.445(-4.892, -3.998) |
| Jordan | 0(0,0) | 0(0,0) | 0.76(-0.133, 2.567) | -1.394(-1.839,-0.947) | 0.178(-0.415, 0.771) |
| Kuwait | 0(0,2) | 1(1,2) | 1.745(0.037, 6.439) | -2.851(-4.503,-1.170) | 2.23(2.069, 2.391) |
| Lebanon | 0(0,0) | 0(0,0) | -0.091(-0.538, 0.876) | -1.710(-1.941,-1.479) | 3.24(2.803, 3.677) |
| Libya | 1(0,2) | 2(0,4) | 0.774(-0.183, 3.396) | 0.626(0.204,1.049) | -9.796(-10.529, -9.063) |
| Morocco | 13(2,34) | 10(2,21) | -0.269(-0.627, 0.505) | -1.654(-1.948,-1.359) | -5.86(-6.308, -5.411) |
| Palestine | 1(0,4) | 1(0,2) | -0.351(-0.782, 1.722) | -3.854(-4.092,-3.616) | -4.242(-4.659, -3.825) |
| Oman | 0(0,0) | 0(0,0) | -0.343(-0.753, 0.991) | -3.481(-3.807,-3.154) | -3.074(-3.271, -2.876) |
| Qatar | 0(0,0) | 0(0,0) | 5.572(1.246, 15.568) | -1.603(-3.874,0.721) | -2.616(-2.864, -2.368) |
| Saudi Arabia | 1(0,2) | 1(0,1) | -0.246(-0.647, 0.768) | -4.453(-5.298,-3.600) | -0.067(-0.377, 0.243) |
| Syrian Arab Republic | 4(1,9) | 6(2,15) | 0.452(-0.541, 2.637) | 3.477(1.277,5.724) | 0.937(0.63, 1.245) |
| Tunisia | 3(1,6) | 2(1,4) | -0.247(-0.636, 0.896) | -1.825(-1.944,-1.705) | 0.881(0.581, 1.181) |
| Türkiye | 274(95,786) | 65(21,174) | -0.764(-0.9, -0.392) | -5.437(-5.595,-5.279) | -0.855(-1.32, -0.389) |
| United Arab Emirates | 31(7,88) | 33(11,82) | 0.065(-0.597, 3.143) | -4.704(-5.227,-4.178) | -1.395(-1.624, -1.166) |
| Yemen | 9(1,25) | 18(5,39) | 1.004(-0.158, 4.66) | -0.729(-1.036,-0.422) | 0.908(0.644, 1.172) |
| Afghanistan | 10(1,29) | 25(5,58) | 1.536(0.298, 5.836) | -0.849(-1.381,-0.314) | -0.848(-2.081, 0.386) |
| Bangladesh | 18463(4082,38686) | 628(212,1197) | -0.966(-0.987, -0.896) | -14.381(-15.546,-13.200) | 2.351(1.964, 2.737) |
| Bhutan | 429(71,922) | 66(24,136) | -0.845(-0.926, -0.534) | -7.122(-7.514,-6.729) | 0.084(-0.169, 0.338) |
| India | 760115(566591,973214) | 195116(118441,280802) | -0.743(-0.822, -0.64) | -6.434(-6.737,-6.131) | -1.766(-1.972, -1.559) |
| Nepal | 65820(32065,110905) | 25073(13901,39872) | -0.619(-0.796, -0.259) | -4.779(-5.000,-4.558) | -0.012(-0.238, 0.213) |
| Pakistan | 83585(42393,141069) | 36566(22664,55665) | -0.563(-0.738, -0.23) | -5.406(-5.664,-5.147) | 1.765(1.472, 2.058) |
| Angola | 299(93,860) | 289(57,931) | -0.036(-0.677, 1.164) | -3.683(-3.936,-3.430) | -0.141(-0.424, 0.141) |
| Central African Republic | 71(13,236) | 95(17,305) | 0.338(-0.383, 1.799) | -1.349(-1.500,-1.198) | -13.08(-15.839, -10.322) |
| Congo | 27(6,87) | 23(3,73) | -0.171(-0.702, 0.647) | -3.007(-3.192,-2.822) | 0.128(-0.15, 0.407) |
| Democratic Republic of the Congo | 701(130,2318) | 933(177,3015) | 0.331(-0.451, 1.897) | -1.524(-1.829,-1.218) | -1.77(-2.452, -1.089) |
| Equatorial Guinea | 9(1,29) | 8(2,23) | -0.206(-0.749, 2.342) | -5.864(-6.199,-5.527) | 5.328(3.393, 7.263) |
| Gabon | 11(3,32) | 7(1,21) | -0.378(-0.757, 0.262) | -3.064(-3.214,-2.913) | 3.346(3.204, 3.489) |
| Burundi | 1970(903,3737) | 2561(574,7623) | 0.3(-0.6, 2.047) | -1.859(-2.167,-1.551) | 0.041(-0.156, 0.237) |
| Comoros | 116(41,237) | 86(25,204) | -0.26(-0.652, 0.624) | -2.621(-3.070,-2.169) | 1.377(1.039, 1.715) |
| Djibouti | 55(14,149) | 110(32,281) | 1.003(0.01, 3.26) | -1.279(-1.746,-0.810) | -0.133(-0.465, 0.199) |
| Eritrea | 1180(624,2064) | 1165(451,2316) | -0.013(-0.53, 0.973) | -1.995(-2.143,-1.846) | -1.073(-1.344, -0.802) |
| Ethiopia | 117132(35428,279678) | 68521(20502,183503) | -0.415(-0.747, 0.16) | -4.460(-4.695,-4.224) | -0.89(-1.092, -0.689) |
| Kenya | 5506(2320,10988) | 5460(2204,9726) | -0.008(-0.388, 0.608) | -1.669(-2.084,-1.253) | -0.194(-0.355, -0.033) |
| Madagascar | 2002(953,3488) | 3388(1409,6530) | 0.692(-0.24, 2.126) | -0.822(-1.202,-0.441) | -1.515(-2.016, -1.014) |
| Malawi | 4859(2309,9192) | 4563(1818,8664) | -0.061(-0.607, 1.125) | -2.353(-2.680,-2.025) | -0.956(-1.124, -0.789) |
| Mauritius | 1(1,2) | 0(0,0) | -0.992(-0.994, -0.99) | -11.325(-13.544,-9.048) | 1.894(0.95, 2.838) |
| Mozambique | 8007(4051,13780) | 8228(3013,16697) | 0.028(-0.618, 1.034) | -2.114(-2.329,-1.898) | 1.678(1.289, 2.066) |
| Rwanda | 2948(1289,6598) | 1755(291,5950) | -0.405(-0.832, 0.35) | -4.403(-4.861,-3.942) | 0.631(0.412, 0.85) |
| Seychelles | 0(0,0) | 0(0,0) | 2.251(0.521, 5.61) | 2.094(-0.001,4.232) | 1.902(1.571, 2.234) |
| Somalia | 2655(498,7302) | 6962(1248,20198) | 1.622(0.005, 4.358) | -0.023(-0.280,0.234) | -1.665(-1.974, -1.357) |
| United Republic of Tanzania | 10587(4914,19193) | 10657(3338,24183) | 0.007(-0.662, 1.213) | -2.366(-2.632,-2.099) | 2.567(2.315, 2.819) |
| Uganda | 3401(1243,7310) | 5184(1700,10517) | 0.524(-0.344, 2.63) | -1.897(-2.221,-1.571) | 1.208(0.74, 1.676) |
| Zambia | 3718(2095,6165) | 3405(1353,6352) | -0.084(-0.608, 1.103) | -3.159(-3.670,-2.646) | 1.283(0.571, 1.994) |
| Botswana | 70(28,155) | 50(24,96) | -0.289(-0.706, 0.71) | -2.858(-2.999,-2.716) | 0.429(0.136, 0.721) |
| Lesotho | 102(41,212) | 77(34,147) | -0.248(-0.688, 0.858) | -0.882(-1.243,-0.520) | 0.326(-0.042, 0.695) |
| Namibia | 61(28,117) | 57(23,117) | -0.064(-0.582, 1.03) | -1.941(-2.047,-1.835) | -0.13(-0.663, 0.404) |
| South Africa | 1164(637,1949) | 702(402,1099) | -0.397(-0.65, 0.052) | -3.236(-3.930,-2.537) | 0.046(-0.759, 0.851) |
| Eswatini | 53(24,105) | 38(18,75) | -0.275(-0.683, 0.642) | -1.855(-1.958,-1.752) | 1.342(0.996, 1.688) |
| Zimbabwe | 526(255,903) | 924(473,1948) | 0.757(-0.187, 2.759) | 1.241(0.643,1.843) | -0.697(-0.982, -0.411) |
| Benin | 1541(432,3929) | 2611(751,5861) | 0.694(-0.361, 3.554) | -1.377(-1.531,-1.223) | 1.701(1.076, 2.325) |
| Burkina Faso | 4398(1343,9546) | 5303(1541,12154) | 0.206(-0.507, 1.771) | -2.351(-2.586,-2.115) | 1.071(0.899, 1.242) |
| Cameroon | 2365(700,5185) | 4307(1515,8934) | 0.821(-0.223, 3.732) | -1.238(-1.486,-0.989) | -0.808(-1.213, -0.403) |
| Cabo Verde | 49(11,122) | 28(9,57) | -0.426(-0.711, 0.43) | -3.792(-4.073,-3.509) | -0.058(-0.511, 0.396) |
| Chad | 2436(435,6444) | 5237(759,14675) | 1.15(-0.102, 3.684) | -0.842(-0.959,-0.725) | 0.447(0.16, 0.733) |
| Côte d'Ivoire | 2868(715,6725) | 4141(1132,9196) | 0.444(-0.35, 2.46) | -0.984(-1.273,-0.694) | -0.105(-0.332, 0.122) |
| Gambia | 245(56,620) | 360(113,852) | 0.47(-0.356, 2.767) | -1.643(-1.930,-1.356) | 0.853(0.366, 1.339) |
| Ghana | 7008(3096,13136) | 7803(3134,14630) | 0.113(-0.446, 1.274) | -1.929(-2.208,-1.649) | -1.498(-2.042, -0.954) |
| Guinea | 2657(878,5761) | 2879(733,6389) | 0.084(-0.59, 1.34) | -1.749(-1.964,-1.534) | 0.216(-1.196, 1.629) |
| Guinea-Bissau | 397(115,991) | 378(94,896) | -0.047(-0.58, 1.072) | -1.914(-2.157,-1.671) | -1.018(-1.599, -0.438) |
| Liberia | 676(166,1452) | 689(124,1702) | 0.021(-0.65, 1.186) | -2.797(-3.121,-2.471) | -0.993(-1.881, -0.105) |
| Mali | 3712(604,10342) | 5252(1031,14049) | 0.415(-0.424, 2.139) | -1.964(-2.246,-1.682) | 1.501(1.127, 1.875) |
| Mauritania | 437(144,940) | 360(97,782) | -0.176(-0.637, 0.771) | -2.853(-3.081,-2.625) | -1.166(-1.489, -0.842) |
| Niger | 4893(1006,13240) | 8386(1244,24826) | 0.714(-0.51, 3.353) | -1.950(-2.177,-1.722) | -0.567(-1.276, 0.142) |
| Nigeria | 48435(27116,73578) | 67273(28399,114458) | 0.389(-0.397, 1.654) | -1.695(-2.016,-1.374) | 1.293(1.081, 1.504) |
| Sao Tome and Principe | 26(7,62) | 14(4,29) | -0.448(-0.77, 0.378) | -3.832(-4.106,-3.556) | 9.711(3.178, 16.243) |
| Senegal | 2903(925,6265) | 2260(660,5091) | -0.221(-0.651, 0.633) | -2.724(-2.974,-2.474) | -1.368(-1.968, -0.768) |
| Sierra Leone | 1382(491,3073) | 1353(331,3632) | -0.021(-0.642, 1.476) | -2.435(-2.739,-2.131) | -8.487(-9.899, -7.075) |
| Togo | 1041(416,2264) | 1188(405,2576) | 0.141(-0.484, 1.431) | -1.935(-2.085,-1.785) | 0.095(-0.393, 0.583) |
| American Samoa | 0(0,1) | 0(0,1) | -0.055(-0.618, 1.475) | 0.307(0.062,0.553) | -0.666(-0.736, -0.597) |
| Bermuda | 0(0,0) | 0(0,0) | 0.239(-0.22, 0.939) | 1.163(0.353,1.979) | -1.963(-2.415, -1.511) |
| Cook Islands | 0(0,0) | 0(0,0) | -0.397(-0.787, 1.35) | -1.736(-2.055,-1.416) | 2.024(1.432, 2.616) |
| Greenland | 0(0,0) | 0(0,0) | -0.223(-0.705, 0.876) | 0.160(-0.989,1.324) | 0.188(-0.108, 0.484) |
| Guam | 0(0,1) | 0(0,0) | -0.293(-0.606, 0.308) | -0.625(-1.025,-0.224) | 0.339(-0.158, 0.836) |
| Monaco | 0(0,0) | 0(0,0) | -0.33(-0.67, 0.211) | -1.629(-2.444,-0.807) | -2.242(-2.429, -2.056) |
| Nauru | 0(0,0) | 0(0,0) | 0.513(-0.271, 2.179) | 1.204(0.522,1.891) | -0.619(-0.709, -0.53) |
| Niue | 0(0,0) | 0(0,0) | -0.348(-0.762, 0.805) | -0.507(-0.758,-0.256) | -1.313(-1.478, -1.148) |
| Northern Mariana Islands | 0(0,1) | 0(0,1) | -0.088(-0.572, 0.896) | -0.283(-0.577,0.012) | -1.383(-1.869, -0.897) |
| Palau | 0(0,0) | 0(0,0) | 0.446(-0.372, 2.157) | 0.521(0.435,0.606) | -0.896(-1.038, -0.754) |
| Puerto Rico | 0(0,0) | 0(0,0) | 7.938(4.644, 11.239) | 6.768(3.299,10.353) | 9.975(7.627, 12.323) |
| Saint Kitts and Nevis | 0(0,0) | 0(0,0) | -0.374(-0.566, -0.079) | -2.259(-2.552,-1.965) | -1.229(-1.272, -1.186) |
| San Marino | 0(0,0) | 0(0,0) | -0.971(-0.989, -0.93) | -16.874(-19.369,-14.302) | -12.333(-13.742, -10.924) |
| Tokelau | 0(0,0) | 0(0,0) | -0.096(-0.614, 1.255) | -0.525(-0.994,-0.054) | -1.871(-2.112, -1.63) |
| Tuvalu | 0(0,0) | 0(0,0) | -0.187(-0.661, 1.096) | -1.534(-1.697,-1.372) | -2.923(-2.967, -2.88) |
| United States Virgin Islands | 0(0,0) | 0(0,0) | -0.477(-0.754, 0.096) | -0.650(-0.949,-0.350) | -2.03(-2.284, -1.776) |
| South Sudan | 1145(243,2635) | 1997(425,4722) | 0.744(-0.26, 2.576) | 0.190(-0.180,0.561) | 0.149(-0.032, 0.33) |
| Sudan | 17(5,34) | 18(4,38) | 0.062(-0.533, 1.467) | -2.109(-2.411,-1.806) | -2.38(-2.453, -2.307) |

Abbreviations: AAPC, average annual percent change; CI, confidence interval; DALYs, disability-adjusted life years; EAPC, estimated annual percentage change; UI, uncertainty interval.


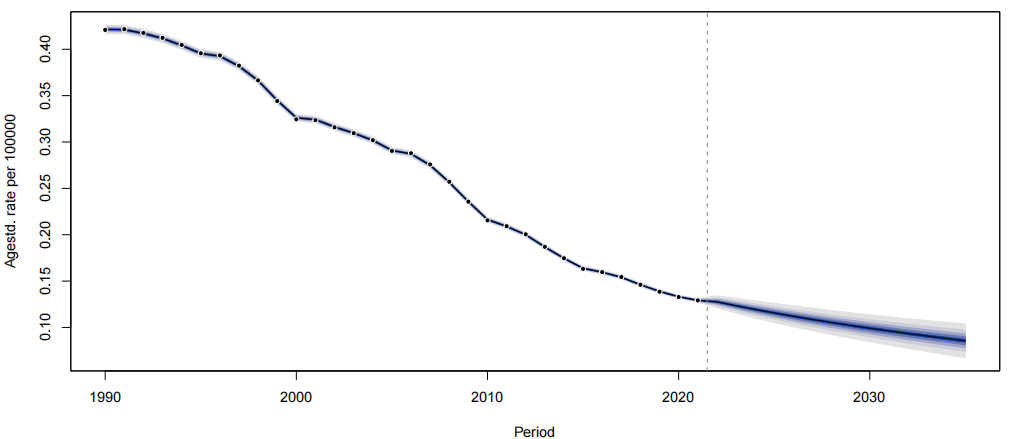


A


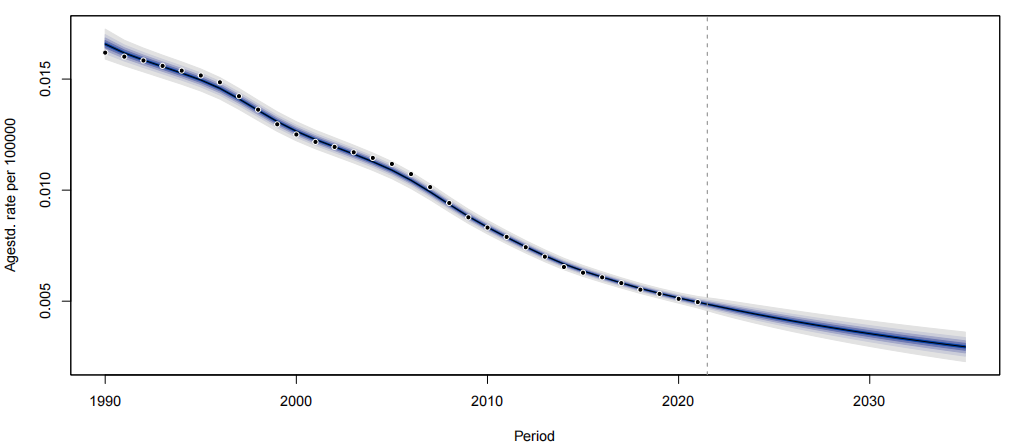


B


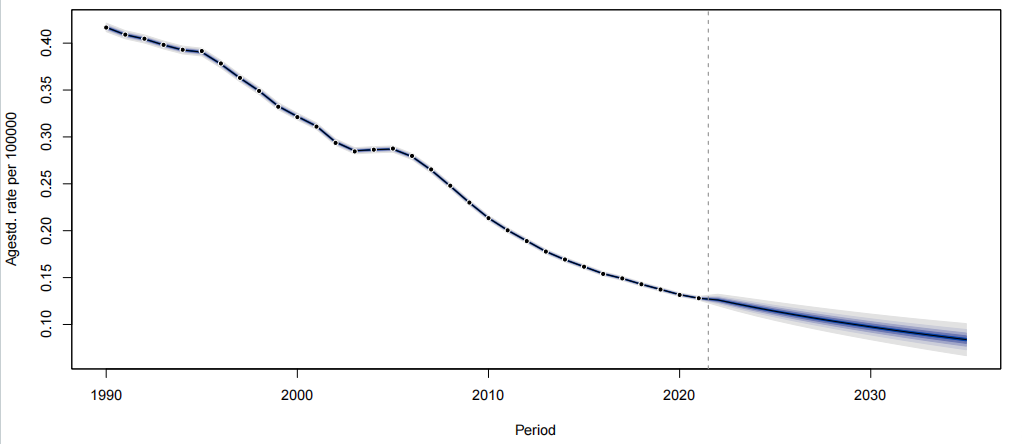


C


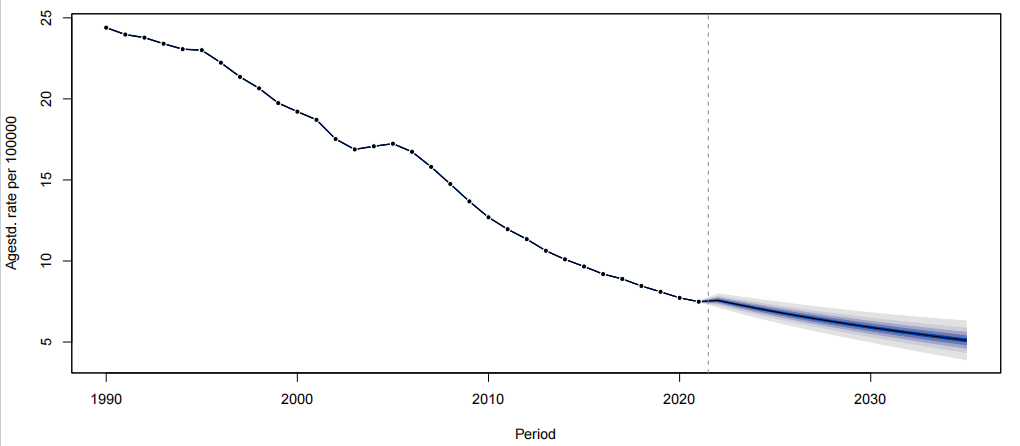


D

Supplementary Figure 1: Prediction of global disease burden of rabies from 2022–2035 years based on the BAPC model (A: ASIR. B: ASPR. C: ASMR. D: ASDR. Abbreviations: ASIR, age-standardized incidence rate; ASMR, age-standardized mortality rate; ASPR, age-standardized prevalence rate; ASDR, age standardized Disability-adjusted life year rate; CI, confidence Interval).

Supplementary Table 13: A decomposition analysis was conducted on the number of DALYs attributable to rabies, examining trends globally and within five SDI regions from 1990 to 2021.

| Location | Overall Difference | Aging | Population | Epidemiological Change | Aging  Percentage | Population Percentage |
| --- | --- | --- | --- | --- | --- | --- |
| Global | -799230.151 | -89233.242 | 414618.667 | -1124615.561 | 11.165 | -51.885 |
| Middle SDI | -130064.922 | -17243.851 | 65210.327 | -178031.395 | 13.267 | -50.145 |
| Low-middle SDI | -491249.121 | 27651.612 | 200215.86 | -719116.525 | -5.635 | -40.767 |
| High SDI | 170.062 | -160.154 | 242.687 | 87.546 | -94.175 | 142.75 |
| High-middle SDI | -10852.412 | -4294.513 | 6401.466 | -12959.375 | 39.577 | -58.997 |
| Low SDI | -167130.151 | 149573.332 | 159387.645 | -476091.125 | -89.55 | -95.375 |

Abbreviations: DALYs, Disability-Adjusted Life Years; SDI, sociodemographic index.

Supplementary Table 14: Frontier analysis exploring ASDR of rabies across 204 countries and territories.

| Id | year | val | SDI | frontier | eff_diff | location_name |
| --- | --- | --- | --- | --- | --- | --- |
| 6 | 2021 | 1.818539993 | 0.72162976 | 0.000296647 | 1.818243346 | China |
| 7 | 2021 | 2.833427708 | 0.569854634 | 0.000414149 | 2.833013559 | Korea |
| 8 | 2021 | 0.002834044 | 0.874747053 | 0.000243422 | 0.002590622 | Taiwan (Province of China) |
| 10 | 2021 | 8.227975524 | 0.473621491 | 0.000558958 | 8.227416566 | Cambodia |
| 11 | 2021 | 1.239592255 | 0.656868336 | 0.000405766 | 1.239186488 | Indonesia |
| 12 | 2021 | 9.200167668 | 0.489136091 | 0.000517688 | 9.19964998 | Lao People's Democratic Republic |
| 13 | 2021 | 0.090023309 | 0.742523828 | 0.000264175 | 0.089759134 | Malaysia |
| 14 | 2021 | 0.047232446 | 0.650886627 | 0.000410385 | 0.046822061 | Maldives |
| 15 | 2021 | 23.44884464 | 0.53390084 | 0.000449687 | 23.44839495 | Myanmar |
| 16 | 2021 | 12.20972112 | 0.651219329 | 0.000412568 | 12.20930855 | Philippines |
| 17 | 2021 | 3.68644499 | 0.701534935 | 0.000336667 | 3.686108323 | Sri Lanka |
| 18 | 2021 | 0.31118341 | 0.682547933 | 0.000401854 | 0.310781556 | Thailand |
| 19 | 2021 | 9.047805726 | 0.444667619 | 0.000734141 | 9.047071585 | Timor-Leste |
| 20 | 2021 | 2.740958067 | 0.627933721 | 0.000400103 | 2.740557964 | Viet Nam |
| 22 | 2021 | 0.191526868 | 0.675051631 | 0.000410021 | 0.191116847 | Fiji |
| 23 | 2021 | 0.859536067 | 0.527186583 | 0.000446414 | 0.859089652 | Kiribati |
| 24 | 2021 | 0.37196756 | 0.574091128 | 0.000408264 | 0.371559296 | Marshall Islands |
| 25 | 2021 | 0.476691542 | 0.587534967 | 0.000408288 | 0.476283254 | Micronesia |
| 26 | 2021 | 0.656358303 | 0.417797443 | 0.000917541 | 0.655440762 | Papua New Guinea |
| 27 | 2021 | 0.48333431 | 0.593392769 | 0.000409337 | 0.482924973 | Samoa |
| 28 | 2021 | 0.6022709 | 0.429360316 | 0.000837358 | 0.601433542 | Solomon Islands |
| 29 | 2021 | 0.326760445 | 0.626349936 | 0.000408259 | 0.326352186 | Tonga |
| 30 | 2021 | 0.484419293 | 0.473100706 | 0.000552449 | 0.483866843 | Vanuatu |
| 33 | 2021 | 0.001733155 | 0.701833194 | 0.000325018 | 0.001408138 | Armenia |
| 34 | 2021 | 0.843328966 | 0.694851274 | 0.000360722 | 0.842968244 | Azerbaijan |
| 35 | 2021 | 0.836869633 | 0.732473604 | 0.000284977 | 0.836584656 | Georgia |
| 36 | 2021 | 0.928338249 | 0.725144495 | 0.00030303 | 0.928035219 | Kazakhstan |
| 37 | 2021 | 0.36358501 | 0.603979328 | 0.000406552 | 0.363178458 | Kyrgyzstan |
| 38 | 2021 | 3.588368909 | 0.617621565 | 0.000405522 | 3.587963387 | Mongolia |
| 39 | 2021 | 0.013980717 | 0.541511187 | 0.000434657 | 0.01354606 | Tajikistan |
| 40 | 2021 | 0.041762829 | 0.682160776 | 0.000402062 | 0.041360767 | Turkmenistan |
| 41 | 2021 | 0.189502686 | 0.662621694 | 0.000407839 | 0.189094848 | Uzbekistan |
| 43 | 2021 | 0.000963641 | 0.706849791 | 0.000320492 | 0.00064315 | Albania |
| 44 | 2021 | 0.000377529 | 0.723077893 | 0.000300315 | 7.72138E-05 | Bosnia and Herzegovina |
| 45 | 2021 | 0.013778852 | 0.768150939 | 0.000250569 | 0.013528283 | Bulgaria |
| 46 | 2021 | 0.000260123 | 0.798341027 | 0.000243467 | 1.66555E-05 | Croatia |
| 47 | 2021 | 0.000230299 | 0.828450433 | 0.000229518 | 7.81402E-07 | Czechia |
| 48 | 2021 | 0.005983345 | 0.790754768 | 0.000253761 | 0.005729584 | Hungary |
| 49 | 2021 | 0.00078858 | 0.750629703 | 0.000257478 | 0.000531102 | North Macedonia |
| 50 | 2021 | 0.014039678 | 0.795800584 | 0.000247522 | 0.013792156 | Montenegro |
| 51 | 2021 | 0.000312993 | 0.812042809 | 0.000256223 | 5.67699E-05 | Poland |
| 52 | 2021 | 0.001081378 | 0.768453864 | 0.000253962 | 0.000827416 | Romania |
| 53 | 2021 | 0.001162228 | 0.792416294 | 0.000253364 | 0.000908864 | Serbia |
| 54 | 2021 | 0.006680599 | 0.81061053 | 0.000247043 | 0.006433556 | Slovakia |
| 55 | 2021 | 0.003879985 | 0.842430731 | 0.000239155 | 0.00364083 | Slovenia |
| 57 | 2021 | 0.220203666 | 0.784484711 | 0.000255881 | 0.219947785 | Belarus |
| 58 | 2021 | 0.005372928 | 0.844917787 | 0.000239565 | 0.005133363 | Estonia |
| 59 | 2021 | 0.103697552 | 0.830663516 | 0.000241255 | 0.103456298 | Latvia |
| 60 | 2021 | 0.000528239 | 0.856484049 | 0.000243617 | 0.000284622 | Lithuania |
| 61 | 2021 | 0.007467585 | 0.732214875 | 0.00028665 | 0.007180935 | Republic of Moldova |
| 62 | 2021 | 0.065533296 | 0.808536005 | 0.000252927 | 0.065280369 | Russian Federation |
| 63 | 2021 | 0.117085639 | 0.760773913 | 0.000254263 | 0.116831376 | Ukraine |
| 66 | 2021 | 0.000378426 | 0.810234367 | 0.000246236 | 0.00013219 | Brunei Darussalam |
| 67 | 2021 | 0.003749975 | 0.871241813 | 0.000244554 | 0.003505421 | Japan |
| 68 | 2021 | 0.000305366 | 0.886675267 | 0.000239079 | 6.62868E-05 | Republic of Korea |
| 69 | 2021 | 0.000584959 | 0.856097766 | 0.000237807 | 0.000347152 | Singapore |
| 71 | 2021 | 0.022062193 | 0.844252814 | 0.000240662 | 0.021821532 | Australia |
| 72 | 2021 | 0.004541061 | 0.849442499 | 0.000240289 | 0.004300772 | New Zealand |
| 74 | 2021 | 0.001385407 | 0.869444113 | 0.000241291 | 0.001144115 | Andorra |
| 75 | 2021 | 0.004708 | 0.853837004 | 0.000242159 | 0.004465841 | Austria |
| 76 | 2021 | 0.00382599 | 0.853654016 | 0.000243438 | 0.003582553 | Belgium |
| 77 | 2021 | 0.006181152 | 0.835630545 | 0.000240204 | 0.005940948 | Cyprus |
| 78 | 2021 | 0.003527909 | 0.896424204 | 0.000240167 | 0.003287742 | Denmark |
| 79 | 2021 | 0.021883359 | 0.859831368 | 0.000239106 | 0.021644253 | Finland |
| 80 | 2021 | 0.000348269 | 0.838364875 | 0.000244515 | 0.000103754 | France |
| 81 | 2021 | 0.004607341 | 0.902957091 | 0.000240643 | 0.004366699 | Germany |
| 82 | 2021 | 0.021075794 | 0.791854408 | 0.00025108 | 0.020824714 | Greece |
| 83 | 2021 | 0.018025911 | 0.87636168 | 0.000240552 | 0.017785359 | Iceland |
| 84 | 2021 | 0.002846772 | 0.87375385 | 0.000241756 | 0.002605016 | Ireland |
| 85 | 2021 | 0.000588439 | 0.809011652 | 0.000251966 | 0.000336473 | Israel |
| 86 | 2021 | 0.000455099 | 0.805773534 | 0.000249547 | 0.000205552 | Italy |
| 87 | 2021 | 0.003481817 | 0.884428955 | 0.000242275 | 0.003239542 | Luxembourg |
| 88 | 2021 | 0.001589619 | 0.801585034 | 0.00025323 | 0.001336389 | Malta |
| 89 | 2021 | 0.007105285 | 0.888464256 | 0.00023836 | 0.006866925 | Netherlands |
| 90 | 2021 | 0.147202949 | 0.91613281 | 0.000243057 | 0.146959891 | Norway |
| 91 | 2021 | 0.000302036 | 0.744151851 | 0.000262442 | 3.95936E-05 | Portugal |
| 92 | 2021 | 0.000268482 | 0.769283698 | 0.000249987 | 1.84951E-05 | Spain |
| 93 | 2021 | 0.017061771 | 0.886880299 | 0.000240352 | 0.016821419 | Sweden |
| 94 | 2021 | 0.000960481 | 0.933059111 | 0.000241264 | 0.000719216 | Switzerland |
| 95 | 2021 | 0.046031918 | 0.859000182 | 0.000241239 | 0.045790678 | United Kingdom |
| 97 | 2021 | 0.000383846 | 0.723122973 | 0.000300706 | 8.31396E-05 | Argentina |
| 98 | 2021 | 0.000904969 | 0.771514716 | 0.000254579 | 0.00065039 | Chile |
| 99 | 2021 | 0.002151063 | 0.719283445 | 0.000296739 | 0.001854324 | Uruguay |
| 101 | 2021 | 0.028678862 | 0.87317068 | 0.000240593 | 0.028438269 | Canada |
| 102 | 2021 | 0.128062572 | 0.862448354 | 0.000242156 | 0.127820416 | United States of America |
| 105 | 2021 | 0.076526951 | 0.749886887 | 0.000259319 | 0.076267632 | Antigua and Barbuda |
| 106 | 2021 | 0.054513848 | 0.805020668 | 0.000249188 | 0.05426466 | Bahamas |
| 107 | 2021 | 0.146642582 | 0.746748764 | 0.000265744 | 0.146376839 | Barbados |
| 108 | 2021 | 0.01239559 | 0.610229002 | 0.000407779 | 0.01198781 | Belize |
| 109 | 2021 | 0.006039381 | 0.668729864 | 0.000404744 | 0.005634637 | Cuba |
| 110 | 2021 | 0.075770732 | 0.746967185 | 0.00026446 | 0.075506272 | Dominica |
| 111 | 2021 | 0.112823766 | 0.619388201 | 0.000406063 | 0.112417703 | Dominican Republic |
| 112 | 2021 | 0.140897194 | 0.668993028 | 0.000408591 | 0.140488603 | Grenada |
| 113 | 2021 | 0.008873869 | 0.650812335 | 0.000410462 | 0.008463407 | Guyana |
| 114 | 2021 | 0.05449984 | 0.448278285 | 0.000679876 | 0.053819964 | Haiti |
| 115 | 2021 | 0.00682765 | 0.683263064 | 0.000401977 | 0.006425673 | Jamaica |
| 116 | 2021 | 0.009479505 | 0.672509735 | 0.000406562 | 0.009072943 | Saint Lucia |
| 117 | 2021 | 0.014218441 | 0.637195963 | 0.000410198 | 0.013808243 | Saint Vincent and the Grenadines |
| 118 | 2021 | 0.010164155 | 0.633665739 | 0.000404891 | 0.009759264 | Suriname |
| 119 | 2021 | 0.012345788 | 0.768763254 | 0.0002553 | 0.012090488 | Trinidad and Tobago |
| 121 | 2021 | 0.01146719 | 0.599010799 | 0.00041259 | 0.011054599 | Bolivia (Plurinational State of) |
| 122 | 2021 | 0.000536421 | 0.661017053 | 0.00040586 | 0.00013056 | Ecuador |
| 123 | 2021 | 0.002538551 | 0.662054037 | 0.000407411 | 0.002131139 | Peru |
| 125 | 2021 | 0.000678685 | 0.655442913 | 0.000411775 | 0.00026691 | Colombia |
| 126 | 2021 | 0.003838374 | 0.700340477 | 0.000351268 | 0.003487106 | Costa Rica |
| 127 | 2021 | 0.002924644 | 0.563775188 | 0.000405674 | 0.002518969 | El Salvador |
| 128 | 2021 | 0.006936596 | 0.539972424 | 0.00043985 | 0.006496746 | Guatemala |
| 129 | 2021 | 0.000431099 | 0.513037248 | 0.000431099 | 0 | Honduras |
| 130 | 2021 | 0.001264098 | 0.664575304 | 0.000412133 | 0.000851965 | Mexico |
| 131 | 2021 | 0.000532583 | 0.523958472 | 0.000448868 | 8.37148E-05 | Nicaragua |
| 132 | 2021 | 0.004534079 | 0.708864828 | 0.00030732 | 0.004226759 | Panama |
| 133 | 2021 | 0.001079093 | 0.596513059 | 0.000406741 | 0.000672352 | Venezuela |
| 135 | 2021 | 0.009601773 | 0.653043887 | 0.00040942 | 0.009192353 | Brazil |
| 136 | 2021 | 0.001478613 | 0.635718099 | 0.000406642 | 0.001071971 | Paraguay |
| 139 | 2021 | 0.020782716 | 0.659500924 | 0.000404715 | 0.020378 | Algeria |
| 140 | 2021 | 0.082636992 | 0.753043204 | 0.000262149 | 0.082374843 | Bahrain |
| 141 | 2021 | 0.421115302 | 0.606787094 | 0.000407948 | 0.420707354 | Egypt |
| 142 | 2021 | 0.056067518 | 0.697207398 | 0.000343827 | 0.055723691 | Iran (Islamic Republic of) |
| 143 | 2021 | 0.323445582 | 0.662626231 | 0.000404959 | 0.323040623 | Iraq |
| 144 | 2021 | 0.001138057 | 0.725307227 | 0.000289559 | 0.000848497 | Jordan |
| 145 | 2021 | 0.022861072 | 0.846651055 | 0.000242104 | 0.022618968 | Kuwait |
| 146 | 2021 | 0.001407501 | 0.744746351 | 0.000258498 | 0.001149003 | Lebanon |
| 147 | 2021 | 0.029311778 | 0.725771399 | 0.000291722 | 0.029020056 | Libya |
| 148 | 2021 | 0.026873049 | 0.562698301 | 0.000408902 | 0.026464147 | Morocco |
| 149 | 2021 | 0.011962419 | 0.631011665 | 0.000411803 | 0.011550616 | Palestine |
| 150 | 2021 | 0.000368352 | 0.773391602 | 0.000255632 | 0.000112721 | Oman |
| 151 | 2021 | 0.001292942 | 0.846860584 | 0.0002418 | 0.001051142 | Qatar |
| 152 | 2021 | 0.001824814 | 0.815143493 | 0.000251803 | 0.001573011 | Saudi Arabia |
| 153 | 2021 | 0.059452109 | 0.623004075 | 0.000403657 | 0.059048451 | Syrian Arab Republic |
| 154 | 2021 | 0.017177216 | 0.682432216 | 0.000402633 | 0.016774583 | Tunisia |
| 155 | 2021 | 0.077974439 | 0.712692673 | 0.000311546 | 0.077662893 | Turkey |
| 156 | 2021 | 0.710692316 | 0.849317734 | 0.000241089 | 0.710451227 | United Arab Emirates |
| 157 | 2021 | 0.062968407 | 0.450376375 | 0.000694045 | 0.062274363 | Yemen |
| 160 | 2021 | 0.086866715 | 0.337199998 | 0.01661625 | 0.070250465 | Afghanistan |
| 161 | 2021 | 0.474227765 | 0.492420885 | 0.000509713 | 0.473718052 | Bangladesh |
| 162 | 2021 | 9.300604383 | 0.473062378 | 0.000555123 | 9.30004926 | Bhutan |
| 163 | 2021 | 14.04721593 | 0.575401649 | 0.00040637 | 14.04680956 | India |
| 164 | 2021 | 81.65786468 | 0.433174635 | 0.000837755 | 81.65702693 | Nepal |
| 165 | 2021 | 15.05837969 | 0.504028689 | 0.000494909 | 15.05788478 | Pakistan |
| 168 | 2021 | 0.792402996 | 0.453721949 | 0.000648355 | 0.791754641 | Angola |
| 169 | 2021 | 1.596178659 | 0.30916769 | 0.078548259 | 1.5176304 | Central African Republic |
| 170 | 2021 | 0.40777731 | 0.583075236 | 0.000409859 | 0.407367451 | Congo |
| 171 | 2021 | 0.982215313 | 0.383179849 | 0.00118664 | 0.981028672 | Congo |
| 172 | 2021 | 0.473955536 | 0.657857456 | 0.000406051 | 0.473549485 | Equatorial Guinea |
| 173 | 2021 | 0.368725472 | 0.634691393 | 0.000408765 | 0.368316707 | Gabon |
| 175 | 2021 | 17.88684917 | 0.289374365 | 0.078595497 | 17.80825368 | Burundi |
| 176 | 2021 | 11.08915596 | 0.475978688 | 0.000551417 | 11.08860455 | Comoros |
| 177 | 2021 | 8.423284164 | 0.487958371 | 0.000516229 | 8.422767935 | Djibouti |
| 178 | 2021 | 16.64597528 | 0.403863943 | 0.001009825 | 16.64496546 | Eritrea |
| 179 | 2021 | 58.03136175 | 0.358823295 | 0.001475514 | 58.02988624 | Ethiopia |
| 180 | 2021 | 10.34471565 | 0.523768077 | 0.000448484 | 10.34426716 | Kenya |
| 181 | 2021 | 10.9274521 | 0.400246943 | 0.001034557 | 10.92641754 | Madagascar |
| 182 | 2021 | 25.45333507 | 0.384553634 | 0.001189845 | 25.45214522 | Malawi |
| 183 | 2021 | 0.000745874 | 0.718260446 | 0.000288366 | 0.000457508 | Mauritius |
| 184 | 2021 | 23.7977744 | 0.326462614 | 0.07853998 | 23.71923442 | Mozambique |
| 185 | 2021 | 12.41752898 | 0.435588706 | 0.000779612 | 12.41674937 | Rwanda |
| 186 | 2021 | 0.033523751 | 0.730150775 | 0.000274248 | 0.033249503 | Seychelles |
| 187 | 2021 | 31.77804659 | 0.077688109 | 30.56962081 | 1.208425781 | Somalia |
| 189 | 2021 | 15.6793842 | 0.446568273 | 0.000696933 | 15.67868727 | United Republic of Tanzania |
| 190 | 2021 | 10.67077108 | 0.423261181 | 0.000866606 | 10.66990448 | Uganda |
| 191 | 2021 | 16.02244108 | 0.505948954 | 0.000478533 | 16.02196254 | Zambia |
| 193 | 2021 | 2.047880749 | 0.642721629 | 0.000406617 | 2.047474132 | Botswana |
| 194 | 2021 | 3.711139047 | 0.510393066 | 0.00045997 | 3.710679077 | Lesotho |
| 195 | 2021 | 2.129226686 | 0.617564872 | 0.000406309 | 2.128820377 | Namibia |
| 196 | 2021 | 1.275130839 | 0.679626598 | 0.000409113 | 1.274721725 | South Africa |
| 197 | 2021 | 2.917932671 | 0.585459713 | 0.000407746 | 2.917524925 | Eswatini |
| 198 | 2021 | 5.787444795 | 0.473819486 | 0.000560282 | 5.786884513 | Zimbabwe |
| 200 | 2021 | 15.70540001 | 0.373486574 | 0.001347818 | 15.70405219 | Benin |
| 201 | 2021 | 18.57571347 | 0.285118402 | 0.078595549 | 18.49711792 | Burkina Faso |
| 202 | 2021 | 11.78632553 | 0.479691223 | 0.000546449 | 11.78577908 | Cameroon |
| 203 | 2021 | 5.196160088 | 0.533534539 | 0.000451095 | 5.195708993 | Cabo Verde |
| 204 | 2021 | 22.81305065 | 0.240436019 | 0.079008754 | 22.73404189 | Chad |
| 205 | 2021 | 12.89666776 | 0.425941883 | 0.0008774 | 12.89579036 | Côte d'Ivoire |
| 206 | 2021 | 13.43121574 | 0.40971416 | 0.000947108 | 13.43026863 | Gambia |
| 207 | 2021 | 21.68558163 | 0.56493039 | 0.000410619 | 21.68517101 | Ghana |
| 208 | 2021 | 18.13626954 | 0.336401293 | 0.039078966 | 18.09719057 | Guinea |
| 209 | 2021 | 16.91082002 | 0.353109621 | 0.003572972 | 16.90724705 | Guinea-Bissau |
| 210 | 2021 | 11.28413161 | 0.352442452 | 0.003210263 | 11.28092135 | Liberia |
| 211 | 2021 | 18.40145684 | 0.268579941 | 0.078554595 | 18.32290225 | Mali |
| 212 | 2021 | 7.253824237 | 0.4989451 | 0.000493512 | 7.253330725 | Mauritania |
| 213 | 2021 | 26.72832277 | 0.168072774 | 26.28833226 | 0.439990513 | Niger |
| 214 | 2021 | 22.39787083 | 0.503390833 | 0.000491795 | 22.39737903 | Nigeria |
| 215 | 2021 | 6.377830731 | 0.505413747 | 0.000478664 | 6.377352066 | Sao Tome and Principe |
| 216 | 2021 | 12.90144887 | 0.408054193 | 0.000957384 | 12.90049149 | Senegal |
| 217 | 2021 | 13.45449737 | 0.358665881 | 0.001685621 | 13.45281175 | Sierra Leone |
| 218 | 2021 | 13.03279133 | 0.408533695 | 0.000958892 | 13.03183244 | Togo |
| 298 | 2021 | 0.456468944 | 0.723727533 | 0.000299516 | 0.456169428 | American Samoa |
| 305 | 2021 | 0.050619625 | 0.821365422 | 0.000251529 | 0.050368096 | Bermuda |
| 320 | 2021 | 0.556326419 | 0.779109955 | 0.000250679 | 0.55607574 | Cook Islands |
| 349 | 2021 | 0.081763249 | 0.826210336 | 0.000246527 | 0.081516722 | Greenland |
| 351 | 2021 | 0.161490942 | 0.803982203 | 0.000254826 | 0.161236117 | Guam |
| 367 | 2021 | 0.064195704 | 0.908262831 | 0.000240531 | 0.063955174 | Monaco |
| 369 | 2021 | 0.506073743 | 0.625177834 | 0.000407632 | 0.505666112 | Nauru |
| 374 | 2021 | 0.202041677 | 0.72622205 | 0.00028788 | 0.201753797 | Niue |
| 376 | 2021 | 0.882606288 | 0.771535213 | 0.000253868 | 0.88235242 | Northern Mariana Islands |
| 380 | 2021 | 0.047332093 | 0.754046931 | 0.000263084 | 0.04706901 | Palau |
| 385 | 2021 | 0.004582856 | 0.825525847 | 0.00024868 | 0.004334176 | Puerto Rico |
| 393 | 2021 | 0.340452116 | 0.754987055 | 0.000258635 | 0.340193481 | Saint Kitts and Nevis |
| 396 | 2021 | 0.001585111 | 0.888005474 | 0.000242733 | 0.001342378 | San Marino |
| 413 | 2021 | 0.242009028 | 0.686425621 | 0.000392484 | 0.241616544 | Tokelau |
| 416 | 2021 | 0.46711267 | 0.576620529 | 0.000406414 | 0.466706257 | Tuvalu |
| 422 | 2021 | 0.029977901 | 0.821830853 | 0.000249731 | 0.029728171 | United States Virgin Islands |
| 435 | 2021 | 17.8585718 | 0.278371125 | 0.078509202 | 17.7800626 | South Sudan |
| 522 | 2021 | 0.042307757 | 0.541949735 | 0.00043655 | 0.041871207 | Sudan |

Abbreviations: ASDR, age standardized Disability-adjusted life year rate; SDI, sociodemographic index.
